# Simvastatin Sodium Salt and Fluvastatin Interact with Human Gap Junction Gamma-3 Protein

**DOI:** 10.1371/journal.pone.0148266

**Published:** 2016-02-10

**Authors:** Andrew Marsh, Katherine Casey-Green, Fay Probert, David Withall, Daniel A. Mitchell, Suzanne J. Dilly, Sean James, Wade Dimitri, Sweta R. Ladwa, Paul C. Taylor, Donald R. J. Singer

**Affiliations:** 1 Tangent Reprofiling Ltd, c/o SEEK, Central Point, 45 Beech Street, London, EC2Y 8AD, United Kingdom; 2 University Hospital Coventry and Warwickshire, Clifford Bridge Road, Coventry CV2 2DX, United Kingdom; 3 Department of Chemistry, University of Warwick, Coventry, CV4 7AL, United Kingdom; 4 Fellowship of Postgraduate Medicine, 11 Chandos St, London W1G 9EB, United Kingdom; 5 Division of Metabolic and Vascular Health, Clinical Sciences Research Laboratories, Warwick Medical School, University of Warwick, Coventry, CV2 2DX, United Kingdom; Albert Einstein College of Medicine, UNITED STATES

## Abstract

Finding pleiomorphic targets for drugs allows new indications or warnings for treatment to be identified. As test of concept, we applied a new chemical genomics approach to uncover additional targets for the widely prescribed lipid-lowering pro-drug simvastatin. We used mRNA extracted from internal mammary artery from patients undergoing coronary artery surgery to prepare a viral cardiovascular protein library, using T7 bacteriophage. We then studied interactions of clones of the bacteriophage, each expressing a different cardiovascular polypeptide, with surface-bound simvastatin in 96-well plates. To maximise likelihood of identifying meaningful interactions between simvastatin and vascular peptides, we used a validated photo-immobilisation method to apply a series of different chemical linkers to bind simvastatin so as to present multiple orientations of its constituent components to potential targets. Three rounds of biopanning identified consistent interaction with the clone expressing part of the gene *GJC3*, which maps to *Homo sapiens* chromosome 7, and codes for gap junction gamma-3 protein, also known as connexin 30.2/31.3 (mouse connexin Cx29). Further analysis indicated the binding site to be for the *N*-terminal domain putatively ‘regulating’ connexin hemichannel and gap junction pores. Using immunohistochemistry we found connexin 30.2/31.3 to be present in samples of artery similar to those used to prepare the bacteriophage library. Surface plasmon resonance revealed that a 25 amino acid synthetic peptide representing the discovered N-terminus did not interact with simvastatin lactone, but did bind to the hydrolysed HMG CoA inhibitor, simvastatin acid. This interaction was also seen for fluvastatin. The gap junction blockers carbenoxolone and flufenamic acid also interacted with the same peptide providing insight into potential site of binding. These findings raise key questions about the functional significance of *GJC3* transcripts in the vasculature and other tissues, and this connexin’s role in therapeutic and adverse effects of statins in a range of disease states.

## Introduction

**Fig 1:**
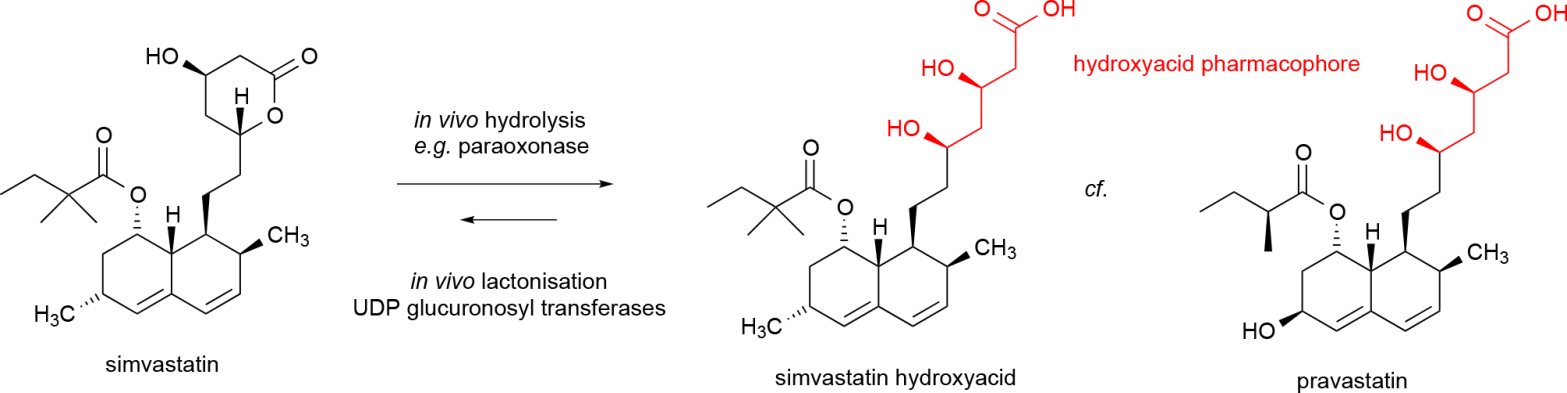
Simvastatin pro-drug compared with its hydrolysis product and pravastatin.

Treatment with statins has had a major impact on cardiovascular disease and mortality. The primary mechanism by which statins reduce ischaemic cardiovascular disease is accepted as a reduction in circulating cholesterol achieved both by inhibiting HMG CoA reductase [1], and increased cholesterol scavenging by upregulation of LDL receptors [2]. However, statins have important pleiotropic actions [3,4], independent of their lipid-lowering properties. These pleiotropic actions may contribute both to the cardiovascular benefits of statins and to their adverse effects. Simvastatin is a lactone prodrug, hydrolysed by non-specific carboxyesterases or non-enzymatic processes to its active hydroxyacid (Fig 1); lactonisation of the hydroxyacid form of all statins can occur *in vivo* by the action of UDP-glucuronosyl transferases [5,6]. Therapeutic or adverse effects of simvastatin and other statins may occur due to actions of these lactones or due to active hydroxyacid statins and their metabolites. For example β2 integrin leukocyte function antigen-1, has been identified as a significant, beneficial off-target effect of the lactone form of several statins [7], although the lactone form has also been implicated in statin-associated myotoxicity [8]. New approaches to understanding pleiotropic effects of drugs and their metabolites on molecular, cellular and other systemic networks are important both for early stage drug discovery and safety pharmacology [9,10].

We have developed a novel approach to understanding the impact of drugs on such a network pharmacology approach. This combines chemical genomics phage display [11,12] with rapid immobilisation of a bioactive molecule in multiple orientations (Magic Tag^®^) [13] to explore beneficial and adverse targets and actions of therapeutic drugs [14], metabolites and other ligands [15]. We here apply a modification of this approach to identifying new molecular targets for the pharmacological effects of simvastatin, one of which is of particular interest and is confirmed as present in the tissue under investigation.

**Fig 2:**
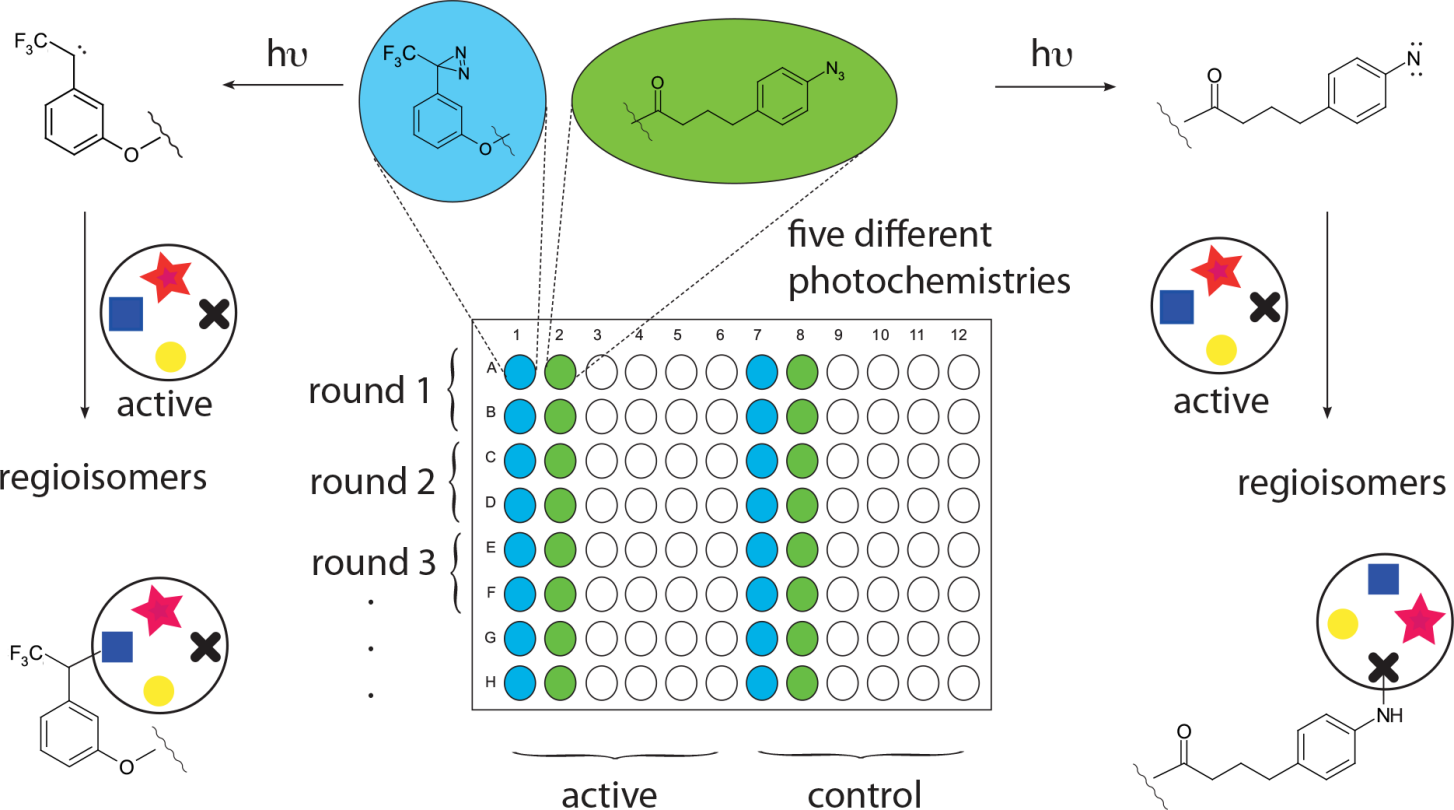
Immobilisation of a small molecule active using multiple photochemistries. The use of five different photochemistries in a multiwall format allows the simultaneous, rapid exploration of several regio- and other isomeric derivatives.

Classic biochemical approaches to identifying cellular and molecular targets [16] often rely on displaying a ligand on a support which is screened against cell lysate, which has several drawbacks. Immobilising the ligand in multiple orientations [13,14,17–22]

offers improved exploration of chemical space (Fig 2) and the use of genomic polypeptide display libraries [23] offers the chance to better explore the breadth of biological sequence space [24,25]. The chemistry, morphology of the support [26] and screening conditions can also be tuned to improve selection of meaningful interactors [27] and reduce non-specific binding [28]. Rather than selecting bound phage by elution with the small molecule active, which can result in inhibition of phage replication, we innovate herein the direct use of the host bacterium, *Escherichia coli*. BLT5615 in a concomitant elution and amplification step.

We provide biophysical evidence, using surface plasmon resonance measurements [29], that the discovered N-terminal sequence codes for a peptide that interacts in a meaningful way with statins. In addition, a selection of known, structurally diverse and commercially available gap junction inhibitors [30,31] were investigated providing new structure-function insight for the connexin proteins.

## Results

### Preparing the viral library

**Fig 3:**
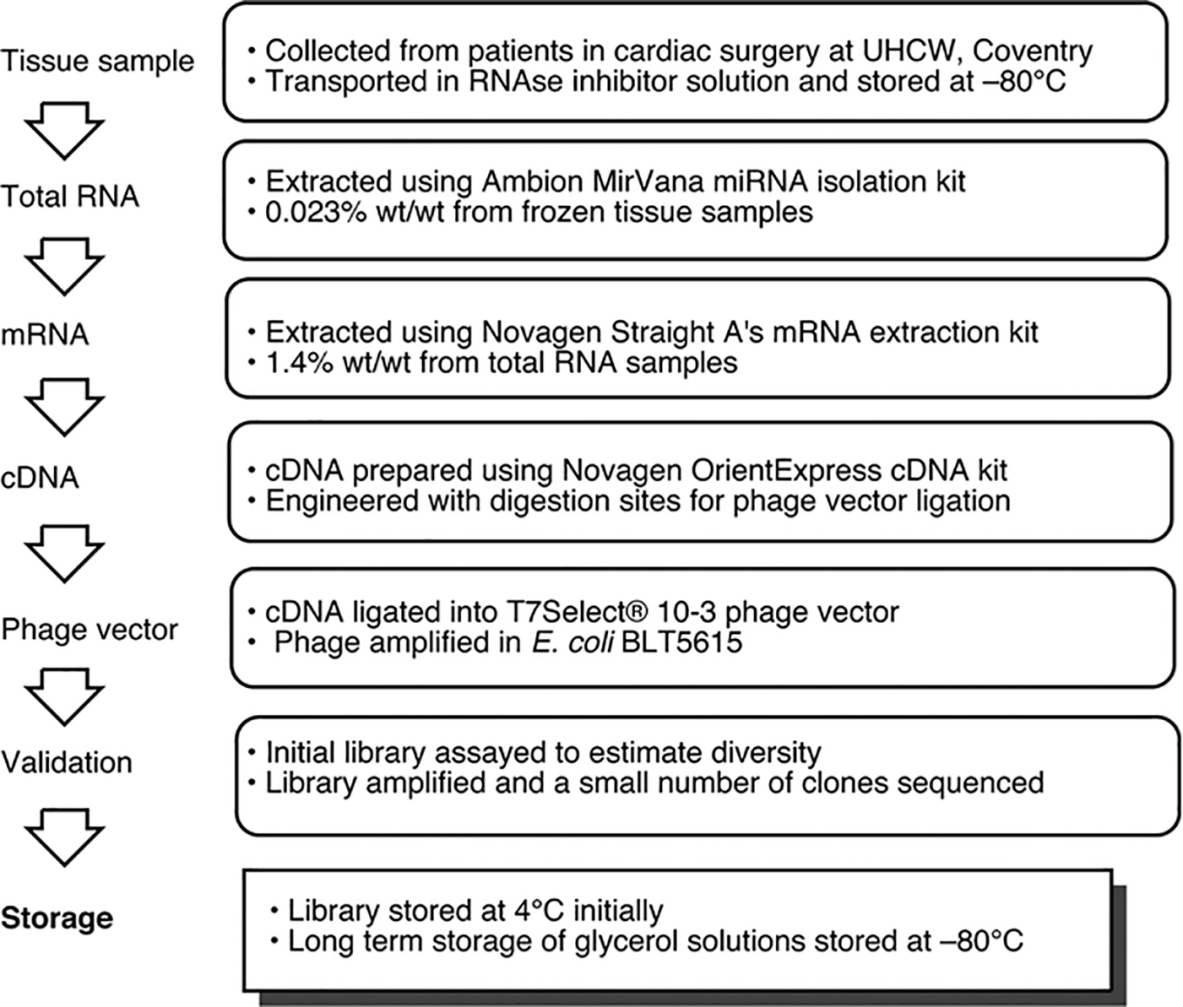
Preparation of a T7 phage display library from vascular tissue.

Standard procedures to extract and purify mRNA from total RNA obtained from internal mammary artery samples (Fig 3) were used see S1 Protocol and K. Casey-Green Ph. D. Thesis, University of Warwick [32] for full detail. A cDNA library was then created using an oligo(dT) priming approach which was ligated into phage vector arms using the mid-copy number display vector T7Select^®^ 10–3, leading to 5–15 copies of the insert on the head group of the phage [33] at a molar ratio of 2:1. This was in order to minimise the number of vectors which self-ligate, leading to 'empty' vectors [34,35].

**Fig 4:**
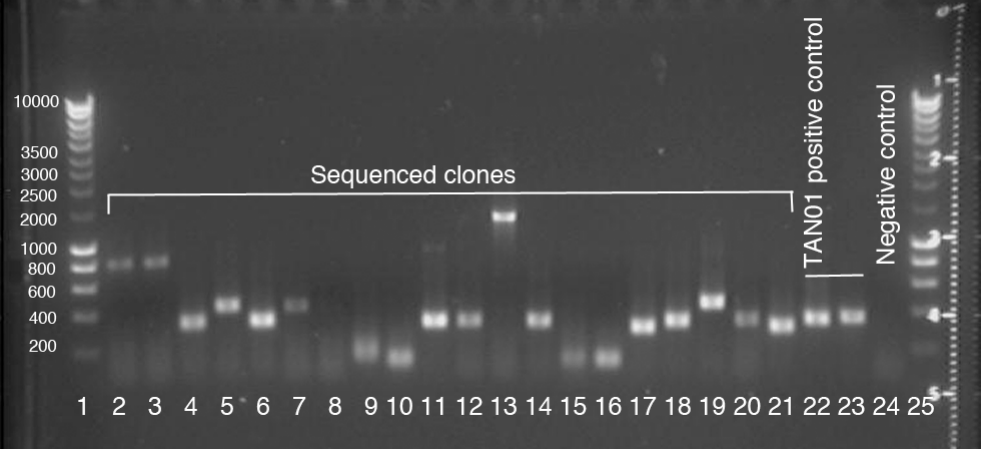
Ethidium bromide stained agarose gel showing a sample of clones from the phage display library after PCR to amplify the insert DNA. Hyperladder in lanes 1 and 25 allows estimation of insert length. PCR controls in lanes 22, 23 and 24 (positive, positive and negative).

The diversity of the library (number of unique clones) was determined prior to amplification by selecting two samples, for which titres were assessed by a plaque counting assay on *E*. *coli* lawns, which were seen to be entirely lysed after 3 hours. This suggested a population of greater than 5,000 plaques for 100 μl at a dilution of 10^−2^, inferring a diversity of greater than 1.5 x 10^5^ unique clones. The remaining library was amplified in two halves (to avoid possible disproportionation, detrimental to library diversity) in *E*. *coli* culture, strain BLT5615, at its exponential replication stage (*i*.*e*. ‘log phase’). Sixteen plaques were picked from the surface of agar titre plates and their genetic material amplified by touchdown PCR with agarose gel electrophoresis confirming inserts of length between 200–1500 base pairs (Fig 4).

### Screening against simvastatin pro-drug

**Fig 5:**
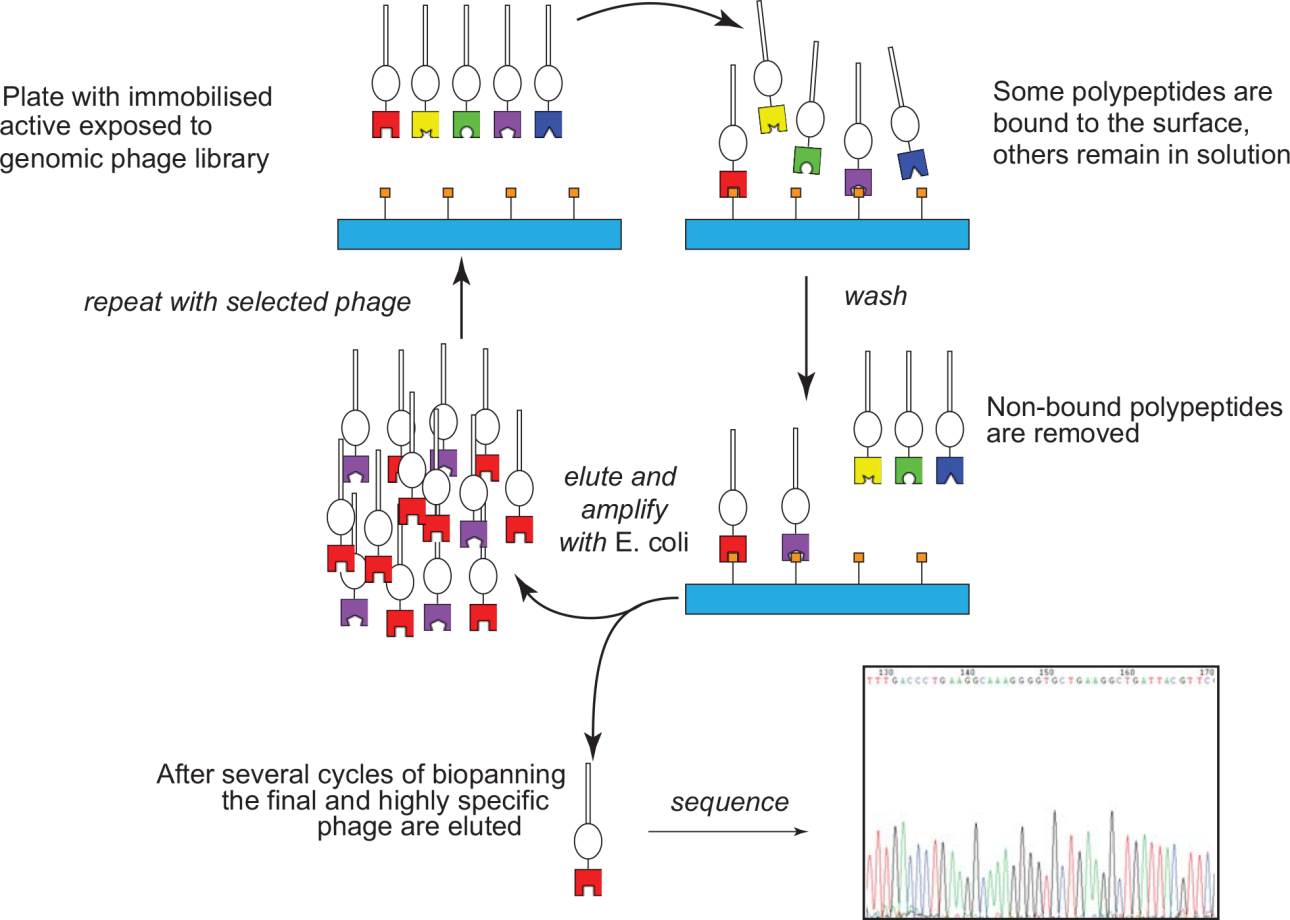
Biopanning against the genomic phage library displaying 5–15 polypeptide copies per headgroup. Elution is carried out herein using the host bacterium, *E*. *coli* BLT5615 avoiding potential inhibitory or other effects of an active small molecule.

Screening of the phage library was carried out using simvastatin immobilized on 5 different photosensitive moieties following published procedures (Fig 5) [13,14]. In brief, by using these different chemical linkers in separate wells on a test plate, we sought to display simvastatin in many orientations to potential binding polypeptides of interest. Following a usual wash procedure to remove non-specifically bound phage, phage selected against the immobilized simvastatin were eluted and amplified by directly adding host *E*. *coli* BLT5615 to the plate. After three rounds of binding, elution and amplification, phage clones binding to simvastatin in given wells were detected from areas of lysis in an *E*. *coli* agarose lawn grown over fresh agar plates.

Following this biopanning process, phages selected from the final *E*. *coli* agarose lawn were submitted to Sanger sequencing (S1 Dataset) to give 120 sequences (S1 Table) which were aligned using the MegaBLAST tool against the coding region for T7Select 10–3 vector (18 clones vector only, S2 Table) from which coding regions for the T7Select^®^ 10–3 vector were removed. Highly similar sections of clone sequence were noted and all low quality ends trimmed, resulting in 31 contiguous sequences (S3 Table). Simvastatin-associated phage clones bearing polypeptide sequences were corrected for the 60 non-specific binding clones in control wells (S2 Dataset) leaving 23 consensus sequences from these contiguous regions, or ‘contigs’. These were first searched against the NCBI Genome (all assemblies scaffolds) using BLASTN optimized for highly similar sequences [36] and the human genome results further assessed by searching against the human RefSeq database [37] using the BLASTX algorithm (S4 Table).

This led to identification of 11 sequences (‘contigs’) sufficiently annotated with biological function in the literature to be considered of significance to human health (S1 Summary) [32]. We note that one of these contigs has excellent homology with myosin light chain which has previously been identified as a strong interactor in a T7 phage screen [38]. In this communication, we focus on the sequence of our contig29, which we recognised to have an identical amino acid sequence to the N-terminal residues 1–25 of the human gap junction gamma 3 protein GJC3 [39,40][41], (human connexin 30.2/31.3, hCx30.2/31.3, NP_853516, UniProt Q8NFK1.1) synonymous with mouse connexin mCx29 [39,42–46].

### Structure of discovered sequence

**Fig 6:**
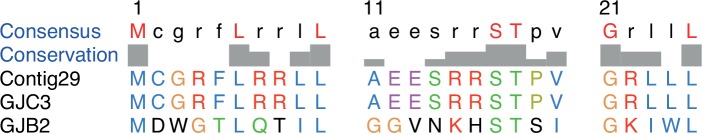
Alignment of discovered contig29 versus GJC3 (residues 1–25) and the sequence for GJB2 (residues 1–25). Carried out using T-Coffee (www.ebi.ac.uk) with GJC3 NCBI sequence NP_853516.1 and the protein databank file corresponding to GJB2 code 2zw3.pdb. Consensus residues are shown in the upper row; figure prepared using UCSF *Chimera* [50].

A molecular structure has not been solved for GJC3 (hCx30.2) or its C-terminal splice variant hCx31.3, but the X-ray crystal structure of human connexin 26 (hCx26) gap junction GJB2 (2zw3.pdb) [47] allowed us to perform simple homology modelling [48]. Alignment of our contig29 sequence, and NP_853516.1 against that for GJB2 (Fig 6) highlights the sequence identity between the first two, but difference between the latter two, as might be expected. S1 Fig shows the full sequence alignment for GJC3 and GJB2 from S1 Alignment performed using T-Coffee [49] (www.ebi.ac.uk) and rendered using UCSF Chimera.[50]

**Fig 7:**
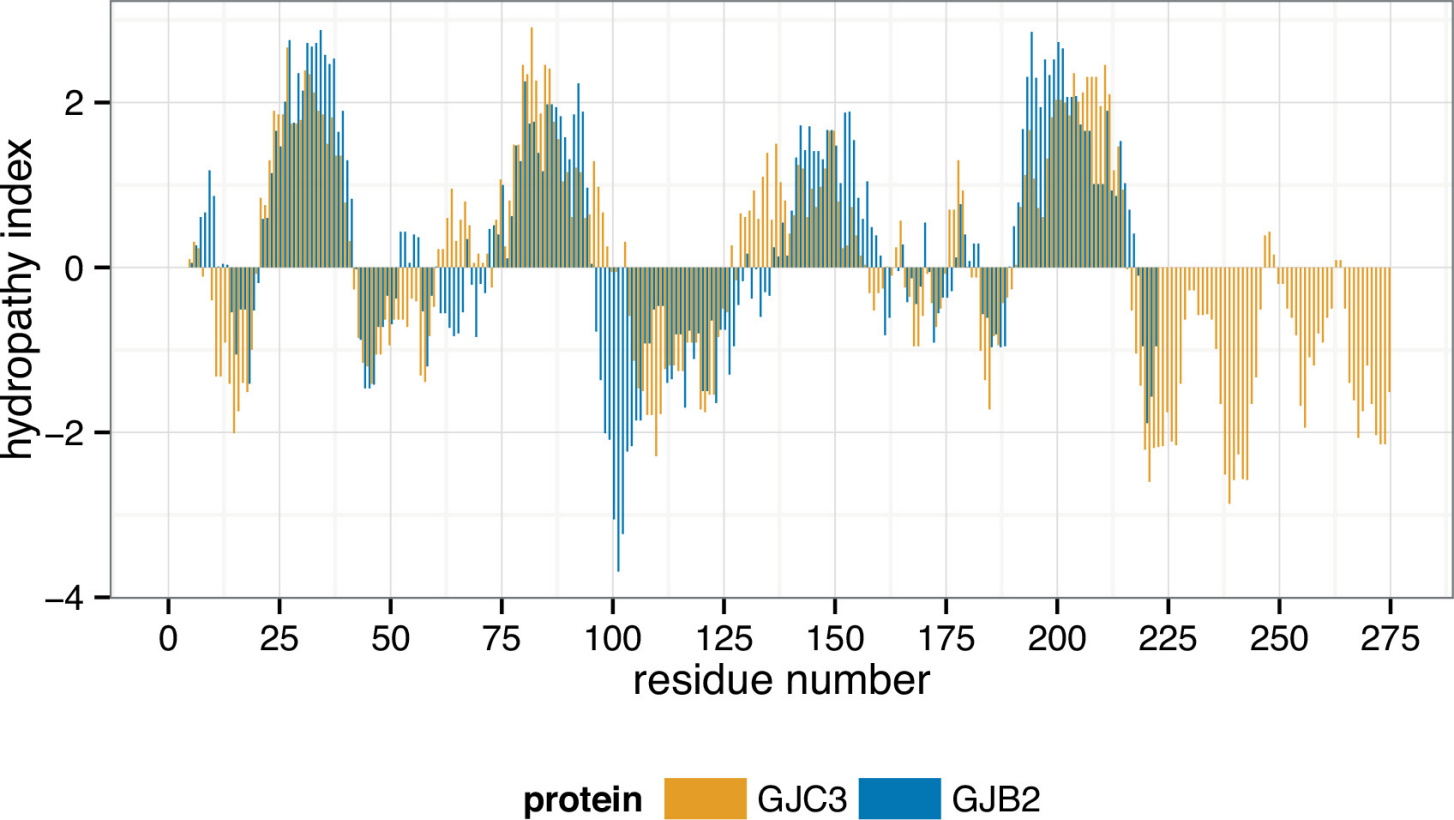
Comparison of Kyte-Doolittle index hydropathy plot of GJC3 (orange) and GJB2 (blue) showing predicted transmembrane regions as positive values. Kyte-Doolittle analysis using ProtScale [124] http://web.expasy.org/protscale/. Parameters used were: window size = 9, relative weight 100%, linear model, not normalized. Data replotted using *ggplot2* in *R* [125].

**Fig 8:**
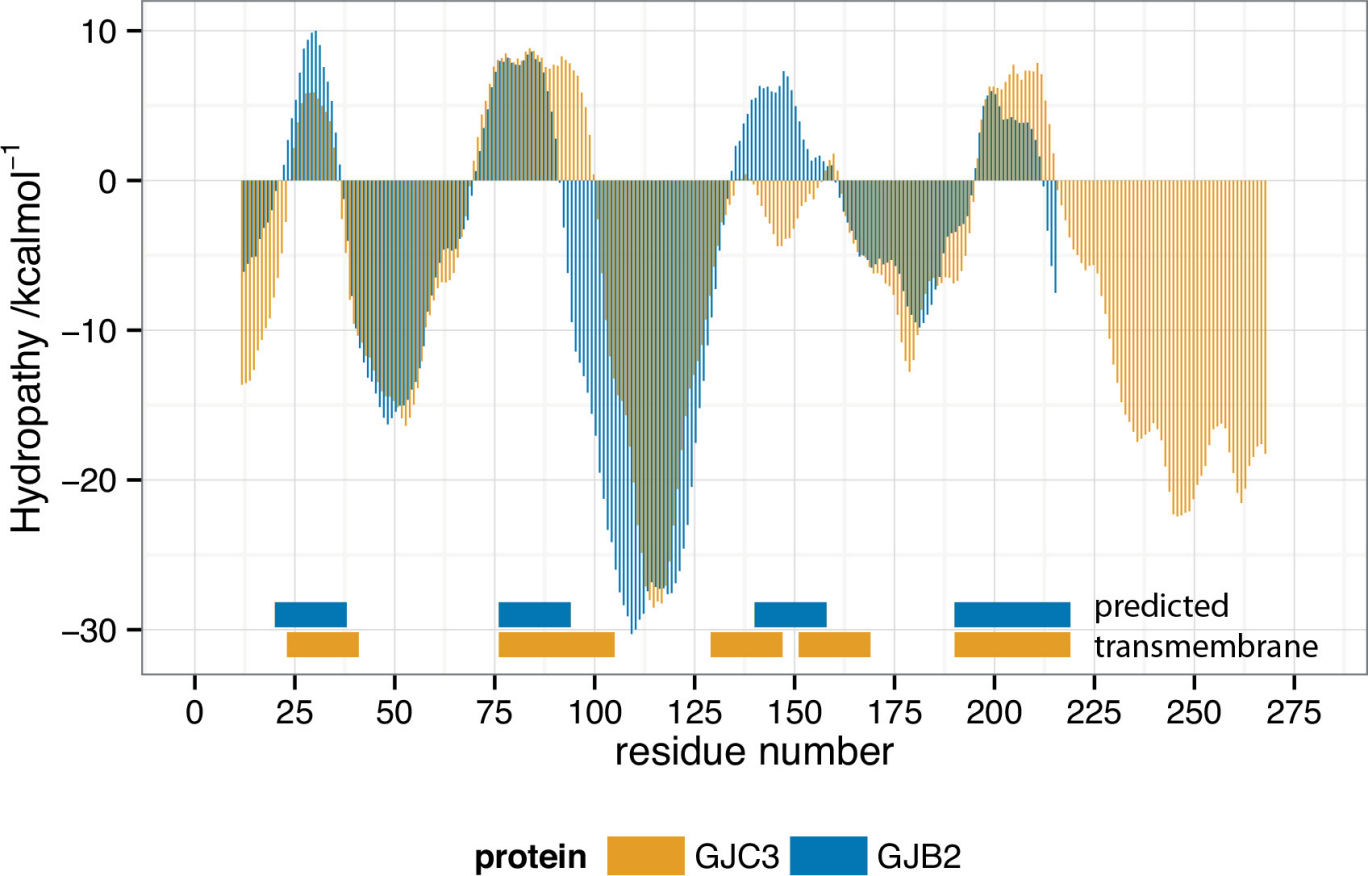
Wimley-White hydropathy plot of GJC3 (orange) and GJB2 (blue) showing predicted transmembrane regions as positive negative values. Wimley-White octanol-to-water scale smoothed values for GJC3 and GJB2 are shown, where positive hydropathy values indicate expected membrane associated residues. The predicted transmembrane (TM) segments indicated are at least 19 residues in length and were calculated using a validated algorithm [126]. Wimley-White smoothed hydropathy calculated using MPEx [127] http://blanco.biomol.uci.edu/mpex/ and replotted using *ggplot2* in *R* [125].

**Fig 9:**
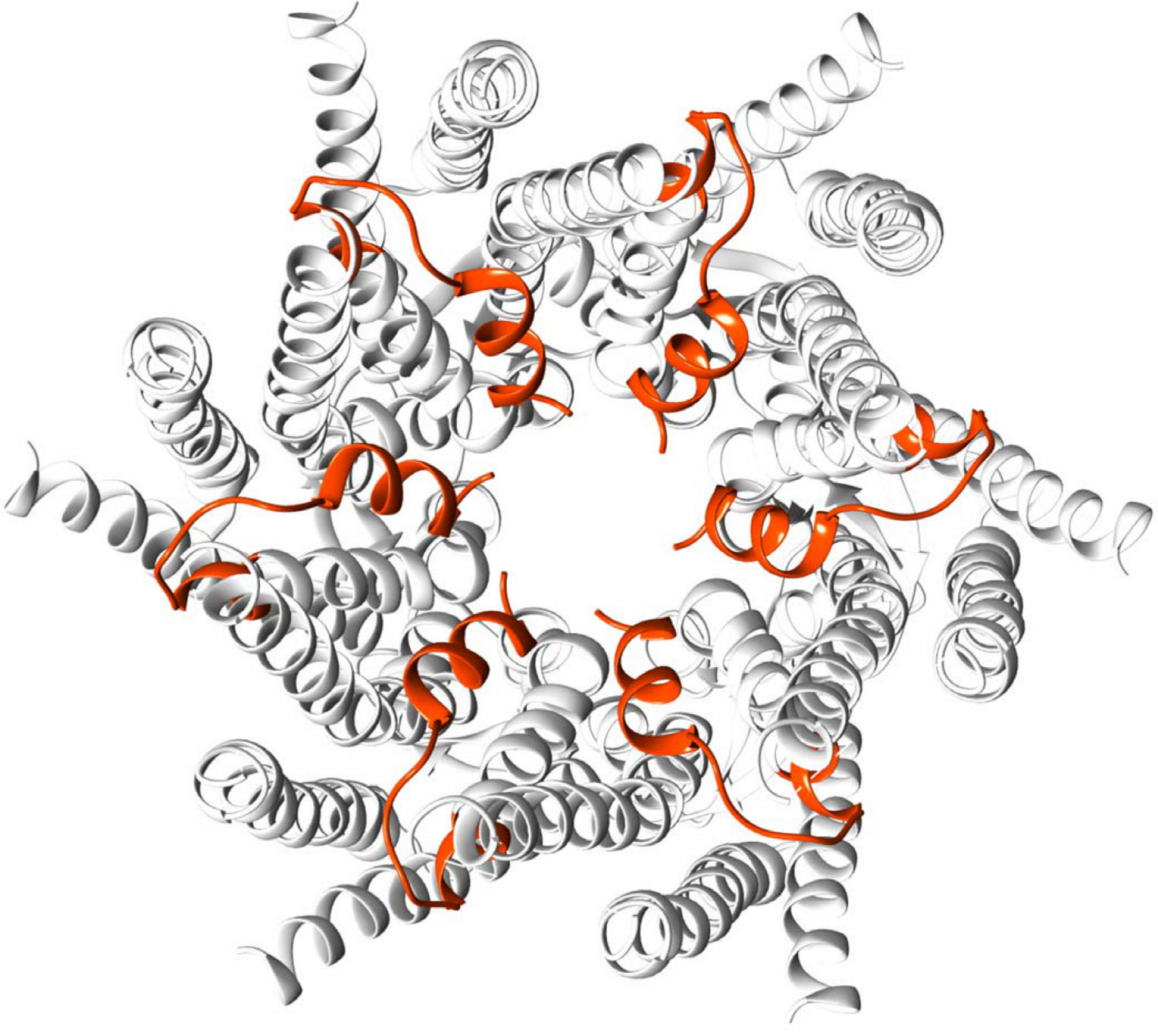
Discovered *N*-terminal residues 1–25 (orange) aligned on a simple model based on GJB2.pdb X-ray structure. Hexameric structure viewed from cytoplasmic side of the transmembrane hemichannel (prepared using UCSF *Chimera* [50]).

Hydropathy plots (Figs 7 and 8) were nonetheless well matched for the two full-length proteins, indicating that a similar transmembrane structure is retained. Both the Kyte-Doolittle and Wimley-White scales show good agreement between predicted transmembrane regions for GJC3 and GJB2. The discovered sequence, corresponding to residues 1–25 of GJC3 is highlighted in orange on the secondary structure for the known coordinates of GJB2 (2zw3.pdb) in Fig 9. Its position corresponds to the non-transmembrane portion residing in the cytoplasmic channel opening expected to be formed by homology [48,51] with a number of gap junction proteins in hemichannels and connexons. We continue to develop a more thorough homology model of GJC3 in a phospholipid bilayer and to explore its structure and function in detail.

### Immunohistochemistry

**Fig 10:**
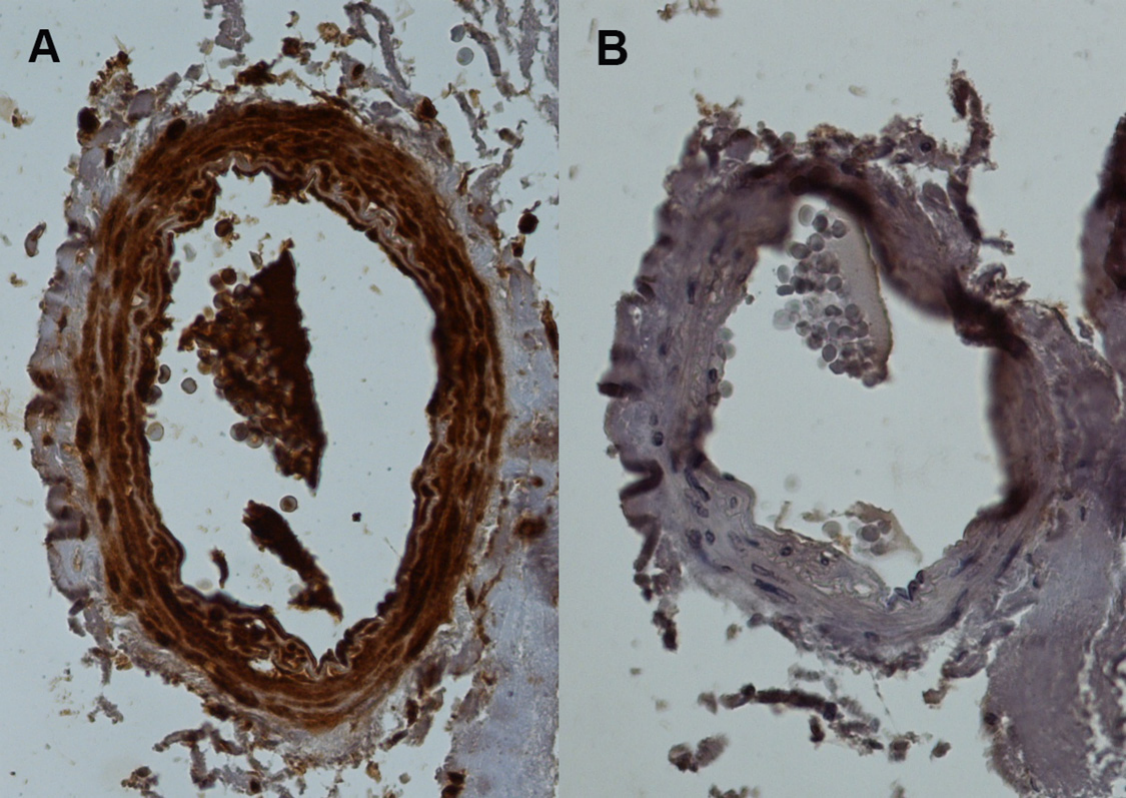
Immunohistochemistry for human artery with antibody against GJC3, hCx30.2/31.3. (A) H-86—sc-68376, Santa Cruz Biotechnology, Inc., vs. (B) control section, both micrographs at 20 x magnification. Brown staining in (A) indicates selective localisation of GJC3 in media and endothelium of the artery.

There was selective GJC3 expression in vascular tissue from patients with ischaemic cardiovascular disease (Fig 10). Expression was localized to diffuse staining in media and intima but not in adventitia (Fig 10). Immunohistochemistry also showed expression of GJC3 in capillary endothelium [data not shown].

### Additional Bioinformatics

**Fig 11:**
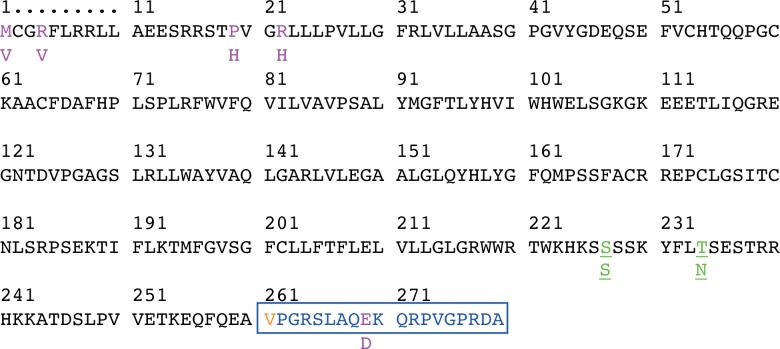
Alternative mRNA splicing and selected single nucleotide polymorphisms for *GJC3* products. CCDS entry CCDS34697.1 (accessed 24 May 2012) for transcribed mRNA from *GJC3* (NM_181538.2 and NP_853516.1) and its translated 279 amino acid polypeptide showing the alternate exon region, designated with a blue rectangle. Residues highlighted in purple (M1V, R4V, P19H, R22H, E268D) indicate single nucleotide polymorphisms (SNPs: see main text) leading to variation in coded amino acids within predicted geometrically neighbouring and functionally significant N- and C-terminal regions. V261 highlighted in orange indicates the C-terminal splice variant start point. Discovered residues correspond to amino acids 1–25. Underlined residues S227S, T234N highlighted in green indicate phosphorylation sites identified from PhosphositePlus and genomic SNPs.

A search of the Human Protein Atlas [52] for GJC3 reveals wide tissue (S5 Table) and cellular (S3 Dataset) expression of this protein. Data on vascular tissue is absent, although the presence of GJC3 at medium levels of expression is noted in heart muscle. S4 Dataset summarises tissue expression for the wider set of connexins from the same database. The ICR CanSAR resources [53] and EBI Catalogue of Somatic Mutations in Cancer (COSMIC) [54], accessed Dec 2015, reveal over-expression and copy number variant gain (42/278 samples) in a variety of cancer cell lines and tumour samples [623/28982 tested with 46 unique samples with mutations, e.g. adrenal gland (10/79 tested), breast (68/1092 tested), oesophagus (24/125 tested) stomach (31/285 tested) pancreas (17/168 tested)]. Single nucleotide polymorphism (SNP) missense mutations for *GJC3* present in the 1000 Genomes Project [55] from NCBI search 19 Dec 2015 are listed in S5 Dataset and insertion/deletion mutations in S6 Dataset. A search of PhosphositePlus [56] for human GJC3 shows sites for post-translational phosphorylation found by proteomic mass spectrometry to be confined to the C-terminal portion: S227 and T234 (Fig 11). In the orthologous mouse *Gjc3* gene product mCx29 (Uniprot Q921C1; see S7 Dataset and NCBI HomoloGene 15399 used to generate a Multiple Sequence Alignment such as S2 Fig), different phosphorylation sites have been identified: mCx29_S247, mCx29_S249, mCx29_S250, mCx29_S253, mCx29_S261, together with sites of ubiquitinylation mCx29_K254, mCx29_K266 and a site of *N*-acetylation mCx29_K141.

### Surface Plasmon Resonance (SPR)

**Fig 12:**
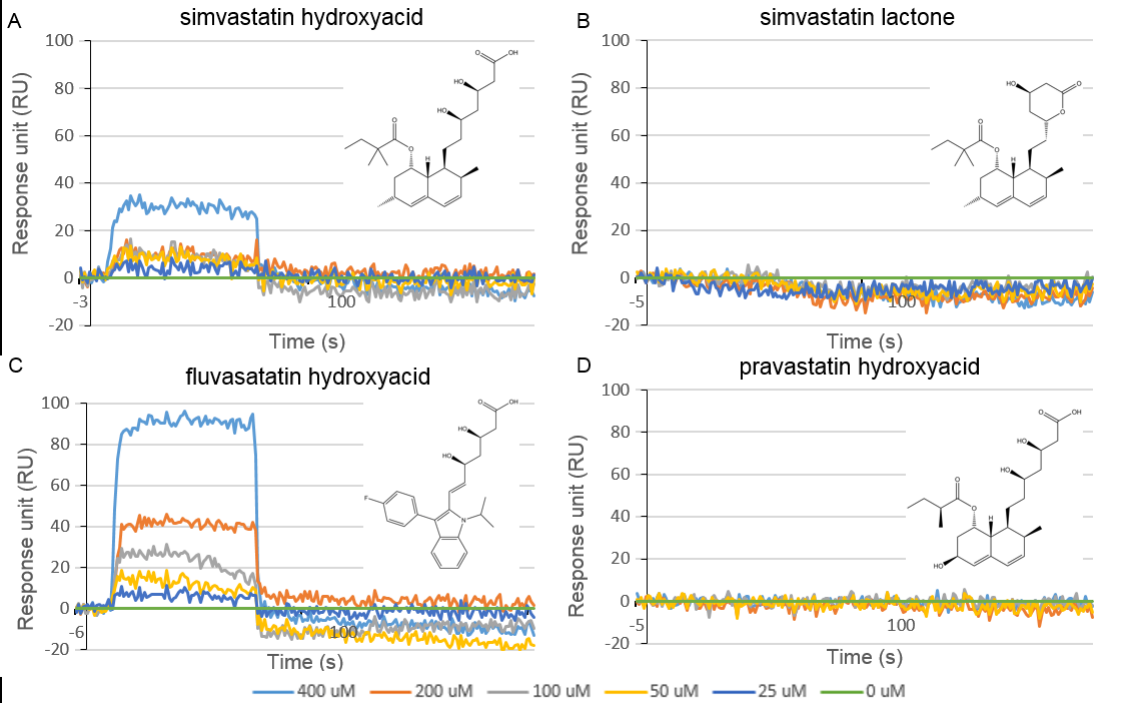
Surface plasmon resonance sensorgrams of peptide A interacting with statins. (A) Simvastatin hydroxyacid, (B) simvastatin lactone, (C) fluvastatin hydroxyacid, and (D) pravastatin hydroxycid, each at concentrations of 400 μM (light blue), 200 μM (red), 100 μM (grey), 50 μM (yellow), 25 μM (dark blue) 0 μM (green) for each ligand in PBS. Concentration dependent interactions are observed for both simvastatin and fluvastatin hydroxyacid sodium salts with the most pronounced interaction observed for fluvastatin.

Each chemically synthesized peptide corresponding to the expected reading frame for contig29 (peptide A), frame +1 nucleotide (peptide B), frame +2 nucleotides (peptide C) (S3 Fig) was successfully immobilised in separate lanes of a 6-channel ProteOn NLC sensor chip (S4 Fig) and then exposed to increasing concentrations of a range of statins (simvastatin, fluvastatin and pravastatin hydroxyacids, as well as simvastatin lactone). Fig 12 shows the SPR chromatograms of the statins interacting with the in-frame peptide A while the corresponding chromatograms for the out-of-frame peptides B and C are shown in S5 Fig.

Simvastatin (*i*.*e*. the lactone) showed no interaction with the immobilised peptides, whereas the sodium salt of its hydroxyacid demonstrated a weak concentration dependent profile with the in-frame peptide A only (Fig 12A). When each peptide was exposed to fluvastatin sodium salt, more pronounced binding is seen to peptide A (Fig 12C), with little interaction seen with peptide B and no interaction with peptide C (S5 Fig, Panel C). Interestingly, pravastatin sodium salt, which is more hydrophilic, showed no interaction with any of the immobilised peptides (Fig 12D and S5 Fig, Panel D) suggesting the hydroxyacid pharmacophore alone is not sufficient for binding of statins to peptide A.

**Fig 13:**
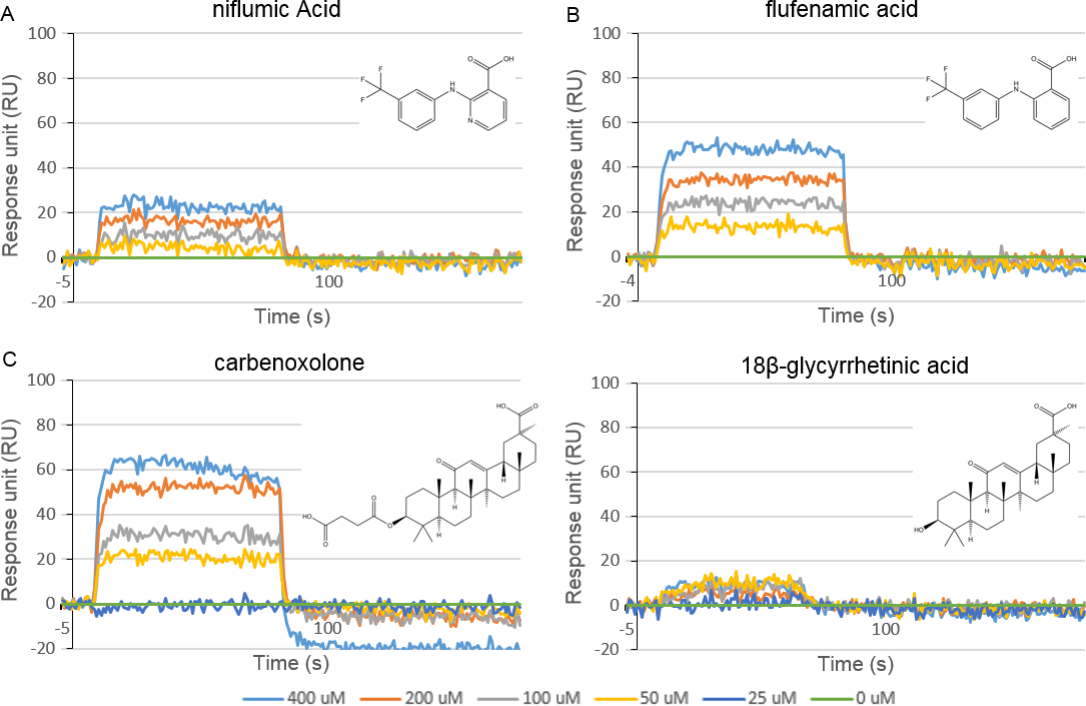
Surface plasmon resonance sensorgrams of peptide A interacting with known hemichannel blockers. (A) Niflumic acid, (B) flufenamic acid, (C) carbenoxolone, and (D) 18β-glycyrrhetinic acid each at concentrations of 400 μM (light blue), 200 μM (red), 100 μM (grey), 50 μM (yellow), 25 μM (dark blue) 0 μM (green) for each ligand in PBS. Concentration dependent binding profiles are observed for niflumic acid with more pronounced effects observed for flufenamic acid and carbenoxolone.

In order to further investigate the binding characteristics of peptide A, a number of known gap junction inhibitors [30,57] (for a list of inhibitors abstracted from these references and their known and shared targets from ChEMBL_20, [58] see S8 Dataset) were chosen for SPR analysis. Fig 13 illustrates increasing concentrations of niflumic acid (A), flufenamic acid (B), carbenoxelone (C), and its parent 18β-glycyrrhetinic acid (D) binding to peptide A while the corresponding chromatograms of peptides B and C are shown in S6 Fig. As expected, no interaction was observed for any of the compounds tested with the out-of-frame peptides B and C. A concentration dependent binding profile of niflumic acid was observed with peptide A and a stronger interaction observed for flufenamic acid. The glycyrrhetinic acid derivative, carbenoxolone, exhibited a pronounced binding interaction, while there was no evidence for the parent 18β-glycyrrhetinic acid or 18α-glycyrrhetinic acid binding to peptide A. Additional compounds which were investigated using SPR included 3-hydroxymethylglutaric acid, arachidonylethanolamide, heptanol, steviol, stevioside and 18α-glycyrrhetinic acid. None exhibited any binding to the peptides (S7 Fig). Data for SPR experiments is included in S9 Dataset.

## Discussion

We report here a previously unrecognized molecular interaction between simvastatin in its HMG CoA reductase inhibitory hydroxyacid form and the N-terminal domain of gap junction gamma-3 protein. The position of the N-terminal domain in what is expected to be the cytoplasmic channel opening formed by the gap junction proteins indicates that this putative interaction site for simvastatin hydroxyacid may influence function as a component either of hemichannels or cell-cell gap junctions. Interactions of additional small molecules explored in this work including niflumic acid, flufenamic acid and carbenoxolone with the N-terminus may explain the known inhibitory effect on other connexins of these compounds. Diffuse staining of immunohistochemically labelled samples herein is consistent with expression of GJC3 in human arteries. Tissue-specific data [59] from The Human Protein Atlas [60] suggest much wider expression of GJC3 than previously recognized, although the vasculature does not appear as a separate entry in that database [61].

### Connexins in cardiovascular tissue

The connexins are a large structurally-related family of membrane proteins capable of building gap junction channels, which permit direct exchange of small molecules (≤ 1200 Da) and electrical current among neighbouring cells; and hemichannels, capable of allowing cells to communicate between the intra-cellular and extra-cellular space [62]. Both amino and carboxy termini of connexins are found in the cytoplasm [40]. A large number of genes are involved in direct cell-cell communication in cardiovascular and other tissues [63]. Genetic and acquired variability in connexin structure and function may contribute to disease risk, natural history and variable response to treatment including adverse events [64,65]; and provide biomarkers of disease for diagnosis, prognosis and prediction of and monitoring of treatment response. Connexins have in particular been suggested to play a role in the pathogenesis of atherosclerosis [66]. Each of the three key cell types involved in atherogenesis, monocytes/macrophages, endothelial cells, and smooth muscle cells, express a distinct pattern of connexins, with recognized vascular connexins, Cx37, Cx40, and Cx43, differentially modified as atherosclerosis develops [67,68]. Of particular interest to this current work is the observation that lovastatin has been found to inhibit gap junction intercellular communication in cultured aortic smooth muscle cells [69].

### Physiological significance of human gap junction gamma-3 protein

Human connexin 30.2 [61] (NP_853516, hCx30.2) is an integral membrane protein expressed from *GJC3* (NCBI Gene ID: 349149) and is known by its official name GJC3, “human gap junction gamma-3 protein, 30.2 kDa”, which also has a splice isoform of molecular mass 31.3 kDa, hence it is also called hCx30.2/31.3. It is now recognised as a member of the γ-subfamily of connexins [70], although “connexin 31.3” [71], has previously been referred to as gap junction ε-1 protein GJE1 [71,72] (‘gje1’ is the approved symbol for an orthologous protein in frogs [70,73] and *GJE1* in primates is now considered a pseudogene for Cx23 whose expression is prevented by a nonsense mutation [74]) and the ortholog mouse connexin 29 (mCx29) [42,43,45,46] has been used synonymously. Hence in this publication we use names for all three gene products “hCx30.2/31.3 (mCx29)” where possible, noting for clarity that mouse connexin 30.2 (mCx30.2) is not the same as our protein, but is orthologous with human connexin hCx31.9 [75].

The human genetic locus for *GJC3* maps to 7q22.1 (CCDS 34697.1 GRCh37.p5) [71,72] and has been reported to group with [76,77] *Gjc3* in *Mus musculus*, *gjc3* in *Canis familiaris* [78] and other mammals (Homologene 15399 alignment, S7 Dataset and S7 Fig). As with other mouse/human connexin orthologs, expression profiles across the species differ. Our study is the first we are aware of to suggest that this connexin plays a vascular role, although hCx30.2/31.3 (mCx29) is reported in humans to be expressed strongly in heart, liver and skeletal muscle (1.9 kb transcript), with a 1.6 kb transcript in pancreas [61]. Immunohistochemistry data recently made available through the Human Protein Atlas [60] shows much wider expression of GJC3 than previously recognized, although that antibody data is marked as “uncertain reliability”. In that work, strong expression (assayed through polyclonal antibody HPA015024 raised against a hapten representing GJC3 amino acids 220–279) was observed in many tissues, although only low expression was detected in skeletal muscle, or the liver (S5 Table, and expression of GJC3 in primary cell types S3 Dataset comparing the wider set of gap junction proteins’ tissue expression in S4 Dataset). Mouse Cx29 expression by contrast is considered to be restricted to the central and peripheral nervous system, including brain, spinal cord and sciatic nerve [42], where it is expressed in Schwann cells in small myelinating fibres and is postulated to exist as hemichannels [43,79,80]. In humans, hCx30.2/Cx31.3 R15G and L23H mutants expressed in the inner ear have, along with several other connexins [81], been implicated in non-syndromic hearing impairment [82,83]. Its over-expression in a variety of cancer tumour and cell line samples together with copy number variant gain seen in data from COSMIC [54], confirm unexplored tissue profiles and linkage with disease. Hence the observed function of GJC3 in mediating ATP release in conjunction with acknowledged experimental difficulty in differentiating connexin from pannexin 1 purinergic signalling [84,85] (which has been positively linked with P2 receptors and sympathetic vasoconstriction [86]), make this and its related channels of particular interest [87].

N-Terminal sequences are recognized to be crucial for function in many connexins [88,89] and there are 4 single nucleotide polymorphisms (SNPs) observed in this discovered region of GJC3 (Fig 11: M1V (C/T rs192406037), F5V (A/T rs187126645), P19H (G/T rs201016616), and R22H (C/T rs200074250)) and three somatic mutations p.R8Q (COSM3267470, c.23G/A; carcinoma, upper aerodigestive tract), p.L25I (COSM1093913, c.73C/I; carcinoma, endometrium) and p.V27M (COSM5184309, c.79G/A; carcinoma, large intestine). Other SNPs are seen in the putative structurally neighbouring cytoplasmic C-terminus with at least 35 documented in the RefSeq genome NM_181538.2 associated with *GJC3* gene products, not including two in the 5’-untranslated region, one of which is associated clinically with peripheral neuropathy induced by bortezomib [90,91]. The C-terminus of hCx30.2/31.3 also contains two sites for post-translational phosphorylation, S227 and T234 (Fig 11), identified by proteomic mass spectrometry, according to PhosphositePlus. Those sites of phosphorylation, the only ones reported in GJC3, are subject to genomic variation (Fig 11: the synonymous variant S227S [rs769486892 C/T] and T234N [rs140894833 C/A] and in the C-terminus from amino acids 219–279 there are 14 known somatic mutations including the neighbouring R220S (COSM219466, c.660G/T; breast carcinoma) and S228T (COSM4149446, c.682.T/A; carcinoma, ovary). They introduce potentially complementary phosphate groups to the N-terminal arginine functions seen at R4, R7, R8, R15, R16, offering a testable hypothesis by which the gating of the channel might be controlled (see ‘Functional significance of GJC3 N-terminus‘ below). Importantly the clinically observed R15G mutant has already been shown to lead to decreased ATP release, but not affect trafficking to the membrane [83].

We note that an alternative translation initiation site in the mouse gene *Gjc3* has been shown to contain a 450 bp exon, overlapped with the main exon when spliced, which produces an 11 amino acid residue extended N-terminal sequence in mCx29 [46] containing what may be, for that species, a potentially important glutamic acid and a lysine residue (see multiple sequence alignments, S7 Dataset and S2 Fig).

The mRNA splice variant of *GJC3*, hCx31.3 is known to be expressed in human oligodendrocytes [41] and in line with earlier observations [43] this protein, as with mCx29 [92] has been found not to form full connexon or gap junction channels, but “shares functional properties with pannexin (hemi) channels” [93]. Nonetheless, connexins such as these are both disease-mediating and drug-modifiable targets of cardiovascular relevance [64] and their observation in internal mammary artery, and from The Human Protein Atlas, many other tissues with the exception of liver and skeletal muscle, is thus significant.

### Statins and human gap junction gamma-3 protein

Statins have previously been linked indirectly to other connexins, for example exhibiting potential vascular anti-proliferative effects by reducing Cx43 expression in primary human vascular cells, and in pravastatin-treated LDLR-/- mice [67]. In contrast both mCx37 and mCx40 have been reported to be down-regulated in aortic endothelial cells from cholesterol-enriched diet mice, with selective recovery of mCx37 but not mCx40 endothelial cell expression by simvastatin treatment [68]. Expression of Cx40 and Cx43 mRNA and protein is reduced both by lovastatin and fluvastatin [66]. Statins have not previously been linked with hCx30.2/31.3 (mCx29), but the possibility that GJC3 is expressed within internal mammary artery and that simvastatin hydroxyacid may interact with its N-terminal sequence offers potential new insight into additional mechanisms for the clinically observed actions of statins.

In order to be functionally active as an inhibitor of HMG CoA reductase, simvastatin used herein is interconverted enzymatically *in vivo* between the lactone and HMG CoA reductase-inhibitory hydroxyacid form [6] and this varies according to genetic subpopulations [5,94]. The pharmacokinetics and clinical effects of statins are also known to be associated with tissue-specific membrane protein expression of anion transporters and neutral ATP binding cassette (ABC)-type transporters [95] and thus this new linkage to what can be classified as a membrane transporter is of particular relevance. An example of how recognition events at N-terminal sequences affect the channel behavior of connexins is the formation of a carbamate within human connexin 26, GJB2 by carbon dioxide [96].

### Biophysical investigation of the new interaction

Further investigation of the uncovered interaction is therefore important and we chose to use surface plasmon resonance [29,97–99] to study the interaction between simvastatin, simvastatin hydroxyacid as its sodium salt, along with fluvastatin, and pravastatin which are both administered as hydroxyacids and the N-terminal polypeptide discovered in the phage display screen. It is recognised that viruses are susceptible to the expression of frame-shifted peptides [100], something we have observed for the T7 phage system [101]. In order to explore this possibility and provide biophysical validation that the uncovered sequence in contig29 interacts in a meaningful way with simvastatin we purchased chemically synthesized peptides A, B, C in the expected reading frame, frame + 1 nucleotide and frame + 2 nucleotides respectively. We presented each peptide on a neutravidin-coated gold SPR chip by adding a C-terminal lysine bearing an ω-NH_2_-coupled biotin moiety (S3 Fig) thereby mimicking its position within the connexin. This approach has previously been used to successfully investigate binding selectivity in many systems, including the discovery of candidate binding partners for the carboxyl terminus of connexin 43 [99] and the first demonstration of C-terminal peptide tail/intracellular loop interactions [98].

No interactions were observed for any of the compounds tested with the out-of-frame peptides B or C (S5 and S6 Figs). While simvastatin *i*.*e*. the lactone showed no interaction with the uncovered sequence contig29 (peptide A), simvastatin acid sodium salt exhibited a weak, concentration dependent binding profile (Fig 12A). This validates the results obtained by the T7 phage system by confirming that the sequence contig29 (and not the out of frame peptides) does interact in a meaningful way with simvastatin. However, this also suggests that hydrolysis to the active, hydroxyacid form has taken place during the immobilisation or biopanning process. In order to investigate this further, fluvastatin and pravastatin (which are both administered in the hydroxyacid form) were investigated. A significant binding profile, exhibiting some non-ideality not untypical of small molecule SPR, was observed for fluvastatin (Fig 12C) while pravastatin showed no interaction with peptide A indicating that the hydroxyacid alone is not sufficient for statin binding to the GJC3 N-terminus (Fig 12D). In order to further investigate contig29 a number of known gap junction (albeit not validated ‘hemichannel’) inhibitors were investigated (niflumic acid, flufenamic acid, carbenoxolone, 18α-glycyrrhetinic acid (Fig 13), 18β-glycyrrhetinic acid, arachidonylethanolamide, and heptanol (S6 Fig, Panel C)) [30] along with compounds sharing functional significance with statins (3-hydroxy-3-methylglutaric acid (S6 Fig, Panel A), or other chemical structural similarities (the sweeteners steviol and stevioside S6 Fig, Panels D and E)). To the best of our knowledge little is known regarding the mechanism of action of the gap junction inhibitors and so it is not clear if these compounds bind directly to particular features within connexin channels, or perhaps act through some other membrane-mediated process [102]. Indeed, differentiating functional activity of connexin 43 hemichannels from pannexin 1 [84,103–105] in assays such as ATP release is recognised as problematic [84,106,107] and we note that carbenoxolone for example is also an inhibitor of pannexin 1 activity [84,86,108]. Investigation of simvastatin, fluvastatin and pravastatin hydroxyacids as potential inhibitors of pannexin 1 (Panx1) channel function is thus warranted.

Fenamates are known reversible inhibitors of gap junctions and so we also chose to investigate binding of niflumic acid and flufenamic acid to peptide A using SPR (Fig 13). Dose dependent binding was observed for both compounds with the most pronounced effect observed for flufenamic acid.

**Fig 14:**
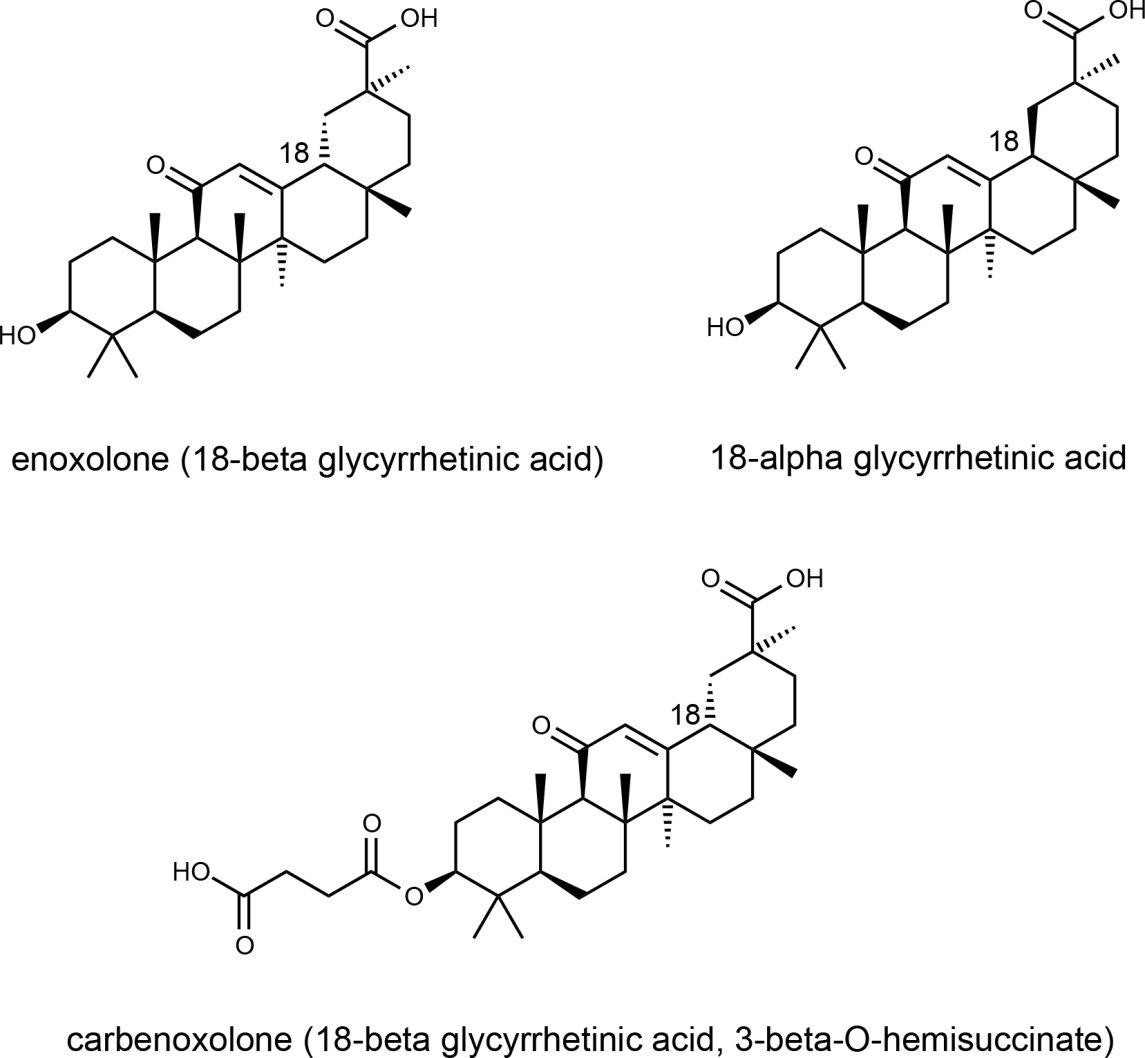
Chemical structures of enoxolone (18β-glycyrrhetinic acid), 18α-glycyrrhetinic acid) and carbenoxolone (18β-glycyrrhetinic acid).

The data presented here suggest that carbenoxolone, a derivative of 18β-glycyrrhetinic acid may function by direct interaction with the N-terminal domain, while 18α-glycyrrhetinic acid, lacking the flexible succinate moiety and 18β-glycyrrhetinic acid (enoxolone, Fig 14) do not. This data should not be over-interpreted, but indicates the latter connexin gap junction inhibitors may have a different selectivity or mode of action, if indeed they act against GJC3 or other hemichannels rather than gap junctions, which does not seem to have been fully explored.

### Functional significance of GJC3 N-terminus

**Fig 15:**
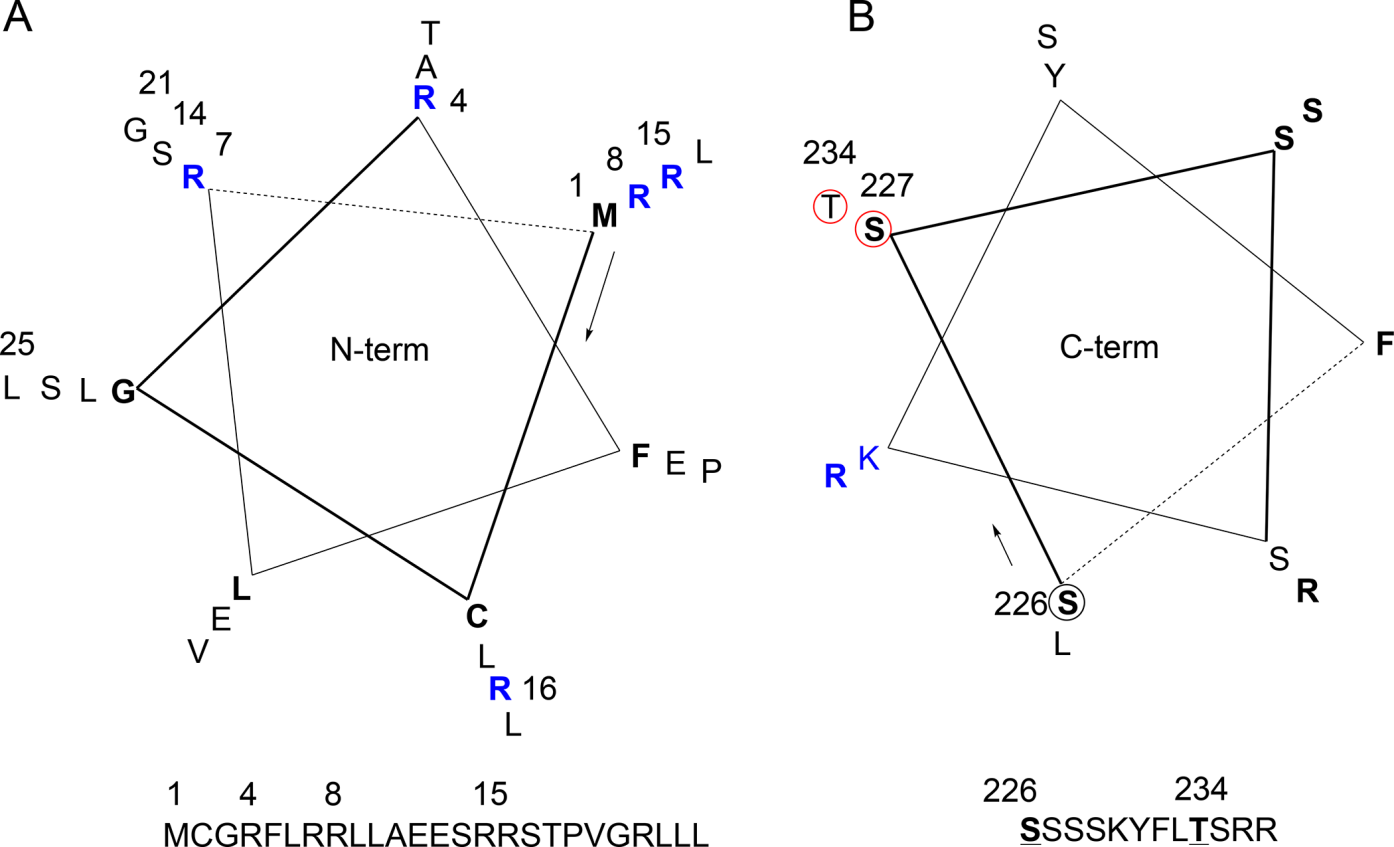
Helical wheel projections of: (A) discovered N-terminal residues 1–25 and (B) C-terminal residues 226–227. Serine 227 and threonine 234 subject to post-translational phosphorylation circled in red.

As noted above, the N-terminus of many connexins is well-known to be important in determining their function as channels [89,109]. For example, in connexin 32 hemichannel the N-terminus has been shown to act as a voltage sensor in a proposed gating mechanism [110] and is recognized as bearing many loss of function genetic mutations associated with Charcot-Marie-Tooth disease [111]. The fact that acidic residues including glutamate and aspartate are conserved in the N-terminus of many connexins (see for example Fig 1 in Beyer *et al*. ref [89]) together with the observed gap junction inhibitory properties of polyamines has led to the investigation of “interfering N-terminal peptides” such as Ac-KLLDK-NH_2_ from Cx43 as inhibitors of gating functions in other connexins including Cx40 [112]. The presence of basic residues in the form of arginines in our discovered sequence at positions 4, 7, 8 in particular is thus striking (see helical wheel, Fig 15 (A)). The statins and the gap junction inhibitors we find to bind to the hCx30.2/31.3 (mCx29) N-terminus by SPR contain at least one, and in the case of carbenoxolone, two carboxylic acid functionalities that might interact with the guanidinium side-chain(s) of these arginine(s). Furthermore, the same arginines could interact with the C-terminal S227, T234 when phosphorylated (Fig 15 (B)), in order to control movement of N- and C-termini and hence access to the channel [113]. As has been proposed for rat Cx43 [114], such a mechanistic hypothesis would enable a serine/threonine kinase e.g. protein kinase C to control the opening of GJC3 in response to an intracellular signal thereby releasing ATP; see for example Kang *et al*. rat Cx43 phosphorylation regulated by IP_3_/IP_3_ receptor [115]. We have now expressed human GJC3 in order to further study the discovered ligand–target interactions and the proposed mechanistic hypothesis.

### Additional sequences identified and a network perspective

From this screen of the new internal mammary artery genomic library we identified an additional six ‘contig’ sequences with sufficient homology and annotated biomedical literature to merit further investigation (S1 Summary). Several are of significance for developing a cardiovascular network of interacting proteins and the statins in order to better contextualize their polypharmacology, which is work in progress. Gap junction inhibitors also possess additional molecular targets, including other channels and transporters (S8 Dataset) and our data will aid further characterizing their in vitro and in vivo activity.

## Conclusions

As test of concept, we applied a new chemical genomics approach, using a viral display library in conjunction with the photo-immobilised simvastatin ligand, to study possible new targets for this widely prescribed lipid-lowering pro-drug. Using these methods, supported by in silico studies, immunohistochemistry and biophysical data, we have provided evidence for the previously unrecognized presence of gap junction gamma-3 protein in arteries from patients with ischaemic heart disease and interaction of simvastatin and fluvastatin hydroxyacids with the N-terminal sequence of this human gap junction gamma-3 protein. We also demonstrate for the first time a selective interaction of the same sequence with the known inhibitors of connexin channel function: niflumic acid, flufenamic acid and carbenoxolone, offering a new entry with which to study their mechanism of action. There are few pharmacological tools with which to probe connexin function making statins high priority for further investigation against these channels.

Our work raises several key questions relevant to understanding aetiology and improving treatment of cardiovascular disease, in particular relating to treatments using statins. Cardiac gap junction channels comprising connexins are known to undergo remodeling in disease [116] and hetero-oligomerisation of connexins is a recognized phenomenon [117,118] raising the possibility that gap junction gamma-3 protein may play a role within homo- or hetero-hexamers in the pathophysiology of human conduit arteries. Interaction of simvastatin hydroxy acid or other statins such as fluvastatin may also thus take place in connexon channels with intercellular conductive functions, as well as hemichannels expected to show extracellular communication as part of signalling microdomains [119]. Whether interaction with gap junction gamma-3 protein contributes to the therapeutic or adverse effects of statin hydroxyacids remains to be investigated and these effects may be tissue-specific. We speculate that the low expression of GJC3 in human liver compared to many other tissues is noteworthy given the high retention of clinically used statins by that organ. A more complete understanding of the biological significance of the novel statin-GJC3 interaction and whether or not particular statins might interact with other connexins is thus important. That knowledge may lead to the development of new classes of treatment with cardiovascular connexins as therapeutic targets [120], perhaps by influencing intercellular conductive function of gap junctions, or connexin derived hemichannels associated with extracellular communication.

## Subjects, Materials and Methods

### Ethics Statement

All patients gave written informed consent and the study was approved by the Local Research Ethics Committee (NHS National Research Ethics Service, Coventry Research Ethics Committee, Coventry UK: ref—07/H1210/126).

We studied the interactions of simvastatin with a viral library of clones prepared from mRNA extracted from internal mammary arteries from 7 patients. Their cardiovascular risk factors included smoking, hypertension, hyperlipidaemia and diabetes mellitus. These samples were obtained as segments of surplus internal mammary artery harvested during coronary artery bypass surgery for treatment of ischaemic heart disease.

All work involving the phage library was carried out under the UK Health and Safety Executive's Genetically Modified Organisms (HSE GMO) guidelines (contained use). Phage library preparation from mRNA, validation, and subsequent work involving the phage library was performed at Tangent Reprofiling Ltd, Warwick HRI, Wellesbourne, Warwickshire, UK.

All plasticware was purchased as RNase-free. RNA*later* RNase inhibitor solution from Ambion [121] (Catalogue #7020) was used to transport the tissues on ice from theatre to the biological lab with at most 30 minutes between extraction of the tissue and processing, or freezing to –80°C. *mir*Vana^™^ miRNA Isolation Kit from Ambion [122,123] (Catalogue #1560, 1561) was used to extract total RNA from the tissues obtained. Straight A’s^™^ mRNA extraction Kit from Novagen (Catalogue #69962–3) was used to extract mRNA from the total RNA samples. OrientExpress^™^ Oligo(dT) Primer cDNA synthesis kit from Novagen (Catalogue #69992–3) and the T7 Select^®^ Phage Display System from Novagen (Catalogue #70550–3) were used to prepare the T7 Phage Display Library. Water used in total RNA and mRNA extraction was RNase free water, purchased from Ambion. Water used in cDNA and Phage Library preparation was Milli-Q filtered sterile water prepared on site.

Tris-buffered saline (TBS: 0.05 M tris(hydroxyethyl)aminomethane (tris), 0.15 M NaCl, pH 7.6 at 25°C) with Tween^®^ 20 surfactant was prepared using 13 TBS tablets (Sigma Aldrich, Cat# T5030) added to Milli-Q^®^ water (195 mL). Two concentrations of Tween^®^ 20 were used, 0.5% and 0.1%, 975 μL and 195 μL respectively.

For preparation of agarose gels, GelRed^®^ (10,000X in water, Biotium, Cambridge Biosciences, Cat# BT41003) was substituted for ethidium bromide, except during library preparation (Fig 4). GelRed^®^ was added to molten agarose solution (5 μL per 100 mL) and not added to the running buffer. All other processes for preparation of agarose gel and loading of wells are identical to those described in the supporting information.

Sequencing Master Mix (per sample): 2 μL Big Dye^®^ (Applied Biosystems, Cat# 4337454), 1 μL 5X sequencing buffer (Applied Biosystems, Cat# 4336697), 0.4 μL T7Select Up Primer, water to make up to 9 μL. Cleaned DNA solution (1 μL) was added before submission for Sanger dideoxynucelotide sequencing. The amount of water in the sequencing PCR master mix was adjusted to account for 1 μL sequencing buffer.

Simvastatin Immobilisation Simvastatin (5 mg, Sigma-Aldrich, Cat# S6196, ≥97% (HPLC) solid) was dissolved in DMSO (1 mL) as a working stock solution (5 mg/mL, 0.012 M, stored at 4°C, allowed to return to room temperature before use). A 100-fold dilution of the stock drug solution was prepared (4 mL, 40 μL in 3.96 mL water, 0.12 mM), and this solution, in a dark room, was added to three wells each of 5 Magic Tag^®^ photochemically active moieties as previously described [13–15] (200 μL, 10 ng, 0.024 nmol per well). Water (200 μL per well) was added to three wells each of the same moieties to form the negative controls. Photochemical activation was achieved by irradiating wells for 10 minutes under a 254 nm UV lamp (220 W). All wells were then washed with water six times and the plate stored at 4°C until use.

### Biopanning

The combined phage library (50 μL library 1 and 50 μL library 2: see library preparation, S1 Protocol) was amplified in 5 mL log phase *E*. *coli* BLT5615 solution at OD_600_ ≈ 0.5, with agitation for 3 h at 37°C. The lysate was centrifuged at 12k × *g* and the supernatant decanted into fresh tubes. For round 1 of biopanning, amplified human vascular phage library (200 μL per well) was added to wells containing bound simvastatin and to control wells containing water, and the plate agitated for 45 minutes. Excess phage solution was then removed and wells washed with 0.5% Tween^®^ in tris-buffered saline (TBS) six times, with 2 minutes agitation for each wash. Log phase *E*. *coli* was prepared as follows. Stock *E*. *coli* strain BLT5615 (1 mL, OD_600_ ~2.0 in lysogeny broth (LB) medium with ampicillin was added to LB medium (100 mL) with ampicillin (1 μL/mL) for 2 h at 37°C. At 1.5 h isopropyl β-D-1-thiogalactopyranoside (IPTG, 500 μL, 100 mM) was added. When this mixture reached OD_600_ ~0.5 it was added to each well (200 μL), and the plate agitated for 10 minutes to elute the remaining bound phage.

The first round well contents were again amplified in log phase *E*. *coli* BLT5615 (5 mL) in labelled 50 mL tubes. The tubes were incubated and shaken for 3 h at 37°C, during which time the solution cleared as the *E*. *coli* were lysed. Aliquots (2 mL) of amplified phage from each tube were separated from any cellular debris by centrifuge and the clarified lysates decanted into fresh, labelled tubes.

The contents of each well from the first round of biopanning (200 μL per well) were then introduced to the next row of immobilized drug and control wells and a second biopanning process performed. On the third round of biopanning, the contents of each well from the elution step were sampled and diluted to 10^−3^, 10^−5^ and 10^−7^, then mixed with stock *E*. *coli* BLT5615 culture and poured on to agarose/agar assay plates directly to avoid any disproportionation of the phage present.

From these agarose lawns, plaques of lysed bacteria were selected that were non-confluent and regular in shape and size to give: (a) 24 phage clones from those wells that contained simvastatin (positive wells, a total of 120 clones) and (b) 12 phage clones from control wells containing water (a total of 60 clones, S2 Dataset). These selected phage clones were submitted to PCR and sequencing (see S1 Dataset). All phage clones and products from PCR were stored at 4°C initially, then archived as glycerol stocks (1:9 80% glycerol solution to phage solution) at –80°C.

### Sequencing

The PCR products were placed in a 96-well Millipore filter plate (Cat# LSKMPCR10) and filtered under vacuum. Water (100 μL) was added and the plate was agitated for 5 minutes. Big Dye™ sequencing master mix (9 μL) was added to each well, followed by DNA solution (1 μL), then submitted for Sanger dideoxynucleotide sequencing. Initial alignment, to identify and remove T7Select^®^ 10–3 vector sequence, correct any base mis-reads, and note highly similar sequences, was carried out using Lasergene^®^ SeqMan Pro (DNAStar Inc.).

### Immunohistochemistry

Sections of internal mammary artery were prepared using standard methods. We performed immunohistochemistry using a rabbit polyclonal antibody [sc-68376, described as “connexin 29 Antibody (H-86)”: Santa Cruz Biotechnology, Inc., Santa Cruz, CA, USA] raised against an epitope corresponding to amino acids 194–279 of NP_853516.1 mapping at the C-terminus of human connexin 30.2/31.3 (NP_853516.1; 86/86 Identities, 86/86 Positives, 0/86 Gaps), to identify the presence and location of human gap junction gamma-3 protein.

### Surface Plasmon Resonance (SPR)

The following chemically synthesized peptides were purchased at >90% purity (Abingdon Health Laboratory Services, UK; HPLC-MS identity and purity analysis).

                1               10                20

Peptide A: MCGRFLRRLL AEESRRSTPV GRLLL—K(biotin)-CONH_2_

Peptide B: CVAGSCGGCW RRRAGAPPPW GASCF—K(biotin)-CONH_2_

Peptide C: VWQVPAAAAG GGEPALHPRG APLAF—K(biotin)-CONH_2_

Surface plasmon resonance sensorgrams were recorded on a BioRad ProteOn XPR36 biosensor. Biotin labelled peptides A, B, C were immobilized from 1 mg/mL solutions in PBS onto a ProteOn NLC Sensor Chip (BioRad, #176–5021), composed of NeutrAvidin immobilized on a modified alginate polymer layer. Exploratory binding experiments were performed using *ca*. 1500 μM or 400 μM solutions (in PBS buffer) of simvastatin (Cayman Chemicals, Cat#CAYM10010344-100), simvastatin sodium salt (Cayman Chemicals, Cat#CAY10010345-10 MG), fluvastatin sodium salt (Cayman Chemicals, Cat#CAYM10010337-100), pravastatin sodium salt (Cayman chemicals, Cat#CAYM10010343), niflumic acid (Sigma-Aldrich Cat#N0630), flufenamic acid (Sigma-Aldrich Cat#F9005), carbenoxolone disodium salt (Sigma-Aldrich Cat#C4790), 18α-glycyrrhetinic acid (Sigma-Aldrich Cat#G8503), 18β-glycyrrhetinic acid (Sigma-Aldrich Cat#G10105), 3-hydroxy-3-methylglutaric acid (Sigma-Aldrich Cat#H4392), 1-heptanol (Sigma-Aldrich Cat#H2805), arachidonylethanolamide (Sigma-Aldrich Cat#A0580), steviol hydrate (Sigma-Aldrich Cat#H8664), and stevioside hydrate (Sigma-Aldrich Cat#S3572). Subsequent titrations were performed in triplicate (simvastatin and fluvastatin hydroxyacid sodium salts, niflumic acid, flufenamic acid and carbenoxolone) or duplicate (simvastatin lactone, pravastatin sodium salt, 3-hydroxy-3-methylglutaric acid, 18β-glycyrrhetinic acid, 18α-glycyrrhetinic acid, arachidonylethanolamide, 1-heptanol, steviol hydrate, and stevioside hydrate) at concentrations of 400 μM, 200 μM, 100 μM, 50 μM, 25 μM 0 μM for each ligand in PBS. Data were analysed at the time using ProteOn Manager™ using both Channel and Interspot referencing (the latter was preferred for final analysis and the data presented herein) then exported as.xlsx files for subsequent analysis using Microsoft Excel^®^.

## Supporting Information

S1 Alignment T-Coffee aligned sequences in.afasta format for GJC3 NP_853516.1 and GJB2_NP003995.2. (AFASTA)

S1 Dataset The complete Sanger dideoxy sequencing data for a biopanning screen of simvastatin, immobilised from solution to a set 5 photochemistries on a 96-well polystyrene plate, versus a genomic T7 phage display created from samples of human vascular tissue total mRNA. (ZIP)

S2 Dataset Negative controls for sequencing of phage which bind to the same 5 photochemistries on a 96-well plate in the absence of simvastatin ligand. (ZIP)

S3 Dataset Normal tissue cell expression of gap junction protein, gamma 3, 30.2kDa (GJC3) analyzed using polyclonal antibody HPA015024. Accessed from *Human Protein Atlas* using keyword GJC3, July 2015 and saved as.xlsx spreadsheet.
(XLSX)

S4 Dataset The *Human Protein Database* annotates 19 gap junction proteins as connexins. http://www.proteinatlas.org/search/connexin with CNST, CAV3, CXADR data removed; exported as TAB data. Saved as.xslx file at http://figshare.com/articles/connexin_annotated_HTA_2015_xlsx/1422052
(XSLX)

S5 Dataset Human GJC3 missense single nucleotide polymorphisms from NCBI present in 1000 Genomes Project data (19 Dec 2015). (TXT)

S6 Dataset Human GJC3 insertion/deletion polymorphisms from NCBI (19 Dec 2015). (TXT)

S7 Dataset GJC3 multiple sequence alignment for human, mouse and rat proteins showing how an alternative site of translation initiation results in extended N-terminus for mCx29. (RTF)

S8 Dataset Gap junction inhibitors and known targets from ChEMBL_20 database. (XLSX)

S9 Dataset Complete set of SPR graphs as.xlsx spreadsheets. (ZIP)

S1 Fig T-Coffee (www.ebi.ac.uk) sequence alignment for GJC3 NP_853516.1 and GJB2 NP_003995.2. Graphical output in Kyte-Doolittle hydrophobicity colour scheme.
(PDF)

S2 Fig Multiple sequence alignment for mammalian GJC3 from HomoloGene 15399. Sequences from *H*. *sapiens*, *Pan trogolodytes*, *Macaca mulatta*, *Canis lupus*, *Bos taurus*, *M*. *musculus*, *R*. *norvegicus*.
(PDF)

S3 Fig Chemically synthesized C-terminal *K*(biotin) labelled peptides. Peptide A in expected reading frame for contig29; peptide B in frame +1 nucleotide, peptide C in frame +2 nucleotides.
(PDF)

S4 Fig Surface plasmon resonance sensorgrams showing injection onto a neutravidin coated chip pre-equilibrated with phosphate buffered saline (PBS): lane 1 peptide A, lane 2 peptide B, lane 3 peptide C. Chromatograms are corrected to the average values of two reference channels containing PBS alone. Solid black line represents the mean of all channels in the corresponding lane with grey areas representing the standard deviation across channels.
(EPS)

S5 Fig Surface plasmon resonance graphs of frame-shifted peptides B and C with statins: (A) simvastatin hydroxyacid, (B) simvastatin lactone, (C) fluvastatin hydroxyacid, and (D) pravastatin hydroxyacid. (TIF)

S6 Fig Surface plasmon resonance graphs of frame-shifted peptides B and C with known connexin inhibitors: (A) niflumic acid, (B) flufenamic acid, (C) carbenoxelone, and (D) 18β-glycyrrhetinic acid. (PDF)

S7 Fig Surface plasmon resonance graphs of peptides A, B, and C with increasing concentrations of: (A) 3-hydroxy-3-methylglutaric acid, (B) arachidonylethanolamide, (C) heptanol, (D) steviol, and (E) stevioside. (PDF)

S1 Protocol Preparation of T7 phage library from human vascular tissue. (DOCX)

S1 Summary Text summary of 30 discovered sequences from T7 phage genomic library screen versus simvastatin on Magic Tag^®^ plates and BLASTX alignment of NCBI *H*. *sapiens* RefSeq protein. From K. Casey-Green PhD thesis, University of Warwick 2011.
(DOCX)

S1 Table Sanger dideoxy sequencing output for 120 phage clones from simvastatin biopan. (DOCX)

S2 Table Phage clones which align with T7 Select^®^ vector sequence. (DOCX)

S3 Table Lasergene SeqMan^®^ analysis of simvastatin biopan sequences to give library contiguous sequences (contigs). (DOCX)

S4 Table Simvastatin biopan contigs BLASTX alignment versus NCBI *H*. *sapiens* RefSeq protein. (DOCX)

S5 Table Tissue specific expression for gap junction protein, gamma 3, 30.2kDa (GJC3) detected at High or Medium expression level in 54 of 79 analyzed normal tissue cell types using polyclonal antibody HPA015024. Data from *Human Protein Atlas* using keyword GJC3, http://www.proteinatlas.org/ENSG00000176402-GJC3/tissue accessed 18 May 2015. ^§^RNA-Seq data expressed as number of *F*ragments *P*er *K*ilobase gene model and *M*illion reads (FPKM): Not detected 0–1; Low 1–10; Medium 10–50; High >50.
(DOCX)
