# Supplementary material for: Simvastatin Sodium Salt and Fluvastatin Interact with Human Gap Junction Gamma-3 Protein
Source: PLoS One. 2016 Feb 10;11(2):e0148266. doi: 10.1371/journal.pone.0148266 (PMC4749215; doi:10.1371/journal.pone.0148266)
Supplement: S7 Dataset — (RTF) [file pone.0148266.s008.rtf]

CLUSTAL W (1.83) multiple sequence alignmentGJC3_human      -----------MCGRFLRRLLAEESRRSTPVGRLLLPVLLGFRLVLLAASGJC3_mouse      MLLLELPIKCRMCGRFLRQLLAQESQHSTPVGRFLLPMLMGFRLLILVSSGJC3_rat        ----------RMCCSFLRQLLARESQHSTPVGRFLLPVLVGFRLLILVSS                           **  ***:***.**::******:***:*:****::*.:*GJC3_human      GPGVYGDEQSEFVCHTQQPGCKAACFDAFHPLSPLRFWVFQVILVAVPSAGJC3_mouse      GPGVFGNDENEFICHLGQPGCKTICYDVFRPLSPLRFWAFQVILMAVPSAGJC3_rat        GPGVFGNDENEFMCHLGQPGCKTICYDVFRPLSPLRFWAFQVILMAVPSA                ****:*:::.**:**  *****: *:*.*:********.*****:*****GJC3_human      LYMGFTLYHVIWHWELSGK-GKEEETLIQGREGNTDVPGAGSLRLLWAYVGJC3_mouse      IYVAFTLYHVIGYWEVPGKENKEQETQISKGDHSKDVSGAKSLKLLWAYVGJC3_rat        IYVAFTLYHVIGYWEVPGR-NKEQEAQICKGGRCKDVSGAMSLKLLWAYV                :*:.******* :**:.*: .**:*: *      .**.** **:******GJC3_human      AQLGARLVLEGAALGLQYHLYGFQMPSSFACRREPCLGSITCNLSRPSEKGJC3_mouse      AHLGVRLALEGAALGVQYNLYGFKMSSTFICREDPCIGSTTCFQSHPSEKGJC3_rat        AHLGVRLVLEGAALGVQYHLYGFKMPSTFICREDPCIGSTTCFQSHPSEK                *:**.**.*******:**:****:*.*:* **.:**:** **  *:****GJC3_human      TIFLKTMFGVSGFCLLFTFLELVLLGLGRWWRTWKHKSSSSKYFLTSESTGJC3_mouse      TIFLNIMFGISGACFLFIFLELALLGLGRFWRIYKHKLSFLKKLPTSESSGJC3_rat        TILLNTMFGISGACLLFIFLELVLLGLGRVWKTYRHKLPLFKNLSTSERS                **:*: ***:** *:** ****.****** *: ::** .  * : *** :GJC3_human      RRHKKATDSLPVVETKEQFQEAVPGRSLAQEKQRPVGPRDAGJC3_mouse      VRSKDTTDELSVVEAKEPF----------------------GJC3_rat        VRHKDTTDDLSVVETKEPF----------------------                 * *.:**.*.***:** *                      
