# Supplementary material for: Simvastatin Sodium Salt and Fluvastatin Interact with Human Gap Junction Gamma-3 Protein
Source: PLoS One. 2016 Feb 10;11(2):e0148266. doi: 10.1371/journal.pone.0148266 (PMC4749215; doi:10.1371/journal.pone.0148266)
Supplement: S1 Fig — Graphical output in Kyte-Doolittle hydrophobicity colour scheme. (PDF) [file pone.0148266.s011.pdf]

```
                               1                    11                   21
Consensus                      M c w r t L q r i L  a e e s k h S T p i  G k i l L p V L l i
Conservation
gi|31559821|ref|NP_853516.1|   M C G R F L R R L L  A E E S R R S T P V  G R L L L P V L L G
gi|42558283|ref|NP_003995.2|   M D W G T L Q T I L  G G V N K H S T S I  G K I W L T V L F I

                               31                   41                   51
Consensus                      F R i m i L a a a a  p e V y G D E Q a e  F V C h T q Q P G C
Conservation
gi|31559821|ref|NP_853516.1|   F R L V L L A A S G  P G V Y G D E Q S E  F V C H T Q Q P G C
gi|42558283|ref|NP_003995.2|   F R I M I L V V A A  K E V W G D E Q A D  F V C N T L Q P G C

                               61                   71                   81
Consensus                      K a a C y D a y h P  i S p i R l W a l Q  l I l V a t P a a L
Conservation
gi|31559821|ref|NP_853516.1|   K A A C F D A F H P  L S P L R F W V F Q  V I L V A V P S A L
gi|42558283|ref|NP_003995.2|   K N V C Y D H Y F P  I S H I R L W A L Q  L I F V S T P A L L

                               91                   101                  111
Consensus                      y a m h t a Y h r -  - i k h k e i s G k  i K s E e k l I q e
Conservation
gi|31559821|ref|NP_853516.1|   Y M G F T L Y H V -  - I W H W E L S G K  G K E E E T L I Q G
gi|42558283|ref|NP_003995.2|   V A M H V A Y R R H  E K K R K F I K G E  I K S E F K D I E E

                               121                  131                  141
Consensus                      i k - t q k V p i a  G S - - L l W a Y t  a q i g a R l i l E
Conservation
gi|31559821|ref|NP_853516.1|   R E G N T D V P G A  G S L R L L W A Y V  A Q L G A R L V L E
gi|42558283|ref|NP_003995.2|   I K - T Q K V R I E  G S - - L W W T Y T  S S I F F R V I F E

                               151                  161                  171
Consensus                      a A a m y l q Y h m  Y - G F q M q s s v  a C r a e P C p g s
Conservation
gi|31559821|ref|NP_853516.1|   G A A L G L Q Y H L  Y - G F Q M P S S F  A C R R E P C L G S
gi|42558283|ref|NP_003995.2|   A A F M Y V F Y V M  Y D G F S M Q R L V  K C N A W P C P N T

                               181                  191                  201
Consensus                      i t C f l S R P s E  K T i F l k t M i a  V S G i C i L l t v
Conservation
gi|31559821|ref|NP_853516.1|   I T C N L S R P S E  K T I F L K T M F G  V S G F C L L F T F
gi|42558283|ref|NP_003995.2|   V D C F V S R P T E  K T V F T V F M I A  V S G I C I L L N V

                               211                  221                  231
Consensus                      l E L c y L l i - R  y - - - - - - - - -  - - - - - - - - - -
Conservation
gi|31559821|ref|NP_853516.1|   L E L V L L G L G R  W W R T W K H K S S  S S K Y F L T S E S
gi|42558283|ref|NP_003995.2|   T E L C Y L L I - R  Y - - - - - - - - -  - - - - - - - - - -

                               241                  251                  261
Consensus                      - - - - - - - - - -  - - - - - - - - - -  - - - - - - - - c a
Conservation
gi|31559821|ref|NP_853516.1|   T R R H K K A T D S  L P V V E T K E Q F  Q E A V P G R S L A
gi|42558283|ref|NP_003995.2|   - - - - - - - - - -  - - - - - - - - - -  - - - - - - - - C S

                               271                  281
Consensus                      q k k q k - - - - -  p a
Conservation
gi|31559821|ref|NP_853516.1|   Q E K Q R P V G P R  D A
gi|42558283|ref|NP_003995.2|   G K S K K - - - - -  P V
```
