# Supplementary material for: Simvastatin Sodium Salt and Fluvastatin Interact with Human Gap Junction Gamma-3 Protein
Source: PLoS One. 2016 Feb 10;11(2):e0148266. doi: 10.1371/journal.pone.0148266 (PMC4749215; doi:10.1371/journal.pone.0148266)
Supplement: S2 Fig — Sequences from H. sapiens, Pan trogolodytes, Macaca mulatta, Canis lupus, Bos taurus, M. musculus, R. norvegicus. (PDF) [file pone.0148266.s012.pdf]

HomoloGene

Display Settings: Multiple Alignment

HomoloGene:15399. Gene conserved in Boreoeutheria   Download , Links

Multiple Sequence Alignment

Generated by MUSCLE [see reference] version 3.6 (using option: -maxiters 2).

```
NP_853516.1         1   ------------MCGRFLRRLLAEESRRSTPVGRLLLPVLLGFRLVLLAA   38
XP_001143440.2      1   ------------MCGRFLRRLLAEESRRSTPVGRLLLPVLLGFRLVLLAA   38
XP_002803266.1          --------------------------------------------------
XP_005621129.1      1   ------------MCGSFLRQLLAEDSRHSTAVGHLLLPVLLGFRLVLLAA   38
NP_001076962.1      1   ------------MCGSFLRRVAAEESRHPTPVGRLLLPALLGLRLVLLAA   38
NP_536698.2         1   -MLLLELPIKCRMCGRFLRQLLAQESQHSTPVGRFLLPMLMGFRLLILVS   49
XP_006249079.1      1   MDCFLEL-----MCCSFLRQLLARESQHSTPVGRFLLPVLVGFRLLILVS   45

NP_853516.1        39   SGPGVY--GDEQSEFVCHTQQPGCKAACFDAFHPLSPLRFWVFQVILVAV   86
XP_001143440.2     39   SGPGVY--GDEQSEFVCHTQQPGCKAACFDAFHPLSPLRFWVFQVILVAV   86
XP_002803266.1      1   -----------------HTQQPGCKAACFDAFHPLSPLRFWVFQVILVAV   33
XP_005621129.1     39   SGTGIY--GDEQSEFVCHTQQAGCKAACYDAFHPFSPLRFWAFQVILVAV   86
NP_001076962.1     39   GGTGVFGGGEEQSEFVCHTQQAGCKAVCYDAFHPLSPLRFWAFQVTLVAV   88
NP_536698.2        50   SGPGVF--GNDENEFICHLGQPGCKTICYDVFRPLSPLRFWAFQVILMAV   97
XP_006249079.1     46   SGPGVF--GNDENEFMCHLGQPGCKTICYDVFRPLSPLRFWAFQVILMAV   93

NP_853516.1        87   PSALYMGFTLYHVIWHWELSGK-GKEEETLIQGREGNTDVPGAGSLRLLW  135
XP_001143440.2     87   PSALYMGFTLYQVIWHWELSGK-GKEEETLIQGREGNTDVPGAGSLRLLW  135
XP_002803266.1     34   PSALYMGFTLYHVIWHWELSGK-GKEEETLIQGGEGNTDVPGAGSLRLLW   82
XP_005621129.1     87   PSTLYMGLILYHVIWRWEESGK-VK-EETLIHQGEKSRDASGAGSPRLLW  134
NP_001076962.1     89   PSALYMGFILYHVIWHWEASEK-VKTEEETLSQGEKGGEASRAGSSRLLW  137
NP_536698.2        98   PSAIYVAFTLYHVIGYWEVPGKENKEQETQISKGDHSKDVSGAKSLKLLW  147
XP_006249079.1     94   PSAIYVAFTLYHVIGYWEVPGR-NKEQEAQICKGGRCKDVSGAMSLKLLW  142

NP_853516.1       136   AYVAQLGARLVLEGAALGLQYHLYGFQMPSSFACRREPCLGSITCNLSRP  185
XP_001143440.2    136   AYVAQLGARLVLEGAALGLQYHLYGFQMPSSFACRREPCLGSITCNLSRP  185
XP_002803266.1     83   AYVAQLGARLVLEGTALGLQYHLYGFQMPSSFACRREPCLGSITCHLSRP  132
XP_005621129.1    135   AYVAQLGVRLVLEGAALGLQYHLYGFKIPSSFACRREPCLGSITCYLSRP  184
NP_001076962.1    138   AYVAQLGVRLALEGAALGGQYHLYGFRMPSSFVCRLEPCLGSTNCYLSRP  187
NP_536698.2       148   AYVAHLGVRLALEGAALGVQYNLYGFKMSSTFICREDPCIGSTTCFQSHP  197
XP_006249079.1    143   AYVAHLGVRLVLEGAALGVQYHLYGFKMPSTFICREDPCIGSTTCFQSHP  192

NP_853516.1       186   SEKTIFLKTMFGVSGFCLLFTFLELVLLGLGRWWRTWKHKSSSSKYFLTS  235
XP_001143440.2    186   SEKTIFLKTMFGVSGFCLLFTFLELVLLGLGRWWRTWKHKSSSSKYFPTS  235
XP_002803266.1    133   SEKTIFLKTMFGVSGFCLLFTFLELVLLGLGRWWRTWKHKPSSSKYFPTS  182
XP_005621129.1    185   FEKTIFLKTMFGVSGLCLLFTLVELVLLGLGKWWRTWKHK-SPSNYSSTS  233
NP_001076962.1    188   SEKSIFLKTMFGVTGLCLLFTLLELVLLGLGRWWRIWRHKSPSSNYSPTS  237
NP_536698.2       198   SEKTIFLNIMFGISGACFLFIFLELALLGLGRFWRIYKHKLSFLKKLPTS  247
XP_006249079.1    193   SEKTILLNTMFGISGACLLFIFLELVLLGLGRVWKTYRHKLPLFKNLSTS  242

NP_853516.1       236   ESTRRHKKATDSLPVVETKEQFQEAVPGRSLAQEKQRPVGPRDA  279
XP_001143440.2    236   ESTRRHKKATDSLPVVETKEQFQEAVPGRSLAQEKQRPVGPRDA  279
XP_002803266.1    183   ESTRRHKEATDSLPVVETKEQFQEAVPGRSSAQEKQRPVGPRDA  226
XP_005621129.1    234   ESTKKHKDPTDNFPAVEAKEQFQEA-----------------EL  260
NP_001076962.1    238   QSAKRCKAPTDNFPVVEIRERPGEAGERGSEVPLSARP------  275
NP_536698.2       248   ESSVRSKDTTDELSVVEAKEPF----------------------  269
XP_006249079.1    243   ERSVRHKDTTDDLSVVETKEPF----------------------  264
```

Protein Acc.	Gene	Organism
NP_853516.1	GJC3	H.sapiens
XP_001143440.2	GJC3	P.troglodytes
XP_002803266.1	LOC100424576	M.mulatta
XP_005621129.1	GJC3	C.lupus

NP_001076962.1	GJC3	B.taurus
NP_536698.2	Gjc3	M.musculus
XP_006249079.1	Gjc3	R.norvegicus
