# Supplementary material for: Simvastatin Sodium Salt and Fluvastatin Interact with Human Gap Junction Gamma-3 Protein
Source: PLoS One. 2016 Feb 10;11(2):e0148266. doi: 10.1371/journal.pone.0148266 (PMC4749215; doi:10.1371/journal.pone.0148266)
Supplement: S3 Fig — Peptide A in expected reading frame for contig29; peptide B in frame +1 nucleotide, peptide C in frame +2 nucleotides. (PDF) [file pone.0148266.s013.pdf]

residue	1	11	21
**Peptide A:**	MCGRFLRRLL	AEESRRSTPV	GRLLL*K*(biotin)$CONH_2$
**Peptide B:**	CVAGSCGGCW	RRRAGAPPPW	GASCF*K*(biotin)$CONH_2$
**Peptide C:**	VWQVPAAAAG	GGEPALHPRG	APLAF*K*(biotin)$CONH_2$
