# Supplementary figures and images for: Simvastatin Sodium Salt and Fluvastatin Interact with Human Gap Junction Gamma-3 Protein
Source: PLoS One. 2016 Feb 10;11(2):e0148266. doi: 10.1371/journal.pone.0148266 (PMC4749215; doi:10.1371/journal.pone.0148266)

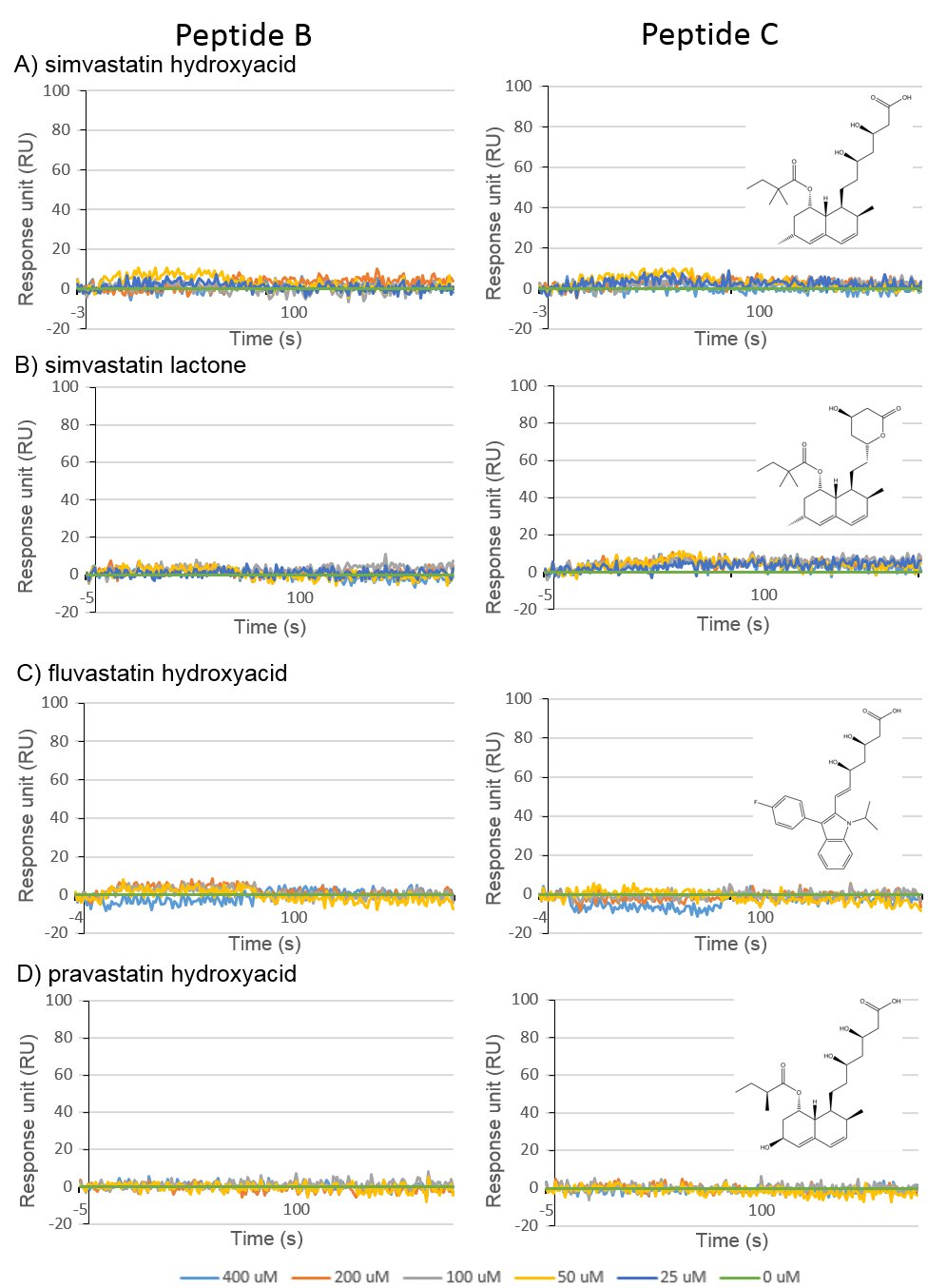

Supplement: S5 Fig — (TIF) [file pone.0148266.s015.tif]

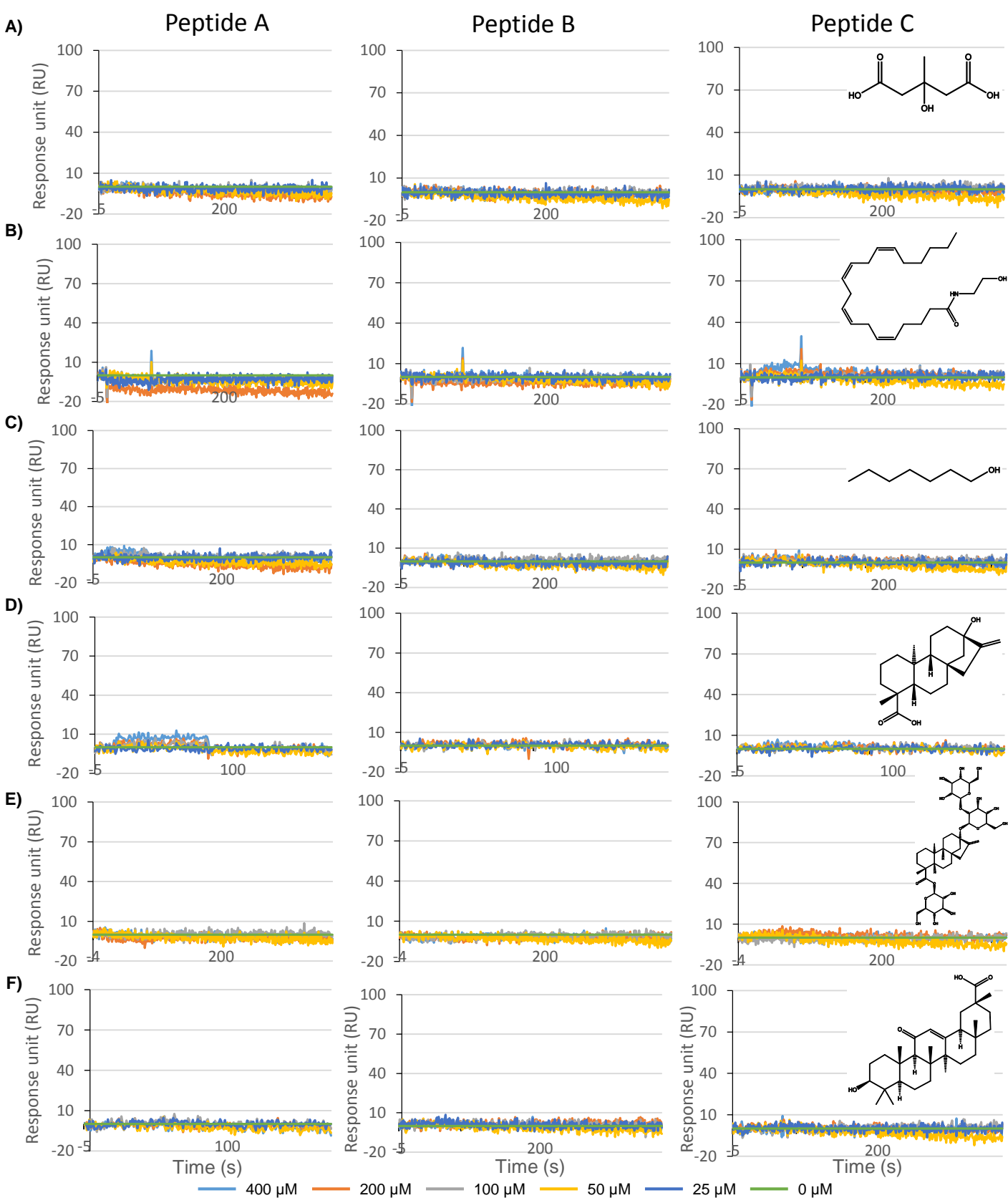

Supplement: S7 Fig — (PDF) [file pone.0148266.s017.pdf]
