# Supplementary material for: Simvastatin Sodium Salt and Fluvastatin Interact with Human Gap Junction Gamma-3 Protein
Source: PLoS One. 2016 Feb 10;11(2):e0148266. doi: 10.1371/journal.pone.0148266 (PMC4749215; doi:10.1371/journal.pone.0148266)
Supplement: S6 Fig — (PDF) [file pone.0148266.s016.pdf]

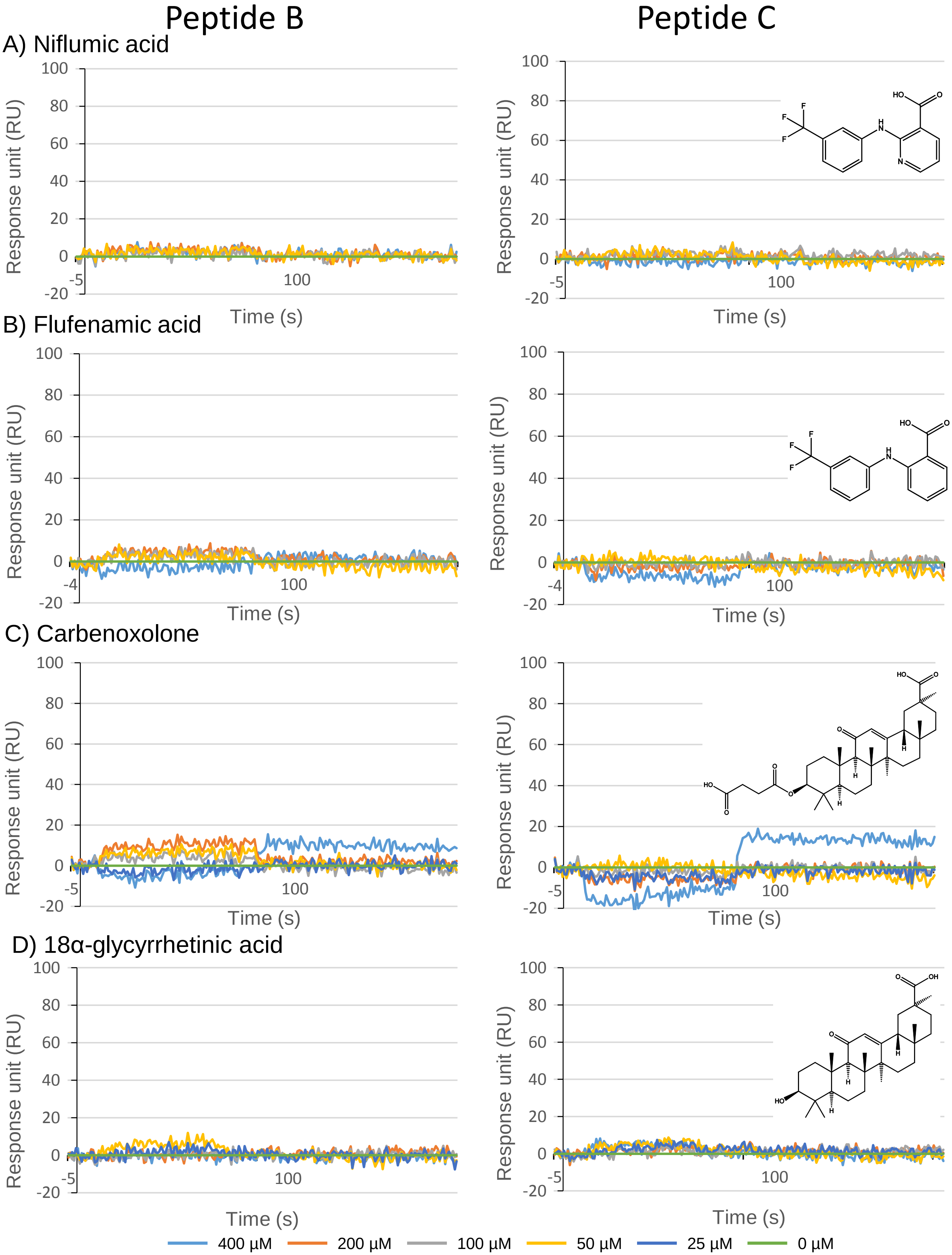

Peptide B
Peptide C
A) Niflumic acid
B) Flufenamic acid
C) Carbenoxolone
D) 18α-glycyrrhetinic acid
Response unit (RU)
Time (s)
100
80
60
40
20
0
-20
-5
-4
HO
O
F
N
H
HO
OH
400 µM
200 µM
100 µM
50 µM
25 µM
0 µM
