# Supplementary material for: Simvastatin Sodium Salt and Fluvastatin Interact with Human Gap Junction Gamma-3 Protein
Source: PLoS One. 2016 Feb 10;11(2):e0148266. doi: 10.1371/journal.pone.0148266 (PMC4749215; doi:10.1371/journal.pone.0148266)
Supplement: S1 Protocol — (DOCX) [file pone.0148266.s018.docx]

## Preparation of T7 phage library.

### General Reagents and Media

**Preparation of *E. coli* BLT5615 working plates and working stocks**: *E. coli* strain BLT5615 (Glycerol Stock, Novagen, Cat# 69905) was taken from the –80 °C freezer and kept on ice to minimise defrosting of the sample. The top of the frozen glycerol stock was scraped using a sterile loop which was streaked across an agar plate containing ampicillin, along one edge only without damaging the agar surface. Using a new sterile loop, the plate was turned by 60° and streaked again, spreading the *E. coli* solution across a fresh edge of the plate. This turning and streaking was repeated twice further and the plate sealed with Parafilm^®^, inverted and incubated overnight at 37 °C. After growth, the plates were stored at 4 °C. To prepare a working (overnight) stock of *E. coli* one cleanly defined colony was removed from the plate using a P1000 pipette tip and placed in 100 mL liquid LB in a 250 mL flask to allow adequate aeration. This flask was agitated at 37 °C overnight to give an OD_600_ ~ 2.0. This stock *E. coli* solution was stored at 4 °C for up to two weeks.

**Standard PCR mastermix** (per sample): 5 µl 10X Nova*Taq^™^* buffer with MgCl_2_ (Novagen, Cat#71005), 1 µL T7Select^®^ Up primer (5 pmol/ µL), 1 µL T7Select^®^ Down primer (5 pmol/ µl) (Sigma Genosys Primers), 1 µL dNTP mix (10 mM each dNTP) (Invitrogen, Cat#18427-013), 0.25 µL Nova*Taq^™^* DNA polymerase (1.25U x 12) (Novagen, Cat#71005), made up to 49 µL with water.

**Touchdown PCR mastermix** (per sample): 5 μL 10X Nova*Taq^™^* buffer with MgCl_2_, 1 µL T7Select^®^ Up primer (5 pmol/ µL), 1 μL T7 Select Down primer (5 pmol/ µL), 1 µL dNTP mix (10mM each dNTP), 0.25 µl Nova*Taq^™^* DNA polymerase (1.25Ux12), made up to 40 µL with water.

**Standard PCR protocol**: Phage pick solution (1 µL) and PCR mix (49 µL) was added to the wells of the PCR plate and PCR was set up to run: 3 mins, 94 °C, 35 cycles: 0.5 mins, 94 °C; 1 min, 50 °C; 1 min, 72 °C, final extension: 6 mins, 72 °C; hold at 25 °C.

**For agarose gel electrophoresis**, gels were prepared as follows – TAE (100 mL, 1X) and agarose (1 g) were melted in microwave (scaled up for larger gels). Ethidium bromide (10 μL, 1 mg/mL, 10 µg) was added and poured into a gel frame and allowed to set with combs in place to prepare the running lanes. Ethidium bromide (50 µL) was added to the running solution (1X TAE, 500 mL).

After the gel was set the comb was removed and the PCR product (8 µL) was loaded with 20% volume Crystal 5X DNA loading buffer blue (Bioline, Cat# BIO-37045). 5 ul Hyperladder^™^ I (Bioline, Cat# Bio-33025) was added to the lanes on either side of the samples being assayed. Where two combs were used, two lanes of Hyperladder^™^ I were added on both rows. The gel was run in the electrophoresis module at 100-130 V for 30-45 mins. Gel was visualised under UV light and photographed.

PCR products were subjected to cleanup using Millipore filter plates (Millipore, Cat# LSKMPCR10) under vacuum before sequencing.

**Sequencing mastermix** (per sample): 2 μL Big Dye^®^ Terminator v3.1 (Applied Biosystems, Cat# 4337454), 0.4 µL T7Select^®^ Up Primer, water to make up to 9 µL. Cleaned DNA solution (1 μL) added before submission for sequencing.

### Tissue Sample Collection

Artery and vein material was collected from surgery in cryogenic tubes containing a RNase inhibitor solution RNAlater^®^, for transport in ice back to the research lab (30 minutes maximum transport time).

Sample size ranged from 3896 - 61 mg for the internal mammary artery samples, average 957 mg ± 670 mg and 548 - 43 mg for the saphenous vein samples, average 165 mg ± 170 mg. These were divided into smaller samples (998 - 56 mg for the internal mammary artery samples, average 279 mg ± 130 mg and 213 - 43 mg for the saphenous vein samples, average 120 mg ± 56 mg) for later processing. Samples were not weighed until work with the respective sample was about to start, to avoid prolonged exposure of the sample to room temperature, risking degradation of any nucleic material. Any RNase inhibitor was removed and the sample was frozen and stored at –80°C.

### Total RNA extraction

The sample was weighed and 10:1 volume:mass (mL:g) lysis/binding buffer was added to the tissue. The sample was homogenised using a mechanical homogeniser until no further lumps of tissue were visible. Homogenate (0.1 eq. volume of lysis/binding buffer) was added and mixed by brief vortexing. The solution was separated into two tubes, and any remaining lumps of adipose tissue were left behind. Phenol:chloroform:isoamyl alcohol (1 eq. volume of lysis/binding buffer) was added, followed by 1-bromo-3-chloropropane (0.1 eq. volume lysis/binding buffer). The tubes were mixed thoroughly by inverting and vortexing, then centrifuged at 12k x *g* for 5 mins. The aqueous layer was removed without disturbing the protein band at the interface and placed in a new tube, recording the approximate volume of material. Ethanol (neat, 1.25 volumes) was added to precipitate the nucleic acids. While a tube of RNase free water was being heated to 95 °C, the ethanol solution was added to a filter cartridge in a collecting tube in 700 μl volumes and the solution was drawn through under centrifuge (12k x *g*, 10 secs). The run-through from the filter was discarded and after all the material had been added, the product was washed with Wash 1 solution (700 μl, ethanol based solution containing guanidinium thiocyanate to denature any remaining proteins) and drawn through under centrifuge (12k x *g*, 5 secs), then Wash solution 2/3 (500 μl x 2, ethanol based solution containing EDTA to protect RNA from damage caused by any metal present when eluted from the filter) and drawn through under centrifuge (12k x *g*, 5 secs). Any remaining solution was removed from the filter by centrifuge (12k x *g*, 1 minute) and the collecting tube was discarded. The filter was placed in a fresh tube and the RNA was eluted with 95 °C RNase free water (50-150 μl).

The concentration and purity of RNA was determined by NanoDrop[[1](#_ENREF_1)] assay and the RNA solution was stored at –80 °C. An average of 58 1 µg ± 35 µg total RNA was extracted from each internal mammary artery sample of average 273 mg ± 136 mg and an average of 29 µg ± 14 µg extracted from each saphenous vein sample of average mass 120 mg ± 56 mg, using the Ambion mirVana^™^ miRNA Isolation Kit.

### Extraction of mRNA [[2](#_ENREF_2)]

Dithiotheritol (DTT, 1mM) and the total RNA samples were thawed in the fridge and on ice respectively. The volume of particles required for the extraction was calculated from the known amount of total RNA, and the volume of lysis buffer required to make the concentration of samples up to 20% of total volume was also calculated. The samples of total RNA were pooled and if necessary concentrated to a concentration of over 0.5 mg/mL (carried out by adding 2 volume equivalents of ethanol to the pooled samples on dry ice and leaving to precipitate for ten minutes). The solution was then spun at 12k x *g* for 20 mins at 4 °C; all supernatant was removed and the sample redissolved in the appropriate amount of RNase free water. NanoDrop assay was carried out before continuing to confirm sample concentration. Four particle volumes of lysis buffer were made up (1 mL buffer to 10 μl DTT). One volume of the particles was pipetted out, captured in the magnetic stand and the supernatant removed. The particles were then washed with two sequential volumes of the lysis buffer. One further volume of lysis buffer plus the extra lysis buffer to make the concentration of total RNA up to 20% were added to the particles and they were incubated at room temperature for 5 mins. The particles were captured and the supernatant removed, then washed with 2 volumes of wash buffer and all solution was removed from the tube. RNase free water (0.5 mL) was added and the tube was incubated for ten minutes at 60 °C to elute the mRNA. The supernatant, now containing the mRNA, was transferred to a clean tube and glycogen (2 μl), sodium acetate (50 μl) and isopropyl alcohol (330 μl) were added. The tube was then centrifuged for ten minutes at 12k x *g*, following which the supernatant was removed (the pellet is not visible at this point). Ethanol (70% v/v, 0.5 mL) was added and the tube was spun for a further 5 mins. The supernatant was removed and the mRNA was dissolved in RNase free water (0.02 mL). The concentration of the mRNA was determined by NanoDrop assay and stored at –80 °C.

Twelve samples from seven patients were sampled, and a pool of 290 µL of 0.58 ng/μL total RNA was collected. From this pooled total RNA, 2.48 µg PolyA+ mRNA was extracted using the Novagen Straight A's^™^ mRNA Isolation Kit.

### cDNA Library

The formation of a cDNA library from an mRNA sample was carried out using Novagen's T7Select^®^10-3 OrientExpress^™^ cDNA Cloning System and mRNA starting material MT1801. NanoDrop: 123.5 ng/µL (A260/280 = 1.81 and A260/230 = 2.29). For this study, oligo(dT) primed cDNA synthesis was chosen.

mRNA (1.99 µg, 16.1 µL MT1801) and oligo(dT) primer (1 µg at 1 µg/μL) was mixed, and made up to 20 μL with water. This was incubated at 70 °C for ten minutes and chilled on ice, followed by 12k x *g* centrifuge for ten seconds.

First strand buffer (5X, 10 µL), DTT (100mM, 5 µL), methylation dNTP mix (10X, 2.5 µL) was added and made up to 46 µL with water. Solution was mixed and equilibrated for 1 min at 37 °C. MMLV reverse transcriptase (4 µL, 800 units) was added and incubated at 37 °C for a further 60 mins. The reaction mixture was then heated at 70 °C for ten minutes, cooled on ice and centrifuged briefly to collect the product.

Second strand buffer (5X, 50 µL), DTT (100mM, 6 µL), methylation dNTP mix (10X, 2 µL), DNA polymerase (5 µL, 50units) and RNase H (1.6 µL, 1.6 units) were added and made up to 250 µL total volume. This was then incubated at r.t. for 90 minutes.

TE buffered phenyl:choloroform:isoamyl alcohol (25:24:1, 250 μL) was added and vortexed for 30 seconds, followed by centrifugation for 1 minute at 12 x *g*. The aqueous phase was removed to fresh tube. Glycogen (1 µL), ammonium acetate (4M, 250 µL) and isopropanol (300 µL) were added to precipitated the nucleotide material. The reaction mixture was then inverted several times and centrifuged at 12 x *g* for 8 mins. No pellet was visible, so the reaction was centrifuged for a further 3 mins and the supernatant was removed. The pellet was washed with 70% ethanol and centrifuged for 3 mins. Repeated with 100% ethanol. The washed pellet was then suspended in TE buffer (20 µL) and stored overnight at -20 °C.

Flush buffer (3 µL), DTT (100mM, 1.5 µL), dNTPs (3 µL, 1mM made up from 10mM solution – 1 µL in 9 µL water), T4 DNA polymerase (0.6 µL 1.5units) were added with water to make up to 30 µl. Solution was mixed gently and allowed to incubate at r.t. for 20 mins. TE buffer (20 µL) was added followed by TE buffered phenyl:choloroform:isoamyl alcohol (50 µL). This mixture was vortexed for 30 seconds and centrifuged at 12k x *g* for 1 min. The aqueous layer was removed to a fresh tube. This was repeated with further TE buffered phenyl:choloroform:isoamyl alcohol (50 µL). Glycogen (1 µL), ammonium acetate (4M, 50 µL) and ethanol (250 µL) were added and mixed well and stored at -20 °C for 5 h. Product was centrifuged at 12k x *g* for 10 mins wherepon the supernatant was removed and the resultant pellet washed with 70% ethanol and centrifuged for 3 mins. Repeated with 100% ethanol and then suspended in TE buffer (10 μL).

Ligation buffer (10X, 2 µL), ATP (2 µL, 1mM made up from 10mM solution – 1 µL in 9 µL water), DTT (100mM, 2 µL), *Eco*RI (2 µL), T4 polynucleotide kinase (0.5 µL, 5units) were added and made up to 20 µl (including volume addition of T4 DNA ligase in later step). The mixture was incubated for 5 mins at 37 °C, and placed on ice. T4 DNA ligase (1.75 µL, 7 units) were added. This was incubated at room temperature overnight.

The ligase was inactivated by incubating the reaction at 70 °C for ten minutes. *Hin*dIII buffer (10X, 10 µL), *Hin*d III (5 µL, 100 units) were added and made up to a total volume of 100 µl. This mixture was incubated at 37 °C for 2 h. 10 μL *Eco*RI adjustment buffer and *Eco*RI (5 µL, 100units) added. This was incubated at 37 °C for 4 h.

A mini-column was filled with gel filtration resin (2 mL), equilibrated with column buffer (5 x 1 mL 1X made up as 700 µL buffer in 6.3 mL Milli-Q water). The cDNA sample was extracted with phenol:choloroform:isoamyl alcohol (115 µL), and the aqueous layer was removed to prepared column. The sample was allowed to settle into the column (fraction 1). Washed through with 200 µL column buffer (fraction 2). Washed through with 2 x 250 µL column buffer (fractions 3 and 4), washed through with 250 µL water (fraction 5).

To fractions 3 and 4 was added glycogen (1 µL), isopropyl alcohol (150 µL) - salt is already present in column. The precipitation was allowed to incubate for 5 mins at room temperature. This mixture was spun for ten minutes at 12k x *g* and washed with 70% and 100% ethanol. The final pellet was dissolved in TE Buffer (10 µL).

Fraction 3 was initially dissolved in TE buffer (20 µL) but reprecipitated in glycogen, ammonium acetate and ethanol at -20 °C, followed by washing with 70% and 100% ethanol. The resulting cDNA was analysed by NanoDrop assay and stored at -20 °C.

MT31F3: *NanoDrop:* 11.0 ng/µL, *A_260/280_ =* 1.99, *A_260/230_ =* 2.21

MT31F4: *NanoDrop:* 33.3 ng/µL, *A_260/280_ =* 1.88, *A_260/230_ =* 2.46

### Packaging into Phage Vector Arms

To MT31F4 (1 µL, 3.33 ng) was added ligation buffer (0.5 µL, 10X), ATP (0.5 µL, 10mM), DTT (0.5 µL, 100mM), T7 Select Vector Arms (0.8 µL, 0.5 µg, 0.02 pmol), T4 DNA ligase (1 µL, 0.4 U / μL, diluted from 4 U / µL: 1 µL in 9 µL water) and made up to a total volume of 5 µL, then mixed by pipette tip and incubated at 18 °C overnight. Reaction mixture was added to T7 Phage Packaging Extract (25 µL) which was incubated at 20 °C for 2 h.

Aliquots (2x1 µL, D1 and D2) were taken from the mixture and stored for diversification assay – see below. Stock *E. coli* strain BLT5615 (500 µL, OD_600_ ~2.0 in LB medium with ampicillin - see general experimental for recipe) was added to LB medium (5 mL) with ampicillin (1 µL/mL) for 2 h at 37 °C. At 1.5 h IPTG (500 µL, 100 mM) was added. The remaining reaction mixture was split into two halves (14 µL each), and each was added to *E. coli* (2.5 mL, OD_600_ ~0.5) as prepared above, giving two amplification reactions. These were shaken at 37 °C for 3.5 h and then centrifuged at 8000 rpm for 10 mins. The two supernatants were decanted off and stored separately at 4 °C as Library 1 and Library 2. These two libraries were eventually combined and after amplification a series of samples were taken to prepare glycerol stocks for storage at –80 °C. Glycerol stocks were prepared as 1:9 80% glycerol solution to LB phage solution.

### Phage Library Validation

As a diversification assay the two 1 µL aliquots taken from the unamplified library, D1 and D2 were added to water (99 µL) and then added to *E. coli* solution (100 µL, OD_600_ ~2.0) and IPTG (100 µL, 100 mM). Molten LB agarose (3 mL, ~50˚C) was added and the mixture was spread across the top of a prewarmed agar plate containing ampicillin (1 µL/mL). This agarose layer was allowed to set, and the plate was inverted and incubated at 37 °C for 4 h. The *E. coli* lawns on these plates were completely lysed, giving an undefined diversity of >10^6^.

To quantify the amplified library, the phage solution was diluted to four different dilutions (10^-2^ - 10^-8^) and 100 µL of each was added to *E. coli* solution (100 µL, OD_600_ ~2.0) and IPTG (100 µL, 100 mM). Molten LB agarose (3 mL, ~50˚C) was added and the mixture was spread across the top of a prewarmed agar plate containing ampicillin (1 µL/mL). This agarose layer was allowed to set, and the plate was inverted and incubated at 37 °C for 4 h (or until plaques appeared evenly across the plate). In the dilutions of 10^-2^ and 10^-4^ the *E. coli* lawn was completely lysed, but in the case of the 10^-6^ and 10^-8^ dilutions, there were a countable number of plaques visible. Several plaques were picked from each of the two libraries were taken (1 plug of agarose in 100 µL water) and a PCR mastermix was made up - see General Experimental.

Phage pick solution (1 µL) and PCR mix (49 µL) was added to the wells of the PCR plate and PCR was run - cycle program described in general experimental. An agarose gel was prepared for electrophoresis. Ethidium bromide (80 µL) was added to the running solution (1X TAE, 800 mL). PCR was repeated using touchdown PCR technique.

Amplification was repeated on the two libraries (100 µL of amplified library in log phase *E. coli* 5 mL, OD_600_ ~0.5) and these were poured onto plates in dilutions as above. Sixteen new plaques were picked. See main text for gel results.

The PCR product was introduced into a 96-well Millipore filter plate (Millipore, Cat#LSKMPCR10) and filtered under vacuum. Water (100 µL) was added and the plate was agitated for 5 mins before collection of the clean PCR product solution. Big Dye^™^ sequencing master mix was prepared as described in the general experimental and 9 µL was added to each well, followed by 1 µL DNA solution. This plate was submitted for sequencing.

Picked clone solutions were archived as glycerol stocks (1:9 80% glycerol solution to phage solution) and stored at –80 °C.

### References

1. Kidwell H (March 2008) Nanodrop Makes Low Volume Analysis a Cinch. labtechnologistcom.

2. Sambrook J, Fritsch EF, Maniatis T (1989) Molecular Cloning, A Laboratory Manual. New York: CSHL Press.
