# Supplementary material for: Simvastatin Sodium Salt and Fluvastatin Interact with Human Gap Junction Gamma-3 Protein
Source: PLoS One. 2016 Feb 10;11(2):e0148266. doi: 10.1371/journal.pone.0148266 (PMC4749215; doi:10.1371/journal.pone.0148266)
Supplement: S1 Summary — From K. Casey-Green PhD thesis, University of Warwick 2011. (DOCX) [file pone.0148266.s019.docx]

**NCBI Database: Homo sapiens RefSeq protein**

Query= Simvastatin Contig 3

Length=302

>ref|NP_001002.1| 40S ribosomal protein S7 [Homo sapiens]

Length=194

Score = 171 bits (432), Expect = 2e-43

Identities = 85/85 (100%), Positives = 85/85 (100%), Gaps = 0/85 (0%)

Frame = +3

Query 3 TKNKQKRPRSRTLTAVHDAILEDLVFPSEIVGKRIRVKLDGSRLIKVHLDKAQQNNVEHK 182

TKNKQKRPRSRTLTAVHDAILEDLVFPSEIVGKRIRVKLDGSRLIKVHLDKAQQNNVEHK

Sbjct 110 TKNKQKRPRSRTLTAVHDAILEDLVFPSEIVGKRIRVKLDGSRLIKVHLDKAQQNNVEHK 169

Query 183 VETFSGVYKKLTGKDVNFEFPEFQL 257

VETFSGVYKKLTGKDVNFEFPEFQL

Sbjct 170 VETFSGVYKKLTGKDVNFEFPEFQL 194

Query= Simvastatin Contig 7

Length=218

>ref|NP_001158312.1| LYR motif-containing protein 4 isoform 2 [Homo sapiens]

Length=130

Score = 89.4 bits (220), Expect = 7e-19

Identities = 43/61 (71%), Positives = 46/61 (76%), Gaps = 0/61 (0%)

Frame = -3

Query 213 EMKSHSVTQPGVQWCNLS*LQPPPPRFKQFFCLSLPSSWDYRHAPSCMANFCIFSRDGIS 34

+M SHSV Q GV W +LS LQP PP FKQF CLSLPSSWDYR P +ANFCI SRD IS

Sbjct 69 QMDSHSVAQAGVHWNDLSSLQPLPPWFKQFSCLSLPSSWDYRRTPPRLANFCILSRDVIS 128

Query 33 L 31

L

Sbjct 129 L 129

Query= Simvastatin Contig 14

Length=387

>ref|NP_001158011.1| disrupted in schizophrenia 1 protein isoform c [Homo sapiens]

Length=755

Score = 95.1 bits (235), Expect = 1e-20

Identities = 51/69 (74%), Positives = 53/69 (77%), Gaps = 1/69 (1%)

Frame = -2

Query 326 SLQPPPPEFTPFSCLSLPSS*DYRRPPACLALFFVFFVEIGFRHVAQAGLGFLNSSDPPA 147

SLQP PPEF FSCLSL SS DYR PP CLA F VF VE+GF HV Q GL L SSDPP+

Sbjct 678 SLQPLPPEFKQFSCLSLRSSWDYRCPPPCLANF-VFLVEMGFYHVDQTGLKLLTSSDPPS 736

Query 146 SASQSAGIT 120

SASQSAGIT

Sbjct 737 SASQSAGIT 745

Query= Simvastatin Contig 15

Length=369

>ref|NP_872601.1| histone demethylase UTY isoform 1 [Homo sapiens]

Length=1079

Score = 38.9 bits (89), Expect(3) = 2e-11

Identities = 17/19 (90%), Positives = 18/19 (95%), Gaps = 0/19 (0%)

Frame = +1

Query 64 VETGFHHVGQAALKLLTSG 120

VETGFHHVGQA L+LLTSG

Sbjct 1041 VETGFHHVGQACLELLTSG 1059

Score = 37.0 bits (84), Expect(3) = 2e-11

Identities = 14/20 (70%), Positives = 17/20 (85%), Gaps = 0/20 (0%)

Frame = +2

Query 2 LPSSWDYRRPPSRPSNFCIF 61

LP+SW+YR PS P+NFCIF

**S4 Summary** Discovered sequences from T7 phage genomic library screen versus simvastatin on Magic Tag^®^ plates. From K. Casey-Green PhD thesis, University of Warwick 2011.

Sbjct 1021 LPNSWNYRHLPSCPTNFCIF 1040

Score = 27.7 bits (60), Expect(3) = 2e-11

Identities = 13/16 (82%), Positives = 13/16 (82%), Gaps = 0/16 (0%)

Frame = +3

Query 132 SASQSGGITGVSHCTR 179

SASQS GITGVSH R

Sbjct 1064 SASQSAGITGVSHHAR 1079

Query= Simvastatin Contig 23

Length=164

>ref|NP_001158312.1| LYR motif-containing protein 4 isoform 2 [Homo sapiens]

Length=130

Score = 80.1 bits (196), Expect = 4e-16

Identities = 37/53 (70%), Positives = 40/53 (76%), Gaps = 0/53 (0%)

Frame = -1

Query 164 EMKSHSVTQPGVQWCNLS*LQPPPPRFKQFFCLSLPSSWDYRHAPSCMANFCI 6

+M SHSV Q GV W +LS LQP PP FKQF CLSLPSSWDYR P +ANFCI

Sbjct 69 QMDSHSVAQAGVHWNDLSSLQPLPPWFKQFSCLSLPSSWDYRRTPPRLANFCI 121

Query= Simvastatin Contig 26

Length=460

>ref|NP_000423.2| myosin regulatory light chain 2, ventricular/cardiac muscle isoform

[Homo sapiens]

Length=166

Score = 151 bits (382), Expect = 1e-37

Identities = 73/73 (100%), Positives = 73/73 (100%), Gaps = 0/73 (0%)

Frame = +2

Query 2 DPEETILNAFKVFDPEGKGVLKADYVREMLTTQAERFSKEEVDQMFAAFPPDVTGNLDYK 181

DPEETILNAFKVFDPEGKGVLKADYVREMLTTQAERFSKEEVDQMFAAFPPDVTGNLDYK

Sbjct 94 DPEETILNAFKVFDPEGKGVLKADYVREMLTTQAERFSKEEVDQMFAAFPPDVTGNLDYK 153

Query 182 NLVHIITHGEEKD 220

NLVHIITHGEEKD

Sbjct 154 NLVHIITHGEEKD 166

Query= Simvastatin Contig 28

Length=155

>ref|NP_963998.2| thromboxane A2 receptor isoform beta [Homo sapiens]

Length=407

Score = 35.8 bits (81), Expect = 0.009

Identities = 16/22 (73%), Positives = 19/22 (87%), Gaps = 0/22 (0%)

Frame = -1

Query 155 ASASQSAGITGVSHCA*PIFLY 90

ASAS++AGITGVSHCA P L+

Sbjct 359 ASASRAAGITGVSHCARPCMLF 380

Query= Simvastatin Contig 29

Length=175

>ref|NP_853516.1| gap junction gamma-3 protein [Homo sapiens]

Length=279

Score = 52.4 bits (124), Expect = 9e-08

Identities = 25/25 (100%), Positives = 25/25 (100%), Gaps = 0/25 (0%)

Frame = -1

Query 76 MCGRFLRRLLAEESRRSTPVGRLLL 2

MCGRFLRRLLAEESRRSTPVGRLLL

Sbjct 1 MCGRFLRRLLAEESRRSTPVGRLLL 25

Query= Simvastatin Contig 31

Length=470

>ref|NP_060190.2| signal-transducing adaptor protein 2 isoform 1 [Homo sapiens]

Length=449

Score = 60.1 bits (144), Expect = 4e-10

Identities = 31/40 (78%), Positives = 32/40 (80%), Gaps = 1/40 (2%)

Frame = +2

Query 2 ETGFHPVCQAGLELLTSGSPPTSSSQSAGWITGVSHHIRP 121

E GFH V QAGLELLTS PPTS+SQSAG ITGVSHH P

Sbjct 359 EKGFHHVAQAGLELLTSSDPPTSASQSAG-ITGVSHHTWP 397

**S4 Summary** Discovered sequences from T7 phage genomic library screen versus simvastatin on Magic Tag plates. From K. Casey-Green PhD thesis, University of Warwick 2011.
