# Supplementary material for: Simvastatin Sodium Salt and Fluvastatin Interact with Human Gap Junction Gamma-3 Protein
Source: PLoS One. 2016 Feb 10;11(2):e0148266. doi: 10.1371/journal.pone.0148266 (PMC4749215; doi:10.1371/journal.pone.0148266)
Supplement: S1 Table — (DOCX) [file pone.0148266.s020.docx]

## S1 Simvastatin Biopan *vs*. Human Vascular Tissue Library Sequences

| Clone ID | Sequence |
| --- | --- |
| SimvaBP1 | CCGGNANTCGTCGANAAGCTTGCGGCCGCACTCNANNNNCTNNNNAACCCCTTGGGGCCTCTAAACGGGTCTTGANGGGTTAACTAGTTANTCGAGTGNGGNNGCNNGCTTGT |
| SimvaBP2 | CTGGTNCCATTCCTTCTGAAACTATTCCAATCAATAGAAAAAGAGGGAATCCTCCCTAACTCATTTTATGAGGCCAGCATCATTCTGATACCAAAGCTGGGCAGAGACACAACCAAAAAAGAGAATTTTAGCCCAATATCCTTGATGAACATTGATGCAAAAATCCTCAATAAAATACTGGCAAAACAAATCCAGCAGCTCATCAAAAAGCTTGCGGCCGCACTCGAGTAACTAGTTAACCCCTTGGGGCCTCTAAACGGGTCTTGAGGGGTTAACT |
| SimvaBP3 | TCCCGGGANCTCGTCGANAAGCTTGCGGCCGCACTCGANTAACTAGTTAACCCCTTGGGGCCTCTAAACGGGTCTTGAGGGNNNNNNNNGTTACTNGAGTGCGG |
| SimvaAP4 | CGATTNCCTGANGTCAGGANTTCAATACCACCCTGGCCAACATAGTGAAATCCCNTCTCTACTAAAAATACAAAAATTAGCCATGCATGATGGTGCATGCCGATAGTCCCAGCTACTAGGGAGGCTGAGGCAGAAGAATTGCTTGAACCTGGGAGGTGGAGGTTGCAGTCAGCTGAGATTGCACCACTGTACTCCAGGCTGGGTGACAGAGTGAGACTTCATCTC |
| SimvaAP5 | CTTGCGGCCGCACTCNNNNNNNNNNNNNNNNNNNNGGGGCCTCTAAACGGGTCTTGAGGG |
| SimvaAP6 | GGTACCATTCCTTCTGAACTATTCCAATCANNNNNNNNNNANNNAATCCTCCCTAACTCATTTTATGAGGCCANNNNCNTTCTGATACCAAAGCTGGGCAGAGACACAACCAAAAAAGAGAATTTTAGCCCAATATCCTTGATGAACATTGATGCAAAAATCCTCAATAAAATACTGGCAAAACAAATCCAGCAGCTCATCAAAAAGCTTGCGGCCGCACTCGAGTAACTAGTTAACCCCTTGGGGCCTCTAAACGG |
| SimvaDP7 | TCCCGGGANCTCGTCGANAAGCTTGCGGCCGCACTCGANNANCTNNTTAACCCCTTGGGGCCTCTAAACGGGTCTTGAGGGGTTAACTAGTTACTCGAGTGCGGNCGCNAGCTTGTCGACGAGCT |
| SimvaDP8 | No Signal |
| SimvaDP9 | CCTCCNGCCTCANCCTCCCAAGTAGCTGGGATTACAGACGCCCGCCATCTCGTCCGTCTAATTTTTGTATTTTTTAGTAGAGACGGGGTTTCACCACGTTGGCCAGGCTGCTCTCAAACTCCTGACCTCAGGTGATCCGCCACCTCAGCCTCCCAAAGTGGTGGGATTACAGGCGTGAGCCACTGCACCCGGCCTAGAATTTCTTTAAACAATAAGACTTCTGATATGTAGCTTGAAGAGAGAAATGACTATTCAGATATGATCGTCAGGTATACATCCAAACCTTTCTAAAGAAAAGCCTAGGATTAAGCTTGCGGCCGCACTCGAGTAACTAGTTAACCCCTTGGGGCCTCTAAACGGGTCTTGAGGGGTTAACTAGTTACTCGAGTGCGG |
| SimvaCP10 | ATCCCGGGANCTCGTCGANAAGCTTGCGGCCGCACTCGANNNNCNAGTTAACCCCTTGGGGCCTCTAAACGGGTCTTGAGGGGTTAACNAGNNACTCGAGTGCGG |
| SimvaCP11 | ATGGGAAGGANCCTGGGGTGCTGGAACCTCTTCCTTATGTCATAATAGGTATTAAAAAAAAAGACCTCTGGACTGAAAAAAATCAAGAGAAGATTTACAATGCATGGAAATAACTGTAATTTATCATATTAATTTGGGCTACCAGTAGCATCGTTTAGAGATTGTGCAAATTGAGTTCACAAATATCACAGCTTTGGAAGATCAAGAAGTGGTTTTGAACATAGAGATTCCAGGGAAAAAAAGTAATAATAAGATT |
| SimvaBP12 | TGACTGTAGAACTCTTCCTTTTGTTGTTTTCACNAAGAGACTGAAAACATGGCAGTGAACTGCCTGAGGTTTTCATTCTCTGTTCATCTCTAATTTTATCTGGACTCTATTGTCCCTGCCCTAGTCAACTGGGAATCTATTCTCAACATTTGCTCCTCAATGTAGGGCTTTGTCATGGAAAGTCAGTTTGGTTTGTTACTTTCAAGAATTCTGTGATTAAAGTAAGCTGGACGCACAGATTTTAAAACATAATTGGATTAATTGACATTTTTCTGTCCCTACTTTATTAGAAACATTATGCATTTAGCTTTTCAAATATATACAGTTTTTTTACTCTAAACTTGACACTTCTAGAGGGCCAGTATTAGATTTTATGCTATATGAAAGCTACCTCTCTATCTTGAACTAGAACCATACCAAAGCTTGCGGCCGCACTCNAG |
| SimvaBP13 | AGAACCCCATCTCTAAAAAAAGAAAAAAAATCGTATAAAAAATAAAAAGTAAAAAAAGAAAAAATTACTAAACAAAGAAACAAACAAAATATGGTAGCACATATTGTAATGCCAAAGGTTCTTGCCTTAGCCACGCCAAAGAATTAGTGTGGCGGCTGCCCGCGGAGAGTGATGGAGACACGGACCAAGAGAAAAAAAGCTGTAGGCTTTATTGAGCAGAGTGACAGTACAAAGCTTGCGGCCGCACTCGAGTAACTAGTTAACCCCTTGGGGCCTCTAAACGGGTCTTGAGGGGTTAAC |
| SimvaBP14 | AAAAGGCAATAAATTAAAATACACTTCAAGGTAAATTANAAACACTGTTATTTATTTCTTTATTCATTTATTTATTTACTTTAGAGACATGGTCTCACTCTGTTGCCACAATCATAGCTCACTGCAGCCTCAAGCTTGCGGCCGCACTCGAGTAACTAGTTAACCCCTTGGGGCCTCTAAACGGGTCTTGAGGGGTTAACTAGTT |
| SimvaAP15 | No Signal |
| SimvaAP16 | No Signal |
| SimvaAP17 | TCCCGNGANCTCGTCGACAAGCTTGCGGCCGCACTCGNNNNNNNNNNNNNNNNNNTGGGGCCTCTAAACGGGTCTTGAGGG |
| SimvaDP18 | TCCCGGNNNNCGTCNANAGCTTGCGGCCGCACTCGANNNNNNNNNAANNNNTTGGGGCCTCTAAACGGGTCTTGAGGGGT |
| SimvaDP19 | GTCNACAGCTTGCGGCCGCACTCGNNNNNNNNNNNNNNNNNNNNGGGGCCTCTAAACGGGTCTTGAGG |
| SimvaDP20 | GANCTCCAGCCACCCCGCAGTCACTTTCTTTGTAACAACTTCCGTTGCTGCCATCGTAAACTGACACANNGTTTATAACGTGTACATACATTAACTTATTACCTCATTTTGTNATTTTTCGAAACAAAGCCCTGTGGAAGAAAATGGAAAACTTGANNAAGCATTAAAGTCATTCTGTTAAGCTGCGAAAAAAAAAAAAAA |
| SimvaCP21 | No Signal |
| SimvaCP22 | CANGGNNNTCCCGGGANCTCGTCGANAAGCTTGCGGCCGCACTCGAGTAACTAGTTAACCCCTTGGGGCCTCTAAACGGGTCTTGAGGGGTTAACTAGTTACTCGAGTGCGGCCGCAAGCNTGTCGACGAGNNCCCGGGATATCCCTGCAGGAGAATTCGGATCA |
| SimvaBP23 | ACCAACTGCAGAGAGGAGCTACTCACTGTGNNNNTTCTCTGAGCTGTTCTAATACTAAATAAAGTTCTTCGTCTTCTTCACTCTTCACTTGTCTGCGTACCTCATTCTTCCCAGATGCAGGACGAGAACTCGGACAAAGACGCCATGGAGGTTTCCAGGAAGAAAATCAACACCAATCAACTCCCCAAAGATCCCGAAACAAAGGATCCTAATGTACTTGCTACTCCCGTCCCATCAGGACCAATCAGCATAGTGTCAATGGTGTGGCCCACTATGGTTTTCTGTGGCATCTCTGTGGATGCTATCATGGCAGAGAGAGCAGGAAAACTGTCATAACCCTGAGGCAAACTGGGA |
| SimvaBP24 | CAGCCCATAAAATCATTTTTCTCTCTTTGGCCTCCAGGCCTGTGACGGGAAGTGCTGCTGTGGTTTCTGACATGCCCCGGANACATTTTCCCCATTGTCTTGGNAATTAACCTTGGGCTCCTTGTTACATATGCTAATTTCTGCAGCAGGCTTGAATTTCTCCCAAGAAAATCTTTATTTCTTTTCTATCGCATCATCAGACTGCAAATTTTCCAAACTTTTATGNNCTGCTTCCTCTTGAACGCTTTGTTG |
| SimvaBP25 | CCGGGNGCTCGTCGACAAGCTTGCGGCCGCACTCGANNNNNNNNNNNNNNNNNNTGGGGCCTCTAAACGGGTCTTGAGGGG |
| SimvaAP26 | TCCCGNGNNNTCGTCGACAAGCTTGCGGCCGCACTCGANNNNNNNNNNNNANNNNNTGGGGCCTCTAAACGGGTCTTGAGGG |
| SimvaAP27 | No Signal |
| SimvaAP28 | ACAGCTTGCGGCCGCCTCNNNNNNNNNNNNNNNNNNTGGGGCCTCTAAACGGGNCTTGAGG |
| SimvaDP29 | GGNTNTCCCGGGNGCTCGTCGANAGCTTGCGGCCGCACTCGANNNNNNNNNNNAANCNCTTGGGGCCTCTAAACGGGTCTTGAGGGGTTA |
| SimvaDP30 | No Signal |
| SimvaDP31 | No Signal |
| SimvaCP32 | No Signal |
| SimvaCP33 | GTACAAAAAATAAGCAAAAGCGTCCCAGGAGCCGTACTCTGACAGCTGTGCACGATGCCATCCTTGAGGACTTGGTCTTCCCAAGCGAAATTGTGGGCAAGAGAATCCGCGTCAAACTAGATGGCAGCCGGCTCATAAAGGTTCATTTGGACAAAGCACAGCAGAACAATGTGGAACACAAGGTTGAAACTTTTTCTGGTGTCTATAAGAAGCTCACGGGCAAGGATGTTAATTTTGAATTCCCAGAGTTTCAATTGTAAACAAAAATGACTAAATAAAAAGTATATATTCACAGTAAAAAAAAAAAAAAAA |
| SimvaBP34 | No Signal |
| SimvaBP35 | AGCGNNTNCCTGANGTCAGGAGTTCAATACCACCCTGGCCAACATAGTGAAATCCCATCTCTACTAAAAATACAAAAATTAGCCATGCATGATGGTGCATGCCGATAGTCCCAGCTACTAGGGAGGCTGAGGCAGAAGAATTGCTTGAACCTGGGAGGTGGAGGTTGCAGTCAGCTGAGATTGCACCACTGTACTCCAGGCTGGGTGACAGAGTGAGACTTCATCTCA |
| SimvaBP36 | GGCTGTTGTTGGTGTACAGGAATGTTAGNGATTTTTGCACATTGATTTTGTATTGTGAGACTTTGCTGAAGTTGTTTATCAGCTTAAGGAGCTTTTGGACTGAGACTGTGGGGTTTTCTAGATATAGGATTATGTCATCTGCAAACAGGGATAGTTTGACTTCTTCTCTTCTTATTTGGATGCCCTTTATTTCTTTCTCTTGTCTAATTGCCCTGNCNANGACTTCCAATACTATGTTGAATAGGAGTGGNGAGAGAGGGCATCCTTGTCTTGNGCTGATTTTCAAGGGGAATGCTTCCAGCTTTTGCCCATTCAGTATGATGTTGGCTGTGGGTCGAAAAAAAAAAAAA |
| SimvaAP37 | No Signal |
| SimvaAP38 | No Signal |
| SimvaAP39 | ACTAAAAATACAAAAATTAGCCATGCATGATGGNGCATGCCGATAGNCCCAGCTACTAGGGAGGCTGAGGCAGAAGAATTGCTTGAACCTGGGAGGTGGAGGTTGCAGTCAGCTGANATTGCACNNCTGTACTCCAGGCTGGGTGACAGAGTGAGACTTCATCTC |
| SimvaDP40 | AAAGAGGGAATCCTCCCTAACTCATTTTATGAGGCCAGCATCATTCTGATACCAAAGCTGGGCAGAGACACAACCAAAAAAGAGAATTTTAGCCCAATATCCTTGATGAACATTGATGCAAAAATCCTCAATAAAATACTGGCAAAACAAATCCAGCAGCTCATCAAAAAGCTTGCGGCCGCACTCGAGTAACTAGTTAACCCCTTGGGGCCTCTAAACGGGTCTTGAGGGGTTAA |
| SimvaDP41 | No Signal |
| SimvaDP42 | No Signal |
| SimvaCP43 | TAATAGGTATTAAAAAAAAAAGACCTCTGGNCTGANAAAAATCAAGAGAAGATTTACAATGCATGGAAATAACTGTAATTTATCATATTAATTTGGGCTACCAGTAGCATCGTTTAGAGATTGTGCAAATTGAGTTCACAAATNTCACAGCTTTGGNNNANCAAGAAGTGGTTTTGAACATANAGATTCCAGGGAAAAAAAGTAATAATAAGATTAAAA |
| SimvaCP44 | No Signal |
| SimvaBP45 | AAGAGGGAATCCTCCCTAACTCATTTTATGAGGCCAGCNTCATTCTGATACCAAAGCTGGGCAGAGACACAACCAAAAAAGAGAATTTTAGCCCAATATCCTTGATGAACATTGATGCAAAAATCCTCAATAAAATACTGGCAAAACAAATCCAGCAGCTCATCAAAAAGCTTGCGGCCGCACTCGAGTAACTAGTTAACCCCTTGGGGCCTCTAAACGGGTCTTGAGGGGTTAA |
| SimvaBP46 | TACTCCAGGCCAGGAATGTCCAAAATTTTGGCTTCCCCTGGGCCACATCATAGAAGAATTGTCTTGGGCCACCCATAAAATACACTAACACTAATAATAGCTGATGAGCTAAAAAAAAAAAAAAAAT |
| SimvaBP47 | GAACTGGTNCCATTCCTTCTGAAACTATTCCAANNNNNNNNNAAAGAGGGAATCCTCCCTAACTCATTTTATGAGGCCAGCATCATTCTGATACCAAAGCTGGGCAGAGACACAACCAAAAAAGAGAATTTTAGCCCAATATCCTTGATGAACATTGATGCAAAAATCCTCAATAAAATACTGGCAAAACAAATCCAGCAGCTCATCAAAAAGCTTGCGGCCGCACTCGAGTAACTAGTTAACCCCTTGGGGCCTCTAAACGGGTCTTGAGGGGTTAACTAG |
| SimvaAP48 | No Signal |
| SimvaAP49 | No Signal |
| SimvaAP50 | No Signal |
| SimvaDP51 | ATCCCGGGANCTCGTCGANAAGCTTGCGGCCGCACTCGANTNNCTANTTAACCCCTTGGGGCCTCTAAACGGGTCTTGAGGGGTTAACTNGTTANTCGAGTGCGGCCGCAAGC |
| SimvaDP52 | No Signal |
| SimvaDP53 | AGAGGGAATCCTCCCTAACTCATTTTATGAGGCCANCNTCNTTCTGATACCAAAGCTGGGCAGAGACACAACCAAAAAAGAGAATTTTAGCCCAATATCCTTGATGAACATTGATGCAAAAATCCTCAATAAAATACTGGCAAAACAAATCCAGCAGCTCATCAAAAAGCTTGCGGCCGCACTCGAGTAACTAGTTAACCCCTTGGGGCCTCTAAACGGGTCTTGAGGGGTTAA |
| SimvaCP54 | No Signal |
| SimvaCP55 | AGCCTGCTCTCANCCAGANCAGGACAGANGANGGTCAGCCAGAGGCCGGANAGANGNTGGGATGACCAACTGCAGAGAGGAGCTACTCACTGTGGGTCTTCTCTGAGCTGTTCTAATACTAAATAAAGTTCTTCGTCTTCTTCACTCTTCACTTGTCTGCGTACCTCATTCTTCCCAGATGCAGGACGAGAACTCGGACAAAGACGCCATGGAGGTTTCCAGGAAGAAAATCAACACCAATCAACTCCCCAAAGATCCCGAAACAAAGGATCCTAATGTACTTGCTACTCCCGTCCCATCAGGACCAATCAGCATAGTGTCAATGGTGTGGCCCACTATGGTTTTCTGTGGCATCTCTGTGGATGCTATCATGGCAGAGAGAGCAGGAAAACTGGCATAACCCTGAGGCAAACTGGGAAGGGGTGCTGTGGGTGCCCTAGACACGAGCAGACTGTTTCTGGTGTTCCTGGAAATTGACATAGAGATTAAGAGCCAACTCTCAGTGCTGCGGGGCTGCAACTGTGGCTCCCAATGAAGAAGCTTGCGGCCGCACTCGAGTAACTAGTTAACCCCTTGGGGCCTCTAAACGGGTC |
| SimvaBP56 | CCGGGAGCTCGTCGANAAGCTTGCGGCCGCACTCGNNNNNNNNNNNAACCCCTTGGGGCCTCTAAACGGGTCTTGAGGGGTTA |
| SimvaBP57 | CCTTTATAAAGCCAAAGAAACCCCACAAAAAAACCTTCAACTATTCGAACAATGGTAAATTTTAGAAAAGACTACATTAGCATTAAAAAAAGATCTAAGTTCCTTCAGAAATTGACTTTCATATATGCCACACTGCAGCCCAAAGACAGAAAAATAATAGTGGTTTATGACTATATGTTATGACTTTCCTAAATCTATGGAGTAAGTCCTAAACATAGAAAAGAAAGACATCATTCACATCTCTCTGTAACAAACTTCTTACCTTCTTAAAGGAAGCACAGAAGCTTGCGGCCGCACTCGAGTAACTAGTTAACCCCTTGGGGCCTCTAAACGGGTCTTGAGGGGTTAACTAGTT |
| SimvaBP58 | GGACCCTGAGGAAACCATTCTCAACGCATTCNANGTGTTTGACCCTGAAGGCAAAGGGGTGCTGAAGGCTGATTACGTTCGGGAAATGCTGACCACGCAGGCGGAGAGGTTTTCCAAGGAGGAGGTTGACCAGATGTTCGCCGCCTTCCCCCCTGACGTGACTGGCAACTTGGACTACAAGAACCTGGTGCACATCATCACCCACGGAGAAGAGAAGGACTAGGAGGGGGCTCGCTGCTGCGCCCTGGGCTCGTCTTTGCNNAGTGGTCCCTGCCCTCATCTCTCTCCCCCGAGTACCGCCTCTGTCCCTACCTTGNCTGTTAGCCATGTGGCTGCCCCATTTATCCACCTCCNNCTTCNTTGCAGCCTGGGNGGCNATGGGTACTTCGTGGCCGCNCATCCTACAGTTGGNNATCCATCCNGAGGCNNTGTTCCNATAAACAGGANGTCGTGTAAAAAAAAAAAAAAAA |
| SimvaAP59 | No Signal |
| SimvaAP60 | No Signal |
| SimvaAP61 | No Signal |
| SimvaDP62 | GGAAAAAGGCAATAAATTAAAATACACTTCAAGGTAAATTANAAACACTGTTATTTATTTCTTTATTCATTTATTTATTTACTTTAGAGACATGGTCTCACTCTGTTGCCACAATCATAGCTCACTGCAGCCTCAAGCTTGCGGCCGCACTCGAGTAACTAGTTAACCCCTTGGGGCCTCTAAACGGGTCTTGAGGGGTTAACTAG |
| SimvaDP63 | No Signal |
| SimvaDP64 | AAAAAGAGGGAATCCTCCCTAACTCATTTTATGAGGCCAGCATCATTCTGATACCAAAGCTGGGCAGAGACACAACCAAAAAAGAGAATTTTAGCCCAATATCCTTGATGAACATTGATGCAAAAATCCTCAATAAAATACTGGCAAAACAAATCCAGCAGCTCATCAAAAAGCTTGCGGCCGCACTCGAGTAACTAGTTAACCCCTTGGGGCCTCTAAACGGGTCTTGAGGGGTTAACTAGTTAC |
| SimvaCP65 | ATAGTCCCAGCTACTAGGGAGGCTGAGGCAGAAGAATTGCTTGAACCTGGGAGGTGGAGGTTGCAGTCAGCTGAGATTGCACCACTGTACTCCAGGCTGGGTGACAGAGTGAGACTTCATCTCA |
| SimvaCP66 | No Signal |
| SimvaBP67 | TTGCGGCCGCACTCNNNNNNNNNNNNNNNNNCTTGGGGCCTCTAAACGGGTCTTGAGGGGT |
| SimvaBP68 | CAGAAACTATAATATTTTAGGAGTTGTAAGAATACTATGTAAAACATGTTTCACATAAAAATGTTNATATGAATTACATAAATTTTTAAATATAGAAAGATTGGTCAAGCGCAATGGNTCACGCCTGTAATTCCAGCACTTTGAGAGGCCGAGGCNNGNGGANNNCGGTCAGGAGATCGAGACCATCCTGGCTAACACTGTGAANNCCTGTGTCNACCAAAAATNCAANAAATTAGCTGGGCGTGGTAGCGGGTGCCTGTAGTCCCAGCTACTTGGNAGGNTGAGGCAGGAGAATG |
| SimvaBP69 | No Signal |
| SimvaAP70 | No Signal |
| SimvaAP71 | No Signal |
| SimvaAP72 | ACGGGAAGCAAGAGGCGCCCCACGGGGGNGGAGCGNCGGCTCTCCTCCGCCAGCAGCCGCCGCAGGAACCTGCCACACATCCTGTTTTGGAGCAGAGGACAAGAGATCAGTGTTGTTCACTGTCCTTCAGAGGGAGCTCCAGTCCACCTTGTCACTCCTAATCCAAGGATCACTGAAGGCAAGCTTGCGGCCGCACTCGAGTAACTAGTTAACCCCTTGGGGCCTCTAAACGGGTCTTGAGGGGTTAA |
| SimvaDP73 | CCCGGGNGCTCGCNACAGCTTGCGGCCGCCTCGANNNNNNNNNANNNNNNGGGGCCTCTAAACGGGTCTTGAGGG |
| SimvaDP74 | No Signal |
| SimvaDP75 | No Signal |
| SimvaCP76 | TTCTGATACCAAAGCTGGGCAGAGACACAACCAAAAAAGAGAATTTTAGCCCAATATCCTTGATGAACATTGATGCNAANNTCCTCAATAAAATACTGGCAAAACAAATCCAGCAGCTCATCAAAAAGCTTGCGGCCGCACTCGAGTANCTAGTTAACCCCTTGGGGCCTCTAAACGGGTCTTGAGGGGTTA |
| SimvaCP77 | AGNAGAGACGGGGTTTCATCCTGTTTGTCAGGCTGGTCTTGAACTCCTGACCTCAGGCAGTCCACCCACCTCATCCTCACAAAGTGCTGGGTGGATTACAGGTGTAAGCCACCACATCCGGCCACTAGTATTTTATTTTTTTTAGGGTGGTAAATGTAATGGACTCACAAATTCTTTCCAAGGGATTATGGACCTTCGGTATTTGAAATAAAAAGACAGTTGGAATTTTTTGCTTCCGATAGTAAGACTATACTGGTCAGGCACTGTCTATTCTGATGGAGCAGCTGTTGCTGCTTGGCTGNCTTTCAGAAGCAAGCTGCTCACACTGATATTGGTTGGTGAGCAAGGCCAGTGGTCATTGATCGATTGACTAGATTTTNAACTGGCTCTGGCTGGCTTCTTGTTACCATGGCTACAGGTCANTTCNTTCCTAAGTTTGAGTCAAACTTTAACCAGAAATTTTTCTGTTCAAA |
| SimvaBP78 | No Signal |
| SimvaBP79 | No Signal |
| SimvaBP80 | No Signal |
| SimvaAP81 | No Signal |
| SimvaAP82 | No Signal |
| SimvaAP83 | No Signal |
| SimvaDP84 | No Signal |
| SimvaDP85 | TCCCGNNNNNCGTCGACAGCTTGCGGCCGCCTCNNNNNNNNNNNNNNNNNTTGGGGCCTCTAAACGGGTCTTGAGGGGN |
| SimvaDP86 | No Signal |
| SimvaCP87 | TGAGTGTGTTTTATTAATGAAGGGTAGAGAGCACAGCAGCTAAACAGAAACTATAATATTTTAGGAGTTGTAACAATACTATGTAAAACATGTTTCACATAAAAATGTTAATATGAATTACATAAATTTTTAAATATAGAAAGATTGGNCAAGCGCAATGGCTCACGCCTGTAATTCCAGCACTTTTGANAGGCTCGAGGCATGTGGATCACGGTCAGGAGATCGAGACCTTCCTGGCTAACACTGTNAAACCCTGTGTCTACC |
| SimvaCP88 | TCTACTAAAAATACAAAAATTAGCCATGCATGATGGTGCATGCCGATAGTCCCAGCTACTAGGGAGGCTGAGGCAGAAGAATTGCTTGAACCTGGGAGGTGGAGGTTGCAGTCAGCTGAGATTGCACCACTGTACTCCAGGCTGGGTGACAGAGTGAGACTTCATCTCAAAAAAA |
| SimvaCP89 | No Signal |
| SimvaEP90 | TCCTCCCTAACTCNTTTTATGANGCCAGCATCATTCTGATACCAAAGCTGGNCAGAGACACAACCAAAAAAGAGAATTTTANCCCAATATCCTTGATGAACATTGATGCAAAAATCCTCAATAAAATACTGGCAAAACAAATCCAGCAGCTCATCAAAAAGCTTGCGGCCGCACTCGAGTAACTAGTTAACCCCTTGGGGCCTCTAAACGGGTCTTGA |
| SimvaEP91 | No Signal |
| SimvaEP92 | No Signal |
| SimvaBN93 | No Signal |
| SimvaBN94 | No Signal |
| SimvaAN95 | No Signal |
| SimvaDN96 | No Signal |
| SimvaDN97 | GTCAATGGTGTGGCCCACTATGGTTTTCTGTGGCATCTCTGTGGATGCTATCATGGCANANAGAGCAGGAAAACTGGCATAACCCTGAGGCAAACTGGGAAGGGGTGCTGTGGGTGCCCTAGACACGAGCAGACTGTTTCTGGNGTTCCTGGAAATTGACATANAGATTAAGAGCCAACTCTCANTGCTGCGGGGCTG |
| SimvaCN98 | No Signal |
| SimvaEN99 | ATCNTTCTNATACCAAAGCTGGGNAGAGACACAACCAAAAAAGAGAATTTTAGCCCAATATCCTTGATGAACATTGATGCAAAAATCCTCAATAAAATACTGGCAAAACAAATCCAGCAGCTCATCAAAAAGCTTGCGGCCGCACTCGAGTAACTAGTTAACCCCTTGGGGCCTCTAAACGGGTCTTGA |
| SimvaEN100 | CCTCTATAAAATACTGGCAAAACAAATCCAGCAGCTCATCAAAAANCTTGCGGCCGCACTCGAGTAACTAGTTAACCCCTTGGGGCCTCTANNCGGGTCTTGAGGGANTAAACGGATTT |
| SimvaCP101 | No Signal |
| SimvaEP102 | No Signal |
| SimvaEP103 | AAGAATTGTCTTGGGCCACCCATAAAATACACTAACACTAATAATAGCTGATGAGCTAAAAAAAAAAAAAAAANCNCAAAAAAAAANCCCCAAAAAAATCCCATAAGGTTTATAAATTTCTATTGGGCCACTTTCAAAGCTGTCTTGGGTCACATGTTGGACAAGCTTGCGGCCNCCCTCNAGTAACTAGTTAACCCCTTGGGGCCTCNAAACGGGNCTTGAGGGGTAAANTGGTTNACNGGGTGGGGGGGCA |
| SimvaEP104 | No Signal |
| SimvaBN105 | No Signal |
| SimvaBN106 | No Signal |
| SimvaAN107 | TTCTNATNCCAAAGCTGGGCAGANACACAACCAAAAAAGAGAATTTTAGCCCAATATCCTTGATGAACATTGATGCAAAAATCCTCAATAAAATACTGGCAAAACAAATCCAGCAGCTCATCAAAAAGCTTGCGGCCGCACTCGAGTAACTAGTTAACCCCTTGGGGCCTCTAAACGGGTCTTGA |
| SimvaDN108 | No Signal |
| SimvaDN109 | No Signal |
| SimvaCN110 | No Signal |
| SimvaEN111 | No Signal |
| SimvaEN112 | No Signal |
| SimvaCP113 | No Signal |
| SimvaEP114 | No Signal |
| SimvaEP115 | GAGGGAATCCTCCCTAACTCATTTTATGAGGCCAGCATCATTCTGATACCAAAGCTGGGCAGAGACACAACCAAAAAAGAGAATTTTAGCCCAATATCCTTGATGAACATTGATGCAAAAATCCTCAATAAAATACTGGCAAAACAAATCCAGCAGCTCATCAAAAAGCTTGCGGCCGCACTCGAGTAACTAGTTAACCCCTTGGGGCCTCTAAACGGGTCTTGA |
| SimvaEP116 | No Signal |
| SimvaBN117 | No Signal |
| SimvaBN118 | No Signal |
| SimvaAN119 | CCTCCCTAANTCATTTTATGAGGCCAGCATCATTCTGATACCAAAGCTGGNCAGAGACACAACCAAAAAAGAGAATTTTAGCCCAATATCCTTGATGAACATTGATGCAAAAATCCTCAATAAAATACTGGCAAAACAAATCCAGCAGCTCATCAAAAAGCTTGCGGCCGCACTCGAGTAACTAGTTAACCCCTTGGGGCCTCTAAACGGGTCTTGAGGGGTTAACTGGNNATNCGNGTGNGGNCGCNNGCNTTTTGATGAGCTGNTGNNNNTGTTTTG |
| SimvaDN120 | GACNNANTAAAGGAAATAGACTCTATTTAAAACATACATAGGCTAGGNGCGATGGCTCATGCCTCCAATCCCAGCNNNTNGNAAGTCCAAGGNNANAGGATCGTTTGAGCCTGGAGCTCAAGACTAGCCCCGGNAACATTGCGAGACCCTGTCTCTACAAAAAAGAAANCNAAAAATTANCCAGCATGTANNGTGCACCTGTANTACTAGCTACTTGGGAGGCTGAAATGGGANGACGGCTTANGCTTGNNNNCNNNNNCNAGNANNTAGNTAACCCCNTGGGGCCTCTANACGGGTCNTGAGG |
| SimvaDN121 | CCTCTTTCTGGCTAGGNGTGGTGACTCATTCTTGTAATCCCAGNNNTNNGNGAGGCCAAGGTGGGCANATCAATTGAGCTCAGGAGTTTGANACCAGCCTANGCNACNTGGCAAAACACCATCTCTNCNAAAAAGNNNNNNAAATTAGCCAGGCATGGNGNCATGTGTCTGTGGNCCNGGCTACTCAAGAGNCNNNNNNGGGAGGATCACCTGANTCNNNGAGGNTGAGGCTGCNNGANCCATGATTGCNCCNCTGCNNTCC |
| SimvaCN122 | No Signal |
| SimvaEN123 | TANGGNNTGTANNANNTGCAGACNTAANGATACNNGAAGCTCANNGATACCCAAGCANATCTAACCTAAAAAGGTCTTTCCCAAGGCATGTTATAGTCAAACTGTCAAAACTCAAAGACAACAAGAGGATTCTAAAAACCATAAGATAAATGCATCAGGTCACATATAAGGACATTGCCATCAAACAAACAACAGATTTCTCAGAAGAAACTACAGGCCANAAGAGCCTGGGATGATGTGTTTAAAATGCTAAAAGTTAAAGAAAAAAAAAAAGCTTGCGGCCGCNCTCNANTAACTAGTTAACCCCTTGGGGCCTCTAAACGGNNCNTGANGNNNTA |
| SimvaEN124 | No Signal |
| SimvaCP125 | No Signal |
| SimvaEP126 | GATGAACCTTGATGCATAAATCCTCAATAAAATACTGGCAAAACAAATCCAGCAGCTCATCAAAAAGCTTGCGGNCGCACTCNAATAACTANTTAACCCCTTGGGGCCTCTAAACGGGNCTTGAGGG |
| SimvaEP127 | No Signal |
| SimvaEP128 | CACCANNTTGGCCAGGCTGNNNTTAAACTCCTGACCTCAGGTGATCCNCCACCTCAGCCTCCCAAAGTGGTGGNNNNNCAGGCGTGAGCCACTGCACCCGGNCTNNAATTTCTTTAAACNNNNNGACTTCTGATATGNNNTNGAANANNGAAANGACTANTCNNANNNGATCGNCANGTATACNTCCAAACCTTTCTNNNNNAAAGCCTANGATTAAGCTNGCNGCCGNNCTCNNNTAACTAGNTAACCCCTNGGGNCNNCNNAACNGNNCT |
| SimvaBN129 | CATNCNGATACCNAAGCTGGGCTGAGACACAACCAAAAAAGAGAATTTTAGCCCAATATCCTTGATGAACATNGATGCAAAAATCCTCAATAAAATACTGGCAAAACAAATCCAGCAGCTCATCAAAAAGCTTGCGGCCGCACTCNAGTAACTAGTNAACCCCTTGGGGCCTCTAA |
| SimvaBN130 | No Signal |
| SimvaAN131 | No Signal |
| SimvaDN132 | GANGAATCCTCCCTAACTCATTTTATGAGGCCAGNATCATTCTGATACCAAAGCTGGGCAGAGACACAACCAAAAAAGAGAATTTTAGCCCAATATCCTTGATGAACATTGATGCAAAAATCCTCAATAAAATACTGGCAAAACAAATCCAGCAGCTCATCAAAAAGCTTGCGGCCGCACTCGAGTAACTAGTTAACCCCTTGGGGCCTCTAAACGGGTCTTGA |
| SimvaDN133 | No Signal |
| SimvaCN134 | No Signal |
| SimvaEN135 | GANGNAATCCTCCCTAACTCATTTTATGAGGCCAGCATCATTCTGATACCAAAGCTGGGCAGAGACACAACCAAAAAAGAGAATTTTANCCCAATATCCTTGATGAACATTGATGCAAAAATCCTCAATAAAATACTGGCAAAACAAATCCAGCAGCTCATCAAAAAGCTTGCGGCCGCACTCGAGTAACTAGTTAACCCCTTGGGGCCTCTAAACGGGTCTTGA |
| SimvaEN136 | CCTGCCNCACATCCTGTTTTGGAGCANAGGACAANANATCANTGTTGTTCACTGTCCTTCANANGGAGCTCCNNNCCACCTTGTCACTCCTAATCCANNGNTCACTGAAGGCAAGCTTGCGGCCGCACTCNAGTAACTAGTTAACCCCTTGGGGCCTCTAAACGGGTCTTGAGGGNNTAANTNGTNNTNNGNGTGNGGNNGCTNGANNGNNNTTAGAGAAANTNGNATTAGGAGNNNCATGGCGGNCTGGAGC |
| SimvaCP137 | No Signal |
| SimvaEP138 | GGCAATAAATTAAAATACACTTCAAGGTAAATTANAAACACTGTTATTTATTTCTTTATTCATTTATTTATTTACTTTAGAGACATGGTCTCACTCTGTTGCCACAATCATAGCTCACTGCAGCCTCAAGCTTGCGGCCGCACTCGAGTAACTAGTTAACCCCTTGGGGCCTCTAAACGGGTCTTGAGGGGTTAACTNGTTACTNGAGTGCGGCCGCNAGCNTGAGGCTGCAGTGNGCTATGATTGTGGCAACAGAGTGAGACCATGTCTCTAAAGTAAATAAATAAATGAATAAAGAAATAAATAACAGTGTTTCTAATTTACCTTGAAGTGTATTTTAATTTATTGCCTTTTTCCCTTTGTCCTCTGAAATTATTATGANNACCACTGCTTTGTGTGCCGNANTGTTTTTTNNGCTTGAATTCGGATCCC |
| SimvaEP139 | AAAAAGAGATTTTAGCCCAATATCCTTGATGAACATTGATGCAAAATCCTCAATAAAATACTGGCAAAACAAATCCAGCAGCTCATCAAAAAGCTTGCTTGCNGTCGCNCTCGANNAACTAGTTACCCCTTGGGGCCTCTATTCGNNTCTTG |
| SimvaEP140 | No Signal |
| SimvaBN141 | No Signal |
| SimvaAN142 | ATGAGCTAAAAAAAAAAAAAAANNCCCAAAAAAAATTCCCCAAAAAAATNNCATAAGGTNNANAAATTTCNATGGGNCCNCATTCAAAGNNGNNTTGGNNNNNNTGTNGNACAANCTNGNGGCCNNNCNCNANNAACAAGTAACCCCCTGGGGNCCTCNAAACGGGNCTTGGGGGGNNAN |
| SimvaAN143 | No Signal |
| SimvaDN144 | No Signal |
| SimvaCN145 | No Signal |
| SimvaCN146 | No Signal |
| SimvaEN147 | ANCTCGNCNACNAGCTTGCGNCCGCACTCNAGTAACTAGTTAACCCCTTGGGGCCTCTAAACGGGTCTTGAGGGGTTAACTGGAN |
| SimvaCP148 | AATCCTANTAAAATACTGGCAAAACAAATCCAGCANCTCATCAAAAAGCTTGCAGCCGCACTCGAGTAACTAGNTAACCCCTTGGGGCCTCTAAACGGGCCTTGAGGNGATAACCANNNNNNCCANNNNTAACTNTNTAANNT |
| SimvaEP149 | No Signal |
| SimvaEP150 | No Signal |
| SimvaEP151 | TGAAACAAAAAAAAGNGNCTGCTTTCCCCTTAAAGAATCATAAAGAAATTTAATCANTTAGACTCAAATACCCTAAAATTTAAAGNTNGCGGCCGCNCTCNATTAACTNGTTAACCCNTGGGGGCCTNNAAANGGGTNTNGAGGGGTTAA |
| SimvaBN152 | No Signal |
| SimvaAN153 | No Signal |
| SimvaAN154 | TGCCCGCGNANAGTGATGGANACNCNNACCAANACAAAAAAANCTGNNNNTNTATTGNNNNNANNGNCNNTCNAAGCTTGCGGCCGCACTCNANTAACTAGTTAACCCCTTGGGGCCTCTNNACGGGTCTTGAGGGGTTAACT |
| SimvaDN155 | No Signal |
| SimvaCN156 | No Signal |
| SimvaCN157 | No Signal |
| SimvaEN158 | No Signal |
| SimvaCP159 | No Signal |
| SimvaEP160 | No Signal |
| SimvaEP161 | No Signal |
| SimvaEP162 | No Signal |
| SimvaBN163 | AAGAATTGNNTTGNNCCACCCATNANATACNCTANCNCTAATAATAGCTGATGAGCTAAAAAAAAAAAAAAATCNCAAAAAAAAATCACCAAAAAAATCTCATAAGGTTTANNAATTTCTNTTGGGCCCCTTTCAAAGNTGNCTTGGGTCACATGTTGAACAAGCTTGCGGGCGCNCNCTANAAACTAGTTAACCCCTTGGGGCCTCTAAACGGGTCTTGGGGGGTTAA |
| SimvaAN164 | AAGAATTGTCTTGGGCCACCCATAAAATACACTAACACTAATAATAGCTGATGAGCTAAAAAAAAAAAAAAAATCNCAAAAAAAAAACCCCAAAAAATCTCATAATGTTTATAAATTTCTATNGGGCCACATTCAAAGNTGTCTTGGGTCACNTGTTGAACAACNTTGCGGCCNCCCTCAAGTAANTAGTTAACCCCTTGGGCCCTCTAAACGGGTCTTGNGGGGTNAACTGGTTACCGGGGGGGGGNGGCAGGCTGGNTCAACTAGGGCCCAAAGANAGNTTNGATT |
| SimvaAN165 | No Signal |
| SimvaDN166 | GGGTTAAACGTCCCNNNNNNNNNNCTCTGACAGCTGTGCACGATGCCATCCTTGANNACNTGNNCTTCCCAAGCGAAATTGTGGGCAAGAGAATCCGCGTCAAANTAGATGGCAGCCGGCTCATAAAGGTTCNTTNGNACAAAGCACAGCANANCAATGTGGAACACAAGGTTGAAACTTTTTCTGGTGTCTATAANAAGCTCACGGGCAAGGATGTTAATTTTGAATTCCCAGAGTTTCAATTGTAAACAAAAATGACTAAATAAAAAGNATATATTCACAGT |
| SimvaCN167 | GNGGGTNTTCTCNGANCTGNTCTAATACTAAATAAAGNTCTTCGTCTTCTTCACTCTTCACTTGTCTGCGTACCTCATTCTTCCCAGATGCANGACGAGAACTCGGACAAAGACGCCATGGAGGTTTCCAGGAAGAAAATCAACACCAATCAACTCCCCAAAGATCCCGAAACAAAGGATCCTAATGTACTTGCTACTCCCGNCCCATCAGGACCAATCANNATAGTGTCAATGGTGTGGCCCACTATGGNTTTCNGTGGNATCTCTGTGGATGCTATCATGGCAGAGAGAGCAGGAAAACTGGCATAACCCTGANGCAAACTGGGAAGGGGTGCTGTGGGTGCCCTAGACACGAGCAGACNTGTTTTCTGGTGTTCCTGGAAATTGACATANAGATTAAGAGCCAACTCTCANNGCTGCGGGGGCTGCAACTGTGNNNNNNAATGAAGAAACTTTGCNGGCC |
| SimvaCN168 | GGTCTTTCCCAAGGCATGTTATANTCAAACTGNCAAAACTCNNANACAACANNAGGATTCTAANNACCATAAGATAAATGCATCANGNNGCATATAANGACATTGCCNTCAAACAAACAACAGATTTCTNNNAGAAACTACNGGCCANAANAGCCTGGNATGATGTGNTTAAAATGCTANAAGTTAAAGAAAAAAAAANNNNTTGCGGCCGCACTCNNNNNNNNGTTAACCCCTTGNNGNCTCTAANCGGGTCTTG |
| SimvaEN169 | No Signal |
| SimvaCP170 | CAAGACCCTGTCTCAAATATAAATAAATACATTTTAAAAAGAAATAAAAAAGGAAACACAAAGCANNCTTGGCTCCATAGAGTTTTCTTTTCTCANTTTTAAGAGTTATTTCTTATGCCTGTAATCCCAGCACTTTGGGAGGCTGANGCNNGAGGATCACTTGAGTTCAGGAATCCAAGGCCAGCCTGGGCAACATGGCGAAACCCTATCTCTACAAAAAATACAAAAAATAAGGCCAGGCATGCTGGCGGGCGCCTGTAATCCTAGCTACTTGGGAGGCTGAGGCAGGAGAATGGCNNGAACTCGGGAGGCGGAGGTTGCAGTGAGCCCAAATTACGCCACTGCACTCCAGCCTTGGGTGACAGAGCGAGACTCTGTCTC |
| SimvaEP171 | GANGNAATCCTCCCTAACTCATTTTATGAGGCCAGCATCATTCTGATACCAAAGCTGGGCAGAGACACAACCAAAAAAGAGAATTTTAGCCCAATATCCTTGATGAACATTGATGCAAAAATCCTCAATAAAATACTGGCAAAACAAATCCAGCAGCTCATCAAAAAGCTTGCGGCCGCACTCGAGTAACTAGTTAACCCCTTGGGGCCTCTAAACGG |
| SimvaEP172 | CATAATAATTTCNNANNACAAAGGGAAAAAGGCAATAAATTAAAATACACTTCAAGGTAAATTANAAACACTGTTATTTATTTCTTTATTCATTTATTTATTTACTTTAGAGACATGGTCTCACTCTGTTGCCACAATCATAGCTCACTGCAGCCTCAAGCTTGCGGCCGCACTCGAGTAACTAGTTAACCCCTTGGGGCCTCTAAACGGGTCTTGAGGGGTTAACTAGTTACNCGAGTGCGGNCGCANGCTTGAGGCTGNNGTGNGCTATGATTGTGGCAACAGAGTGAGACCATGTCTCTAAAGTAAATAAATAAATGAATAAAGAAATAAATAACAGTGTTTCTAATTTACCTTGAAGTGTATTTTAATTTATTGCCTTTTTCCCTTTGTCCTCTGAAATTATTATGACTACCACTGCTTTGTGTGCCGAATGTTTTTTCAGCTTGAATTCGGATCCCCNGAGCNTCACACCTGACTGGAATACGACAGCTNCCA |
| SimvaEP173 | CNGNCAGNTGNGCACGATGNCNTCCTTGAGGACTTGNNCTTCCCAAGCGAAATTGTGGGCAAGAGAATCCGCGTCAAACTAGATGGCAGCCGGCTCATAAAGGTTCATTTGGACAAAGCACANCNNAACAATGTGNAACACAAGGTTGAAACTTTTTCTGGTGTCTATAANAAGCTCACGGGCAAGGATGTTAATTTTGAATTCCCAGAGTTTCAATTGTAAACAAAAATGACTAAATAAAAAGTATATATTCACAGT |
| SimvaBN174 | GANGNAATCCTCCCTAACTCATTTTATGAGGCCNGNATCATTCTGATACCAAAGCTGGGCAGAGACACAACCAAAAAAGAGAATTTTAGCCCAATATCCTTGATGAACATTGATGCAAAAATCCTCAATAAAATACTGGCAAAACAAATCCAGCAGCTCATCAAAAAGCTTGCGGCCGCACTCGAGTAACTAGTTAACCCCTTGGGGCCTCTAAACGGGTCTTGA |
| SimvaAN175 | No Signal |
| SimvaAN176 | ATTAACATTGATNCNAATATCCTCAATTTAATACTGGNNAAACNAATCCANCTGCTCATCAANNCCTTGCGGNCGCATCGAGTAACTNGNTCACCCNTTGGGGCCTCNNAACGGGCCT |
| SimvaDN177 | TCCCTAACTCATTTTATGAGGCCAGNATCATTCTGATACCAAAGCTGGGCAGAGACACAACCAAAAAAGAGAATTTTAGCCCAATATCCTTGATGAACATTGATGCAAAAATCCTCAATAAAATACTGGCAAAACAAATCCAGCAGCTCATCAAAAAGCTTGCGGCCGCACTCGAGTAACTAGTTAACCCCTTGGGGCCTCTAAACGGGTCTTGAGGGGTTAACNGGA |
| SimvaCN178 | GAAATAGACTCTATNTAAAACANACATANGCTAGGNGCGATGGCTCATGCCTCCNATCCCAGNAATTTGNAAGTCCAAGGCNANAGGATCGTTTGAGCCTGAANCTCAAGACTAGCCCCGGCAACATTGCGAGACCCTGTCTCTACAAAAAGAAATCTAAAAATTNNCCNGATGTNGTGTGNACCTGTNGTACTAGCTACTTGGGAGGCTGAAATGGGAGGACGGCTTNAGCTTGCNNCCGCACTCGAGTAANTAGTTAACCCCNTGGGGCCTCTT |
| SimvaCN179 | TCTTGTNAGAGATGCNGACNTCAAGATACAGGAAGCTCAAAGATACCCAAGCAGATCTAACCTAAAAAGGTCTTTCCCAAGGCATGTTATANNCNAACTGTCAAAACTCAAAGACAACAAGAGGATTCTAAAAACCATAAGATAAATGCATCAGGTCACATATAAGGACATTGCCATCAAACAAACAACAGATTTCTCAGAAGAAACTACAGGCCANAAGAGCCTGGGATGATGTGTTTAAAATGCTAAAAGTTAAAGAAAAAAAAAAAGCTTGCGGCCGCACTCGAGTAACTAGTTAACCCCTTGGGGCCTCTAAACGGGTCTTGAGG |
| SimvaEN180 | No Signal |
