# Supplementary material for: Simvastatin Sodium Salt and Fluvastatin Interact with Human Gap Junction Gamma-3 Protein
Source: PLoS One. 2016 Feb 10;11(2):e0148266. doi: 10.1371/journal.pone.0148266 (PMC4749215; doi:10.1371/journal.pone.0148266)
Supplement: S2 Table — (DOCX) [file pone.0148266.s021.docx]

## S2 Simvastatin Biopan *vs.* Human Vascular Tissue Library Sequences Aligned against T7Select^®^ Vector

| Clone ID | Sequence |
| --- | --- |
| SimvaBP1 | CCGGNANTCGTCGANAAGCTTGCGGCCGCACTCNANNNNCTNNNNAA  CCCCTTGGGGCCTCTAAACGGGTCTTGANGGGTTAACTAGTTANTCGA  GTGNGGNNGCNNGCTTGT |
| SimvaBP3 | TCCCGGGANCTCGTCGANAAGCTTGCGGCCGCACTCGANTAACTAGTT  AACCCCTTGGGGCCTCTAAACGGGTCTTGAGGGNNNNNNNNGTTACTN  GAGTGCGG |
| SimvaAP5 | CTTGCGGCCGCACTCNNNNNNNNNNNNNNNNNNNNGGGGCCTCTAAA  CGGGTCTTGAGGG |
| SimvaDP7 | TCCCGGGANCTCGTCGANAAGCTTGCGGCCGCACTCGANNANCTNNT  TAACCCCTTGGGGCCTCTAAACGGGTCTTGAGGGGTTAACTAGTTACTC  GAGTGCGGNCGCNAGCTTGTCGACGAGCT |
| SimvaCP10 | ATCCCGGGANCTCGTCGANAAGCTTGCGGCCGCACTCGANNNNCNAG  TTAACCCCTTGGGGCCTCTAAACGGGTCTTGAGGGGTTAACNAGNNAC  TCGAGTGCGG |
| SimvaAP17 | TCCCGNGANCTCGTCGACAAGCTTGCGGCCGCACTCGNNNNNNNNNN  NNNNNNNNTGGGGCCTCTAAACGGGTCTTGAGGG |
| SimvaDP18 | TCCCGGNNNNCGTCNANAGCTTGCGGCCGCACTCGANNNNNNNNNAA  NNNNTTGGGGCCTCTAAACGGGTCTTGAGGGGT |
| SimvaDP19 | GTCNACAGCTTGCGGCCGCACTCGNNNNNNNNNNNNNNNNNNNNGG  GGCCTCTAAACGGGTCTTGAGG |
| SimvaCP22 | CANGGNNNTCCCGGGANCTCGTCGANAAGCTTGCGGCCGCACTCGAG  TAACTAGTTAACCCCTTGGGGCCTCTAAACGGGTCTTGAGGGGTTAACT  AGTTACTCGAGTGCGGCCGCAAGCNTGTCGACGAGNNCCCGGGATAT  CCCTGCAGGAGAATTCGGATCA |
| SimvaBP25 | CCGGGNGCTCGTCGACAAGCTTGCGGCCGCACTCGANNNNNNNNNNN  NNNNNNNTGGGGCCTCTAAACGGGTCTTGAGGGG |
| SimvaAP26 | TCCCGNGNNNTCGTCGACAAGCTTGCGGCCGCACTCGANNNNNNNNN  NNNANNNNNTGGGGCCTCTAAACGGGTCTTGAGGG |
| SimvaAP28 | ACAGCTTGCGGCCGCCTCNNNNNNNNNNNNNNNNNNTGGGGCCTCTA  AACGGGNCTTGAGG |
| SimvaDP29 | GGNTNTCCCGGGNGCTCGTCGANAGCTTGCGGCCGCACTCGANNNNN  NNNNNNAANCNCTTGGGGCCTCTAAACGGGTCTTGAGGGGTTA |
| SimvaDP51 | ATCCCGGGANCTCGTCGANAAGCTTGCGGCCGCACTCGANTNNCTANT  TAACCCCTTGGGGCCTCTAAACGGGTCTTGAGGGGTTAACTNGTTANTC  GAGTGCGGCCGCAAGC |
| SimvaBP56 | CCGGGAGCTCGTCGANAAGCTTGCGGCCGCACTCGNNNNNNNNNNNA  ACCCCTTGGGGCCTCTAAACGGGTCTTGAGGGGTTA |
| SimvaDP73 | CCCGGGNGCTCGCNACAGCTTGCGGCCGCCTCGANNNNNNNNNANNN  NNNGGGGCCTCTAAACGGGTCTTGAGGG |
| SimvaDP85 | TCCCGNNNNNCGTCGACAGCTTGCGGCCGCCTCNNNNNNNNNNNNNN  NNNTTGGGGCCTCTAAACGGGTCTTGAGGGGN |
| SimvaEN147 | ANCTCGNCNACNAGCTTGCGNCCGCACTCNAGTAACTAGTTAACCCCTT  GGGGCCTCTAAACGGGTCTTGAGGGGTTAACTGGAN |

BLASTN 2.2.24+

Reference: Stephen F. Altschul, Thomas L. Madden, Alejandro

A. Schaffer, Jinghui Zhang, Zheng Zhang, Webb Miller, and

David J. Lipman (1997), "Gapped BLAST and PSI-BLAST: a new

generation of protein database search programs", Nucleic

Acids Res. 25:3389-3402.

RID: EXZ0CZKB11R

Query= Simva1

Length=113

Score E

Sequences producing significant alignments: (Bits) Value

lcl|21049 44.6 4e-10

ALIGNMENTS

>lcl|21049

Length=116

Score = 44.6 bits (48), Expect = 4e-10

Identities = 25/26 (97%), Gaps = 0/26 (0%)

Strand=Plus/Plus

Query 8 TCGTCGANAAGCTTGCGGCCGCACTC 33

||||||| ||||||||||||||||||

Sbjct 77 TCGTCGACAAGCTTGCGGCCGCACTC 102

Score = 41.0 bits (44), Expect = 5e-09

Identities = 28/34 (83%), Gaps = 0/34 (0%)

Strand=Plus/Minus

Query 80 TTAACTAGTTANTCGAGTGNGGNNGCNNGCTTGT 113

||||||||||| ||||||| || || ||||||

Sbjct 116 TTAACTAGTTACTCGAGTGCGGCCGCAAGCTTGT 83

Query= Simva2

Length=277

Score E

Sequences producing significant alignments: (Bits) Value

lcl|21049 64.4 1e-15

ALIGNMENTS

>lcl|21049

Length=116

Score = 64.4 bits (70), Expect = 1e-15

Identities = 40/43 (94%), Gaps = 0/43 (0%)

Strand=Plus/Plus

Query 197 AGCTCATCAAAAAGCTTGCGGCCGCACTCGAGTAACTAGTTAA 239

||||| || | ||||||||||||||||||||||||||||||||

Sbjct 74 AGCTCGTCGACAAGCTTGCGGCCGCACTCGAGTAACTAGTTAA 116

Query= Simva3

Length=104

Score E

Sequences producing significant alignments: (Bits) Value

lcl|21049 80.6 6e-21

ALIGNMENTS

>lcl|21049

Length=116

Score = 80.6 bits (88), Expect = 6e-21

Identities = 47/50 (94%), Gaps = 0/50 (0%)

Strand=Plus/Plus

Query 1 TCCCGGGANCTCGTCGANAAGCTTGCGGCCGCACTCGANTAACTAGTTAA 50

|||||||| |||||||| |||||||||||||||||||| |||||||||||

Sbjct 67 TCCCGGGAGCTCGTCGACAAGCTTGCGGCCGCACTCGAGTAACTAGTTAA 116

Query= Simva5

Length=60

Score E

Sequences producing significant alignments: (Bits) Value

lcl|21049 28.3 2e-05

ALIGNMENTS

>lcl|21049

Length=116

Score = 28.3 bits (30), Expect = 2e-05

Identities = 15/15 (100%), Gaps = 0/15 (0%)

Strand=Plus/Plus

Query 1 CTTGCGGCCGCACTC 15

|||||||||||||||

Sbjct 88 CTTGCGGCCGCACTC 102

Query= Simva6

Length=257

Score E

Sequences producing significant alignments: (Bits) Value

lcl|21049 64.4 1e-15

ALIGNMENTS

>lcl|21049

Length=116

Score = 64.4 bits (70), Expect = 1e-15

Identities = 40/43 (94%), Gaps = 0/43 (0%)

Strand=Plus/Plus

Query 194 AGCTCATCAAAAAGCTTGCGGCCGCACTCGAGTAACTAGTTAA 236

||||| || | ||||||||||||||||||||||||||||||||

Sbjct 74 AGCTCGTCGACAAGCTTGCGGCCGCACTCGAGTAACTAGTTAA 116

Query= Simva7

Length=125

Score E

Sequences producing significant alignments: (Bits) Value

lcl|21049 71.6 3e-18

ALIGNMENTS

>lcl|21049

Length=116

Score = 71.6 bits (78), Expect = 3e-18

Identities = 41/43 (96%), Gaps = 0/43 (0%)

Strand=Plus/Minus

Query 83 TTAACTAGTTACTCGAGTGCGGNCGCNAGCTTGTCGACGAGCT 125

|||||||||||||||||||||| ||| ||||||||||||||||

Sbjct 116 TTAACTAGTTACTCGAGTGCGGCCGCAAGCTTGTCGACGAGCT 74

Score = 66.2 bits (72), Expect = 1e-16

Identities = 43/50 (86%), Gaps = 0/50 (0%)

Strand=Plus/Plus

Query 1 TCCCGGGANCTCGTCGANAAGCTTGCGGCCGCACTCGANNANCTNNTTAA 50

|||||||| |||||||| |||||||||||||||||||| | || ||||

Sbjct 67 TCCCGGGAGCTCGTCGACAAGCTTGCGGCCGCACTCGAGTAACTAGTTAA 116

Query= Simva9

Length=393

Score E

Sequences producing significant alignments: (Bits) Value

lcl|21049 59.0 7e-14

ALIGNMENTS

>lcl|21049

Length=116

Score = 59.0 bits (64), Expect = 7e-14

Identities = 32/32 (100%), Gaps = 0/32 (0%)

Strand=Plus/Plus

Query 308 AAGCTTGCGGCCGCACTCGAGTAACTAGTTAA 339

||||||||||||||||||||||||||||||||

Sbjct 85 AAGCTTGCGGCCGCACTCGAGTAACTAGTTAA 116

Score = 41.0 bits (44), Expect = 2e-08

Identities = 22/22 (100%), Gaps = 0/22 (0%)

Strand=Plus/Minus

Query 372 TTAACTAGTTACTCGAGTGCGG 393

||||||||||||||||||||||

Sbjct 116 TTAACTAGTTACTCGAGTGCGG 95

Query= Simva10

Length=105

Score E

Sequences producing significant alignments: (Bits) Value

lcl|21049 68.0 4e-17

ALIGNMENTS

>lcl|21049

Length=116

Score = 68.0 bits (74), Expect = 4e-17

Identities = 44/51 (87%), Gaps = 0/51 (0%)

Strand=Plus/Plus

Query 1 ATCCCGGGANCTCGTCGANAAGCTTGCGGCCGCACTCGANNNNCNAGTTAA 51

||||||||| |||||||| |||||||||||||||||||| | ||||||

Sbjct 66 ATCCCGGGAGCTCGTCGACAAGCTTGCGGCCGCACTCGAGTAACTAGTTAA 116

Score = 30.1 bits (32), Expect = 9e-06

Identities = 19/22 (87%), Gaps = 0/22 (0%)

Strand=Plus/Minus

Query 84 TTAACNAGNNACTCGAGTGCGG 105

||||| || ||||||||||||

Sbjct 116 TTAACTAGTTACTCGAGTGCGG 95

Query= Simva12

Length=440

Score E

Sequences producing significant alignments: (Bits) Value

lcl|21049 35.6 9e-07

ALIGNMENTS

>lcl|21049

Length=116

Score = 35.6 bits (38), Expect = 9e-07

Identities = 20/21 (96%), Gaps = 0/21 (0%)

Strand=Plus/Plus

Query 420 AAGCTTGCGGCCGCACTCNAG 440

|||||||||||||||||| ||

Sbjct 85 AAGCTTGCGGCCGCACTCGAG 105

Query= Simva13

Length=300

Score E

Sequences producing significant alignments: (Bits) Value

lcl|21049 59.0 5e-14

ALIGNMENTS

>lcl|21049

Length=116

Score = 59.0 bits (64), Expect = 5e-14

Identities = 32/32 (100%), Gaps = 0/32 (0%)

Strand=Plus/Plus

Query 232 AAGCTTGCGGCCGCACTCGAGTAACTAGTTAA 263

||||||||||||||||||||||||||||||||

Sbjct 85 AAGCTTGCGGCCGCACTCGAGTAACTAGTTAA 116

Query= Simva14

Length=205

Score E

Sequences producing significant alignments: (Bits) Value

lcl|21049 60.8 1e-14

ALIGNMENTS

>lcl|21049

Length=116

Score = 60.8 bits (66), Expect = 1e-14

Identities = 33/33 (100%), Gaps = 0/33 (0%)

Strand=Plus/Plus

Query 131 CAAGCTTGCGGCCGCACTCGAGTAACTAGTTAA 163

|||||||||||||||||||||||||||||||||

Sbjct 84 CAAGCTTGCGGCCGCACTCGAGTAACTAGTTAA 116

Query= Simva17

Length=81

Score E

Sequences producing significant alignments: (Bits) Value

lcl|21049 60.8 4e-15

ALIGNMENTS

>lcl|21049

Length=116

Score = 60.8 bits (66), Expect = 4e-15

Identities = 35/37 (95%), Gaps = 0/37 (0%)

Strand=Plus/Plus

Query 1 TCCCGNGANCTCGTCGACAAGCTTGCGGCCGCACTCG 37

||||| || ||||||||||||||||||||||||||||

Sbjct 67 TCCCGGGAGCTCGTCGACAAGCTTGCGGCCGCACTCG 103

Query= Simva18

Length=80

Score E

Sequences producing significant alignments: (Bits) Value

lcl|21049 35.6 2e-07

ALIGNMENTS

>lcl|21049

Length=116

Score = 35.6 bits (38), Expect = 2e-07

Identities = 19/19 (100%), Gaps = 0/19 (0%)

Strand=Plus/Plus

Query 18 AGCTTGCGGCCGCACTCGa 36

|||||||||||||||||||

Sbjct 86 AGCTTGCGGCCGCACTCGA 104

Query= Simva19

Length=68

Score E

Sequences producing significant alignments: (Bits) Value

lcl|21049 33.7 4e-07

ALIGNMENTS

>lcl|21049

Length=116

Score = 33.7 bits (36), Expect = 4e-07

Identities = 23/25 (92%), Gaps = 1/25 (4%)

Strand=Plus/Plus

Query 1 GTCNACA-GCTTGCGGCCGCACTCG 24

||| ||| |||||||||||||||||

Sbjct 79 GTCGACAAGCTTGCGGCCGCACTCG 103

Query= Simva22

Length=165

Score E

Sequences producing significant alignments: (Bits) Value

lcl|21049 123 8e-34

ALIGNMENTS

>lcl|21049

Length=116

Score = 123 bits (136), Expect = 8e-34

Identities = 71/74 (96%), Gaps = 0/74 (0%)

Strand=Plus/Minus

Query 91 TTAACTAGTTACTCGAGTGCGGCCGCAAGCNTGTCGACGAGNNCCCGGGATATCCCTGCA 150

|||||||||||||||||||||||||||||| |||||||||| |||||||||||||||||

Sbjct 116 TTAACTAGTTACTCGAGTGCGGCCGCAAGCTTGTCGACGAGCTCCCGGGATATCCCTGCA 57

Query 151 GGAGAATTCGGATC 164

||||||||||||||

Sbjct 56 GGAGAATTCGGATC 43

Score = 84.2 bits (92), Expect = 7e-22

Identities = 48/50 (96%), Gaps = 0/50 (0%)

Strand=Plus/Plus

Query 9 TCCCGGGANCTCGTCGANAAGCTTGCGGCCGCACTCGAGTAACTAGTTAA 58

|||||||| |||||||| ||||||||||||||||||||||||||||||||

Sbjct 67 TCCCGGGAGCTCGTCGACAAGCTTGCGGCCGCACTCGAGTAACTAGTTAA 116

Query= Simva25

Length=81

Score E

Sequences producing significant alignments: (Bits) Value

lcl|21049 62.6 1e-15

ALIGNMENTS

>lcl|21049

Length=116

Score = 62.6 bits (68), Expect = 1e-15

Identities = 35/36 (98%), Gaps = 0/36 (0%)

Strand=Plus/Plus

Query 1 CCGGGNGCTCGTCGACAAGCTTGCGGCCGCACTCGa 36

||||| ||||||||||||||||||||||||||||||

Sbjct 69 CCGGGAGCTCGTCGACAAGCTTGCGGCCGCACTCGA 104

Query= Simva26

Length=82

Score E

Sequences producing significant alignments: (Bits) Value

lcl|21049 55.4 2e-13

ALIGNMENTS

>lcl|21049

Length=116

Score = 55.4 bits (60), Expect = 2e-13

Identities = 34/38 (90%), Gaps = 0/38 (0%)

Strand=Plus/Plus

Query 1 TCCCGNGNNNTCGTCGACAAGCTTGCGGCCGCACTCGa 38

||||| | ||||||||||||||||||||||||||||

Sbjct 67 TCCCGGGAGCTCGTCGACAAGCTTGCGGCCGCACTCGA 104

Query= Simva28

Length=61

Score E

Sequences producing significant alignments: (Bits) Value

lcl|21049 24.7 2e-04

ALIGNMENTS

>lcl|21049

Length=116

Score = 24.7 bits (26), Expect = 2e-04

Identities = 13/13 (100%), Gaps = 0/13 (0%)

Strand=Plus/Plus

Query 3 AGCTTGCGGCCGC 15

|||||||||||||

Sbjct 86 AGCTTGCGGCCGC 98

Query= Simva29

Length=90

Score E

Sequences producing significant alignments: (Bits) Value

lcl|21049 55.4 2e-13

ALIGNMENTS

>lcl|21049

Length=116

Score = 55.4 bits (60), Expect = 2e-13

Identities = 38/43 (89%), Gaps = 1/43 (2%)

Strand=Plus/Plus

Query 1 GGNTNTCCCGGGNGCTCGTCGANA-GCTTGCGGCCGCACTCGa 42

|| | ||||||| ||||||||| | ||||||||||||||||||

Sbjct 62 GGATATCCCGGGAGCTCGTCGACAAGCTTGCGGCCGCACTCGA 104

Query= Simva40

Length=236

Score E

Sequences producing significant alignments: (Bits) Value

lcl|21049 64.4 1e-15

ALIGNMENTS

>lcl|21049

Length=116

Score = 64.4 bits (70), Expect = 1e-15

Identities = 40/43 (94%), Gaps = 0/43 (0%)

Strand=Plus/Plus

Query 158 AGCTCATCAAAAAGCTTGCGGCCGCACTCGAGTAACTAGTTAA 200

||||| || | ||||||||||||||||||||||||||||||||

Sbjct 74 AGCTCGTCGACAAGCTTGCGGCCGCACTCGAGTAACTAGTTAA 116

Query= Simva45

Length=235

Score E

Sequences producing significant alignments: (Bits) Value

lcl|21049 64.4 1e-15

ALIGNMENTS

>lcl|21049

Length=116

Score = 64.4 bits (70), Expect = 1e-15

Identities = 40/43 (94%), Gaps = 0/43 (0%)

Strand=Plus/Plus

Query 157 AGCTCATCAAAAAGCTTGCGGCCGCACTCGAGTAACTAGTTAA 199

||||| || | ||||||||||||||||||||||||||||||||

Sbjct 74 AGCTCGTCGACAAGCTTGCGGCCGCACTCGAGTAACTAGTTAA 116

Query= Simva47

Length=282

Score E

Sequences producing significant alignments: (Bits) Value

lcl|21049 64.4 1e-15

ALIGNMENTS

>lcl|21049

Length=116

Score = 64.4 bits (70), Expect = 1e-15

Identities = 40/43 (94%), Gaps = 0/43 (0%)

Strand=Plus/Plus

Query 200 AGCTCATCAAAAAGCTTGCGGCCGCACTCGAGTAACTAGTTAA 242

||||| || | ||||||||||||||||||||||||||||||||

Sbjct 74 AGCTCGTCGACAAGCTTGCGGCCGCACTCGAGTAACTAGTTAA 116

Query= Simva51

Length=113

Score E

Sequences producing significant alignments: (Bits) Value

lcl|21049 71.6 3e-18

ALIGNMENTS

>lcl|21049

Length=116

Score = 71.6 bits (78), Expect = 3e-18

Identities = 45/51 (89%), Gaps = 0/51 (0%)

Strand=Plus/Plus

Query 1 ATCCCGGGANCTCGTCGANAAGCTTGCGGCCGCACTCGANTNNCTANTTAA 51

||||||||| |||||||| |||||||||||||||||||| | ||| ||||

Sbjct 66 ATCCCGGGAGCTCGTCGACAAGCTTGCGGCCGCACTCGAGTAACTAGTTAA 116

Score = 48.2 bits (52), Expect = 4e-11

Identities = 28/30 (94%), Gaps = 0/30 (0%)

Strand=Plus/Minus

Query 84 TTAACTNGTTANTCGAGTGCGGCCGCAAGC 113

|||||| |||| ||||||||||||||||||

Sbjct 116 TTAACTAGTTACTCGAGTGCGGCCGCAAGC 87

Query= Simva53

Length=234

Score E

Sequences producing significant alignments: (Bits) Value

lcl|21049 64.4 1e-15

ALIGNMENTS

>lcl|21049

Length=116

Score = 64.4 bits (70), Expect = 1e-15

Identities = 40/43 (94%), Gaps = 0/43 (0%)

Strand=Plus/Plus

Query 156 AGCTCATCAAAAAGCTTGCGGCCGCACTCGAGTAACTAGTTAA 198

||||| || | ||||||||||||||||||||||||||||||||

Sbjct 74 AGCTCGTCGACAAGCTTGCGGCCGCACTCGAGTAACTAGTTAA 116

Query= Simva55

Length=593

Score E

Sequences producing significant alignments: (Bits) Value

lcl|21049 59.0 1e-13

ALIGNMENTS

>lcl|21049

Length=116

Score = 59.0 bits (64), Expect = 1e-13

Identities = 32/32 (100%), Gaps = 0/32 (0%)

Strand=Plus/Plus

Query 538 AAGCTTGCGGCCGCACTCGAGTAACTAGTTAA 569

||||||||||||||||||||||||||||||||

Sbjct 85 AAGCTTGCGGCCGCACTCGAGTAACTAGTTAA 116

Query= Simva56

Length=83

Score E

Sequences producing significant alignments: (Bits) Value

lcl|21049 60.8 4e-15

ALIGNMENTS

>lcl|21049

Length=116

Score = 60.8 bits (66), Expect = 4e-15

Identities = 34/35 (98%), Gaps = 0/35 (0%)

Strand=Plus/Plus

Query 1 CCGGGAGCTCGTCGANAAGCTTGCGGCCGCACTCG 35

||||||||||||||| |||||||||||||||||||

Sbjct 69 CCGGGAGCTCGTCGACAAGCTTGCGGCCGCACTCG 103

Query= Simva57

Length=355

Score E

Sequences producing significant alignments: (Bits) Value

lcl|21049 59.0 6e-14

ALIGNMENTS

>lcl|21049

Length=116

Score = 59.0 bits (64), Expect = 6e-14

Identities = 32/32 (100%), Gaps = 0/32 (0%)

Strand=Plus/Plus

Query 282 AAGCTTGCGGCCGCACTCGAGTAACTAGTTAA 313

||||||||||||||||||||||||||||||||

Sbjct 85 AAGCTTGCGGCCGCACTCGAGTAACTAGTTAA 116

Query= Simva62

Length=206

Score E

Sequences producing significant alignments: (Bits) Value

lcl|21049 60.8 1e-14

ALIGNMENTS

>lcl|21049

Length=116

Score = 60.8 bits (66), Expect = 1e-14

Identities = 33/33 (100%), Gaps = 0/33 (0%)

Strand=Plus/Plus

Query 134 CAAGCTTGCGGCCGCACTCGAGTAACTAGTTAA 166

|||||||||||||||||||||||||||||||||

Sbjct 84 CAAGCTTGCGGCCGCACTCGAGTAACTAGTTAA 116

Query= Simva64

Length=246

Score E

Sequences producing significant alignments: (Bits) Value

lcl|21049 64.4 1e-15

ALIGNMENTS

>lcl|21049

Length=116

Score = 64.4 bits (70), Expect = 1e-15

Identities = 40/43 (94%), Gaps = 0/43 (0%)

Strand=Plus/Plus

Query 160 AGCTCATCAAAAAGCTTGCGGCCGCACTCGAGTAACTAGTTAA 202

||||| || | ||||||||||||||||||||||||||||||||

Sbjct 74 AGCTCGTCGACAAGCTTGCGGCCGCACTCGAGTAACTAGTTAA 116

Score = 22.9 bits (24), Expect = 0.003

Identities = 12/12 (100%), Gaps = 0/12 (0%)

Strand=Plus/Minus

Query 235 TTAACTAGTTAC 246

||||||||||||

Sbjct 116 TTAACTAGTTAC 105

Query= Simva67

Length=61

Score E

Sequences producing significant alignments: (Bits) Value

lcl|21049 26.5 6e-05

ALIGNMENTS

>lcl|21049

Length=116

Score = 26.5 bits (28), Expect = 6e-05

Identities = 14/14 (100%), Gaps = 0/14 (0%)

Strand=Plus/Plus

Query 1 TTGCGGCCGCACTC 14

||||||||||||||

Sbjct 89 TTGCGGCCGCACTC 102

Query= Simva69

Length=71

Score E

Sequences producing significant alignments: (Bits) Value

lcl|21049 35.6 1e-07

ALIGNMENTS

>lcl|21049

Length=116

Score = 35.6 bits (38), Expect = 1e-07

Identities = 20/21 (96%), Gaps = 0/21 (0%)

Strand=Plus/Plus

Query 1 CGNCGACAAGCTTGCGGCCGC 21

|| ||||||||||||||||||

Sbjct 78 CGTCGACAAGCTTGCGGCCGC 98

Query= Simva72

Length=248

Score E

Sequences producing significant alignments: (Bits) Value

lcl|21049 60.8 1e-14

ALIGNMENTS

>lcl|21049

Length=116

Score = 60.8 bits (66), Expect = 1e-14

Identities = 33/33 (100%), Gaps = 0/33 (0%)

Strand=Plus/Plus

Query 180 CAAGCTTGCGGCCGCACTCGAGTAACTAGTTAA 212

|||||||||||||||||||||||||||||||||

Sbjct 84 CAAGCTTGCGGCCGCACTCGAGTAACTAGTTAA 116

Query= Simva73

Length=75

Score E

Sequences producing significant alignments: (Bits) Value

lcl|21049 35.6 1e-07

ALIGNMENTS

>lcl|21049

Length=116

Score = 35.6 bits (38), Expect = 1e-07

Identities = 32/37 (87%), Gaps = 3/37 (8%)

Strand=Plus/Plus

Query 1 CCCGGGNGCTCG-CNACA-GCTTGCGGCCGC-CTCGa 34

|||||| ||||| | ||| |||||||||||| |||||

Sbjct 68 CCCGGGAGCTCGTCGACAAGCTTGCGGCCGCACTCGA 104

Query= Simva76

Length=192

Score E

Sequences producing significant alignments: (Bits) Value

lcl|21049 60.8 1e-14

ALIGNMENTS

>lcl|21049

Length=116

Score = 60.8 bits (66), Expect = 1e-14

Identities = 39/43 (91%), Gaps = 0/43 (0%)

Strand=Plus/Plus

Query 115 AGCTCATCAAAAAGCTTGCGGCCGCACTCGAGTANCTAGTTAA 157

||||| || | ||||||||||||||||||||||| ||||||||

Sbjct 74 AGCTCGTCGACAAGCTTGCGGCCGCACTCGAGTAACTAGTTAA 116

Query= Simva85

Length=79

Score E

Sequences producing significant alignments: (Bits) Value

lcl|21049 30.1 6e-06

ALIGNMENTS

>lcl|21049

Length=116

Score = 30.1 bits (32), Expect = 6e-06

Identities = 20/21 (96%), Gaps = 1/21 (4%)

Strand=Plus/Plus

Query 11 CGTCGACA-GCTTGCGGCCGC 30

|||||||| ||||||||||||

Sbjct 78 CGTCGACAAGCTTGCGGCCGC 98

Query= Simva90

Length=218

Score E

Sequences producing significant alignments: (Bits) Value

lcl|21049 64.4 9e-16

ALIGNMENTS

>lcl|21049

Length=116

Score = 64.4 bits (70), Expect = 9e-16

Identities = 40/43 (94%), Gaps = 0/43 (0%)

Strand=Plus/Plus

Query 148 AGCTCATCAAAAAGCTTGCGGCCGCACTCGAGTAACTAGTTAA 190

||||| || | ||||||||||||||||||||||||||||||||

Sbjct 74 AGCTCGTCGACAAGCTTGCGGCCGCACTCGAGTAACTAGTTAA 116

Query= Simva99

Length=189

Score E

Sequences producing significant alignments: (Bits) Value

lcl|21049 64.4 8e-16

ALIGNMENTS

>lcl|21049

Length=116

Score = 64.4 bits (70), Expect = 8e-16

Identities = 40/43 (94%), Gaps = 0/43 (0%)

Strand=Plus/Plus

Query 119 AGCTCATCAAAAAGCTTGCGGCCGCACTCGAGTAACTAGTTAA 161

||||| || | ||||||||||||||||||||||||||||||||

Sbjct 74 AGCTCGTCGACAAGCTTGCGGCCGCACTCGAGTAACTAGTTAA 116

Query= Simva100

Length=119

Score E

Sequences producing significant alignments: (Bits) Value

lcl|21049 60.8 6e-15

ALIGNMENTS

>lcl|21049

Length=116

Score = 60.8 bits (66), Expect = 6e-15

Identities = 39/43 (91%), Gaps = 0/43 (0%)

Strand=Plus/Plus

Query 33 AGCTCATCAAAAANCTTGCGGCCGCACTCGAGTAACTAGTTAA 75

||||| || | || |||||||||||||||||||||||||||||

Sbjct 74 AGCTCGTCGACAAGCTTGCGGCCGCACTCGAGTAACTAGTTAA 116

Query= Simva103

Length=253

Score E

Sequences producing significant alignments: (Bits) Value

lcl|21049 51.8 7e-12

ALIGNMENTS

>lcl|21049

Length=116

Score = 51.8 bits (56), Expect = 7e-12

Identities = 32/35 (92%), Gaps = 0/35 (0%)

Strand=Plus/Plus

Query 160 GACAAGCTTGCGGCCNCCCTCNAGTAACTAGTTAA 194

||||||||||||||| | ||| |||||||||||||

Sbjct 82 GACAAGCTTGCGGCCGCACTCGAGTAACTAGTTAA 116

Query= Simva107

Length=185

Score E

Sequences producing significant alignments: (Bits) Value

lcl|21049 64.4 8e-16

ALIGNMENTS

>lcl|21049

Length=116

Score = 64.4 bits (70), Expect = 8e-16

Identities = 40/43 (94%), Gaps = 0/43 (0%)

Strand=Plus/Plus

Query 115 AGCTCATCAAAAAGCTTGCGGCCGCACTCGAGTAACTAGTTAA 157

||||| || | ||||||||||||||||||||||||||||||||

Sbjct 74 AGCTCGTCGACAAGCTTGCGGCCGCACTCGAGTAACTAGTTAA 116

Query= Simva115

Length=225

Score E

Sequences producing significant alignments: (Bits) Value

lcl|21049 64.4 9e-16

ALIGNMENTS

>lcl|21049

Length=116

Score = 64.4 bits (70), Expect = 9e-16

Identities = 40/43 (94%), Gaps = 0/43 (0%)

Strand=Plus/Plus

Query 155 AGCTCATCAAAAAGCTTGCGGCCGCACTCGAGTAACTAGTTAA 197

||||| || | ||||||||||||||||||||||||||||||||

Sbjct 74 AGCTCGTCGACAAGCTTGCGGCCGCACTCGAGTAACTAGTTAA 116

Query= Simva119

Length=279

Score E

Sequences producing significant alignments: (Bits) Value

lcl|21049 64.4 1e-15

ALIGNMENTS

>lcl|21049

Length=116

Score = 64.4 bits (70), Expect = 1e-15

Identities = 40/43 (94%), Gaps = 0/43 (0%)

Strand=Plus/Plus

Query 147 AGCTCATCAAAAAGCTTGCGGCCGCACTCGAGTAACTAGTTAA 189

||||| || | ||||||||||||||||||||||||||||||||

Sbjct 74 AGCTCGTCGACAAGCTTGCGGCCGCACTCGAGTAACTAGTTAA 116

Query= Simva123

Length=338

Score E

Sequences producing significant alignments: (Bits) Value

lcl|21049 48.2 1e-10

ALIGNMENTS

>lcl|21049

Length=116

Score = 48.2 bits (52), Expect = 1e-10

Identities = 29/32 (91%), Gaps = 0/32 (0%)

Strand=Plus/Plus

Query 272 aaGCTTGCGGCCGCNCTCNANTAACTAGTTAA 303

|||||||||||||| ||| | |||||||||||

Sbjct 85 AAGCTTGCGGCCGCACTCGAGTAACTAGTTAA 116

Query= Simva129

Length=176

Score E

Sequences producing significant alignments: (Bits) Value

lcl|21049 57.2 1e-13

ALIGNMENTS

>lcl|21049

Length=116

Score = 57.2 bits (62), Expect = 1e-13

Identities = 38/43 (89%), Gaps = 0/43 (0%)

Strand=Plus/Plus

Query 117 AGCTCATCAAAAAGCTTGCGGCCGCACTCNAGTAACTAGTNAA 159

||||| || | |||||||||||||||||| |||||||||| ||

Sbjct 74 AGCTCGTCGACAAGCTTGCGGCCGCACTCGAGTAACTAGTTAA 116

Query= Simva132

Length=224

Score E

Sequences producing significant alignments: (Bits) Value

lcl|21049 64.4 9e-16

ALIGNMENTS

>lcl|21049

Length=116

Score = 64.4 bits (70), Expect = 9e-16

Identities = 40/43 (94%), Gaps = 0/43 (0%)

Strand=Plus/Plus

Query 154 AGCTCATCAAAAAGCTTGCGGCCGCACTCGAGTAACTAGTTAA 196

||||| || | ||||||||||||||||||||||||||||||||

Sbjct 74 AGCTCGTCGACAAGCTTGCGGCCGCACTCGAGTAACTAGTTAA 116

Query= Simva135

Length=225

Score E

Sequences producing significant alignments: (Bits) Value

lcl|21049 64.4 9e-16

ALIGNMENTS

>lcl|21049

Length=116

Score = 64.4 bits (70), Expect = 9e-16

Identities = 40/43 (94%), Gaps = 0/43 (0%)

Strand=Plus/Plus

Query 155 AGCTCATCAAAAAGCTTGCGGCCGCACTCGAGTAACTAGTTAA 197

||||| || | ||||||||||||||||||||||||||||||||

Sbjct 74 AGCTCGTCGACAAGCTTGCGGCCGCACTCGAGTAACTAGTTAA 116

Query= Simva136

Length=253

Score E

Sequences producing significant alignments: (Bits) Value

lcl|21049 57.2 2e-13

ALIGNMENTS

>lcl|21049

Length=116

Score = 57.2 bits (62), Expect = 2e-13

Identities = 32/33 (97%), Gaps = 0/33 (0%)

Strand=Plus/Plus

Query 112 CAAGCTTGCGGCCGCACTCNAGTAACTAGTTAA 144

||||||||||||||||||| |||||||||||||

Sbjct 84 CAAGCTTGCGGCCGCACTCGAGTAACTAGTTAA 116

Query= Simva138

Length=432

Score E

Sequences producing significant alignments: (Bits) Value

lcl|21049 60.8 2e-14

ALIGNMENTS

>lcl|21049

Length=116

Score = 60.8 bits (66), Expect = 2e-14

Identities = 33/33 (100%), Gaps = 0/33 (0%)

Strand=Plus/Plus

Query 127 CAAGCTTGCGGCCGCACTCGAGTAACTAGTTAA 159

|||||||||||||||||||||||||||||||||

Sbjct 84 CAAGCTTGCGGCCGCACTCGAGTAACTAGTTAA 116

Score = 46.4 bits (50), Expect = 5e-10

Identities = 29/33 (88%), Gaps = 0/33 (0%)

Strand=Plus/Minus

Query 192 TTAACTNGTTACTNGAGTGCGGCCGCNAGCNTG 224

|||||| |||||| |||||||||||| ||| ||

Sbjct 116 TTAACTAGTTACTCGAGTGCGGCCGCAAGCTTG 84

Score = 24.7 bits (26), Expect = 0.002

Identities = 13/13 (100%), Gaps = 0/13 (0%)

Strand=Plus/Minus

Query 420 GAATTCGGATCCC 432

|||||||||||||

Sbjct 53 GAATTCGGATCCC 41

Query= Simva147

Length=85

Score E

Sequences producing significant alignments: (Bits) Value

lcl|21049 57.2 5e-14

ALIGNMENTS

>lcl|21049

Length=116

Score = 57.2 bits (62), Expect = 5e-14

Identities = 36/41 (88%), Gaps = 0/41 (0%)

Strand=Plus/Plus

Query 3 CTCGNCNACNAGCTTGCGNCCGCACTCNAGTAACTAGTTAA 43

|||| | || |||||||| |||||||| |||||||||||||

Sbjct 76 CTCGTCGACAAGCTTGCGGCCGCACTCGAGTAACTAGTTAA 116

Query= Simva148

Length=143

Score E

Sequences producing significant alignments: (Bits) Value

lcl|21049 53.6 1e-12

ALIGNMENTS

>lcl|21049

Length=116

Score = 53.6 bits (58), Expect = 1e-12

Identities = 36/41 (88%), Gaps = 0/41 (0%)

Strand=Plus/Plus

Query 37 CTCATCAAAAAGCTTGCAGCCGCACTCGAGTAACTAGNTAA 77

||| || | |||||||| ||||||||||||||||||| |||

Sbjct 76 CTCGTCGACAAGCTTGCGGCCGCACTCGAGTAACTAGTTAA 116

Query= Simva154

Length=143

Score E

Sequences producing significant alignments: (Bits) Value

lcl|21049 51.8 4e-12

ALIGNMENTS

>lcl|21049

Length=116

Score = 51.8 bits (56), Expect = 4e-12

Identities = 30/32 (94%), Gaps = 0/32 (0%)

Strand=Plus/Plus

Query 74 AAGCTTGCGGCCGCACTCNANTAACTAGTTAA 105

|||||||||||||||||| | |||||||||||

Sbjct 85 AAGCTTGCGGCCGCACTCGAGTAACTAGTTAA 116

Query= Simva163

Length=229

Score E

Sequences producing significant alignments: (Bits) Value

lcl|21049 37.4 1e-07

ALIGNMENTS

>lcl|21049

Length=116

Score = 37.4 bits (40), Expect = 1e-07

Identities = 28/34 (83%), Gaps = 0/34 (0%)

Strand=Plus/Plus

Query 160 ACAAGCTTGCGGGCGCNCNCTANAAACTAGTTAA 193

|||||||||||| ||| | | | ||||||||||

Sbjct 83 ACAAGCTTGCGGCCGCACTCGAGTAACTAGTTAA 116

Query= Simva168

Length=256

Score E

Sequences producing significant alignments: (Bits) Value

lcl|21049 26.5 3e-04

ALIGNMENTS

>lcl|21049

Length=116

Score = 26.5 bits (28), Expect = 3e-04

Identities = 14/14 (100%), Gaps = 0/14 (0%)

Strand=Plus/Plus

Query 203 TTGCGGCCGCACTC 216

||||||||||||||

Sbjct 89 TTGCGGCCGCACTC 102

Query= Simva171

Length=218

Score E

Sequences producing significant alignments: (Bits) Value

lcl|21049 64.4 9e-16

ALIGNMENTS

>lcl|21049

Length=116

Score = 64.4 bits (70), Expect = 9e-16

Identities = 40/43 (94%), Gaps = 0/43 (0%)

Strand=Plus/Plus

Query 155 AGCTCATCAAAAAGCTTGCGGCCGCACTCGAGTAACTAGTTAA 197

||||| || | ||||||||||||||||||||||||||||||||

Sbjct 74 AGCTCGTCGACAAGCTTGCGGCCGCACTCGAGTAACTAGTTAA 116

Query= Simva172

Length=498

Score E

Sequences producing significant alignments: (Bits) Value

lcl|21049 71.6 1e-17

ALIGNMENTS

>lcl|21049

Length=116

Score = 71.6 bits (78), Expect = 1e-17

Identities = 44/46 (96%), Gaps = 1/46 (2%)

Strand=Plus/Minus

Query 449 GAATTCGGATCCCCNGAGCNTCACACCTGACTGGAATACGACAGCT 494

|||||||||||||| |||| ||||||||||||||||||||||||||

Sbjct 53 GAATTCGGATCCCC-GAGCATCACACCTGACTGGAATACGACAGCT 9

Score = 60.8 bits (66), Expect = 3e-14

Identities = 33/33 (100%), Gaps = 0/33 (0%)

Strand=Plus/Plus

Query 157 CAAGCTTGCGGCCGCACTCGAGTAACTAGTTAA 189

|||||||||||||||||||||||||||||||||

Sbjct 84 CAAGCTTGCGGCCGCACTCGAGTAACTAGTTAA 116

Score = 50.0 bits (54), Expect = 5e-11

Identities = 30/33 (91%), Gaps = 0/33 (0%)

Strand=Plus/Minus

Query 222 TTAACTAGTTACNCGAGTGCGGNCGCANGCTTG 254

|||||||||||| ||||||||| |||| |||||

Sbjct 116 TTAACTAGTTACTCGAGTGCGGCCGCAAGCTTG 84

Query= Simva174

Length=225

Score E

Sequences producing significant alignments: (Bits) Value

lcl|21049 64.4 9e-16

ALIGNMENTS

>lcl|21049

Length=116

Score = 64.4 bits (70), Expect = 9e-16

Identities = 40/43 (94%), Gaps = 0/43 (0%)

Strand=Plus/Plus

Query 155 AGCTCATCAAAAAGCTTGCGGCCGCACTCGAGTAACTAGTTAA 197

||||| || | ||||||||||||||||||||||||||||||||

Sbjct 74 AGCTCGTCGACAAGCTTGCGGCCGCACTCGAGTAACTAGTTAA 116

Query= Simva177

Length=228

Score E

Sequences producing significant alignments: (Bits) Value

lcl|21049 64.4 1e-15

ALIGNMENTS

>lcl|21049

Length=116

Score = 64.4 bits (70), Expect = 1e-15

Identities = 40/43 (94%), Gaps = 0/43 (0%)

Strand=Plus/Plus

Query 145 AGCTCATCAAAAAGCTTGCGGCCGCACTCGAGTAACTAGTTAA 187

||||| || | ||||||||||||||||||||||||||||||||

Sbjct 74 AGCTCGTCGACAAGCTTGCGGCCGCACTCGAGTAACTAGTTAA 116

Query= Simva178

Length=276

Score E

Sequences producing significant alignments: (Bits) Value

lcl|21049 46.4 3e-10

ALIGNMENTS

>lcl|21049

Length=116

Score = 46.4 bits (50), Expect = 3e-10

Identities = 28/31 (91%), Gaps = 0/31 (0%)

Strand=Plus/Plus

Query 230 AGCTTGCNNCCGCACTCGAGTAANTAGTTAA 260

||||||| |||||||||||||| |||||||

Sbjct 86 AGCTTGCGGCCGCACTCGAGTAACTAGTTAA 116

Query= Simva179

Length=329

Score E

Sequences producing significant alignments: (Bits) Value

lcl|21049 59.0 6e-14

ALIGNMENTS

>lcl|21049

Length=116

Score = 59.0 bits (64), Expect = 6e-14

Identities = 32/32 (100%), Gaps = 0/32 (0%)

Strand=Plus/Plus

Query 268 aaGCTTGCGGCCGCACTCGAGTAACTAGTTAA 299

||||||||||||||||||||||||||||||||

Sbjct 85 AAGCTTGCGGCCGCACTCGAGTAACTAGTTAA 116
