# Supplementary material for: Simvastatin Sodium Salt and Fluvastatin Interact with Human Gap Junction Gamma-3 Protein
Source: PLoS One. 2016 Feb 10;11(2):e0148266. doi: 10.1371/journal.pone.0148266 (PMC4749215; doi:10.1371/journal.pone.0148266)
Supplement: S3 Table — (DOCX) [file pone.0148266.s022.docx]

## S3 Lasergene^®^ SeqMan Analysis of Simvastatin Biopan *vs.* Human Vascular Tissue Library Sequences

| >Simvastatin Contig 1 |
| --- |
| CCTGGGGTGCTGGAACCTCTTCCTTATGTCATAATAGGTATTAAAAAAAAAGACCTCTGGACTGAAAAAAATCAAGAGAAGATTTACAATGCATGGAAATAACTGTAATTTATCATATTAATTTGGGCTACCAGTAGCATCGTTTAGAGATTGTGCAAATTGAGTTCACAAATATCACAGCTTTGGAAGATCAAGAAGTGGTTTTGAACATAGAGATTCCAGGGAAAAAAAGTAATAATAAGATTAAAAAAAAAAAAAAAAAAAAAAAAAAAAAAAAAAAAAAAAAAAAAAAAAAAAAAAAAAAA |
| >Simvastatin Contig 2 |
| ACAAAGGGAAAAAGGCAATAAATTAAAATACACTTCAAGGTAAATTAGAAACACTGTTATTTATTTCTTTATTCATTTATTTATTTACTTTAGAGACATGGTCTCACTCTGTTGCCACAATCATAGCTCACTGCAGCCTCAAGCTTGCGGCCGCACTCGAGTAACTAGTTAACCCCTTGGGGCCTCTAAACGGGTCTTGAGGGGTTAACTAGTTACTCGAGTGCGGCCGCAAGCTTGAGGCTGCAGTGAGCTATGATTGTGGCAACAGAGTGAGACCATGTCTCTAAAGTAAATAAATAAATGAATAAAGAAATAAATAACAGTGTTTCTAATTTACCTTGAAGTGTATTTTAATTTATTGCCTTTTTCCCTTTGTCCTCTGAAATTATTATGACTACCACTGCTTTGTGTGCCG |
| >Simvastatin Contig 3 |
| GTACAAAAAATAAGCAAAAGCGTCCCAGGAGCCGTACTCTGACAGCTGTGCACGATGCCATCCTTGAGGACTTGGTCTTCCCAAGCGAAATTGTGGGCAAGAGAATCCGCGTCAAACTAGATGGCAGCCGGCTCATAAAGGTTCATTTGGACAAAGCACAGCAGAACAATGTGGAACACAAGGTTGAAACTTTTTCTGGTGTCTATAAGAAGCTCACGGGCAAGGATGTTAATTTTGAATTCCCAGAGTTTCAATTGTAAACAAAAATGACTAAATAAAAAGTATATATTCACAGTAAAAAAAAAAAAAAAAGCT |
| >Simvastatin Contig 4 |
| TCAAGATACAGGAAGCTCAAAGATACCCAAGCAGATCTAACCTAAAAAGGTCTTTCCCAAGGCATGTTATAGTCAAACTGTCAAAACTCAAAGACAACAAGAGGATTCTAAAAACCATAAGATAAATGCATCAGGTCACATATAAGGACATTGCCATCAAACAAACAACAGATTTCTCAGAAGAAACTACAGGCCAGAAGAGCCTGGGATGATGTGTTTAAAATGCTAAAAGTTAAAGAAAAAAAAA |
| >Simvastatin Contig 5 |
| GGTCAGCCAGAGGCCGGAGAGATGATGGGATGACCAACTGCAGAGAGGAGCTACTCACTGTGGGTCTTCTCTGAGCTGTTCTAATACTAAATAAAGYTCTTCGTCTTCTTCACTCTTCACTTGTCTGCGTACCTCATTCTTCCCAGATGCAGGACGAGAACTCGGACAAAGACGCCATGGAGGTTTCCAGGAAGAAAATCAACACCAATCAACTCCCCAAAGATCCCGAAACAAAGGATCCTAATGTACTTGCTACTCCCGTCCCATCAGGACCAATCAGCATAGTGTCAATGGTGTGGCCCACTATGGTTTTCTGTGGCATCTCTGTGGATGCTATCATGGCAGAGAGAGCAGGAAAACTGGCATAACCCTGAGGCAAACTGGGAAGGGGTGCTGTGGGTGCCCTAGACACGAGCAGACnTGtTTTCTGGTGTTCCTGGAAATTGACATAGAGATTAAGAGCCAACTCTCAGTGCTGCgGGGGCTGCAACTGTGGCTCCCAATGAAGA |
| >Simvastatin Contig 6 |
| CCATTCCTTCTGAAACTATTCCAATCAATAGAAAAAGAGGGAATCCTCCCTAACTCATTTTATGAGGCCAGCATCATTCTGATACCAAAGCTGGGCAGAGACACAACCAAAAAAGAGAATTTTAGCCCAATATCCTTGATGAACATTGATGCAAAAATCCTCAATAAAATACTGGCAAAACAAATCCAGCAGCTCATCAAAAAGCTTGCGGCCGCACTCGAGTAACTAGTTAACCCCTTGGGGCCTCTAAACGGGTCTTGAGGGGTTAACTGGTTAC |
| >Simvastatin Contig 7 |
| GTCAGGAGTTCAATACCACCCTGGCCAACATAGTGAAATCCCATCTCTACTAAAAATACAAAAATTAGCCATGCATGATGGTGCATGCCGATAGTCCCAGCTACTAGGGAGGCTGAGGCAGAAGAATTGCTTGAACCTGGGAGGTGGAGGTTGCAGTCAGCTGAGATTGCACCACTGTACTCCAGGCTGGGTGACAGAGTGAGACTTCATCTCAAAAAAAAAAAAAAAAAAAAAAAAAAAAAAAAAAAAAAAAAAAAAAAAAAAAAAAAAAAAAAAAAAAA |
| >Simvastatin Contig 8 |
| ATACCAAAGCTGGGCAGAGACACAACCAAAAAAGAGAATTTTAGCCCAATATCCTTGATGAACATTGATGCAAAAATCCTCAATAAAATACTGGCAAAACAA |
| >Simvastatin Contig 9 |
| AAGAATTGTCTTGGGCCACCCATAAAATACACTAACACTAATAATAGCTGATGAGCTAAAAAAAAAAAAAAAATCSCAAAAAAAAATCCCCAAAAAAATC |
| >Simvastatin Contig 10 |
| TCATTTTATGAGGCCAGCATCATTCTGATACCAAAGCTGGGCAGAGACACAACCAAAAAAGAGAATTTTAGCCCAATATCCTTGATGAACATTGATGCAAAAATCCTCAATAAAATACTGGCAAAACAA |
| >Simvastatin Contig 11 |
| AGGATCGTTTGAGCCTGGAGCTCAAGACTAGCCCCGGCAACATTGCGAGACCCTGTCTCTACAAAAAAGAAATCTAAAAATTAGCCAGCATGTAGTGTGCACCTGTAGTACTAGCTACTTGGGAGGCTGAAATGGGAGGACGGCTTA |
| >Simvastatin Contig 12 |
| CCACCTTGTCACTCCTAATCCAWKGATCACTGAAGGCAAGCTTGCGGCCGCACTCGAGTAACTAGTTAACCCCTTGGGGCCTCTAAACGGGTCTTGAGGG |
| >Simvastatin Contig 13 |
| CAATGTGGAACACAAGGTTGAAACTTTTTCTGGTGTCTATAAGAAGCTCACGGGCAAGGATGTTAATTTTGAATTCCCAGAGTTTCAATTGTAAACAAAAATGACTAAATAAAAAGTATATATTCACAGTAAAAAAAAAAAAAAAAG |
| >Simvastatin Contig 14 |
| CAAGACCCTGTCTCAAATATAAATAAATACATTTTAAAAAGAAATAAAAAAGGAAACACAAAGCAKTCTTGGCTCCATAGAGTTTTCTTTTCTCAGTTTTAAGAGTTATTTCTTATGCCTGTAATCCCAGCACTTTGGGAGGCTGAGGCAGGAGGATCACTTGAGTTCAGGAATCCAAGGCCAGCCTGGGCAACATGGCGAAACCCTATCTCTACAAAAAATACAAAAAATAAGGCCAGGCATGCTGGCGGGCGCCTGTAATCCTAGCTACTTGGGAGGCTGAGGCAGGAGAATGGCGTGAACTCGGGAGGCGGAGGTTGCAGTGAGCCCAAATTACGCCACTGCACTCCAGCCTTGGGTGACAGAGCGAGACTCTGTCTCAAAAAAAA |
| >Simvastatin Contig 15 |
| CCTCCCAAGTAGCTGGGATTACAGACGCCCGCCATCTCGTCCGTCTAATTTTTGTATTTTTTAGTAGAGACGGGGTTTCACCACGTTGGCCAGGCTGCTCTCAAACTCCTGACCTCAGGTGATCCGCCACCTCAGCCTCCCAAAGTGGTGGGATTACAGGCGTGAGCCACTGCACCCGGCCTAGAATTTCTTTAAACAATAAGACTTCTGATATGTAGCTTGAAGAGAGAAATGACTATTCAGATATGATCGTCAGGTATACATCCAAACCTTTCTAAAGAAAAGCCTAGGATTAAGCTTGCGGCCGCACTCGAGTAACTAGTTAACCCCTTGGGGCCTCTAAACGGGTCTTGAGGGGTTAACTAGTTA |
| >Simvastatin Contig 16 |
| AGAGACTGAAAACATGGCAGTGAACTGCCTGAGGTTTTCATTCTCTGTTCATCTCTAATTTTATCTGGACTCTATTGTCCCTGCCCTAGTCAACTGGGAATCTATTCTCAACATTTGCTCCTCAATGTAGGGCTTTGTCATGGAAAGTCAGTTTGGTTTGTTACTTTCAAGAATTCTGTGATTAAAGTAAGCTGGACGCACAGATTTTAAAACATAATTGGATTAATTGACATTTTTCTGTCCCTACTTTATTAGAAACATTATGCATTTAGCTTTTCAAATATATACAGTTTTTTTACTCTAAACTTGA |
| >Simvastatin Contig 17 |
| AGAACCCCATCTCTAAAAAAAGAAAAAAAATCGTATAAAAAATAAAAAGTAAAAAAAGAAAAAATTACTAAACAAAGAAACAAACAAAATATGGTAGCACATATTGTAATGCCAAAGGTTCTTGCCTTAGCCACGCCAAAGAATTAGTGTGGCGGCTGCCCGCGGAGAGTGATGGAGACACGGACCAAGAGAAAAAAAGCTGTAGGCTTTATTGAGCAGAGTGACAGTACAAAGCTTGCGGCCGCACTCGAGTAACTAGTTAACCCCTTGGGGCCTCTAAACGGGTCTTGAGGGGTTAACTAGTTACTCGAGTGCGGCCGC |
| >Simvastatin Contig 18 |
| AAAAGGCAATAAATTAAAATACACTTCAAGGTAAATTAGAAACACTGTTATTTATTTCTTTATTCATTTATTTATTTACTTTAGAGACATGGTCTCACTCTGTTGCCACAATCATAGCTCACTGCA |
| >Simvastatin Contig 19 |
| CTCCAGCCACCCCGCAGTCACTTTCTTTGTAACAACTTCCGTTGCTGCCATCGTAAACTGACACAGTGTTTATAACGTGTACATACATTAACTTATTACCTCATTTTGTTATTTTTCGAAACAAAGCCCTGTGGAAGAAAATGGAAAACTTGAAGAAGCATTAAAGTCATTCTGTTAAGCTGCGAAAAAAAAAAAAAA |
| >Simvastatin Contig 20 |
| ACTAAATAAAGTTCTTCGTCTTCTTCACTCTTCACTTGTCTGCGTACCTCATTCTTCCCAGATGCAGGACGAGAACTCGGACAAAGACGCCATGGAGGTTTCCAGGAAGAAAATCAACACCAATCAACTCCCCAAAGATCCCGAAACAWAGGATCCTAATGTACTTGCTACTCCCGTCCC |
| >Simvastatin Contig 21 |
| TCAGCCCATAAAATCATTTTTCTCTCTTTGGCCTCCAGGCCTGTGACGGGAAGTGCTGCTGTGGTTTCTGACATGCCCCGGAGACATTTTCCCCATTGTCTTGGTAATTAACCTTGGGCTCCTTGTTACATATGCTAATTTCTGCAGCAGGCTTGAATTTCTCCCAAGAAAATCTTTATTTCTTTTCTATCGCATCATCAGACTGCAAATTTTCCAAACTTTTATGCTCTGCTTCCTCTTGAACGCTTTGTTG |
| >Simvastatin Contig 22 |
| GGCTGTTGTTGGTGTACAGGAATGTTAGTGATTTTTGCACATTGATTTTGTATTGTGAGACTTTGCTGAAGTTGTTTATCAGCTTAAGGAGCTTTTGGACTGAGACTGTGGGGTTTTCTAGATATAGGATTATGTCATCTGCAAACAGGGATAGTTTGACTTCTTCTCTTCTTATTTGGATGCCCTTTATTTCTTTCTCTTGTCTAATTGCCCTGCCCAGGACTTCCAATACTATGTTGAATAGGAGTGGTGAGAGAGGGCATCCTTGTCTTGTGCTGATTTTCAAGGGGAATGCTTCCAGCTTTTGCCCATTCAGTATGATGTTGGCTGTGGGTCGAAAAAAAAAAA |
| >Simvastatin Contig 23 |
| CTAAAAATACAAAAATTAGCCATGCATGATGGTGCATGCCGATAGTCCCAGCTACTAGGGAGGCTGAGGCAGAAGAATTGCTTGAACCTGGGAGGTGGAGGTTGCAGTCAGCTGAGATTGCACCACTGTACTCCAGGCTGGGTGACAGAGTGAGACTTCATCTC |
| >Simvastatin Contig 24 |
| TACTCCAGGCCAGGAATGTCCAAAATTTTGGCTTCCCCTGGGCCACATCATAGAAGAATTGTCTTGGGCCACCCATAAAATACACTAACACTAATAATAGCTGATGAGCTAAAAAAAAAAAAAAAAT |
| >Simvastatin Contig 25 |
| CCTTTATAAAGCCAAAGAAACCCCACAAAAAAACCTTCAACTATTCGAACAATGGTAAATTTTAGAAAAGACTACATTAGCATTAAAAAAAGATCTAAGTTCCTTCAGAAATTGACTTTCATATATGCCACACTGCAGCCCAAAGACAGAAAAATAATAGTGGTTTATGACTATATGTTATGACTTTCCTAAATCTATGGAGTAAGTCCTAAACATAGAAAAGAAAGACATCATTCACATCTCTCTGTAACAAACTTCTTACCTTCTTAAAGGAAGCACAGAAGCTTGCGGCCGCACTCGAGTAACTAGTTAACCCCTTGGGGCCTCTAAACGGGTCTTGAGGGGTTAACTAGTT |
| >Simvastatin Contig 26 |
| GGACCCTGAGGAAACCATTCTCAACGCATTCAAAGTGTTTGACCCTGAAGGCAAAGGGGTGCTGAAGGCTGATTACGTTCGGGAAATGCTGACCACGCAGGCGGAGAGGTTTTCCAAGGAGGAGGTTGACCAGATGTTCGCCGCCTTCCCCCCTGACGTGACTGGCAACTTGGACTACAAGAACCTGGTGCACATCATCACCCACGGAGAAGAGAAGGACTAGGAGGGGGCTCGCTGCTGCGCCCTGGGCTCGTCTTTGCAGAGTGGTCCCTGCCCTCATCTCTCTCCCCCGAGTACCGCCTCTGTCCCTACCTTGTCTGTTAGCCATGTGGCTGCCCCATTTATCCACCTCCATCTTCTTTGCAGCCTGGGTGGCTATGGGTACTTCGTGGCCGCWCATCCTACAGTTGGAAATCCATCCAGAGGCCATGTTCCAATAAACAGGAGGTCGTGTAAAAAAAAAAAAAAAAK |
| >Simvastatin Contig 27 |
| GGAAAAAGGCAATAAATTAAAATACACTTCAAGGTAAATTAGAAACACTGTTATTTATTTCTTTATTCATTTATTTATTTACTTTAGAGACATGGTCTCACTCTGTTGCCACAATCATAGCTCACTGCA |
| >Simvastatin Contig 28 |
| CAGAAACTATAATATTTTAGGAGTTGTAAGAATACTATGTAAAACATGTTTCACATAAAAATGTTAATATGAATTACATAAATTTTTAAATATAGAAAGATTGGTCAAGCGCAATGGCTCACGCCTGTAATTCCAGCACTTTGAGAGGCCGAGGC |
| >Simvastatin Contig 29 |
| GAAGCAAGAGGCGCCCCACGGGGGTGGAGCGCCGGCTCTCCTCCGCCAGCAGCCGCCGCAGGAACCTGCCACACATCCTGTTTTGGAGCAGAGGACAAGAGATCAGTGTTGTTCACTGTCCTTCAGAGGGAGCTCCAGTCCACCTTGTCACTCCTAATCCAAGGATCACTGAAGG |
| >Simvastatin Contig 30 |
| TTCTGATACCAAAGCTGGGCAGAGACACAACCAAAAAAGAGAATTTTAGCCCAATATCCTTGATGAACATTGATGCAAAAATCCTCAATAAAATACTGGCAAAACAA |
| >Simvastatin Contig 31 |
| AGAGACGGGGTTTCATCCTGTTTGTCAGGCTGGTCTTGAACTCCTGACCTCAGGCAGTCCACCCACCTCATCCTCACAAAGTGCTGGGTGGATTACAGGTGTAAGCCACCACATCCGGCCACTAGTATTTTATTTTTTTTAGGGTGGTAAATGTAATGGACTCACAAATTCTTTCCAAGGGATTATGGACCTTCGGTATTTGAAATAAAAAGACAGTTGGAATTTTTTGCTTCCGATAGTAAGACTATACTGGTCAGGCACTGTCTATTCTGATGGAGCAGCTGTTGCTGCTTGGCTGTCTTTCAGAAGCAAGCTGCTCACACTGATATTGGTTGGTGAGCAAGGCCAGTGGTCATTGATCGATTGACTAGATTTTGAACTGGCTCTGGCTGGCTTCTTGTTACCATGGCTACAGGTCAATTCTTTCCTAAGTTTGAGTCAAACTTTAACCAGAAATTTTTCTGTTCAAA |

| SimvaBP2 | (51>321) | Contig 6 |
| --- | --- | --- |
| SimvaAP4 | (51>285) | Contig 7 |
| SimvaAP6 | (123>229 | Contig 6 |
| SimvaDP9 | (43>411) | Contig 15 |
| SimvaCP11 | (62>366) | Contig 1 |
| SimvaBP12 | (129>438 | Contig 16 |
| SimvaBP13 | (42>362) | Contig 17 |
| SimvaBP14 | (86>211) | Contig 18 |
| SimvaDP20 | (131>328 | Contig 19 |
| SimvaBP23 | (138>317 | Contig 20 |
| SimvaBP24 | (123>375 | Contig 21 |
| SimvaCP33 | (46>357) | Contig 3 |
| SimvaBP35 | (41>302) | Contig 7 |
| SimvaBP36 | (91>438) | Contig 22 |
| SimvaAP39 | (91>254) | Contig 23 |
| SimvaDP40 | (87>235) | Contig 6 |
| SimvaCP43 | (91>233) | Contig 1 |
| SimvaBP45 | (87>235) | Contig 6 |
| SimvaBP46 | (33>159) | Contig 24 |
| SimvaBP47 | (92>240) | Contig 6 |
| SimvaDP53 | (95>239) | Contig 6 |
| SimvaCP55 | (59>564) | Contig 5 |
| SimvaBP57 | (32>386) | Contig 25 |
| SimvaBP58 | (91>561) | Contig 26 |
| SimvaDP62 | (88>216) | Contig 27 |
| SimvaDP64 | (86>237) | Contig 6 |
| SimvaCP65 | (131>256) | Contig 7 |
| SimvaBP68 | (174>328) | Contig 28 |
| SimvaAP72 | (99>273) | Contig 29 |
| SimvaCP76 | (129>235) | Contig 30 |
| SimvaCP77 | (88>557) | Contig 31 |
| SimvaCP88 | (90>325) | Contig 7 |
| SimvaEP90 | (107>325) | Contig 6 |
| SimvaEN99 | (127>228) | Contig 8 |
| SimvaEP103 | (83>182) | Contig 9 |
| SimvaEP115 | (82>320) | Contig 6 |
| SimvaAN119 | (100>228) | Contig 10 |
| SimvaDN120 | (205>351) | Contig 11 |
| SimvaEN123 | (139>364) | Contig 4 |
| SimvaDN132 | (83>319) | Contig 6 |
| SimvaEN135 | (90>321) | Contig 6 |
| SimvaEN136 | (229>328) | Contig 12 |
| SimvaEP139 | (81>487) | Contig 2 |
| SimvaDN166 | (209>355) | Contig 13 |
| SimvaCN167 | (129>544) | Contig 5 |
| SimvaCP170 | (147>535) | Contig 14 |
| SimvaEP171 | (88>319) | Contig 6 |
| SimvaEP172 | (76>301) | Contig 2 |
| SimvaEP173 | (220>360) | Contig 3 |
| SimvaBN174 | (94>235) | Contig 6 |
| SimvaDN177 | (97>233) | Contig 6 |
| SimvaCN179 | (124>370) | Contig 4 |
