# Supplementary material for: Simvastatin Sodium Salt and Fluvastatin Interact with Human Gap Junction Gamma-3 Protein
Source: PLoS One. 2016 Feb 10;11(2):e0148266. doi: 10.1371/journal.pone.0148266 (PMC4749215; doi:10.1371/journal.pone.0148266)
Supplement: S4 Table — (DOCX) [file pone.0148266.s023.docx]

## S6 Simvastatin Biopan *vs.* Human Vascular Tissue Library Contigs BLASTX Alignment vs. Homo sapiens RefSeq protein

BLASTX 2.2.24+

Reference: Stephen F. Altschul, Thomas L. Madden, Alejandro

A. Schaffer, Jinghui Zhang, Zheng Zhang, Webb Miller, and

David J. Lipman (1997), "Gapped BLAST and PSI-BLAST: a new

generation of protein database search programs", Nucleic

Acids Res. 25:3389-3402.

RID: EYBN4U46014

Database: Homo sapiens RefSeq protein

34,071 sequences; 18,406,605 total letters

Query= Simvastatin Contig 3

Length=302

Score E

Sequences producing significant alignments: (Bits) Value

ref|NP_001002.1| 40S ribosomal protein S7 [Homo sapiens] 171 2e-43

ALIGNMENTS

>ref|NP_001002.1| 40S ribosomal protein S7 [Homo sapiens]

Length=194

Score = 171 bits (432), Expect = 2e-43

Identities = 85/85 (100%), Positives = 85/85 (100%), Gaps = 0/85 (0%)

Frame = +3

Query 3 TKNKQKRPRSRTLTAVHDAILEDLVFPSEIVGKRIRVKLDGSRLIKVHLDKAQQNNVEHK 182

TKNKQKRPRSRTLTAVHDAILEDLVFPSEIVGKRIRVKLDGSRLIKVHLDKAQQNNVEHK

Sbjct 110 TKNKQKRPRSRTLTAVHDAILEDLVFPSEIVGKRIRVKLDGSRLIKVHLDKAQQNNVEHK 169

Query 183 VETFSGVYKKLTGKDVNFEFPEFQL 257

VETFSGVYKKLTGKDVNFEFPEFQL

Sbjct 170 VETFSGVYKKLTGKDVNFEFPEFQL 194

Query= Simvastatin Contig 4

Length=244 Score E

Sequences producing significant alignments: (Bits) Value

ref|NP_061952.3| LINE-1 type transposase domain-containing pr... 46.2 6e-06

ALIGNMENTS

>ref|NP_061952.3| LINE-1 type transposase domain-containing protein 1 [Homo sapiens]

ref|NP_001158307.1| LINE-1 type transposase domain-containing protein 1 [Homo sapiens]

Length=865

Score = 46.2 bits (108), Expect = 6e-06

Identities = 23/67 (35%), Positives = 38/67 (57%), Gaps = 0/67 (0%)

Frame = +3

Query 3 KIQEAQRYPSRSNLKRSFPRHVIVKLSKLKDNKRILKTIR*MHQVTYKDIAIKQTTDFSE 182

+I A R PS+ + KR PRH++VK D ++I++ R ++TY+ I+ T D S

Sbjct 739 EIVSACRVPSKIDEKRLTPRHILVKFWNSSDKEKIIRASRERREITYQGTRIRLTADLSL 798

Query 183 ETTGQKS 203

+T +S

Sbjct 799 DTLDARS 805

Query= Simvastatin Contig 7

Length=218

Score E

Sequences producing significant alignments: (Bits) Value

ref|XP_003121047.1| PREDICTED: KN motif and ankyrin repeat do... 110 3e-25

ref|XP_003118634.1| PREDICTED: putative uncharacterized prote... 102 1e-22

ref|XP_003120094.1| PREDICTED: putative uncharacterized prote... 102 1e-22

ref|XP_003119819.1| PREDICTED: putative uncharacterized prote... 96.7 4e-21

ref|XP_003119960.1| PREDICTED: putative uncharacterized prote... 94.7 2e-20

ref|XP_003119989.1| PREDICTED: hypothetical protein LOC100508... 92.8 6e-20

ref|NP_001158312.1| LYR motif-containing protein 4 isoform 2 ... 89.4 7e-19

ref|XP_003120115.1| PREDICTED: putative uncharacterized prote... 89.0 9e-19

ref|XP_003118843.1| PREDICTED: zinc finger protein ENSP000003... 87.8 2e-18

ref|NP_872601.1| histone demethylase UTY isoform 1 [Homo sapi... 78.6 4e-18

ref|NP_689672.4| hypothetical protein LOC146556 isoform 1 pre... 73.2 2e-17

ref|XP_003118780.1| PREDICTED: hypothetical protein LOC100507... 83.2 5e-17

ref|XP_003119895.1| PREDICTED: uncharacterized protein FLJ395... 79.7 5e-16

ref|XP_003119710.1| PREDICTED: hypothetical protein LOC100507... 77.8 2e-15

ref|NP_001158011.1| disrupted in schizophrenia 1 protein isof... 55.5 6e-15

ref|XP_003119048.1| PREDICTED: hypothetical protein LOC100506... 73.2 5e-14

ref|XP_003120111.1| PREDICTED: putative uncharacterized prote... 72.8 6e-14

ref|NP_001123498.2| hypothetical protein LOC285966 isoform B ... 68.2 7e-14

ref|NP_001164252.1| hypothetical protein LOC159091 isoform 4 ... 65.5 7e-14

ref|XP_003120008.1| PREDICTED: putative uncharacterized prote... 70.9 2e-13

ref|NP_078926.3| putative uncharacterized protein C11orf80 [H... 69.3 7e-13

ref|XP_003119846.1| PREDICTED: hypothetical protein LOC100507... 65.5 1e-11

ref|XP_003118557.1| PREDICTED: histone demethylase UTY-like [... 52.8 1e-11

ref|NP_001136036.1| cGMP-gated cation channel alpha-1 isoform... 63.5 4e-11

ref|XP_003119248.1| PREDICTED: hypothetical protein LOC100506... 58.5 5e-11

ref|NP_963998.2| thromboxane A2 receptor isoform beta [Homo s... 61.6 1e-10

ref|XP_003119783.1| PREDICTED: serine/threonine-protein phosp... 60.8 2e-10

ref|XP_003119925.1| PREDICTED: histone demethylase UTY-like [... 50.8 3e-10

ref|NP_862828.1| zinc finger protein 283 [Homo sapiens] 60.1 4e-10

ref|NP_997719.2| methyltransferase-like protein 10 [Homo sapi... 55.5 8e-10

ref|XP_003119043.1| PREDICTED: hypothetical protein LOC100506... 58.9 9e-10

ref|NP_001077368.1| platelet glycoprotein VI isoform 1 [Homo ... 58.2 2e-09

ref|NP_001171696.1| BEN domain-containing protein 2 isoform 2... 57.4 3e-09

ref|NP_699177.2| BEN domain-containing protein 2 isoform 1 [H... 57.4 3e-09

ref|XP_003120922.1| PREDICTED: putative uncharacterized prote... 57.0 4e-09

ref|NP_114174.1| nuclear prelamin A recognition factor isofor... 57.0 4e-09

ref|XP_003119959.1| PREDICTED: hypothetical protein LOC100287... 56.6 5e-09

ref|NP_001155002.1| granulocyte-macrophage colony-stimulating... 56.2 6e-09

ref|XP_003119678.1| PREDICTED: hypothetical protein LOC100506... 55.8 8e-09

ref|NP_115861.1| peptidyl-prolyl cis-trans isomerase-like 3 i... 54.3 2e-08

ref|NP_001120653.1| centromere protein L isoform 1 [Homo sapi... 52.8 7e-08

ref|NP_689573.3| zinc finger protein 573 isoform 1 [Homo sapi... 52.0 1e-07

ref|NP_001180462.1| serine/threonine-protein kinase Nek4 isof... 52.0 1e-07

ref|NP_003148.2| serine/threonine-protein kinase Nek4 isoform... 52.0 1e-07

ref|XP_003119834.1| PREDICTED: zinc finger protein ENSP000003... 52.0 1e-07

ref|NP_001138489.1| proton-coupled amino acid transporter 3 i... 51.2 2e-07

ref|NP_001185728.1| activating signal cointegrator 1 complex ... 51.2 2e-07

ref|NP_056087.2| protein fantom isoform a [Homo sapiens] 50.1 4e-07

ref|NP_009112.1| mitogen-activated protein kinase kinase kina... 49.7 6e-07

ref|NP_001009923.1| hypothetical protein LOC29058 isoform 1 [... 49.3 7e-07

ref|NP_001011657.2| zinc finger matrin-type protein 1 isoform... 48.9 1e-06

ref|XP_003119660.1| PREDICTED: hypothetical protein LOC100506... 48.5 1e-06

ref|XP_003119709.1| PREDICTED: hypothetical protein LOC100509... 48.5 1e-06

ref|XP_003119509.1| PREDICTED: hypothetical protein LOC100507... 45.4 2e-06

ref|XP_003120664.1| PREDICTED: hypothetical protein LOC100510... 48.1 2e-06

ref|XP_003120124.1| PREDICTED: hypothetical protein LOC100507... 48.1 2e-06

ref|XP_003119948.1| PREDICTED: hypothetical protein LOC100508... 47.8 2e-06

ref|NP_150646.3| alpha-1A adrenergic receptor isoform 2 [Homo... 47.8 2e-06

ref|NP_001153585.1| hypothetical protein LOC123207 isoform b ... 47.4 3e-06

ref|XP_003119972.1| PREDICTED: hypothetical protein LOC100508... 47.4 3e-06

ref|NP_001182556.1| protein AF-10 isoform d [Homo sapiens] 47.4 3e-06

ref|NP_001137385.1| hypothetical protein LOC199870 isoform 2 ... 47.4 3e-06

ref|NP_001137384.1| hypothetical protein LOC199870 isoform 1 ... 47.4 3e-06

ref|NP_078841.3| cyclin-J-like protein [Homo sapiens] 47.0 4e-06

ref|NP_001108224.1| complement decay-accelerating factor isof... 46.6 5e-06

ref|XP_003118848.1| PREDICTED: hypothetical protein LOC100130... 46.2 6e-06

ref|NP_714912.1| interleukin-12 receptor subunit beta-1 isofo... 46.2 6e-06

ref|XP_003119870.1| PREDICTED: hypothetical protein LOC100130... 46.2 6e-06

ref|NP_001128626.1| zinc transporter ZIP14 isoform c [Homo sa... 45.1 1e-05

ref|NP_001165113.1| myosin-IIIb isoform 3 [Homo sapiens] 44.7 2e-05

ref|NP_149084.2| tripartite motif-containing protein 5 isofor... 44.7 2e-05

ref|NP_612412.2| myosin regulatory light chain 10 [Homo sapiens] 44.7 2e-05

ref|NP_874362.3| ankyrin repeat and death domain-containing p... 43.5 4e-05

ref|NP_001003690.1| MAD2L1-binding protein isoform 1 [Homo sa... 43.5 4e-05

ref|NP_683685.1| 39S ribosomal protein L10, mitochondrial iso... 42.7 7e-05

ref|NP_001018114.1| fumarylacetoacetate hydrolase domain-cont... 42.4 9e-05

ref|NP_777547.1| intraflagellar transport protein 20 homolog ... 42.0 1e-04

ref|NP_001139736.1| synaptotagmin-14 isoform 2 [Homo sapiens] 41.6 2e-04

ref|NP_001139733.1| synaptotagmin-14 isoform 1 [Homo sapiens] 41.6 2e-04

ref|NP_060313.3| breast carcinoma-amplified sequence 4 isofor... 41.6 2e-04

ref|NP_001153583.1| nitric oxide synthase, endothelial isofor... 41.2 2e-04

ref|XP_938432.4| PREDICTED: hypothetical protein LOC646021 [H... 40.8 3e-04

ref|NP_001166173.1| probable sodium-coupled neutral amino aci... 40.8 3e-04

ref|NP_001012677.1| arginine-fifty homeobox [Homo sapiens] 40.8 3e-04

ref|NP_000865.2| interferon alpha/beta receptor 2 isoform b p... 39.7 6e-04

ref|NP_001158157.1| protein THEMIS isoform 1 [Homo sapiens] 32.0 7e-04

ref|XP_003119888.1| PREDICTED: hypothetical protein LOC100508... 33.5 0.001

ref|NP_001138525.1| EF-hand calcium-binding domain-containing... 38.9 0.001

ref|NP_001098016.1| ribonuclease P protein subunit p30 isofor... 38.9 0.001

ref|NP_001129224.1| protein SGT1 isoform 2 [Homo sapiens] 38.5 0.001

ref|NP_001030127.1| sorbin and SH3 domain-containing protein ... 38.1 0.002

ref|NP_872321.2| zinc finger protein 714 [Homo sapiens] 37.0 0.004

ref|XP_003119512.1| PREDICTED: hypothetical protein LOC100507... 37.0 0.004

ref|NP_062553.1| putative uncharacterized protein C8orf44 [Ho... 37.0 0.004

ref|XP_002343910.2| PREDICTED: testis-specific Y-encoded prot... 36.6 0.005

ref|NP_065184.2| selenoprotein N isoform 1 precursor [Homo sa... 36.6 0.005

ref|NP_057728.1| proline-rich protein 16 [Homo sapiens] 36.6 0.005

ref|NP_660326.2| nucleoredoxin-like protein 2 isoform 2 [Homo... 36.2 0.007

ALIGNMENTS

>ref|XP_003121047.1| PREDICTED: KN motif and ankyrin repeat domain-containing protein

3-like [Homo sapiens]

Length=143

Score = 110 bits (275), Expect = 3e-25

Identities = 49/69 (72%), Positives = 54/69 (79%), Gaps = 0/69 (0%)

Frame = -3

Query 207 KSHSVTQPGVQWCNLS*LQPPPPRFKQFFCLSLPSSWDYRHAPSCMANFCIFSRDGISLC 28

+SHSV Q GV+W +L LQ PPPRFKQF LSL SSWD+RH P C ANFCIFSRDG+S C

Sbjct 73 ESHSVAQAGVRWHDLGSLQSPPPRFKQFSYLSLLSSWDHRHTPPCPANFCIFSRDGVSPC 132

Query 27 WPGWY*TPD 1

WPGW TPD

Sbjct 133 WPGWSPTPD 141

>ref|XP_003118634.1| PREDICTED: putative uncharacterized protein NCRNA00269-like [Homo

sapiens]

ref|XP_003120632.1| PREDICTED: putative uncharacterized protein NCRNA00269-like [Homo

sapiens]

Length=140

Score = 102 bits (253), Expect = 1e-22

Identities = 52/71 (74%), Positives = 55/71 (78%), Gaps = 0/71 (0%)

Frame = -1

Query 215 LR*SLTLSPSLEYSGAISADCNLHLPGSSNSSASASLVAGTIGMHHHAWLIFVFLVEMGF 36

LR SL L P LE G ISA CNLHLPGSS+ ASAS VAGT G HHAWLIFVFLVE GF

Sbjct 26 LRQSLALLPKLECHGTISAHCNLHLPGSSDFPASASQVAGTTGACHHAWLIFVFLVEAGF 85

Query 35 HYVGQGGIELL 3

H+VGQ G+ELL

Sbjct 86 HHVGQDGLELL 96

>ref|XP_003120094.1| PREDICTED: putative uncharacterized protein NCRNA00269-like [Homo

sapiens]

ref|XP_003119097.1| PREDICTED: putative uncharacterized protein NCRNA00269-like [Homo

sapiens]

Length=140

Score = 102 bits (253), Expect = 1e-22

Identities = 52/71 (74%), Positives = 55/71 (78%), Gaps = 0/71 (0%)

Frame = -1

Query 215 LR*SLTLSPSLEYSGAISADCNLHLPGSSNSSASASLVAGTIGMHHHAWLIFVFLVEMGF 36

LR SL L P LE G ISA CNLHLPGSS+ ASAS VAGT G HHAWLIFVFLVE GF

Sbjct 26 LRQSLALLPKLECHGTISAHCNLHLPGSSDFPASASQVAGTTGACHHAWLIFVFLVEAGF 85

Query 35 HYVGQGGIELL 3

H+VGQ G+ELL

Sbjct 86 HHVGQDGLELL 96

>ref|XP_003119819.1| PREDICTED: putative uncharacterized protein C14orf165-like [Homo

sapiens]

ref|XP_003118891.1| PREDICTED: putative uncharacterized protein C14orf165-like [Homo

sapiens]

ref|XP_003120627.1| PREDICTED: putative uncharacterized protein C14orf165-like [Homo

sapiens]

Length=110

Score = 96.7 bits (239), Expect = 4e-21

Identities = 45/71 (64%), Positives = 50/71 (71%), Gaps = 0/71 (0%)

Frame = -3

Query 213 EMKSHSVTQPGVQWCNLS*LQPPPPRFKQFFCLSLPSSWDYRHAPSCMANFCIFSRDGIS 34

EM+ S Q G+QW +LS LQP PRFKQF CLSLPSS DYRH P + NFCIFSRD +

Sbjct 38 EMEFCSAAQAGMQWLSLSSLQPLHPRFKQFSCLSLPSSCDYRHVPPHLVNFCIFSRDKVL 97

Query 33 LCWPGWY*TPD 1

CWPGW T D

Sbjct 98 PCWPGWSQTSD 108

>ref|XP_003119960.1| PREDICTED: putative uncharacterized protein NCRNA00269-like [Homo

sapiens]

ref|XP_003118569.1| PREDICTED: putative uncharacterized protein NCRNA00269-like [Homo

sapiens]

ref|XP_003120465.1| PREDICTED: putative uncharacterized protein NCRNA00269-like [Homo

sapiens]

Length=128

Score = 94.7 bits (234), Expect = 2e-20

Identities = 50/72 (70%), Positives = 54/72 (75%), Gaps = 0/72 (0%)

Frame = -1

Query 218 FLR*SLTLSPSLEYSGAISADCNLHLPGSSNSSASASLVAGTIGMHHHAWLIFVFLVEMG 39

FLR SL LSP LE SGAISA CNL L GSS S ASAS V+G G HHA L FVFLVE G

Sbjct 23 FLRRSLALSPRLECSGAISAHCNLRLLGSSYSLASASRVSGITGSRHHAQLFFVFLVETG 82

Query 38 FHYVGQGGIELL 3

FH++GQ G+ELL

Sbjct 83 FHHIGQAGLELL 94

>ref|XP_003119989.1| PREDICTED: hypothetical protein LOC100508022 [Homo sapiens]

ref|XP_003118579.1| PREDICTED: hypothetical protein LOC100506688 [Homo sapiens]

ref|XP_003120502.1| PREDICTED: hypothetical protein LOC100508022 [Homo sapiens]

Length=176

Score = 92.8 bits (229), Expect = 6e-20

Identities = 41/54 (76%), Positives = 43/54 (80%), Gaps = 0/54 (0%)

Frame = -3

Query 213 EMKSHSVTQPGVQWCNLS*LQPPPPRFKQFFCLSLPSSWDYRHAPSCMANFCIF 52

E +SHSVTQ GVQWCNLS LQPPPP FKQF CLS PSSW+YRH P C ANF F

Sbjct 62 ETESHSVTQAGVQWCNLSSLQPPPPWFKQFSCLSFPSSWNYRHLPPCPANFLYF 115

Score = 48.5 bits (114), Expect = 1e-06

Identities = 24/33 (73%), Positives = 26/33 (79%), Gaps = 0/33 (0%)

Frame = -1

Query 218 FLR*SLTLSPSLEYSGAISADCNLHLPGSSNSS 120

FLR SL++ P LEYSG ISA CN LPGSSNSS

Sbjct 121 FLRWSLSVLPKLEYSGVISAHCNFCLPGSSNSS 153

>ref|NP_001158312.1| LYR motif-containing protein 4 isoform 2 [Homo sapiens]

Length=130

Score = 89.4 bits (220), Expect = 7e-19

Identities = 43/61 (71%), Positives = 46/61 (76%), Gaps = 0/61 (0%)

Frame = -3

Query 213 EMKSHSVTQPGVQWCNLS*LQPPPPRFKQFFCLSLPSSWDYRHAPSCMANFCIFSRDGIS 34

+M SHSV Q GV W +LS LQP PP FKQF CLSLPSSWDYR P +ANFCI SRD IS

Sbjct 69 QMDSHSVAQAGVHWNDLSSLQPLPPWFKQFSCLSLPSSWDYRRTPPRLANFCILSRDVIS 128

Query 33 L 31

L

Sbjct 129 L 129

>ref|XP_003120115.1| PREDICTED: putative uncharacterized protein NCRNA00269-like [Homo

sapiens]

ref|XP_003119111.1| PREDICTED: putative uncharacterized protein NCRNA00269-like [Homo

sapiens]

ref|XP_003118613.1| PREDICTED: putative uncharacterized protein NCRNA00269-like [Homo

sapiens]

ref|XP_003120653.1| PREDICTED: putative uncharacterized protein NCRNA00269-like [Homo

sapiens]

Length=137

Score = 89.0 bits (219), Expect = 9e-19

Identities = 49/73 (68%), Positives = 54/73 (74%), Gaps = 1/73 (1%)

Frame = -1

Query 218 FLR*SLTLSPSLEYSGAISADCNLHLPGSSNSSASASLVAGTIGMHHHAWLIF-VFLVEM 42

FLR SLTLS LE SGAI A CNL L GS+ ASAS VAG G HHAWLIF VFLVEM

Sbjct 27 FLRWSLTLSSRLECSGAILAHCNLRLLGSNEPPASASRVAGITGACHHAWLIFLVFLVEM 86

Query 41 GFHYVGQGGIELL 3

GF ++GQ G++LL

Sbjct 87 GFRHIGQAGLKLL 99

>ref|XP_003118843.1| PREDICTED: zinc finger protein ENSP00000375192-like [Homo sapiens]

ref|XP_003120983.1| PREDICTED: zinc finger protein ENSP00000375192-like [Homo sapiens]

Length=245

Score = 87.8 bits (216), Expect = 2e-18

Identities = 47/72 (66%), Positives = 51/72 (71%), Gaps = 0/72 (0%)

Frame = -1

Query 218 FLR*SLTLSPSLEYSGAISADCNLHLPGSSNSSASASLVAGTIGMHHHAWLIFVFLVEMG 39

FL+ SLTLSP LE +GAIS CNL L GSS+S AS S AG G HHA LIFVFLVE G

Sbjct 102 FLKWSLTLSPKLECNGAISVHCNLRLLGSSDSLASTSQAAGIAGACHHAQLIFVFLVETG 161

Query 38 FHYVGQGGIELL 3

FH+ Q G ELL

Sbjct 162 FHHFDQAGFELL 173

>ref|NP_872601.1| histone demethylase UTY isoform 1 [Homo sapiens]

Length=1079

Score = 78.6 bits (192), Expect(2) = 4e-18

Identities = 33/48 (69%), Positives = 38/48 (80%), Gaps = 0/48 (0%)

Frame = -3

Query 183 GVQWCNLS*LQPPPPRFKQFFCLSLPSSWDYRHAPSCMANFCIFSRDG 40

G+QWC+LS LQPPPP FK+F LSLP+SW+YRH PSC NFCIF G

Sbjct 997 GMQWCDLSSLQPPPPGFKRFSHLSLPNSWNYRHLPSCPTNFCIFVETG 1044

Score = 28.5 bits (62), Expect(2) = 4e-18

Identities = 12/20 (60%), Positives = 14/20 (70%), Gaps = 0/20 (0%)

Frame = -1

Query 62 FVFLVEMGFHYVGQGGIELL 3

F VE GFH+VGQ +ELL

Sbjct 1037 FCIFVETGFHHVGQACLELL 1056

>ref|NP_689672.4| hypothetical protein LOC146556 isoform 1 precursor [Homo sapiens]

Length=402

Score = 73.2 bits (178), Expect(2) = 2e-17

Identities = 35/51 (69%), Positives = 36/51 (71%), Gaps = 0/51 (0%)

Frame = -3

Query 204 SHSVTQPGVQWCNLS*LQPPPPRFKQFFCLSLPSSWDYRHAPSCMANFCIF 52

S SV Q GVQW NL LQP PP FKQF CL LPSSWDYR P +ANF IF

Sbjct 320 SRSVAQAGVQWRNLGSLQPLPPGFKQFSCLILPSSWDYRSVPPYLANFYIF 370

Score = 32.0 bits (71), Expect(2) = 2e-17

Identities = 12/20 (60%), Positives = 16/20 (80%), Gaps = 0/20 (0%)

Frame = -1

Query 62 FVFLVEMGFHYVGQGGIELL 3

++FLVE GFH+V G+ELL

Sbjct 368 YIFLVETGFHHVAHAGLELL 387

>ref|XP_003118780.1| PREDICTED: hypothetical protein LOC100507131 [Homo sapiens]

ref|XP_003120912.1| PREDICTED: hypothetical protein LOC100507131 [Homo sapiens]

Length=165

Score = 83.2 bits (204), Expect = 5e-17

Identities = 42/72 (59%), Positives = 48/72 (67%), Gaps = 0/72 (0%)

Frame = -3

Query 216 FEMKSHSVTQPGVQWCNLS*LQPPPPRFKQFFCLSLPSSWDYRHAPSCMANFCIFSRDGI 37

FEM+S S Q GVQW +L LQ PRFK+ CLSL SSW YR P ANFC+F+RD +

Sbjct 16 FEMESRSGAQVGVQWRDLGSLQLSSPRFKRLSCLSLLSSWYYRCPPPQPANFCVFTRDRV 75

Query 36 SLCWPGWY*TPD 1

SL WPG TPD

Sbjct 76 SLYWPGRSRTPD 87

>ref|XP_003119895.1| PREDICTED: uncharacterized protein FLJ39582-like [Homo sapiens]

ref|XP_003118861.1| PREDICTED: uncharacterized protein FLJ39582-like [Homo sapiens]

ref|XP_003121056.1| PREDICTED: uncharacterized protein FLJ39582-like [Homo sapiens]

Length=122

Score = 79.7 bits (195), Expect = 5e-16

Identities = 35/51 (69%), Positives = 39/51 (77%), Gaps = 0/51 (0%)

Frame = -3

Query 210 MKSHSVTQPGVQWCNLS*LQPPPPRFKQFFCLSLPSSWDYRHAPSCMANFC 58

+KSHSV Q GVQW +LS QPPPPRFKQF CLS+PSSWDYRH P + C

Sbjct 67 LKSHSVAQAGVQWHDLSSPQPPPPRFKQFSCLSIPSSWDYRHNPDKTSQHC 117

>ref|XP_003119710.1| PREDICTED: hypothetical protein LOC100507131 [Homo sapiens]

Length=165

Score = 77.8 bits (190), Expect = 2e-15

Identities = 38/66 (58%), Positives = 45/66 (69%), Gaps = 0/66 (0%)

Frame = -3

Query 216 FEMKSHSVTQPGVQWCNLS*LQPPPPRFKQFFCLSLPSSWDYRHAPSCMANFCIFSRDGI 37

FEM+S S Q GVQW +L LQ PRFK+ CLSL SSW YR P ANFC+F+R+ +

Sbjct 16 FEMESRSGAQVGVQWRDLGSLQLSSPRFKRLSCLSLLSSWYYRCPPPQPANFCVFTRNRV 75

Query 36 SLCWPG 19

SL WPG

Sbjct 76 SLYWPG 81

>ref|NP_001158011.1| disrupted in schizophrenia 1 protein isoform c [Homo sapiens]

Length=755

Score = 55.5 bits (132), Expect(3) = 6e-15

Identities = 24/32 (75%), Positives = 25/32 (79%), Gaps = 0/32 (0%)

Frame = -3

Query 156 LQPPPPRFKQFFCLSLPSSWDYRHAPSCMANF 61

LQP PP FKQF CLSL SSWDYR P C+ANF

Sbjct 679 LQPLPPEFKQFSCLSLRSSWDYRCPPPCLANF 710

Score = 32.7 bits (73), Expect(3) = 6e-15

Identities = 14/20 (70%), Positives = 18/20 (90%), Gaps = 0/20 (0%)

Frame = -1

Query 62 FVFLVEMGFHYVGQGGIELL 3

FVFLVEMGF++V Q G++LL

Sbjct 710 FVFLVEMGFYHVDQTGLKLL 729

Score = 27.7 bits (60), Expect(3) = 6e-15

Identities = 11/16 (69%), Positives = 12/16 (75%), Gaps = 0/16 (0%)

Frame = -2

Query 211 DEVSLCHPAWSTVVQS 164

D VSLC P WS VV+S

Sbjct 661 DGVSLCRPVWSAVVRS 676

>ref|XP_003119048.1| PREDICTED: hypothetical protein LOC100506073 [Homo sapiens]

ref|XP_003120529.1| PREDICTED: hypothetical protein LOC100506073 [Homo sapiens]

Length=190

Score = 73.2 bits (178), Expect = 5e-14

Identities = 36/62 (59%), Positives = 42/62 (68%), Gaps = 1/62 (1%)

Frame = -3

Query 183 GVQWCNLS*LQPPPPRFKQFFCLSLPSSWDYRHA-PSCMANFCIFSRDGISLCWPGWY*T 7

G+QW +L LQP PP FK+F CLSL SSWD R+ P + FCIFSRDG+S W W T

Sbjct 35 GMQWLHLCSLQPLPPGFKRFSCLSLLSSWDCRYTLPFPVNVFCIFSRDGVSAGWSDWSRT 94

Query 6 PD 1

PD

Sbjct 95 PD 96

>ref|XP_003120111.1| PREDICTED: putative uncharacterized protein NCRNA00269-like [Homo

sapiens]

ref|XP_003119110.1| PREDICTED: putative uncharacterized protein NCRNA00269-like [Homo

sapiens]

ref|XP_003118626.1| PREDICTED: putative uncharacterized protein NCRNA00269-like [Homo

sapiens]

ref|XP_003120652.1| PREDICTED: putative uncharacterized protein NCRNA00269-like [Homo

sapiens]

Length=130

Score = 72.8 bits (177), Expect = 6e-14

Identities = 38/71 (54%), Positives = 51/71 (72%), Gaps = 0/71 (0%)

Frame = -1

Query 215 LR*SLTLSPSLEYSGAISADCNLHLPGSSNSSASASLVAGTIGMHHHAWLIFVFLVEMGF 36

LR SLTL P LE GAI A +L LPG +S AS S ++G + + H++ LIFVF+++MGF

Sbjct 30 LRQSLTLLPRLECGGAILAHYSLCLPGLRDSLASVSQLSGIMCVRHYSRLIFVFVIKMGF 89

Query 35 HYVGQGGIELL 3

H+VGQ G+E L

Sbjct 90 HHVGQAGLEFL 100

>ref|NP_001123498.2| hypothetical protein LOC285966 isoform B [Homo sapiens]

Length=815

Score = 68.2 bits (165), Expect(2) = 7e-14

Identities = 33/46 (72%), Positives = 34/46 (74%), Gaps = 0/46 (0%)

Frame = -1

Query 161 ADCNLHLPGSSNSSASASLVAGTIGMHHHAWLIFVFLVEMGFHYVG 24

+ CNLHL G SNSS SAS VAGT G HHAWLIFVFLVE FH G

Sbjct 390 SSCNLHLLGLSNSSLSASCVAGTTGTRHHAWLIFVFLVEREFHRKG 435

Score = 24.6 bits (52), Expect(2) = 7e-14

Identities = 10/16 (63%), Positives = 13/16 (82%), Gaps = 0/16 (0%)

Frame = -3

Query 207 KSHSVTQPGVQWCNLS 160

+SHSV Q G+QW +LS

Sbjct 375 ESHSVIQVGMQWRDLS 390

>ref|NP_001164252.1| hypothetical protein LOC159091 isoform 4 [Homo sapiens]

Length=98

Score = 65.5 bits (158), Expect(2) = 7e-14

Identities = 35/49 (72%), Positives = 37/49 (76%), Gaps = 0/49 (0%)

Frame = -1

Query 206 SLTLSPSLEYSGAISADCNLHLPGSSNSSASASLVAGTIGMHHHAWLIF 60

SLT+SP LE SG ISA CNL LPGSS+S AS S VAGT G HHA LIF

Sbjct 39 SLTVSPRLECSGMISAHCNLCLPGSSDSPASDSRVAGTSGTRHHAHLIF 87

Score = 27.3 bits (59), Expect(2) = 7e-14

Identities = 10/12 (84%), Positives = 11/12 (92%), Gaps = 0/12 (0%)

Frame = -3

Query 63 FCIFSRDGISLC 28

FCIFSRDG+S C

Sbjct 87 FCIFSRDGVSPC 98

>ref|XP_003120008.1| PREDICTED: putative uncharacterized protein NCRNA00269-like [Homo

sapiens]

ref|XP_003118926.1| PREDICTED: putative uncharacterized protein NCRNA00269-like [Homo

sapiens]

ref|XP_003120534.1| PREDICTED: putative uncharacterized protein NCRNA00269-like [Homo

sapiens]

Length=123

Score = 70.9 bits (172), Expect = 2e-13

Identities = 37/55 (68%), Positives = 41/55 (75%), Gaps = 0/55 (0%)

Frame = -1

Query 167 ISADCNLHLPGSSNSSASASLVAGTIGMHHHAWLIFVFLVEMGFHYVGQGGIELL 3

ISA +LHLPGSS S ASAS +AG M HHA LIFVFLVE GFH VGQ G++ L

Sbjct 2 ISAHGSLHLPGSSYSPASASQIAGITVMCHHAGLIFVFLVETGFHRVGQAGLDFL 56

>ref|NP_078926.3| putative uncharacterized protein C11orf80 [Homo sapiens]

Length=677

Score = 69.3 bits (168), Expect = 7e-13

Identities = 35/52 (68%), Positives = 37/52 (72%), Gaps = 0/52 (0%)

Frame = -1

Query 212 R*SLTLSPSLEYSGAISADCNLHLPGSSNSSASASLVAGTIGMHHHAWLIFV 57

R SLTL P E SGA+SA CNLHLPGSS+S AS VAG HHHAWLI V

Sbjct 106 RWSLTLLPRPECSGAVSAHCNLHLPGSSDSHASVPRVAGITDAHHHAWLIMV 157

>ref|XP_003119846.1| PREDICTED: hypothetical protein LOC100507929 [Homo sapiens]

ref|XP_003119023.1| PREDICTED: hypothetical protein LOC100507664 [Homo sapiens]

ref|XP_003120394.1| PREDICTED: hypothetical protein LOC100507929 [Homo sapiens]

Length=122

Score = 65.5 bits (158), Expect = 1e-11

Identities = 35/60 (59%), Positives = 41/60 (69%), Gaps = 0/60 (0%)

Frame = +1

Query 37 NPISTKNTKISHA*WCMPIVPATREAEAEELLEPGRWRLQSAEIAPLYSRLGDRVRLHLK 216

N STKNTKIS W +P++P T EAEA E LEPG+ LQ I L+S LG+RVRL LK

Sbjct 47 NSTSTKNTKISWVWWQVPVIPTTWEAEAGESLEPGKSSLQRTMILTLHSSLGNRVRLCLK 106

>ref|XP_003118557.1| PREDICTED: histone demethylase UTY-like [Homo sapiens]

Length=101

Score = 52.8 bits (125), Expect(2) = 1e-11

Identities = 29/52 (56%), Positives = 31/52 (60%), Gaps = 0/52 (0%)

Frame = -3

Query 216 FEMKSHSVTQPGVQWCNLS*LQPPPPRFKQFFCLSLPSSWDYRHAPSCMANF 61

F M+S SV GVQW NLS LQPPP FK F LSL SS D R C+ F

Sbjct 3 FGMESCSVPHAGVQWHNLSSLQPPPSGFKPFSYLSLLSSRDQRRPLPCLVTF 54

Score = 32.7 bits (73), Expect(2) = 1e-11

Identities = 13/20 (65%), Positives = 17/20 (85%), Gaps = 0/20 (0%)

Frame = -1

Query 62 FVFLVEMGFHYVGQGGIELL 3

FVFL+E FH+VGQ G++LL

Sbjct 54 FVFLIETRFHHVGQAGLKLL 73

>ref|NP_001136036.1| cGMP-gated cation channel alpha-1 isoform 1 [Homo sapiens]

Length=759

Score = 63.5 bits (153), Expect = 4e-11

Identities = 36/64 (57%), Positives = 40/64 (63%), Gaps = 17/64 (26%)

Frame = -1

Query 194 SPSLEYSGAISADCNLHLPGSSNSSASASLVAGTIGMHHHAWLIFVFLVEMGFHYVGQGG 15

SP LE SGAISA C+LHLP SS+ LIFVFLVEMGFH+VGQ G

Sbjct 6 SPRLECSGAISAHCSLHLPDSSDFQ-----------------LIFVFLVEMGFHHVGQAG 48

Query 14 IELL 3

+ELL

Sbjct 49 LELL 52

>ref|XP_003119248.1| PREDICTED: hypothetical protein LOC100506511 [Homo sapiens]

Length=402

Score = 58.5 bits (140), Expect(2) = 5e-11

Identities = 28/45 (63%), Positives = 32/45 (72%), Gaps = 0/45 (0%)

Frame = -3

Query 189 QPGVQWCNLS*LQPPPPRFKQFFCLSLPSSWDYRHAPSCMANFCI 55

Q GV+ +L LQP PP FK+F CLSLPSSW Y HAPS ANF +

Sbjct 308 QAGVRCRDLDSLQPLPPGFKRFSCLSLPSSWGYSHAPSRPANFVV 352

Score = 24.6 bits (52), Expect(2) = 5e-11

Identities = 12/20 (60%), Positives = 14/20 (70%), Gaps = 0/20 (0%)

Frame = -1

Query 62 FVFLVEMGFHYVGQGGIELL 3

FV LVE F +V Q G+ELL

Sbjct 350 FVVLVETVFLHVDQAGLELL 369

>ref|NP_963998.2| thromboxane A2 receptor isoform beta [Homo sapiens]

Length=407

Score = 61.6 bits (148), Expect = 1e-10

Identities = 33/47 (71%), Positives = 35/47 (75%), Gaps = 0/47 (0%)

Frame = -1

Query 212 R*SLTLSPSLEYSGAISADCNLHLPGSSNSSASASLVAGTIGMHHHA 72

R SLTL PSLEYSG ISA CNL LPGSS+S ASAS AG G+ H A

Sbjct 328 RRSLTLWPSLEYSGTISAHCNLRLPGSSDSRASASRAAGITGVSHCA 374

>ref|XP_003119783.1| PREDICTED: serine/threonine-protein phosphatase 5-like [Homo

sapiens]

ref|XP_003118828.1| PREDICTED: serine/threonine-protein phosphatase 5-like [Homo

sapiens]

ref|XP_003120999.1| PREDICTED: serine/threonine-protein phosphatase 5-like [Homo

sapiens]

Length=171

Score = 60.8 bits (146), Expect = 2e-10

Identities = 30/51 (59%), Positives = 36/51 (71%), Gaps = 0/51 (0%)

Frame = -1

Query 203 LTLSPSLEYSGAISADCNLHLPGSSNSSASASLVAGTIGMHHHAWLIFVFL 51

L LSP L+ SG I+A C+L+L G + SAS VA T GMHHH WLIF+FL

Sbjct 121 LALSPRLKCSGTITAHCSLNLLGPRDPPTSASQVAVTEGMHHHTWLIFLFL 171

>ref|XP_003119925.1| PREDICTED: histone demethylase UTY-like [Homo sapiens]

ref|XP_003120437.1| PREDICTED: histone demethylase UTY-like [Homo sapiens]

Length=101

Score = 50.8 bits (120), Expect(2) = 3e-10

Identities = 28/52 (54%), Positives = 30/52 (58%), Gaps = 0/52 (0%)

Frame = -3

Query 216 FEMKSHSVTQPGVQWCNLS*LQPPPPRFKQFFCLSLPSSWDYRHAPSCMANF 61

F M+S SV GVQW NLS LQPPP FK F LSL S D R C+ F

Sbjct 3 FGMESCSVPHAGVQWHNLSSLQPPPSGFKPFSYLSLLRSRDQRRPLPCLVTF 54

Score = 29.6 bits (65), Expect(2) = 3e-10

Identities = 12/20 (60%), Positives = 16/20 (80%), Gaps = 0/20 (0%)

Frame = -1

Query 62 FVFLVEMGFHYVGQGGIELL 3

FVFL+E F +VGQ G++LL

Sbjct 54 FVFLIETRFRHVGQAGLKLL 73

>ref|NP_862828.1| zinc finger protein 283 [Homo sapiens]

Length=679

Score = 60.1 bits (144), Expect = 4e-10

Identities = 30/51 (59%), Positives = 33/51 (65%), Gaps = 3/51 (5%)

Frame = -3

Query 210 MKSHSVTQPGVQWCNLS*LQPPPPRFKQFFCLSLPSSWDYRHAPSCMANFC 58

M+S SV Q GVQWC+L LQ PPP F F CLSL SSWDY S + FC

Sbjct 1 MESRSVAQAGVQWCDLGSLQAPPPGFTLFSCLSLLSSWDY---SSGFSGFC 48

>ref|NP_997719.2| methyltransferase-like protein 10 [Homo sapiens]

Length=291

Score = 55.5 bits (132), Expect(2) = 8e-10

Identities = 26/40 (65%), Positives = 29/40 (73%), Gaps = 0/40 (0%)

Frame = -1

Query 122 SASASLVAGTIGMHHHAWLIFVFLVEMGFHYVGQGGIELL 3

S SAS V GT G HHHAW+IFVFL E F +V Q G+ELL

Sbjct 228 STSASRVGGTTGTHHHAWIIFVFLAETRFCHVVQAGLELL 267

Score = 23.5 bits (49), Expect(2) = 8e-10

Identities = 12/20 (60%), Positives = 12/20 (60%), Gaps = 0/20 (0%)

Frame = -2

Query 184 WSTVVQSQLTATSTSQVQAI 125

WSTV LTA TS QAI

Sbjct 207 WSTVAGFWLTAALTSWAQAI 226

>ref|XP_003119043.1| PREDICTED: hypothetical protein LOC100506191 [Homo sapiens]

Length=118

Score = 58.9 bits (141), Expect = 9e-10

Identities = 30/51 (59%), Positives = 34/51 (67%), Gaps = 0/51 (0%)

Frame = +1

Query 4 RSSIPPWPT**NPISTKNTKISHA*WCMPIVPATREAEAEELLEPGRWRLQ 156

RSS P WPT NP+STKNTKIS A W MP++ AT E E L P R R+Q

Sbjct 68 RSSRPAWPTWRNPVSTKNTKISQAWWSMPMISATWETEVGGSLGPRRQRVQ 118

>ref|NP_001077368.1| platelet glycoprotein VI isoform 1 [Homo sapiens]

Length=620

Score = 58.2 bits (139), Expect = 2e-09

Identities = 32/55 (59%), Positives = 37/55 (68%), Gaps = 0/55 (0%)

Frame = +2

Query 53 KIQKLAMHDGACR*SQLLGRLRQKNCLNLGGGGCSQLRLHHCTPGWVTE*DFISK 217

K QKLA GA SQ L LR +N L+LGG GCS+LR HHCT VT+ DF+SK

Sbjct 555 KTQKLARCGGASLYSQQLRGLRWENGLSLGGRGCSELRSHHCTLARVTKPDFVSK 609

>ref|NP_001171696.1| BEN domain-containing protein 2 isoform 2 [Homo sapiens]

Length=645

Score = 57.4 bits (137), Expect = 3e-09

Identities = 29/44 (66%), Positives = 30/44 (69%), Gaps = 0/44 (0%)

Frame = -3

Query 204 SHSVTQPGVQWCNLS*LQPPPPRFKQFFCLSLPSSWDYRHAPSC 73

S SVTQ GVQW + S LQP P KQFF LSLPSSWD R P C

Sbjct 81 SGSVTQAGVQWHDHSSLQPQPLGLKQFFHLSLPSSWDDRRTPPC 124

>ref|NP_699177.2| BEN domain-containing protein 2 isoform 1 [Homo sapiens]

Length=799

Score = 57.4 bits (137), Expect = 3e-09

Identities = 29/44 (66%), Positives = 30/44 (69%), Gaps = 0/44 (0%)

Frame = -3

Query 204 SHSVTQPGVQWCNLS*LQPPPPRFKQFFCLSLPSSWDYRHAPSC 73

S SVTQ GVQW + S LQP P KQFF LSLPSSWD R P C

Sbjct 81 SGSVTQAGVQWHDHSSLQPQPLGLKQFFHLSLPSSWDDRRTPPC 124

>ref|XP_003120922.1| PREDICTED: putative uncharacterized protein NCRNA00269-like [Homo

sapiens]

Length=127

Score = 57.0 bits (136), Expect = 4e-09

Identities = 28/36 (78%), Positives = 30/36 (84%), Gaps = 0/36 (0%)

Frame = -1

Query 203 LTLSPSLEYSGAISADCNLHLPGSSNSSASASLVAG 96

LTLSP LEYSG SA C+LHLPGSSN+ ASAS VAG

Sbjct 77 LTLSPRLEYSGTTSAHCSLHLPGSSNAPASASRVAG 112

Score = 40.8 bits (94), Expect = 3e-04

Identities = 22/40 (55%), Positives = 27/40 (68%), Gaps = 0/40 (0%)

Frame = -1

Query 203 LTLSPSLEYSGAISADCNLHLPGSSNSSASASLVAGTIGM 84

L LS +E+SG I A +L LPGSSN + SA VAGT G+

Sbjct 38 LALSLRMEFSGPIMAHYSLKLPGSSNPTMSAFPVAGTTGL 77

>ref|NP_114174.1| nuclear prelamin A recognition factor isoform b [Homo sapiens]

Length=502

Score = 57.0 bits (136), Expect = 4e-09

Identities = 28/45 (63%), Positives = 33/45 (74%), Gaps = 0/45 (0%)

Frame = +1

Query 58 TKISHA*WCMPIVPATREAEAEELLEPGRWRLQSAEIAPLYSRLG 192

++IS A WC P++ ATREA A E LEPGR RLQ +IAPL S LG

Sbjct 256 SEISQAWWCTPVITATREAAARESLEPGRQRLQRDKIAPLDSSLG 300

>ref|XP_003119959.1| PREDICTED: hypothetical protein LOC100287290 [Homo sapiens]

ref|XP_002342446.2| PREDICTED: putative uncharacterized protein C3orf66 [Homo sapiens]

ref|XP_002346604.2| PREDICTED: putative uncharacterized protein C3orf66 [Homo sapiens]

Length=70

Score = 56.6 bits (135), Expect = 5e-09

Identities = 25/41 (61%), Positives = 31/41 (76%), Gaps = 4/41 (9%)

Frame = -3

Query 210 MKSHSVTQPGVQWCNLS*LQPPPPRFKQFFCLSLPSSWDYR 88

M+SH+VT+ G+QWC+L PPRFK+F CL LPSSWD R

Sbjct 1 MESHTVTRAGMQWCDLG----SPPRFKRFCCLCLPSSWDSR 37

>ref|NP_001155002.1| granulocyte-macrophage colony-stimulating factor receptor subunit

alpha isoform f precursor [Homo sapiens]

Length=434

Score = 56.2 bits (134), Expect = 6e-09

Identities = 27/40 (68%), Positives = 28/40 (70%), Gaps = 0/40 (0%)

Frame = -3

Query 213 EMKSHSVTQPGVQWCNLS*LQPPPPRFKQFFCLSLPSSWD 94

E SHSVTQ GVQW NL LQPP PR K+F CL LP S D

Sbjct 314 EFGSHSVTQAGVQWHNLGSLQPPSPRLKRFSCLRLPGSDD 353

>ref|XP_003119678.1| PREDICTED: hypothetical protein LOC100506928 [Homo sapiens]

ref|XP_003118761.1| PREDICTED: hypothetical protein LOC100506928 [Homo sapiens]

ref|XP_003120899.1| PREDICTED: hypothetical protein LOC100506928 [Homo sapiens]

Length=159

Score = 55.8 bits (133), Expect = 8e-09

Identities = 30/50 (60%), Positives = 33/50 (66%), Gaps = 0/50 (0%)

Frame = -1

Query 215 LR*SLTLSPSLEYSGAISADCNLHLPGSSNSSASASLVAGTIGMHHHAWL 66

LR LTL P LE SG I+A +L LPGSSN SAS +GT GM HH WL

Sbjct 104 LREGLTLLPRLECSGMITAHYSLGLPGSSNPPTSASQGSGTTGMCHHTWL 153

>ref|NP_115861.1| peptidyl-prolyl cis-trans isomerase-like 3 isoform PPIL3a [Homo

sapiens]

Length=165

Score = 54.3 bits (129), Expect = 2e-08

Identities = 25/36 (70%), Positives = 27/36 (75%), Gaps = 0/36 (0%)

Frame = -3

Query 213 EMKSHSVTQPGVQWCNLS*LQPPPPRFKQFFCLSLP 106

EM+S V Q GVQW +L LQPPPP FKQ FCLSLP

Sbjct 26 EMESRCVPQAGVQWRDLGSLQPPPPGFKQVFCLSLP 61

>ref|NP_001120653.1| centromere protein L isoform 1 [Homo sapiens]

Length=390

Score = 52.8 bits (125), Expect = 7e-08

Identities = 29/52 (56%), Positives = 32/52 (62%), Gaps = 0/52 (0%)

Frame = -1

Query 218 FLR*SLTLSPSLEYSGAISADCNLHLPGSSNSSASASLVAGTIGMHHHAWLI 63

FL L LSP LEYSG I DCNL L GSS+ S A VAGT G HH ++

Sbjct 137 FLVQGLILSPRLEYSGTILVDCNLCLLGSSDPSTLAFQVAGTAGACHHTRIV 188

>ref|NP_689573.3| zinc finger protein 573 isoform 1 [Homo sapiens]

Length=607

Score = 52.0 bits (123), Expect = 1e-07

Identities = 25/34 (74%), Positives = 27/34 (80%), Gaps = 0/34 (0%)

Frame = -3

Query 210 MKSHSVTQPGVQWCNLS*LQPPPPRFKQFFCLSL 109

M+S SV Q GVQW +LS LQPPPPRFKQF C SL

Sbjct 1 MESCSVAQAGVQWPDLSSLQPPPPRFKQFSCHSL 34

>ref|NP_001180462.1| serine/threonine-protein kinase Nek4 isoform 2 [Homo sapiens]

Length=752

Score = 52.0 bits (123), Expect = 1e-07

Identities = 29/45 (65%), Positives = 31/45 (69%), Gaps = 0/45 (0%)

Frame = -1

Query 206 SLTLSPSLEYSGAISADCNLHLPGSSNSSASASLVAGTIGMHHHA 72

SL LSP LE SG I A NL L GSS+S ASAS VAG G+ HHA

Sbjct 368 SLALSPKLECSGTILAHSNLRLLGSSDSPASASRVAGITGVCHHA 412

>ref|NP_003148.2| serine/threonine-protein kinase Nek4 isoform 1 [Homo sapiens]

Length=841

Score = 52.0 bits (123), Expect = 1e-07

Identities = 29/45 (65%), Positives = 31/45 (69%), Gaps = 0/45 (0%)

Frame = -1

Query 206 SLTLSPSLEYSGAISADCNLHLPGSSNSSASASLVAGTIGMHHHA 72

SL LSP LE SG I A NL L GSS+S ASAS VAG G+ HHA

Sbjct 457 SLALSPKLECSGTILAHSNLRLLGSSDSPASASRVAGITGVCHHA 501

>ref|XP_003119834.1| PREDICTED: zinc finger protein ENSP00000375192-like [Homo sapiens]

ref|XP_003119017.1| PREDICTED: zinc finger protein ENSP00000375192-like [Homo sapiens]

ref|XP_003120400.1| PREDICTED: zinc finger protein ENSP00000375192-like [Homo sapiens]

Length=105

Score = 52.0 bits (123), Expect = 1e-07

Identities = 28/53 (53%), Positives = 32/53 (61%), Gaps = 0/53 (0%)

Frame = -1

Query 161 ADCNLHLPGSSNSSASASLVAGTIGMHHHAWLIFVFLVEMGFHYVGQGGIELL 3

A C+L L GS + S S VA TIG HH LIFVF E GFH+V +ELL

Sbjct 2 AHCSLDLSGSGDPPTSTSQVARTIGACHHVQLIFVFFGETGFHHVAPLVLELL 54

>ref|NP_001138489.1| proton-coupled amino acid transporter 3 isoform 1 [Homo sapiens]

Length=511

Score = 51.2 bits (121), Expect = 2e-07

Identities = 29/59 (50%), Positives = 37/59 (63%), Gaps = 3/59 (5%)

Frame = -1

Query 212 R*SLTLSPSLEYSGAISADCNLHLPGSSNSSASASLVAGTIGMHHHAWLIFVFLVEMGF 36

R +L LSP LE SG ISA CN HL GSSNS A AS VA G++ + + + ++GF

Sbjct 135 RWNLALSPRLECSGKISAHCNPHLQGSSNSPAQASRVA---GIYRYTVSFLLVITQLGF 190

>ref|NP_001185728.1| activating signal cointegrator 1 complex subunit 1 isoform a

[Homo sapiens]

Length=400

Score = 51.2 bits (121), Expect = 2e-07

Identities = 27/41 (66%), Positives = 30/41 (74%), Gaps = 0/41 (0%)

Frame = -1

Query 206 SLTLSPSLEYSGAISADCNLHLPGSSNSSASASLVAGTIGM 84

S L P LEY+ AISA CNL LPGSS+S ASAS VAG G+

Sbjct 348 SFALLPRLEYNDAISAHCNLCLPGSSDSPASASQVAGITGV 388

>ref|NP_056087.2| protein fantom isoform a [Homo sapiens]

Length=1315

Score = 50.1 bits (118), Expect = 4e-07

Identities = 27/44 (62%), Positives = 28/44 (64%), Gaps = 0/44 (0%)

Frame = -1

Query 206 SLTLSPSLEYSGAISADCNLHLPGSSNSSASASLVAGTIGMHHH 75

SL LSP L S AISA CN LPGSS+ ASAS V G G HH

Sbjct 1099 SLALSPGLGCSSAISAHCNFRLPGSSDFPASASQVDGITGACHH 1142

>ref|NP_009112.1| mitogen-activated protein kinase kinase kinase kinase 1 isoform

2 [Homo sapiens]

Length=833

Score = 49.7 bits (117), Expect = 6e-07

Identities = 26/37 (71%), Positives = 27/37 (73%), Gaps = 0/37 (0%)

Frame = -1

Query 194 SPSLEYSGAISADCNLHLPGSSNSSASASLVAGTIGM 84

SP LE SG IS CNL LPGSSNS ASAS VAG G+

Sbjct 797 SPRLECSGTISPHCNLLLPGSSNSPASASRVAGITGL 833

>ref|NP_001009923.1| hypothetical protein LOC29058 isoform 1 [Homo sapiens]

Length=183

Score = 49.3 bits (116), Expect = 7e-07

Identities = 28/44 (64%), Positives = 31/44 (71%), Gaps = 0/44 (0%)

Frame = -1

Query 215 LR*SLTLSPSLEYSGAISADCNLHLPGSSNSSASASLVAGTIGM 84

LR SL LSP LE SG ISA CNLHL SS+SSASAS + + M

Sbjct 22 LRWSLVLSPRLEPSGVISAHCNLHLLASSDSSASASRLCQRVMM 65

>ref|NP_001011657.2| zinc finger matrin-type protein 1 isoform 1 [Homo sapiens]

Length=638

Score = 48.9 bits (115), Expect = 1e-06

Identities = 24/31 (78%), Positives = 27/31 (88%), Gaps = 0/31 (0%)

Frame = -1

Query 185 LEYSGAISADCNLHLPGSSNSSASASLVAGT 93

LE SGAISA C+LHLPGSS+S ASAS +AGT

Sbjct 9 LECSGAISAHCSLHLPGSSDSPASASQIAGT 39

>ref|XP_003119660.1| PREDICTED: hypothetical protein LOC100506579 [Homo sapiens]

ref|XP_003118706.1| PREDICTED: hypothetical protein LOC100506579 [Homo sapiens]

ref|XP_003120865.1| PREDICTED: hypothetical protein LOC100510190 [Homo sapiens]

Length=147

Score = 48.5 bits (114), Expect = 1e-06

Identities = 30/70 (43%), Positives = 37/70 (53%), Gaps = 5/70 (7%)

Frame = -2

Query 208 EVSLCHPAWSTVVQSQLTATSTSQVQAILLPQPP**LGLSACTIMHG*FLYF**RW--DF 35

+V LCHP W+TV SQLT SQ Q IL QPP + H +YF W D

Sbjct 30 QVLLCHPGWNTVASSQLTVDLDSQAQVILPLQPP---ARTTAVHCHTRLIYFHMLWRLDN 86

Query 34 TMLARVVLNS 5

+L+R+V NS

Sbjct 87 PVLSRLVSNS 96

>ref|XP_003119709.1| PREDICTED: hypothetical protein LOC100509912 [Homo sapiens]

ref|XP_003118779.1| PREDICTED: hypothetical protein LOC100506809 [Homo sapiens]

ref|XP_003120910.1| PREDICTED: hypothetical protein LOC100509912 [Homo sapiens]

Length=118

Score = 48.5 bits (114), Expect = 1e-06

Identities = 25/40 (63%), Positives = 29/40 (73%), Gaps = 1/40 (2%)

Frame = +1

Query 37 NPISTKNTKISHA*WCMPIVPATREAEAEELLEPGRWRLQ 156

+P KNTKIS A W +P++PA RE EA E LEPGR RLQ

Sbjct 80 SPSLLKNTKISWA-WWVPVIPAIREGEAGESLEPGRQRLQ 118

>ref|XP_003119509.1| PREDICTED: hypothetical protein LOC100507445 [Homo sapiens]

ref|XP_003118667.1| PREDICTED: hypothetical protein LOC100507445 [Homo sapiens]

ref|XP_003120698.1| PREDICTED: hypothetical protein LOC100507445 [Homo sapiens]

Length=121

Score = 45.4 bits (106), Expect(2) = 2e-06

Identities = 22/36 (62%), Positives = 23/36 (64%), Gaps = 0/36 (0%)

Frame = +1

Query 49 TKNTKISHA*WCMPIVPATREAEAEELLEPGRWRLQ 156

TKN IS W P+VPA E EA E LEP RWRLQ

Sbjct 86 TKNINISRVWWQAPVVPAIWETEAGESLEPRRWRLQ 121

Score = 22.3 bits (46), Expect(2) = 2e-06

Identities = 10/15 (67%), Positives = 11/15 (74%), Gaps = 0/15 (0%)

Frame = +3

Query 3 QEFNTTLANIVKSHL 47

QEF T+L NIVK L

Sbjct 71 QEFETSLDNIVKRRL 85

>ref|XP_003120664.1| PREDICTED: hypothetical protein LOC100510512 [Homo sapiens]

Length=177

Score = 48.1 bits (113), Expect = 2e-06

Identities = 29/53 (55%), Positives = 31/53 (59%), Gaps = 0/53 (0%)

Frame = -1

Query 218 FLR*SLTLSPSLEYSGAISADCNLHLPGSSNSSASASLVAGTIGMHHHAWLIF 60

F R L L LE SG I C+L L GS +S SAS VAGT GMH HA L F

Sbjct 109 FERQGLALLSRLECSGMIITYCSLKLLGSRDSPVSASQVAGTTGMHQHARLNF 161

>ref|XP_003120124.1| PREDICTED: hypothetical protein LOC100507236, partial [Homo sapiens]

ref|XP_003119212.1| PREDICTED: hypothetical protein LOC100507236, partial [Homo sapiens]

Length=174

Score = 48.1 bits (113), Expect = 2e-06

Identities = 29/53 (55%), Positives = 31/53 (59%), Gaps = 0/53 (0%)

Frame = -1

Query 218 FLR*SLTLSPSLEYSGAISADCNLHLPGSSNSSASASLVAGTIGMHHHAWLIF 60

F R L L LE SG I C+L L GS +S SAS VAGT GMH HA L F

Sbjct 109 FERQGLALLSRLECSGMIITYCSLKLLGSRDSPVSASQVAGTTGMHQHARLNF 161

>ref|XP_003119948.1| PREDICTED: hypothetical protein LOC100508257 [Homo sapiens]

ref|XP_003118917.1| PREDICTED: hypothetical protein LOC100507149 [Homo sapiens]

ref|XP_003120447.1| PREDICTED: hypothetical protein LOC100508257 [Homo sapiens]

Length=139

Score = 47.8 bits (112), Expect = 2e-06

Identities = 21/38 (56%), Positives = 25/38 (66%), Gaps = 0/38 (0%)

Frame = -1

Query 116 SASLVAGTIGMHHHAWLIFVFLVEMGFHYVGQGGIELL 3

SA V GT G HHAWLI+ +E G HYV Q G++LL

Sbjct 10 SAPQVEGTTGACHHAWLIYFLFLETGSHYVSQAGLQLL 47

>ref|NP_150646.3| alpha-1A adrenergic receptor isoform 2 [Homo sapiens]

Length=475

Score = 47.8 bits (112), Expect = 2e-06

Identities = 25/38 (66%), Positives = 26/38 (69%), Gaps = 0/38 (0%)

Frame = -1

Query 185 LEYSGAISADCNLHLPGSSNSSASASLVAGTIGMHHHA 72

LE SG I A CNL LPGS +S ASAS AGT GM H A

Sbjct 432 LECSGMILAHCNLRLPGSRDSPASASQAAGTTGMCHQA 469

>ref|NP_001153585.1| hypothetical protein LOC123207 isoform b [Homo sapiens]

Length=167

Score = 47.4 bits (111), Expect = 3e-06

Identities = 25/37 (68%), Positives = 27/37 (73%), Gaps = 0/37 (0%)

Frame = -1

Query 218 FLR*SLTLSPSLEYSGAISADCNLHLPGSSNSSASAS 108

FLR +L LSP LE SG + A CNLHL GSS S ASAS

Sbjct 131 FLRQNLALSPKLECSGVVLAHCNLHLLGSSYSPASAS 167

>ref|XP_003119972.1| PREDICTED: hypothetical protein LOC100508228 [Homo sapiens]

ref|XP_003118923.1| PREDICTED: hypothetical protein LOC100506486 [Homo sapiens]

ref|XP_003120486.1| PREDICTED: hypothetical protein LOC100508228 [Homo sapiens]

Length=125

Score = 47.4 bits (111), Expect = 3e-06

Identities = 22/29 (76%), Positives = 24/29 (83%), Gaps = 0/29 (0%)

Frame = -3

Query 216 FEMKSHSVTQPGVQWCNLS*LQPPPPRFK 130

FEM+SHSVTQ GVQW +LS LQP PP FK

Sbjct 88 FEMESHSVTQGGVQWHDLSSLQPQPPGFK 116

Score = 41.6 bits (96), Expect = 2e-04

Identities = 17/27 (63%), Positives = 22/27 (82%), Gaps = 0/27 (0%)

Frame = -1

Query 86 MHHHAWLIFVFLVEMGFHYVGQGGIEL 6

M H WL+FVFLV+MGFH+V Q G++L

Sbjct 1 MCHQVWLVFVFLVKMGFHHVVQVGLKL 27

>ref|NP_001182556.1| protein AF-10 isoform d [Homo sapiens]

Length=126

Score = 47.4 bits (111), Expect = 3e-06

Identities = 22/39 (57%), Positives = 27/39 (70%), Gaps = 0/39 (0%)

Frame = -3

Query 207 KSHSVTQPGVQWCNLS*LQPPPPRFKQFFCLSLPSSWDY 91

+S SV Q VQWC+LS LQP P FK+F CLSLP+ +

Sbjct 82 ESRSVAQAKVQWCDLSPLQPLLPGFKRFSCLSLPNGMQF 120

>ref|NP_001137385.1| hypothetical protein LOC199870 isoform 2 [Homo sapiens]

Length=312

Score = 47.4 bits (111), Expect = 3e-06

Identities = 23/34 (68%), Positives = 27/34 (80%), Gaps = 0/34 (0%)

Frame = -1

Query 185 LEYSGAISADCNLHLPGSSNSSASASLVAGTIGM 84

LE +G ISA CNLHLPGSS+S AS+S VAG G+

Sbjct 50 LECNGTISAHCNLHLPGSSDSPASSSRVAGITGI 83

>ref|NP_001137384.1| hypothetical protein LOC199870 isoform 1 [Homo sapiens]

Length=341

Score = 47.4 bits (111), Expect = 3e-06

Identities = 23/34 (68%), Positives = 27/34 (80%), Gaps = 0/34 (0%)

Frame = -1

Query 185 LEYSGAISADCNLHLPGSSNSSASASLVAGTIGM 84

LE +G ISA CNLHLPGSS+S AS+S VAG G+

Sbjct 50 LECNGTISAHCNLHLPGSSDSPASSSRVAGITGI 83

>ref|NP_078841.3| cyclin-J-like protein [Homo sapiens]

Length=435

Score = 47.0 bits (110), Expect = 4e-06

Identities = 23/29 (80%), Positives = 24/29 (83%), Gaps = 0/29 (0%)

Frame = -1

Query 197 LSPSLEYSGAISADCNLHLPGSSNSSASA 111

LSP L+ SG ISA CNLHLPGSSNS ASA

Sbjct 99 LSPRLKCSGMISAHCNLHLPGSSNSPASA 127

>ref|NP_001108224.1| complement decay-accelerating factor isoform 2 precursor [Homo

sapiens]

Length=440

Score = 46.6 bits (109), Expect = 5e-06

Identities = 22/39 (57%), Positives = 26/39 (67%), Gaps = 0/39 (0%)

Frame = -3

Query 204 SHSVTQPGVQWCNLS*LQPPPPRFKQFFCLSLPSSWDYR 88

S VTQ G++WC+ S LQ P FK+ F SLPSSW YR

Sbjct 362 SRPVTQAGMRWCDRSSLQSRTPGFKRSFHFSLPSSWYYR 400

>ref|XP_003118848.1| PREDICTED: hypothetical protein LOC100130156 [Homo sapiens]

Length=147

Score = 46.2 bits (108), Expect = 6e-06

Identities = 22/35 (63%), Positives = 25/35 (72%), Gaps = 0/35 (0%)

Frame = -2

Query 217 F*DEVSLCHPAWSTVVQSQLTATSTSQVQAILLPQ 113

F + VSLC P WS V QLTAT+ S +QAILLPQ

Sbjct 109 FFERVSLCCPGWSAVAPPQLTATTASWIQAILLPQ 143

>ref|NP_714912.1| interleukin-12 receptor subunit beta-1 isoform 2 precursor [Homo

sapiens]

Length=381

Score = 46.2 bits (108), Expect = 6e-06

Identities = 24/40 (60%), Positives = 26/40 (65%), Gaps = 0/40 (0%)

Frame = -1

Query 173 GAISADCNLHLPGSSNSSASASLVAGTIGMHHHAWLIFVF 54

G ISA CNL LP S +S ASAS VAG G+ HH LI F

Sbjct 342 GMISAHCNLRLPDSRDSPASASRVAGITGICHHTRLILYF 381

>ref|XP_003119870.1| PREDICTED: hypothetical protein LOC100130156 [Homo sapiens]

ref|XP_003121031.1| PREDICTED: hypothetical protein LOC100130156 [Homo sapiens]

Length=147

Score = 46.2 bits (108), Expect = 6e-06

Identities = 22/35 (63%), Positives = 25/35 (72%), Gaps = 0/35 (0%)

Frame = -2

Query 217 F*DEVSLCHPAWSTVVQSQLTATSTSQVQAILLPQ 113

F + VSLC P WS V QLTAT+ S +QAILLPQ

Sbjct 109 FFERVSLCCPGWSAVAPPQLTATTASWIQAILLPQ 143

>ref|NP_001128626.1| zinc transporter ZIP14 isoform c [Homo sapiens]

Length=481

Score = 45.1 bits (105), Expect = 1e-05

Identities = 22/37 (60%), Positives = 26/37 (71%), Gaps = 0/37 (0%)

Frame = -3

Query 210 MKSHSVTQPGVQWCNLS*LQPPPPRFKQFFCLSLPSS 100

M+ SV Q GVQWC+LS LQP P K+ CLSLPS+

Sbjct 445 MEFCSVAQAGVQWCHLSSLQPLPLGLKRLSCLSLPSN 481

>ref|NP_001165113.1| myosin-IIIb isoform 3 [Homo sapiens]

Length=1275

Score = 44.7 bits (104), Expect = 2e-05

Identities = 25/42 (60%), Positives = 28/42 (67%), Gaps = 0/42 (0%)

Frame = -1

Query 206 SLTLSPSLEYSGAISADCNLHLPGSSNSSASASLVAGTIGMH 81

S TL LE + ISADCNL GSS+S ASAS VAG G+H

Sbjct 1194 SFTLLLRLECNSMISADCNLRPLGSSDSPASASRVAGITGIH 1235

>ref|NP_149084.2| tripartite motif-containing protein 5 isoform delta [Homo sapiens]

Length=326

Score = 44.7 bits (104), Expect = 2e-05

Identities = 19/26 (74%), Positives = 23/26 (89%), Gaps = 0/26 (0%)

Frame = -2

Query 184 WSTVVQSQLTATSTSQVQAILLPQPP 107

WS + +S+ TATSTSQ+QAILLPQPP

Sbjct 300 WSAMARSRFTATSTSQIQAILLPQPP 325

>ref|NP_612412.2| myosin regulatory light chain 10 [Homo sapiens]

Length=226

Score = 44.7 bits (104), Expect = 2e-05

Identities = 24/33 (73%), Positives = 25/33 (76%), Gaps = 0/33 (0%)

Frame = -1

Query 206 SLTLSPSLEYSGAISADCNLHLPGSSNSSASAS 108

SL LSP LE +G ISA CNL L GSSNS ASAS

Sbjct 58 SLALSPRLERNGMISAHCNLCLTGSSNSPASAS 90

>ref|NP_874362.3| ankyrin repeat and death domain-containing protein 1A [Homo sapiens]

Length=522

Score = 43.5 bits (101), Expect = 4e-05

Identities = 19/26 (74%), Positives = 23/26 (89%), Gaps = 0/26 (0%)

Frame = -2

Query 184 WSTVVQSQLTATSTSQVQAILLPQPP 107

WST+ +SQLTATS S+VQ IL+PQPP

Sbjct 496 WSTMARSQLTATSASRVQMILVPQPP 521

>ref|NP_001003690.1| MAD2L1-binding protein isoform 1 [Homo sapiens]

Length=306

Score = 43.5 bits (101), Expect = 4e-05

Identities = 24/41 (59%), Positives = 28/41 (69%), Gaps = 0/41 (0%)

Frame = -1

Query 206 SLTLSPSLEYSGAISADCNLHLPGSSNSSASASLVAGTIGM 84

SLTLSP LE++G SA N LPGS +S ASAS VA I +

Sbjct 9 SLTLSPRLEHNGMTSAHHNFRLPGSRDSPASASQVAEIIDL 49

>ref|NP_683685.1| 39S ribosomal protein L10, mitochondrial isoform b [Homo sapiens]

Length=271

Score = 42.7 bits (99), Expect = 7e-05

Identities = 21/27 (78%), Positives = 22/27 (82%), Gaps = 0/27 (0%)

Frame = -1

Query 167 ISADCNLHLPGSSNSSASASLVAGTIG 87

ISA CNLHLPGSS+S ASAS VAG G

Sbjct 2 ISAHCNLHLPGSSDSPASASQVAGITG 28

>ref|NP_001018114.1| fumarylacetoacetate hydrolase domain-containing protein 1 isoform

1 [Homo sapiens]

Length=248

Score = 42.4 bits (98), Expect = 9e-05

Identities = 21/35 (60%), Positives = 26/35 (75%), Gaps = 0/35 (0%)

Frame = -1

Query 215 LR*SLTLSPSLEYSGAISADCNLHLPGSSNSSASA 111

LR LTLSP LE S AI+A C+L LPGSSN +++

Sbjct 212 LRQGLTLSPKLECSSAITAHCSLELPGSSNPPSAS 246

>ref|NP_777547.1| intraflagellar transport protein 20 homolog [Homo sapiens]

Length=148

Score = 42.0 bits (97), Expect = 1e-04

Identities = 23/40 (58%), Positives = 26/40 (65%), Gaps = 0/40 (0%)

Frame = -1

Query 206 SLTLSPSLEYSGAISADCNLHLPGSSNSSASASLVAGTIG 87

SL +SP LE +GAISA C L L SS+S S S V GT G

Sbjct 72 SLAVSPRLECTGAISAHCKLCLSDSSDSPTSPSRVGGTTG 111

>ref|NP_001139736.1| synaptotagmin-14 isoform 2 [Homo sapiens]

Length=600

Score = 41.6 bits (96), Expect = 2e-04

Identities = 21/31 (68%), Positives = 23/31 (75%), Gaps = 0/31 (0%)

Frame = -1

Query 185 LEYSGAISADCNLHLPGSSNSSASASLVAGT 93

LEYSG I A CN L GS++SSASAS V GT

Sbjct 34 LEYSGTILAHCNFRLLGSNDSSASASQVTGT 64

>ref|NP_001139733.1| synaptotagmin-14 isoform 1 [Homo sapiens]

Length=619

Score = 41.6 bits (96), Expect = 2e-04

Identities = 21/31 (68%), Positives = 23/31 (75%), Gaps = 0/31 (0%)

Frame = -1

Query 185 LEYSGAISADCNLHLPGSSNSSASASLVAGT 93

LEYSG I A CN L GS++SSASAS V GT

Sbjct 34 LEYSGTILAHCNFRLLGSNDSSASASQVTGT 64

>ref|NP_060313.3| breast carcinoma-amplified sequence 4 isoform a [Homo sapiens]

Length=211

Score = 41.6 bits (96), Expect = 2e-04

Identities = 21/31 (68%), Positives = 24/31 (78%), Gaps = 0/31 (0%)

Frame = -1

Query 188 SLEYSGAISADCNLHLPGSSNSSASASLVAG 96

++E SG I A CNL LPGSS+S ASAS VAG

Sbjct 163 NVECSGTIPARCNLRLPGSSDSPASASQVAG 193

>ref|NP_001153583.1| nitric oxide synthase, endothelial isoform 4 [Homo sapiens]

Length=629

Score = 41.2 bits (95), Expect = 2e-04

Identities = 23/44 (53%), Positives = 26/44 (60%), Gaps = 0/44 (0%)

Frame = -1

Query 203 LTLSPSLEYSGAISADCNLHLPGSSNSSASASLVAGTIGMHHHA 72

LTL P LE S I+A C+L+L SSN S S V GT G H A

Sbjct 586 LTLWPRLECSSTITAHCSLNLLDSSNPPTSTSQVVGTTGACHDA 629

>ref|XP_938432.4| PREDICTED: hypothetical protein LOC646021 [Homo sapiens]

Length=359

Score = 40.8 bits (94), Expect = 3e-04

Identities = 21/30 (70%), Positives = 22/30 (74%), Gaps = 0/30 (0%)

Frame = -1

Query 197 LSPSLEYSGAISADCNLHLPGSSNSSASAS 108

LSP LE SG I A CNL LPG S+S ASAS

Sbjct 302 LSPRLECSGVILAHCNLRLPGLSDSPASAS 331

>ref|NP_001166173.1| probable sodium-coupled neutral amino acid transporter 6 isoform

1 [Homo sapiens]

Length=521

Score = 40.8 bits (94), Expect = 3e-04

Identities = 24/39 (62%), Positives = 24/39 (62%), Gaps = 0/39 (0%)

Frame = -1

Query 203 LTLSPSLEYSGAISADCNLHLPGSSNSSASASLVAGTIG 87

L LS L SG ISA CNL LP SSN SAS VA T G

Sbjct 432 LILSHRLACSGVISAHCNLCLPDSSNPPTSASRVAETTG 470

>ref|NP_001012677.1| arginine-fifty homeobox [Homo sapiens]

Length=315

Score = 40.8 bits (94), Expect = 3e-04

Identities = 21/38 (56%), Positives = 26/38 (69%), Gaps = 0/38 (0%)

Frame = -1

Query 206 SLTLSPSLEYSGAISADCNLHLPGSSNSSASASLVAGT 93

S TL LE SG +SA C+L+LPGS++ SAS VA T

Sbjct 35 SFTLLSKLECSGTVSAYCSLNLPGSTDPPTSASRVAAT 72

>ref|NP_000865.2| interferon alpha/beta receptor 2 isoform b precursor [Homo sapiens]

ref|NP_997467.1| interferon alpha/beta receptor 2 isoform b precursor [Homo sapiens]

Length=331

Score = 39.7 bits (91), Expect = 6e-04

Identities = 17/33 (52%), Positives = 20/33 (61%), Gaps = 0/33 (0%)

Frame = -3

Query 171 CNLS*LQPPPPRFKQFFCLSLPSSWDYRHAPSC 73

C+ + LQ P KQ CLS PSSWDY+ A C

Sbjct 296 CSHNALQSETPELKQSSCLSFPSSWDYKRASLC 328

>ref|NP_001158157.1| protein THEMIS isoform 1 [Homo sapiens]

Length=680

Score = 32.0 bits (71), Expect(2) = 7e-04

Identities = 14/20 (70%), Positives = 15/20 (75%), Gaps = 0/20 (0%)

Frame = -3

Query 183 GVQWCNLS*LQPPPPRFKQF 124

GVQW +L LQP PP FKQF

Sbjct 588 GVQWRDLGSLQPLPPGFKQF 607

Score = 26.6 bits (57), Expect(2) = 7e-04

Identities = 13/22 (60%), Positives = 13/22 (60%), Gaps = 0/22 (0%)

Frame = -1

Query 140 PGSSNSSASASLVAGTIGMHHH 75

PG SASAS VAG G HH

Sbjct 602 PGFKQFSASASHVAGITGTPHH 623

>ref|XP_003119888.1| PREDICTED: hypothetical protein LOC100508579 [Homo sapiens]

ref|XP_003121045.1| PREDICTED: hypothetical protein LOC100508579 [Homo sapiens]

Length=127

Score = 33.5 bits (75), Expect(2) = 0.001

Identities = 21/58 (37%), Positives = 27/58 (47%), Gaps = 0/58 (0%)

Frame = -1

Query 212 R*SLTLSPSLEYSGAISADCNLHLPGSSNSSASASLVAGTIGMHHHAWLIFVFLVEMG 39

R LTLS LE GAI+A + L GS N S +A + +H LI +G

Sbjct 61 RLGLTLSSRLECCGAITAQGSFDLMGSGNPFTSVDQIAEITNVGNHTLLISCIFCRVG 118

Score = 24.6 bits (52), Expect(2) = 0.001

Identities = 9/15 (60%), Positives = 10/15 (67%), Gaps = 0/15 (0%)

Frame = -3

Query 60 CIFSRDGISLCWPGW 16

CIF R G+S C GW

Sbjct 112 CIFCRVGVSPCCRGW 126

>ref|NP_001138525.1| EF-hand calcium-binding domain-containing protein 5 isoform b

[Homo sapiens]

Length=856

Score = 38.9 bits (89), Expect = 0.001

Identities = 19/34 (56%), Positives = 21/34 (62%), Gaps = 0/34 (0%)

Frame = -3

Query 189 QPGVQWCNLS*LQPPPPRFKQFFCLSLPSSWDYR 88

Q GVQW N S LQPP P K+ L S+WDYR

Sbjct 810 QAGVQWRNCSSLQPPTPGLKRSSHHRLLSNWDYR 843

>ref|NP_001098016.1| ribonuclease P protein subunit p30 isoform a [Homo sapiens]

Length=322

Score = 38.9 bits (89), Expect = 0.001

Identities = 21/33 (64%), Positives = 21/33 (64%), Gaps = 0/33 (0%)

Frame = -3

Query 204 SHSVTQPGVQWCNLS*LQPPPPRFKQFFCLSLP 106

SHSVTQ GVQW NL LQP P K LSLP

Sbjct 267 SHSVTQAGVQWHNLGSLQPLPLGLKPSSHLSLP 299

>ref|NP_001129224.1| protein SGT1 isoform 2 [Homo sapiens]

Length=677

Score = 38.5 bits (88), Expect = 0.001

Identities = 17/29 (59%), Positives = 19/29 (66%), Gaps = 0/29 (0%)

Frame = -3

Query 180 VQWCNLS*LQPPPPRFKQFFCLSLPSSWD 94

VQW + LQ PPP F F CLSL S+WD

Sbjct 379 VQWRDPGLLQAPPPGFTPFICLSLLSTWD 407

>ref|NP_001030127.1| sorbin and SH3 domain-containing protein 1 isoform 4 [Homo sapiens]

Length=1151

Score = 38.1 bits (87), Expect = 0.002

Identities = 20/35 (58%), Positives = 23/35 (66%), Gaps = 0/35 (0%)

Frame = -1

Query 197 LSPSLEYSGAISADCNLHLPGSSNSSASASLVAGT 93

+SP LE SG + A C+L L SSN SAS VAGT

Sbjct 440 MSPRLECSGTVIAHCSLKLLDSSNPPTSASQVAGT 474

>ref|NP_872321.2| zinc finger protein 714 [Homo sapiens]

Length=555

Score = 37.0 bits (84), Expect = 0.004

Identities = 17/23 (74%), Positives = 18/23 (79%), Gaps = 0/23 (0%)

Frame = +2

Query 2 SGVQYHPGQHSEIPSLLKIQKLA 70

SGVQ PGQH + PSLLKIQK A

Sbjct 526 SGVQDQPGQHGKTPSLLKIQKFA 548

>ref|XP_003119512.1| PREDICTED: hypothetical protein LOC100507663 [Homo sapiens]

ref|XP_003118669.1| PREDICTED: hypothetical protein LOC100507663 [Homo sapiens]

ref|XP_003120701.1| PREDICTED: hypothetical protein LOC100507663 [Homo sapiens]

Length=124

Score = 37.0 bits (84), Expect = 0.004

Identities = 16/23 (70%), Positives = 19/23 (83%), Gaps = 0/23 (0%)

Frame = +1

Query 1 VRSSIPPWPT**NPISTKNTKIS 69

VRSS P WPT NP+STKNTK++

Sbjct 95 VRSSRPAWPTLRNPVSTKNTKLA 117

>ref|NP_062553.1| putative uncharacterized protein C8orf44 [Homo sapiens]

Length=159

Score = 37.0 bits (84), Expect = 0.004

Identities = 18/23 (79%), Positives = 18/23 (79%), Gaps = 0/23 (0%)

Frame = +1

Query 1 VRSSIPPWPT**NPISTKNTKIS 69

VRSS P WPT NPI TKNTKIS

Sbjct 59 VRSSKPAWPTWRNPIFTKNTKIS 81

>ref|XP_002343910.2| PREDICTED: testis-specific Y-encoded protein 2-like [Homo sapiens]

Length=140

Score = 36.6 bits (83), Expect = 0.005

Identities = 17/28 (61%), Positives = 20/28 (72%), Gaps = 0/28 (0%)

Frame = -3

Query 213 EMKSHSVTQPGVQWCNLS*LQPPPPRFK 130

EM+SH VTQ GV+W +L LQ PP FK

Sbjct 106 EMESHYVTQAGVKWHDLGSLQTLPPSFK 133

>ref|NP_065184.2| selenoprotein N isoform 1 precursor [Homo sapiens]

Length=590

Score = 36.6 bits (83), Expect = 0.005

Identities = 19/32 (60%), Positives = 20/32 (63%), Gaps = 0/32 (0%)

Frame = -3

Query 204 SHSVTQPGVQWCNLS*LQPPPPRFKQFFCLSL 109

S SVTQ GVQWC+ S LQP P CLSL

Sbjct 102 SCSVTQTGVQWCSHSSLQPQLPWLNUSSCLSL 133

>ref|NP_057728.1| proline-rich protein 16 [Homo sapiens]

Length=281

Score = 36.6 bits (83), Expect = 0.005

Identities = 18/22 (82%), Positives = 19/22 (87%), Gaps = 0/22 (0%)

Frame = -2

Query 175 VVQSQLTATSTSQVQAILLPQP 110

+ QS LTATS SQVQAILLPQP

Sbjct 1 MAQSGLTATSASQVQAILLPQP 22

>ref|NP_660326.2| nucleoredoxin-like protein 2 isoform 2 [Homo sapiens]

Length=135

Score = 36.2 bits (82), Expect = 0.007

Identities = 21/33 (64%), Positives = 22/33 (67%), Gaps = 0/33 (0%)

Frame = -1

Query 206 SLTLSPSLEYSGAISADCNLHLPGSSNSSASAS 108

SL L P LE SG I A CNL L GSS+S A AS

Sbjct 103 SLALLPRLECSGVILAHCNLCLLGSSDSLALAS 135

Query= Simvastatin Contig 13

Length=136

Score E

Sequences producing significant alignments: (Bits) Value

ref|NP_001002.1| 40S ribosomal protein S7 [Homo sapiens] 65.9 8e-12

ALIGNMENTS

>ref|NP_001002.1| 40S ribosomal protein S7 [Homo sapiens]

Length=194

Score = 65.9 bits (159), Expect = 8e-12

Identities = 30/30 (100%), Positives = 30/30 (100%), Gaps = 0/30 (0%)

Frame = +2

Query 2 NVEHKVETFSGVYKKLTGKDVNFEFPEFQL 91

NVEHKVETFSGVYKKLTGKDVNFEFPEFQL

Sbjct 165 NVEHKVETFSGVYKKLTGKDVNFEFPEFQL 194

Query= Simvastatin Contig 14

Length=387

Score E

Sequences producing significant alignments: (Bits) Value

ref|NP_689672.4| hypothetical protein LOC146556 isoform 1 pre... 104 1e-23

ref|XP_003119925.1| PREDICTED: histone demethylase UTY-like [... 104 2e-23

ref|XP_003118557.1| PREDICTED: histone demethylase UTY-like [... 104 2e-23

ref|XP_003119248.1| PREDICTED: hypothetical protein LOC100506... 97.4 2e-21

ref|NP_001158011.1| disrupted in schizophrenia 1 protein isof... 95.1 1e-20

ref|NP_872601.1| histone demethylase UTY isoform 1 [Homo sapi... 89.0 9e-19

ref|XP_003118843.1| PREDICTED: zinc finger protein ENSP000003... 50.1 1e-17

ref|XP_003119043.1| PREDICTED: hypothetical protein LOC100506... 65.9 5e-15

ref|XP_003121047.1| PREDICTED: KN motif and ankyrin repeat do... 56.2 9e-15

ref|XP_003120111.1| PREDICTED: putative uncharacterized prote... 54.3 2e-14

ref|NP_001136036.1| cGMP-gated cation channel alpha-1 isoform... 57.8 3e-14

ref|XP_003119819.1| PREDICTED: putative uncharacterized prote... 58.5 3e-14

ref|XP_003119846.1| PREDICTED: hypothetical protein LOC100507... 46.6 1e-13

ref|XP_003119989.1| PREDICTED: hypothetical protein LOC100508... 63.9 3e-13

ref|XP_003119512.1| PREDICTED: hypothetical protein LOC100507... 70.5 3e-13

ref|XP_003120115.1| PREDICTED: putative uncharacterized prote... 67.8 2e-12

ref|NP_062553.1| putative uncharacterized protein C8orf44 [Ho... 67.8 2e-12

ref|NP_001164252.1| hypothetical protein LOC159091 isoform 4 ... 57.8 3e-12

ref|XP_003119960.1| PREDICTED: putative uncharacterized prote... 63.5 4e-11

ref|XP_003118634.1| PREDICTED: putative uncharacterized prote... 62.4 9e-11

ref|XP_003120094.1| PREDICTED: putative uncharacterized prote... 62.4 9e-11

ref|XP_003119834.1| PREDICTED: zinc finger protein ENSP000003... 45.4 1e-10

ref|XP_938432.4| PREDICTED: hypothetical protein LOC646021 [H... 60.5 3e-10

ref|NP_001158312.1| LYR motif-containing protein 4 isoform 2 ... 59.3 7e-10

ref|XP_003119895.1| PREDICTED: uncharacterized protein FLJ395... 57.4 3e-09

ref|NP_150646.3| alpha-1A adrenergic receptor isoform 2 [Homo... 56.6 5e-09

ref|NP_060190.2| signal-transducing adaptor protein 2 isoform... 56.2 6e-09

ref|NP_009112.1| mitogen-activated protein kinase kinase kina... 54.3 2e-08

ref|NP_078926.3| putative uncharacterized protein C11orf80 [H... 53.9 3e-08

ref|XP_003120008.1| PREDICTED: putative uncharacterized prote... 53.5 4e-08

ref|XP_003119783.1| PREDICTED: serine/threonine-protein phosp... 53.5 4e-08

ref|NP_001180462.1| serine/threonine-protein kinase Nek4 isof... 53.1 5e-08

ref|NP_003148.2| serine/threonine-protein kinase Nek4 isoform... 53.1 5e-08

ref|XP_003119948.1| PREDICTED: hypothetical protein LOC100508... 46.2 9e-08

ref|XP_003120664.1| PREDICTED: hypothetical protein LOC100510... 52.0 1e-07

ref|XP_003120124.1| PREDICTED: hypothetical protein LOC100507... 52.0 1e-07

ref|NP_862828.1| zinc finger protein 283 [Homo sapiens] 52.0 1e-07

ref|XP_003119509.1| PREDICTED: hypothetical protein LOC100507... 52.0 1e-07

ref|NP_001137385.1| hypothetical protein LOC199870 isoform 2 ... 51.6 2e-07

ref|NP_001137384.1| hypothetical protein LOC199870 isoform 1 ... 51.6 2e-07

ref|NP_115861.1| peptidyl-prolyl cis-trans isomerase-like 3 i... 51.6 2e-07

ref|NP_056087.2| protein fantom isoform a [Homo sapiens] 51.2 2e-07

ref|NP_078841.3| cyclin-J-like protein [Homo sapiens] 51.2 2e-07

ref|NP_001012680.1| 4F2 cell-surface antigen heavy chain isof... 50.4 3e-07

ref|XP_002346169.1| PREDICTED: protein MOST-1-like [Homo sapi... 50.4 3e-07

ref|XP_003120114.1| PREDICTED: putative uncharacterized prote... 49.3 3e-07

ref|NP_001077368.1| platelet glycoprotein VI isoform 1 [Homo ... 41.6 4e-07

ref|NP_714912.1| interleukin-12 receptor subunit beta-1 isofo... 50.1 4e-07

ref|NP_963998.2| thromboxane A2 receptor isoform beta [Homo s... 50.1 4e-07

ref|NP_001011657.2| zinc finger matrin-type protein 1 isoform... 49.7 6e-07

ref|NP_001153585.1| hypothetical protein LOC123207 isoform b ... 45.4 7e-07

ref|NP_001155002.1| granulocyte-macrophage colony-stimulating... 49.3 7e-07

ref|XP_003119710.1| PREDICTED: hypothetical protein LOC100507... 48.9 1e-06

ref|XP_003119678.1| PREDICTED: hypothetical protein LOC100506... 48.9 1e-06

ref|XP_003118780.1| PREDICTED: hypothetical protein LOC100507... 48.9 1e-06

ref|NP_001158157.1| protein THEMIS isoform 1 [Homo sapiens] 45.4 1e-06

ref|XP_003119968.1| PREDICTED: hypothetical protein LOC100129... 48.5 1e-06

ref|NP_001158009.1| disrupted in schizophrenia 1 protein isof... 48.5 1e-06

ref|NP_001185728.1| activating signal cointegrator 1 complex ... 48.5 1e-06

ref|NP_060313.3| breast carcinoma-amplified sequence 4 isofor... 48.1 2e-06

ref|NP_001166173.1| probable sodium-coupled neutral amino aci... 47.8 2e-06

ref|NP_001177143.1| inositol hexakisphosphate and diphosphoin... 47.8 2e-06

ref|NP_001124384.1| suppressor of G2 allele of SKP1 homolog i... 47.8 2e-06

ref|NP_777603.1| hypothetical protein LOC283579 isoform 1 [Ho... 47.8 2e-06

ref|XP_003119709.1| PREDICTED: hypothetical protein LOC100509... 36.2 3e-06

ref|NP_001123992.1| zinc finger protein 195 isoform 1 [Homo s... 47.4 3e-06

ref|NP_001123991.1| zinc finger protein 195 isoform 2 [Homo s... 47.4 3e-06

ref|NP_789795.1| sulfotransferase 1C2 isoform b [Homo sapiens] 47.0 4e-06

ref|NP_001166126.1| zinc finger protein 701 isoform 1 [Homo s... 46.6 5e-06

ref|NP_002899.1| proto-oncogene c-Rel [Homo sapiens] 46.2 6e-06

ref|XP_003119048.1| PREDICTED: hypothetical protein LOC100506... 45.8 8e-06

ref|NP_001167449.1| hypothetical protein LOC283579 isoform 2 ... 45.8 8e-06

ref|NP_660344.2| hypothetical protein LOC201158 isoform 1 [Ho... 45.8 8e-06

ref|NP_001030127.1| sorbin and SH3 domain-containing protein ... 45.8 8e-06

ref|XP_003120922.1| PREDICTED: putative uncharacterized prote... 45.4 1e-05

ref|XP_003119972.1| PREDICTED: hypothetical protein LOC100508... 45.4 1e-05

ref|NP_001003690.1| MAD2L1-binding protein isoform 1 [Homo sa... 45.1 1e-05

ref|XP_003119551.1| PREDICTED: hypothetical protein LOC100505... 45.1 1e-05

ref|XP_003119888.1| PREDICTED: hypothetical protein LOC100508... 36.2 2e-05

ref|NP_001138489.1| proton-coupled amino acid transporter 3 i... 44.7 2e-05

ref|NP_689573.3| zinc finger protein 573 isoform 1 [Homo sapi... 44.3 2e-05

ref|NP_955751.1| putative potassium channel regulatory protei... 44.3 2e-05

ref|NP_001153587.1| hypothetical protein LOC123207 isoform d ... 43.9 3e-05

ref|NP_001153583.1| nitric oxide synthase, endothelial isofor... 43.9 3e-05

ref|NP_001005735.1| serine/threonine-protein kinase Chk2 isof... 43.9 3e-05

ref|NP_001129224.1| protein SGT1 isoform 2 [Homo sapiens] 43.5 4e-05

ref|NP_006668.1| ubiquitin carboxyl-terminal hydrolase 19 iso... 43.5 4e-05

ref|NP_114174.1| nuclear prelamin A recognition factor isofor... 43.1 5e-05

ref|NP_001171696.1| BEN domain-containing protein 2 isoform 2... 42.7 7e-05

ref|NP_001180442.1| zinc transporter 6 isoform 1 [Homo sapiens] 42.7 7e-05

ref|NP_116326.2| chromosome 9 open reading frame 37 [Homo sap... 42.7 7e-05

ref|NP_777547.1| intraflagellar transport protein 20 homolog ... 42.7 7e-05

ref|NP_683685.1| 39S ribosomal protein L10, mitochondrial iso... 42.7 7e-05

ref|NP_699177.2| BEN domain-containing protein 2 isoform 1 [H... 42.7 7e-05

ref|NP_872321.2| zinc finger protein 714 [Homo sapiens] 36.2 9e-05

ref|NP_001165113.1| myosin-IIIb isoform 3 [Homo sapiens] 42.4 9e-05

ref|NP_001012677.1| arginine-fifty homeobox [Homo sapiens] 42.4 9e-05

ref|XP_002346405.2| PREDICTED: hypothetical protein LOC100294... 42.0 1e-04

ref|NP_597994.3| DNA repair protein RAD51 homolog 1 isoform 2... 41.2 2e-04

ref|NP_612412.2| myosin regulatory light chain 10 [Homo sapiens] 41.2 2e-04

ALIGNMENTS

>ref|NP_689672.4| hypothetical protein LOC146556 isoform 1 precursor [Homo sapiens]

Length=402

Score = 104 bits (260), Expect = 1e-23

Identities = 52/78 (67%), Positives = 56/78 (72%), Gaps = 0/78 (0%)

Frame = -2

Query 356 KAGVQWRNLGSLQPPPPEFTPFSCLSLPSS*DYRRPPACLALFFVFFVEIGFRHVAQAGL 177

+AGVQWRNLGSLQP PP F FSCL LPSS DYR P LA F++F VE GF HVA AGL

Sbjct 325 QAGVQWRNLGSLQPLPPGFKQFSCLILPSSWDYRSVPPYLANFYIFLVETGFHHVAHAGL 384

Query 176 GFLNSSDPPASASQSAGI 123

L S DPP S SQS G+

Sbjct 385 ELLISRDPPTSGSQSVGL 402

>ref|XP_003119925.1| PREDICTED: histone demethylase UTY-like [Homo sapiens]

ref|XP_003120437.1| PREDICTED: histone demethylase UTY-like [Homo sapiens]

Length=101

Score = 104 bits (259), Expect = 2e-23

Identities = 55/82 (68%), Positives = 58/82 (71%), Gaps = 1/82 (1%)

Frame = -2

Query 359 PKAGVQWRNLGSLQPPPPEFTPFSCLSLPSS*DYRRPPACLALFFVFFVEIGFRHVAQAG 180

P AGVQW NL SLQPPP F PFS LSL S D RRP CL + FVF +E FRHV QAG

Sbjct 11 PHAGVQWHNLSSLQPPPSGFKPFSYLSLLRSRDQRRPLPCL-VTFVFLIETRFRHVGQAG 69

Query 179 LGFLNSSDPPASASQSAGITGI 114

L L S DPPASASQSAGI G+

Sbjct 70 LKLLTSGDPPASASQSAGIRGV 91

>ref|XP_003118557.1| PREDICTED: histone demethylase UTY-like [Homo sapiens]

Length=101

Score = 104 bits (259), Expect = 2e-23

Identities = 55/82 (68%), Positives = 58/82 (71%), Gaps = 1/82 (1%)

Frame = -2

Query 359 PKAGVQWRNLGSLQPPPPEFTPFSCLSLPSS*DYRRPPACLALFFVFFVEIGFRHVAQAG 180

P AGVQW NL SLQPPP F PFS LSL SS D RRP CL + FVF +E F HV QAG

Sbjct 11 PHAGVQWHNLSSLQPPPSGFKPFSYLSLLSSRDQRRPLPCL-VTFVFLIETRFHHVGQAG 69

Query 179 LGFLNSSDPPASASQSAGITGI 114

L L S DPPASASQSAGI G+

Sbjct 70 LKLLTSGDPPASASQSAGIRGV 91

>ref|XP_003119248.1| PREDICTED: hypothetical protein LOC100506511 [Homo sapiens]

Length=402

Score = 97.4 bits (241), Expect = 2e-21

Identities = 56/90 (63%), Positives = 63/90 (70%), Gaps = 1/90 (1%)

Frame = -2

Query 383 LRQSLALSPKAGVQWRNLGSLQPPPPEFTPFSCLSLPSS*DYRRPPACLALFFVFFVEIG 204

LR+S AL+P+AGV+ R+L SLQP PP F FSCLSLPSS Y P+ A FV VE

Sbjct 299 LRRSFALAPQAGVRCRDLDSLQPLPPGFKRFSCLSLPSSWGYSHAPSRPA-NFVVLVETV 357

Query 203 FRHVAQAGLGFLNSSDPPASASQSAGITGI 114

F HV QAGL L S DPP SASQSAGITG+

Sbjct 358 FLHVDQAGLELLISGDPPTSASQSAGITGV 387

>ref|NP_001158011.1| disrupted in schizophrenia 1 protein isoform c [Homo sapiens]

Length=755

Score = 95.1 bits (235), Expect = 1e-20

Identities = 51/69 (74%), Positives = 53/69 (77%), Gaps = 1/69 (1%)

Frame = -2

Query 326 SLQPPPPEFTPFSCLSLPSS*DYRRPPACLALFFVFFVEIGFRHVAQAGLGFLNSSDPPA 147

SLQP PPEF FSCLSL SS DYR PP CLA F VF VE+GF HV Q GL L SSDPP+

Sbjct 678 SLQPLPPEFKQFSCLSLRSSWDYRCPPPCLANF-VFLVEMGFYHVDQTGLKLLTSSDPPS 736

Query 146 SASQSAGIT 120

SASQSAGIT

Sbjct 737 SASQSAGIT 745

>ref|NP_872601.1| histone demethylase UTY isoform 1 [Homo sapiens]

Length=1079

Score = 89.0 bits (219), Expect = 9e-19

Identities = 49/87 (57%), Positives = 60/87 (69%), Gaps = 1/87 (1%)

Frame = -2

Query 362 SPKAGVQWRNLGSLQPPPPEFTPFSCLSLPSS*DYRRPPACLALFFVFFVEIGFRHVAQA 183

S +AG+QW +L SLQPPPP F FS LSLP+S +YR P+C F + FVE GF HV QA

Sbjct 993 SLRAGMQWCDLSSLQPPPPGFKRFSHLSLPNSWNYRHLPSCPTNFCI-FVETGFHHVGQA 1051

Query 182 GLGFLNSSDPPASASQSAGITGIRNNS 102

L L S ASASQSAGITG+ +++

Sbjct 1052 CLELLTSGGLLASASQSAGITGVSHHA 1078

>ref|XP_003118843.1| PREDICTED: zinc finger protein ENSP00000375192-like [Homo sapiens]

ref|XP_003120983.1| PREDICTED: zinc finger protein ENSP00000375192-like [Homo sapiens]

Length=245

Score = 50.1 bits (118), Expect(3) = 1e-17

Identities = 25/48 (53%), Positives = 30/48 (63%), Gaps = 0/48 (0%)

Frame = -3

Query 358 PRLECSGVIWAHCNLRLPSSRHSPASASQVARITGARQHAWPYFLYFL 215

P+LEC+G I HCNLRL S S AS SQ A I GA HA F++ +

Sbjct 111 PKLECNGAISVHCNLRLLGSSDSLASTSQAAGIAGACHHAQLIFVFLV 158

Score = 48.1 bits (113), Expect(3) = 1e-17

Identities = 24/35 (69%), Positives = 24/35 (69%), Gaps = 0/35 (0%)

Frame = -2

Query 233 LFFVFFVEIGFRHVAQAGLGFLNSSDPPASASQSA 129

L FVF VE GF H QAG L SSDPPA ASQSA

Sbjct 152 LIFVFLVETGFHHFDQAGFELLTSSDPPALASQSA 186

Score = 27.3 bits (59), Expect(3) = 1e-17

Identities = 8/11 (73%), Positives = 10/11 (91%), Gaps = 0/11 (0%)

Frame = -1

Query 144 SLPKCWDYRHK 112

S PKCWDY+H+

Sbjct 185 SAPKCWDYKHE 195

>ref|XP_003119043.1| PREDICTED: hypothetical protein LOC100506191 [Homo sapiens]

Length=118

Score = 65.9 bits (159), Expect(2) = 5e-15

Identities = 30/40 (75%), Positives = 35/40 (88%), Gaps = 0/40 (0%)

Frame = +1

Query 109 FLMPVIPALWEAEAGGSLEFRNPRPAWATWRNPISTKNTK 228

+L+PVI AL EA+AGGSLE R+ RPAW TWRNP+STKNTK

Sbjct 48 WLVPVILALGEAKAGGSLEARSSRPAWPTWRNPVSTKNTK 87

Score = 30.8 bits (68), Expect(2) = 5e-15

Identities = 17/33 (52%), Positives = 18/33 (55%), Gaps = 2/33 (6%)

Frame = +3

Query 219 KYKK*GQACWRAPVILATWEAEAGEWRELGRRR 317

K K QA W P+I ATWE E G LG RR

Sbjct 84 KNTKISQAWWSMPMISATWETEVG--GSLGPRR 114

>ref|XP_003121047.1| PREDICTED: KN motif and ankyrin repeat domain-containing protein

3-like [Homo sapiens]

Length=143

Score = 56.2 bits (134), Expect(2) = 9e-15

Identities = 27/46 (59%), Positives = 32/46 (70%), Gaps = 0/46 (0%)

Frame = -2

Query 356 KAGVQWRNLGSLQPPPPEFTPFSCLSLPSS*DYRRPPACLALFFVF 219

+AGV+W +LGSLQ PPP F FS LSL SS D+R P C A F +F

Sbjct 79 QAGVRWHDLGSLQSPPPRFKQFSYLSLLSSWDHRHTPPCPANFCIF 124

Score = 39.7 bits (91), Expect(2) = 9e-15

Identities = 16/23 (70%), Positives = 17/23 (74%), Gaps = 0/23 (0%)

Frame = -1

Query 228 FCIFCRDRVSPCCPGWPWIPELK 160

FCIF RD VSPC PGW P+LK

Sbjct 121 FCIFSRDGVSPCWPGWSPTPDLK 143

>ref|XP_003120111.1| PREDICTED: putative uncharacterized protein NCRNA00269-like [Homo

sapiens]

ref|XP_003119110.1| PREDICTED: putative uncharacterized protein NCRNA00269-like [Homo

sapiens]

ref|XP_003118626.1| PREDICTED: putative uncharacterized protein NCRNA00269-like [Homo

sapiens]

ref|XP_003120652.1| PREDICTED: putative uncharacterized protein NCRNA00269-like [Homo

sapiens]

Length=130

Score = 54.3 bits (129), Expect(2) = 2e-14

Identities = 26/40 (65%), Positives = 30/40 (75%), Gaps = 0/40 (0%)

Frame = -2

Query 233 LFFVFFVEIGFRHVAQAGLGFLNSSDPPASASQSAGITGI 114

L FVF +++GF HV QAGL FL S D PA ASQSAGIT +

Sbjct 79 LIFVFVIKMGFHHVGQAGLEFLTSGDLPALASQSAGITDV 118

Score = 40.4 bits (93), Expect(2) = 2e-14

Identities = 21/48 (44%), Positives = 29/48 (61%), Gaps = 0/48 (0%)

Frame = -3

Query 358 PRLECSGVIWAHCNLRLPSSRHSPASASQVARITGARQHAWPYFLYFL 215

PRLEC G I AH +L LP R S AS SQ++ I R ++ F++ +

Sbjct 38 PRLECGGAILAHYSLCLPGLRDSLASVSQLSGIMCVRHYSRLIFVFVI 85

>ref|NP_001136036.1| cGMP-gated cation channel alpha-1 isoform 1 [Homo sapiens]

Length=759

Score = 57.8 bits (138), Expect(2) = 3e-14

Identities = 33/58 (57%), Positives = 38/58 (66%), Gaps = 7/58 (12%)

Frame = -2

Query 284 LSLPSS*DYRRPPACLALFFVFFVEIGFRHVAQAGLGFLNSSDPPASASQSAGITGIR 111

L LP S D++ L FVF VE+GF HV QAGL L SSD P SASQSAGIT ++

Sbjct 21 LHLPDSSDFQ-------LIFVFLVEMGFHHVGQAGLELLISSDLPTSASQSAGITDMK 71

Score = 36.2 bits (82), Expect(2) = 3e-14

Identities = 15/20 (75%), Positives = 16/20 (80%), Gaps = 0/20 (0%)

Frame = -3

Query 358 PRLECSGVIWAHCNLRLPSS 299

PRLECSG I AHC+L LP S

Sbjct 7 PRLECSGAISAHCSLHLPDS 26

>ref|XP_003119819.1| PREDICTED: putative uncharacterized protein C14orf165-like [Homo

sapiens]

ref|XP_003118891.1| PREDICTED: putative uncharacterized protein C14orf165-like [Homo

sapiens]

ref|XP_003120627.1| PREDICTED: putative uncharacterized protein C14orf165-like [Homo

sapiens]

Length=110

Score = 58.5 bits (140), Expect(2) = 3e-14

Identities = 29/56 (52%), Positives = 34/56 (61%), Gaps = 0/56 (0%)

Frame = -2

Query 386 FLRQSLALSPKAGVQWRNLGSLQPPPPEFTPFSCLSLPSS*DYRRPPACLALFFVF 219

FL + +AG+QW +L SLQP P F FSCLSLPSS DYR P L F +F

Sbjct 36 FLEMEFCSAAQAGMQWLSLSSLQPLHPRFKQFSCLSLPSSCDYRHVPPHLVNFCIF 91

Score = 35.4 bits (80), Expect(2) = 3e-14

Identities = 13/23 (57%), Positives = 16/23 (70%), Gaps = 0/23 (0%)

Frame = -1

Query 228 FCIFCRDRVSPCCPGWPWIPELK 160

FCIF RD+V PC PGW +L+

Sbjct 88 FCIFSRDKVLPCWPGWSQTSDLR 110

>ref|XP_003119846.1| PREDICTED: hypothetical protein LOC100507929 [Homo sapiens]

ref|XP_003119023.1| PREDICTED: hypothetical protein LOC100507664 [Homo sapiens]

ref|XP_003120394.1| PREDICTED: hypothetical protein LOC100507929 [Homo sapiens]

Length=122

Score = 46.6 bits (109), Expect(2) = 1e-13

Identities = 23/38 (61%), Positives = 25/38 (66%), Gaps = 0/38 (0%)

Frame = +3

Query 246 WRAPVILATWEAEAGEWRELGRRRLQ*AQITPLHSSLG 359

W+ PVI TWEAEAGE E G+ LQ I LHSSLG

Sbjct 61 WQVPVIPTTWEAEAGESLEPGKSSLQRTMILTLHSSLG 98

Score = 45.4 bits (106), Expect(2) = 1e-13

Identities = 24/40 (60%), Positives = 27/40 (68%), Gaps = 0/40 (0%)

Frame = +1

Query 109 FLMPVIPALWEAEAGGSLEFRNPRPAWATWRNPISTKNTK 228

+L PVI WEA AG LE R+PR A AT +N STKNTK

Sbjct 16 WLTPVIQVFWEAGAGVLLEPRSPRSACATRQNSTSTKNTK 55

>ref|XP_003119989.1| PREDICTED: hypothetical protein LOC100508022 [Homo sapiens]

ref|XP_003118579.1| PREDICTED: hypothetical protein LOC100506688 [Homo sapiens]

ref|XP_003120502.1| PREDICTED: hypothetical protein LOC100508022 [Homo sapiens]

Length=176

Score = 63.9 bits (154), Expect(2) = 3e-13

Identities = 31/49 (64%), Positives = 36/49 (74%), Gaps = 2/49 (4%)

Frame = -2

Query 356 KAGVQWRNLGSLQPPPPEFTPFSCLSLPSS*DYRRPPACLA--LFFVFF 216

+AGVQW NL SLQPPPP F FSCLS PSS +YR P C A L+F++F

Sbjct 70 QAGVQWCNLSSLQPPPPWFKQFSCLSFPSSWNYRHLPPCPANFLYFLYF 118

Score = 26.9 bits (58), Expect(2) = 3e-13

Identities = 11/19 (58%), Positives = 13/19 (69%), Gaps = 0/19 (0%)

Frame = -1

Query 174 IPELK*SSCLSLPKCWDYR 118

+P SSCLSLP W+YR

Sbjct 146 LPGSSNSSCLSLPSRWNYR 164

>ref|XP_003119512.1| PREDICTED: hypothetical protein LOC100507663 [Homo sapiens]

ref|XP_003118669.1| PREDICTED: hypothetical protein LOC100507663 [Homo sapiens]

ref|XP_003120701.1| PREDICTED: hypothetical protein LOC100507663 [Homo sapiens]

Length=124

Score = 70.5 bits (171), Expect = 3e-13

Identities = 35/50 (70%), Positives = 38/50 (76%), Gaps = 1/50 (2%)

Frame = +1

Query 109 FLMPVIPALWEAEAGGSLEFRNPRPAWATWRNPISTKNTKNKARHAGGRL 258

+L PVIPALWEAE GGS E R+ RPAW T RNP+STKNTK ARH G L

Sbjct 76 WLTPVIPALWEAEVGGSPEVRSSRPAWPTLRNPVSTKNTK-LARHGGVHL 124

>ref|XP_003120115.1| PREDICTED: putative uncharacterized protein NCRNA00269-like [Homo

sapiens]

ref|XP_003119111.1| PREDICTED: putative uncharacterized protein NCRNA00269-like [Homo

sapiens]

ref|XP_003118613.1| PREDICTED: putative uncharacterized protein NCRNA00269-like [Homo

sapiens]

ref|XP_003120653.1| PREDICTED: putative uncharacterized protein NCRNA00269-like [Homo

sapiens]

Length=137

Score = 67.8 bits (164), Expect = 2e-12

Identities = 36/60 (60%), Positives = 39/60 (65%), Gaps = 0/60 (0%)

Frame = -3

Query 355 RLECSGVIWAHCNLRLPSSRHSPASASQVARITGARQHAWPYFLYFL*R*GFAMLPRLAL 176

RLECSG I AHCNLRL S PASAS+VA ITGA HAW FL FL GF + + L

Sbjct 37 RLECSGAILAHCNLRLLGSNEPPASASRVAGITGACHHAWLIFLVFLVEMGFRHIGQAGL 96

Score = 58.2 bits (139), Expect = 2e-09

Identities = 43/106 (41%), Positives = 52/106 (50%), Gaps = 22/106 (20%)

Frame = -2

Query 386 FLRQSLALSPK--------AGVQWRNLGSLQPPPPEFTPFSCLSLPSS*DYRRPPAC--- 240

FLR SL LS + A R LGS +PP AC

Sbjct 27 FLRWSLTLSSRLECSGAILAHCNLRLLGSNEPPASASRVAGITG-----------ACHHA 75

Query 239 LALFFVFFVEIGFRHVAQAGLGFLNSSDPPASASQSAGITGIRNNS 102

+F VF VE+GFRH+ QAGL L SSD P SASQS GITG+ +++

Sbjct 76 WLIFLVFLVEMGFRHIGQAGLKLLASSDVPISASQSVGITGMSHHA 121

>ref|NP_062553.1| putative uncharacterized protein C8orf44 [Homo sapiens]

Length=159

Score = 67.8 bits (164), Expect = 2e-12

Identities = 30/40 (75%), Positives = 34/40 (85%), Gaps = 0/40 (0%)

Frame = +1

Query 109 FLMPVIPALWEAEAGGSLEFRNPRPAWATWRNPISTKNTK 228

+LMPVIPALWEA+AG S E R+ +PAW TWRNPI TKNTK

Sbjct 40 WLMPVIPALWEAKAGRSPEVRSSKPAWPTWRNPIFTKNTK 79

>ref|NP_001164252.1| hypothetical protein LOC159091 isoform 4 [Homo sapiens]

Length=98

Score = 57.8 bits (138), Expect(2) = 3e-12

Identities = 29/47 (62%), Positives = 32/47 (69%), Gaps = 0/47 (0%)

Frame = -3

Query 358 PRLECSGVIWAHCNLRLPSSRHSPASASQVARITGARQHAWPYFLYF 218

PRLECSG+I AHCNL LP S SPAS S+VA +G R HA F F

Sbjct 44 PRLECSGMISAHCNLCLPGSSDSPASDSRVAGTSGTRHHAHLIFCIF 90

Score = 29.6 bits (65), Expect(2) = 3e-12

Identities = 12/14 (86%), Positives = 12/14 (86%), Gaps = 0/14 (0%)

Frame = -1

Query 234 LIFCIFCRDRVSPC 193

LIFCIF RD VSPC

Sbjct 85 LIFCIFSRDGVSPC 98

>ref|XP_003119960.1| PREDICTED: putative uncharacterized protein NCRNA00269-like [Homo

sapiens]

ref|XP_003118569.1| PREDICTED: putative uncharacterized protein NCRNA00269-like [Homo

sapiens]

ref|XP_003120465.1| PREDICTED: putative uncharacterized protein NCRNA00269-like [Homo

sapiens]

Length=128

Score = 63.5 bits (153), Expect = 4e-11

Identities = 44/103 (43%), Positives = 51/103 (50%), Gaps = 17/103 (16%)

Frame = -2

Query 386 FLRQSLALSPK--------AGVQWRNLGSLQPPPPEFTPFSCLSLPSS*DYRRPPACLAL 231

FLR+SLALSP+ A R LGS +S S L

Sbjct 23 FLRRSLALSPRLECSGAISAHCNLRLLGS---------SYSLASASRVSGITGSRHHAQL 73

Query 230 FFVFFVEIGFRHVAQAGLGFLNSSDPPASASQSAGITGIRNNS 102

FFVF VE GF H+ QAGL L S DPP SASQS GITG+ + +

Sbjct 74 FFVFLVETGFHHIGQAGLELLTSGDPPTSASQSVGITGVSHRA 116

>ref|XP_003118634.1| PREDICTED: putative uncharacterized protein NCRNA00269-like [Homo

sapiens]

ref|XP_003120632.1| PREDICTED: putative uncharacterized protein NCRNA00269-like [Homo

sapiens]

Length=140

Score = 62.4 bits (150), Expect = 9e-11

Identities = 29/48 (61%), Positives = 33/48 (69%), Gaps = 0/48 (0%)

Frame = -3

Query 358 PRLECSGVIWAHCNLRLPSSRHSPASASQVARITGARQHAWPYFLYFL 215

P+LEC G I AHCNL LP S PASASQVA TGA HAW F++ +

Sbjct 34 PKLECHGTISAHCNLHLPGSSDFPASASQVAGTTGACHHAWLIFVFLV 81

Score = 59.7 bits (143), Expect = 6e-10

Identities = 43/104 (42%), Positives = 53/104 (51%), Gaps = 21/104 (20%)

Frame = -2

Query 383 LRQSLALSPKAGVQWRNLGSLQPPPPEFTPFSCLSLPSS*DYRRPPACLA---------- 234

LRQSLAL PK G++ + L LP S D+ + +A

Sbjct 26 LRQSLALLPKLECH----GTI-------SAHCNLHLPGSSDFPASASQVAGTTGACHHAW 74

Query 233 LFFVFFVEIGFRHVAQAGLGFLNSSDPPASASQSAGITGIRNNS 102

L FVF VE GF HV Q GL L S+DPP ASQSAGITG+ + +

Sbjct 75 LIFVFLVEAGFHHVGQDGLELLTSNDPPTLASQSAGITGVSHRA 118

>ref|XP_003120094.1| PREDICTED: putative uncharacterized protein NCRNA00269-like [Homo

sapiens]

ref|XP_003119097.1| PREDICTED: putative uncharacterized protein NCRNA00269-like [Homo

sapiens]

Length=140

Score = 62.4 bits (150), Expect = 9e-11

Identities = 29/48 (61%), Positives = 33/48 (69%), Gaps = 0/48 (0%)

Frame = -3

Query 358 PRLECSGVIWAHCNLRLPSSRHSPASASQVARITGARQHAWPYFLYFL 215

P+LEC G I AHCNL LP S PASASQVA TGA HAW F++ +

Sbjct 34 PKLECHGTISAHCNLHLPGSSDFPASASQVAGTTGACHHAWLIFVFLV 81

Score = 59.7 bits (143), Expect = 6e-10

Identities = 43/104 (42%), Positives = 53/104 (51%), Gaps = 21/104 (20%)

Frame = -2

Query 383 LRQSLALSPKAGVQWRNLGSLQPPPPEFTPFSCLSLPSS*DYRRPPACLA---------- 234

LRQSLAL PK G++ + L LP S D+ + +A

Sbjct 26 LRQSLALLPKLECH----GTI-------SAHCNLHLPGSSDFPASASQVAGTTGACHHAW 74

Query 233 LFFVFFVEIGFRHVAQAGLGFLNSSDPPASASQSAGITGIRNNS 102

L FVF VE GF HV Q GL L S+DPP ASQSAGITG+ + +

Sbjct 75 LIFVFLVEAGFHHVGQDGLELLTSNDPPTLASQSAGITGVSHRA 118

>ref|XP_003119834.1| PREDICTED: zinc finger protein ENSP00000375192-like [Homo sapiens]

ref|XP_003119017.1| PREDICTED: zinc finger protein ENSP00000375192-like [Homo sapiens]

ref|XP_003120400.1| PREDICTED: zinc finger protein ENSP00000375192-like [Homo sapiens]

Length=105

Score = 45.4 bits (106), Expect(2) = 1e-10

Identities = 23/36 (64%), Positives = 24/36 (67%), Gaps = 0/36 (0%)

Frame = -2

Query 233 LFFVFFVEIGFRHVAQAGLGFLNSSDPPASASQSAG 126

L FVFF E GF HVA L L+SSDPP ASQS G

Sbjct 33 LIFVFFGETGFHHVAPLVLELLDSSDPPTLASQSGG 68

Score = 36.6 bits (83), Expect(2) = 1e-10

Identities = 18/37 (49%), Positives = 21/37 (57%), Gaps = 0/37 (0%)

Frame = -3

Query 328 AHCNLRLPSSRHSPASASQVARITGARQHAWPYFLYF 218

AHC+L L S P S SQVAR GA H F++F

Sbjct 2 AHCSLDLSGSGDPPTSTSQVARTIGACHHVQLIFVFF 38

>ref|XP_938432.4| PREDICTED: hypothetical protein LOC646021 [Homo sapiens]

Length=359

Score = 60.5 bits (145), Expect = 3e-10

Identities = 30/45 (67%), Positives = 33/45 (74%), Gaps = 0/45 (0%)

Frame = -3

Query 379 DRVSLCHPRLECSGVIWAHCNLRLPSSRHSPASASQVARITGARQ 245

D VSL PRLECSGVI AHCNLRLP SPASAS + R+T R+

Sbjct 297 DGVSLLSPRLECSGVILAHCNLRLPGLSDSPASASGIFRVTCCRR 341

>ref|NP_001158312.1| LYR motif-containing protein 4 isoform 2 [Homo sapiens]

Length=130

Score = 59.3 bits (142), Expect = 7e-10

Identities = 29/45 (65%), Positives = 32/45 (72%), Gaps = 0/45 (0%)

Frame = -2

Query 356 KAGVQWRNLGSLQPPPPEFTPFSCLSLPSS*DYRRPPACLALFFV 222

+AGV W +L SLQP PP F FSCLSLPSS DYRR P LA F +

Sbjct 77 QAGVHWNDLSSLQPLPPWFKQFSCLSLPSSWDYRRTPPRLANFCI 121

>ref|XP_003119895.1| PREDICTED: uncharacterized protein FLJ39582-like [Homo sapiens]

ref|XP_003118861.1| PREDICTED: uncharacterized protein FLJ39582-like [Homo sapiens]

ref|XP_003121056.1| PREDICTED: uncharacterized protein FLJ39582-like [Homo sapiens]

Length=122

Score = 57.4 bits (137), Expect = 3e-09

Identities = 25/37 (68%), Positives = 28/37 (76%), Gaps = 0/37 (0%)

Frame = -2

Query 356 KAGVQWRNLGSLQPPPPEFTPFSCLSLPSS*DYRRPP 246

+AGVQW +L S QPPPP F FSCLS+PSS DYR P

Sbjct 74 QAGVQWHDLSSPQPPPPRFKQFSCLSIPSSWDYRHNP 110

>ref|NP_150646.3| alpha-1A adrenergic receptor isoform 2 [Homo sapiens]

Length=475

Score = 56.6 bits (135), Expect = 5e-09

Identities = 27/34 (80%), Positives = 28/34 (83%), Gaps = 0/34 (0%)

Frame = -3

Query 355 RLECSGVIWAHCNLRLPSSRHSPASASQVARITG 254

RLECSG+I AHCNLRLP SR SPASASQ A TG

Sbjct 431 RLECSGMILAHCNLRLPGSRDSPASASQAAGTTG 464

>ref|NP_060190.2| signal-transducing adaptor protein 2 isoform 1 [Homo sapiens]

Length=449

Score = 56.2 bits (134), Expect = 6e-09

Identities = 27/38 (72%), Positives = 31/38 (82%), Gaps = 0/38 (0%)

Frame = -2

Query 215 VEIGFRHVAQAGLGFLNSSDPPASASQSAGITGIRNNS 102

VE GF HVAQAGL L SSDPP SASQSAGITG+ +++

Sbjct 358 VEKGFHHVAQAGLELLTSSDPPTSASQSAGITGVSHHT 395

>ref|NP_009112.1| mitogen-activated protein kinase kinase kinase kinase 1 isoform

2 [Homo sapiens]

Length=833

Score = 54.3 bits (129), Expect = 2e-08

Identities = 26/35 (75%), Positives = 28/35 (80%), Gaps = 0/35 (0%)

Frame = -3

Query 358 PRLECSGVIWAHCNLRLPSSRHSPASASQVARITG 254

PRLECSG I HCNL LP S +SPASAS+VA ITG

Sbjct 798 PRLECSGTISPHCNLLLPGSSNSPASASRVAGITG 832

>ref|NP_078926.3| putative uncharacterized protein C11orf80 [Homo sapiens]

Length=677

Score = 53.9 bits (128), Expect = 3e-08

Identities = 25/41 (61%), Positives = 27/41 (66%), Gaps = 0/41 (0%)

Frame = -3

Query 358 PRLECSGVIWAHCNLRLPSSRHSPASASQVARITGARQHAW 236

PR ECSG + AHCNL LP S S AS +VA IT A HAW

Sbjct 113 PRPECSGAVSAHCNLHLPGSSDSHASVPRVAGITDAHHHAW 153

>ref|XP_003120008.1| PREDICTED: putative uncharacterized protein NCRNA00269-like [Homo

sapiens]

ref|XP_003118926.1| PREDICTED: putative uncharacterized protein NCRNA00269-like [Homo

sapiens]

ref|XP_003120534.1| PREDICTED: putative uncharacterized protein NCRNA00269-like [Homo

sapiens]

Length=123

Score = 53.5 bits (127), Expect = 4e-08

Identities = 27/44 (62%), Positives = 30/44 (69%), Gaps = 0/44 (0%)

Frame = -2

Query 233 LFFVFFVEIGFRHVAQAGLGFLNSSDPPASASQSAGITGIRNNS 102

L FVF VE GF V QAGL FL SSD PA A SAGITG+ + +

Sbjct 35 LIFVFLVETGFHRVGQAGLDFLTSSDLPALACPSAGITGVSHRA 78

Score = 40.0 bits (92), Expect = 5e-04

Identities = 26/55 (48%), Positives = 33/55 (60%), Gaps = 1/55 (1%)

Frame = -3

Query 337 VIWAHCNLRLPSSRHSPASASQVARITGARQHAWPYFLYFL*R*GFAMLPRLALD 173

+I AH +L LP S +SPASASQ+A IT HA F+ FL GF + + LD

Sbjct 1 MISAHGSLHLPGSSYSPASASQIAGITVMCHHAGLIFV-FLVETGFHRVGQAGLD 54

>ref|XP_003119783.1| PREDICTED: serine/threonine-protein phosphatase 5-like [Homo

sapiens]

ref|XP_003118828.1| PREDICTED: serine/threonine-protein phosphatase 5-like [Homo

sapiens]

ref|XP_003120999.1| PREDICTED: serine/threonine-protein phosphatase 5-like [Homo

sapiens]

Length=171

Score = 53.5 bits (127), Expect = 4e-08

Identities = 25/46 (55%), Positives = 28/46 (61%), Gaps = 0/46 (0%)

Frame = -3

Query 358 PRLECSGVIWAHCNLRLPSSRHSPASASQVARITGARQHAWPYFLY 221

PRL+CSG I AHC+L L R P SASQVA G H W FL+

Sbjct 125 PRLKCSGTITAHCSLNLLGPRDPPTSASQVAVTEGMHHHTWLIFLF 170

>ref|NP_001180462.1| serine/threonine-protein kinase Nek4 isoform 2 [Homo sapiens]

Length=752

Score = 53.1 bits (126), Expect = 5e-08

Identities = 27/40 (68%), Positives = 29/40 (73%), Gaps = 0/40 (0%)

Frame = -3

Query 358 PRLECSGVIWAHCNLRLPSSRHSPASASQVARITGARQHA 239

P+LECSG I AH NLRL S SPASAS+VA ITG HA

Sbjct 373 PKLECSGTILAHSNLRLLGSSDSPASASRVAGITGVCHHA 412

>ref|NP_003148.2| serine/threonine-protein kinase Nek4 isoform 1 [Homo sapiens]

Length=841

Score = 53.1 bits (126), Expect = 5e-08

Identities = 27/40 (68%), Positives = 29/40 (73%), Gaps = 0/40 (0%)

Frame = -3

Query 358 PRLECSGVIWAHCNLRLPSSRHSPASASQVARITGARQHA 239

P+LECSG I AH NLRL S SPASAS+VA ITG HA

Sbjct 462 PKLECSGTILAHSNLRLLGSSDSPASASRVAGITGVCHHA 501

>ref|XP_003119948.1| PREDICTED: hypothetical protein LOC100508257 [Homo sapiens]

ref|XP_003118917.1| PREDICTED: hypothetical protein LOC100507149 [Homo sapiens]

ref|XP_003120447.1| PREDICTED: hypothetical protein LOC100508257 [Homo sapiens]

Length=139

Score = 46.2 bits (108), Expect(2) = 9e-08

Identities = 28/75 (38%), Positives = 38/75 (51%), Gaps = 0/75 (0%)

Frame = -2

Query 233 LFFVFFVEIGFRHVAQAGLGFLNSSDPPASASQSAGITGIRNNS*N*EKKTLWSQXCFVF 54

L + F+E G +V+QAGL L SS PP ASQSAGIT + + + L V

Sbjct 26 LIYFLFLETGSHYVSQAGLQLLGSSSPPTLASQSAGITCTSHRACRLKHLNLKGSFKIVE 85

Query 53 PFLFLFKMYLFIFET 9

F++ Y F F +

Sbjct 86 GFIYFKLTYSFDFHS 100

Score = 25.8 bits (55), Expect(2) = 9e-08

Identities = 12/23 (53%), Positives = 13/23 (57%), Gaps = 0/23 (0%)

Frame = -3

Query 283 SASQVARITGARQHAWPYFLYFL 215

SA QV TGA HAW + FL

Sbjct 10 SAPQVEGTTGACHHAWLIYFLFL 32

>ref|XP_003120664.1| PREDICTED: hypothetical protein LOC100510512 [Homo sapiens]

Length=177

Score = 52.0 bits (123), Expect = 1e-07

Identities = 28/53 (53%), Positives = 34/53 (65%), Gaps = 7/53 (13%)

Frame = -3

Query 355 RLECSGVIWAHCNLRLPSSRHSPASASQVARITGARQHA-------WPYFLYF 218

RLECSG+I +C+L+L SR SP SASQVA TG QHA +FL+F

Sbjct 119 RLECSGMIITYCSLKLLGSRDSPVSASQVAGTTGMHQHARLNFFLSLSFFLFF 171

>ref|XP_003120124.1| PREDICTED: hypothetical protein LOC100507236, partial [Homo sapiens]

ref|XP_003119212.1| PREDICTED: hypothetical protein LOC100507236, partial [Homo sapiens]

Length=174

Score = 52.0 bits (123), Expect = 1e-07

Identities = 28/53 (53%), Positives = 34/53 (65%), Gaps = 7/53 (13%)

Frame = -3

Query 355 RLECSGVIWAHCNLRLPSSRHSPASASQVARITGARQHA-------WPYFLYF 218

RLECSG+I +C+L+L SR SP SASQVA TG QHA +FL+F

Sbjct 119 RLECSGMIITYCSLKLLGSRDSPVSASQVAGTTGMHQHARLNFFLSLSFFLFF 171

>ref|NP_862828.1| zinc finger protein 283 [Homo sapiens]

Length=679

Score = 52.0 bits (123), Expect = 1e-07

Identities = 25/33 (76%), Positives = 27/33 (82%), Gaps = 0/33 (0%)

Frame = -2

Query 356 KAGVQWRNLGSLQPPPPEFTPFSCLSLPSS*DY 258

+AGVQW +LGSLQ PPP FT FSCLSL SS DY

Sbjct 8 QAGVQWCDLGSLQAPPPGFTLFSCLSLLSSWDY 40

>ref|XP_003119509.1| PREDICTED: hypothetical protein LOC100507445 [Homo sapiens]

ref|XP_003118667.1| PREDICTED: hypothetical protein LOC100507445 [Homo sapiens]

ref|XP_003120698.1| PREDICTED: hypothetical protein LOC100507445 [Homo sapiens]

Length=121

Score = 52.0 bits (123), Expect = 1e-07

Identities = 33/65 (51%), Positives = 38/65 (59%), Gaps = 1/65 (1%)

Frame = +3

Query 129 STLGG*GRRIT*VQESKASLGNMAKPYLYKKYKK*GQACWRAPVILATWEAEAGEWRELG 308

STLGG G RIT QE + SL N+ K L K + W+APV+ A WE EAGE E

Sbjct 58 STLGGQGGRITRDQEFETSLDNIVKRRLTKNINI-SRVWWQAPVVPAIWETEAGESLEPR 116

Query 309 RRRLQ 323

R RLQ

Sbjct 117 RWRLQ 121

>ref|NP_001137385.1| hypothetical protein LOC199870 isoform 2 [Homo sapiens]

Length=312

Score = 51.6 bits (122), Expect = 2e-07

Identities = 24/36 (67%), Positives = 28/36 (78%), Gaps = 0/36 (0%)

Frame = -3

Query 355 RLECSGVIWAHCNLRLPSSRHSPASASQVARITGAR 248

RLEC+G I AHCNL LP S SPAS+S+VA ITG +

Sbjct 49 RLECNGTISAHCNLHLPGSSDSPASSSRVAGITGIK 84

>ref|NP_001137384.1| hypothetical protein LOC199870 isoform 1 [Homo sapiens]

Length=341

Score = 51.6 bits (122), Expect = 2e-07

Identities = 24/36 (67%), Positives = 28/36 (78%), Gaps = 0/36 (0%)

Frame = -3

Query 355 RLECSGVIWAHCNLRLPSSRHSPASASQVARITGAR 248

RLEC+G I AHCNL LP S SPAS+S+VA ITG +

Sbjct 49 RLECNGTISAHCNLHLPGSSDSPASSSRVAGITGIK 84

>ref|NP_115861.1| peptidyl-prolyl cis-trans isomerase-like 3 isoform PPIL3a [Homo

sapiens]

Length=165

Score = 51.6 bits (122), Expect = 2e-07

Identities = 22/29 (76%), Positives = 24/29 (83%), Gaps = 0/29 (0%)

Frame = -2

Query 359 PKAGVQWRNLGSLQPPPPEFTPFSCLSLP 273

P+AGVQWR+LGSLQPPPP F CLSLP

Sbjct 33 PQAGVQWRDLGSLQPPPPGFKQVFCLSLP 61

>ref|NP_056087.2| protein fantom isoform a [Homo sapiens]

Length=1315

Score = 51.2 bits (121), Expect = 2e-07

Identities = 26/42 (62%), Positives = 26/42 (62%), Gaps = 0/42 (0%)

Frame = -3

Query 358 PRLECSGVIWAHCNLRLPSSRHSPASASQVARITGARQHAWP 233

P L CS I AHCN RLP S PASASQV ITGA H P

Sbjct 1104 PGLGCSSAISAHCNFRLPGSSDFPASASQVDGITGACHHTQP 1145

>ref|NP_078841.3| cyclin-J-like protein [Homo sapiens]

Length=435

Score = 51.2 bits (121), Expect = 2e-07

Identities = 28/47 (60%), Positives = 31/47 (66%), Gaps = 7/47 (14%)

Frame = -3

Query 373 VSLCHPRLECSGVIWAHCNLRLPSSRHSPASA-------SQVARITG 254

VSL PRL+CSG+I AHCNL LP S +SPASA QVA TG

Sbjct 96 VSLLSPRLKCSGMISAHCNLHLPGSSNSPASAPHPPPTPPQVAETTG 142

>ref|NP_001012680.1| 4F2 cell-surface antigen heavy chain isoform b [Homo sapiens]

Length=631

Score = 50.4 bits (119), Expect = 3e-07

Identities = 37/88 (43%), Positives = 45/88 (52%), Gaps = 3/88 (3%)

Frame = -2

Query 380 RQSLALSPKAGVQWRNLGSLQPPPPEFTPFSCLSLPSS*DYRRPPACLALFFVFFVEIGF 201

RQ +AGVQ + G + + L L +S D PPA + VE GF

Sbjct 17 RQLPGSHSEAGVQGLSAGDDSETGSDCVTQAGLQLLASSD---PPALASKNAEVTVETGF 73

Query 200 RHVAQAGLGFLNSSDPPASASQSAGITG 117

HV+QA + FL S DP ASAS SAGITG

Sbjct 74 HHVSQADIEFLTSIDPTASASGSAGITG 101

>ref|XP_002346169.1| PREDICTED: protein MOST-1-like [Homo sapiens]

ref|XP_003118652.1| PREDICTED: protein MOST-1-like [Homo sapiens]

ref|XP_003120676.1| PREDICTED: protein MOST-1-like [Homo sapiens]

Length=99

Score = 50.4 bits (119), Expect = 3e-07

Identities = 21/30 (70%), Positives = 24/30 (80%), Gaps = 0/30 (0%)

Frame = +1

Query 109 FLMPVIPALWEAEAGGSLEFRNPRPAWATW 198

+L PVIPALW+AEAGG E R+ RPAW TW

Sbjct 70 WLTPVIPALWKAEAGGLPELRSSRPAWTTW 99

>ref|XP_003120114.1| PREDICTED: putative uncharacterized protein LOC65996-like [Homo

sapiens]

ref|XP_003119112.1| PREDICTED: putative uncharacterized protein LOC65996-like [Homo

sapiens]

ref|XP_003118614.1| PREDICTED: putative uncharacterized protein LOC65996-like [Homo

sapiens]

ref|XP_003120654.1| PREDICTED: putative uncharacterized protein LOC65996-like [Homo

sapiens]

Length=120

Score = 49.3 bits (116), Expect(2) = 3e-07

Identities = 29/54 (54%), Positives = 34/54 (63%), Gaps = 4/54 (7%)

Frame = -2

Query 338 RNLGSLQPPPPEFTPFSCLSLPSS*DYRRPPACLALFFVFFVEIGFRHVAQAGL 177

R+L SLQP T SCLSLPS+ +Y R F+F VE+GFRHV QAGL

Sbjct 65 RDLVSLQPA----TSASCLSLPSNWNYYRHAPLQPANFLFLVEMGFRHVGQAGL 114

Score = 20.8 bits (42), Expect(2) = 3e-07

Identities = 8/15 (54%), Positives = 10/15 (67%), Gaps = 0/15 (0%)

Frame = -1

Query 384 FETESRSVTQGWSAV 340

F+TE S GWSA+

Sbjct 50 FKTEFHSYCPGWSAM 64

>ref|NP_001077368.1| platelet glycoprotein VI isoform 1 [Homo sapiens]

Length=620

Score = 41.6 bits (96), Expect(2) = 4e-07

Identities = 23/46 (50%), Positives = 27/46 (59%), Gaps = 0/46 (0%)

Frame = +1

Query 217 KNTKNKARHAGGRL*S*LLGRLRQENGVNSGGGGCSEPKLRHCTPA 354

K T+ AR G L S L LR ENG++ GG GCSE + HCT A

Sbjct 554 KKTQKLARCGGASLYSQQLRGLRWENGLSLGGRGCSELRSHHCTLA 599

Score = 28.1 bits (61), Expect(2) = 4e-07

Identities = 12/20 (60%), Positives = 16/20 (80%), Gaps = 0/20 (0%)

Frame = +3

Query 171 ESKASLGNMAKPYLYKKYKK 230

E+K SL NM KP+LY++ KK

Sbjct 536 ENKISLCNMVKPHLYQQNKK 555

>ref|NP_714912.1| interleukin-12 receptor subunit beta-1 isoform 2 precursor [Homo

sapiens]

Length=381

Score = 50.1 bits (118), Expect = 4e-07

Identities = 27/41 (66%), Positives = 29/41 (71%), Gaps = 1/41 (2%)

Frame = -3

Query 340 GVIWAHCNLRLPSSRHSPASASQVARITGARQHAWPYFLYF 218

G+I AHCNLRLP SR SPASAS+VA ITG H LYF

Sbjct 342 GMISAHCNLRLPDSRDSPASASRVAGITGICHHT-RLILYF 381

>ref|NP_963998.2| thromboxane A2 receptor isoform beta [Homo sapiens]

Length=407

Score = 50.1 bits (118), Expect = 4e-07

Identities = 27/46 (59%), Positives = 29/46 (64%), Gaps = 0/46 (0%)

Frame = -3

Query 358 PRLECSGVIWAHCNLRLPSSRHSPASASQVARITGARQHAWPYFLY 221

P LE SG I AHCNLRLP S S ASAS+ A ITG A P L+

Sbjct 335 PSLEYSGTISAHCNLRLPGSSDSRASASRAAGITGVSHCARPCMLF 380

>ref|NP_001011657.2| zinc finger matrin-type protein 1 isoform 1 [Homo sapiens]

Length=638

Score = 49.7 bits (117), Expect = 6e-07

Identities = 24/35 (69%), Positives = 26/35 (75%), Gaps = 0/35 (0%)

Frame = -3

Query 355 RLECSGVIWAHCNLRLPSSRHSPASASQVARITGA 251

RLECSG I AHC+L LP S SPASASQ+A T A

Sbjct 8 RLECSGAISAHCSLHLPGSSDSPASASQIAGTTDA 42

>ref|NP_001153585.1| hypothetical protein LOC123207 isoform b [Homo sapiens]

Length=167

Score = 45.4 bits (106), Expect(2) = 7e-07

Identities = 20/28 (72%), Positives = 23/28 (83%), Gaps = 0/28 (0%)

Frame = -3

Query 358 PRLECSGVIWAHCNLRLPSSRHSPASAS 275

P+LECSGV+ AHCNL L S +SPASAS

Sbjct 140 PKLECSGVVLAHCNLHLLGSSYSPASAS 167

Score = 23.5 bits (49), Expect(2) = 7e-07

Identities = 10/11 (91%), Positives = 11/11 (100%), Gaps = 0/11 (0%)

Frame = -2

Query 386 FLRQSLALSPK 354

FLRQ+LALSPK

Sbjct 131 FLRQNLALSPK 141

>ref|NP_001155002.1| granulocyte-macrophage colony-stimulating factor receptor subunit

alpha isoform f precursor [Homo sapiens]

Length=434

Score = 49.3 bits (116), Expect = 7e-07

Identities = 22/32 (69%), Positives = 23/32 (72%), Gaps = 0/32 (0%)

Frame = -2

Query 356 KAGVQWRNLGSLQPPPPEFTPFSCLSLPSS*D 261

+AGVQW NLGSLQPP P FSCL LP S D

Sbjct 322 QAGVQWHNLGSLQPPSPRLKRFSCLRLPGSDD 353

>ref|XP_003119710.1| PREDICTED: hypothetical protein LOC100507131 [Homo sapiens]

Length=165

Score = 48.9 bits (115), Expect = 1e-06

Identities = 27/46 (59%), Positives = 29/46 (64%), Gaps = 0/46 (0%)

Frame = -2

Query 356 KAGVQWRNLGSLQPPPPEFTPFSCLSLPSS*DYRRPPACLALFFVF 219

+ GVQWR+LGSLQ P F SCLSL SS YR PP A F VF

Sbjct 25 QVGVQWRDLGSLQLSSPRFKRLSCLSLLSSWYYRCPPPQPANFCVF 70

Score = 42.0 bits (97), Expect = 1e-04

Identities = 33/93 (36%), Positives = 44/93 (48%), Gaps = 7/93 (7%)

Frame = -1

Query 387 FFETESRSVTQ---GWSAVA*FGLTATSASRVHAILLPQPPK*LGLQAPASMPGLIFCIF 217

FFE ESRS Q W + L++ R+ + L + P P FC+F

Sbjct 15 FFEMESRSGAQVGVQWRDLGSLQLSSPRFKRLSCLSLLSS---WYYRCPPPQPAN-FCVF 70

Query 216 CRDRVSPCCPGWPWIPELK*SSCLSLPKCWDYR 118

R+RVS PG +L+ S+ L LPKC DYR

Sbjct 71 TRNRVSLYWPGRSRYSDLRHSARLGLPKCRDYR 103

>ref|XP_003119678.1| PREDICTED: hypothetical protein LOC100506928 [Homo sapiens]

ref|XP_003118761.1| PREDICTED: hypothetical protein LOC100506928 [Homo sapiens]

ref|XP_003120899.1| PREDICTED: hypothetical protein LOC100506928 [Homo sapiens]

Length=159

Score = 48.9 bits (115), Expect = 1e-06

Identities = 23/41 (57%), Positives = 27/41 (66%), Gaps = 0/41 (0%)

Frame = -3

Query 358 PRLECSGVIWAHCNLRLPSSRHSPASASQVARITGARQHAW 236

PRLECSG+I AH +L LP S + P SASQ + TG H W

Sbjct 112 PRLECSGMITAHYSLGLPGSSNPPTSASQGSGTTGMCHHTW 152

>ref|XP_003118780.1| PREDICTED: hypothetical protein LOC100507131 [Homo sapiens]

ref|XP_003120912.1| PREDICTED: hypothetical protein LOC100507131 [Homo sapiens]

Length=165

Score = 48.9 bits (115), Expect = 1e-06

Identities = 27/46 (59%), Positives = 29/46 (64%), Gaps = 0/46 (0%)

Frame = -2

Query 356 KAGVQWRNLGSLQPPPPEFTPFSCLSLPSS*DYRRPPACLALFFVF 219

+ GVQWR+LGSLQ P F SCLSL SS YR PP A F VF

Sbjct 25 QVGVQWRDLGSLQLSSPRFKRLSCLSLLSSWYYRCPPPQPANFCVF 70

Score = 47.0 bits (110), Expect = 4e-06

Identities = 35/93 (38%), Positives = 45/93 (49%), Gaps = 7/93 (7%)

Frame = -1

Query 387 FFETESRSVTQ---GWSAVA*FGLTATSASRVHAILLPQPPK*LGLQAPASMPGLIFCIF 217

FFE ESRS Q W + L++ R+ + L + P P FC+F

Sbjct 15 FFEMESRSGAQVGVQWRDLGSLQLSSPRFKRLSCLSLLSS---WYYRCPPPQPAN-FCVF 70

Query 216 CRDRVSPCCPGWPWIPELK*SSCLSLPKCWDYR 118

RDRVS PG P+L+ S+ L LPKC DYR

Sbjct 71 TRDRVSLYWPGRSRTPDLRHSARLGLPKCRDYR 103

>ref|NP_001158157.1| protein THEMIS isoform 1 [Homo sapiens]

Length=680

Score = 45.4 bits (106), Expect(2) = 1e-06

Identities = 21/28 (75%), Positives = 22/28 (79%), Gaps = 0/28 (0%)

Frame = -2

Query 371 LALSPKAGVQWRNLGSLQPPPPEFTPFS 288

L SPKAGVQWR+LGSLQP PP F FS

Sbjct 581 LPKSPKAGVQWRDLGSLQPLPPGFKQFS 608

Score = 22.7 bits (47), Expect(2) = 1e-06

Identities = 11/22 (50%), Positives = 12/22 (55%), Gaps = 0/22 (0%)

Frame = -3

Query 307 PSSRHSPASASQVARITGARQH 242

P + ASAS VA ITG H

Sbjct 602 PGFKQFSASASHVAGITGTPHH 623

>ref|XP_003119968.1| PREDICTED: hypothetical protein LOC100129516 [Homo sapiens]

ref|XP_003120476.1| PREDICTED: hypothetical protein LOC100129516 [Homo sapiens]

Length=153

Score = 48.5 bits (114), Expect = 1e-06

Identities = 23/32 (72%), Positives = 26/32 (82%), Gaps = 0/32 (0%)

Frame = -2

Query 209 IGFRHVAQAGLGFLNSSDPPASASQSAGITGI 114

+GFR+V QAGL L S DPPAS SQSAGITG+

Sbjct 1 MGFRYVGQAGLEPLTSGDPPASTSQSAGITGV 32

>ref|NP_001158009.1| disrupted in schizophrenia 1 protein isoform a [Homo sapiens]

Length=886

Score = 48.5 bits (114), Expect = 1e-06

Identities = 24/32 (75%), Positives = 26/32 (82%), Gaps = 0/32 (0%)

Frame = -2

Query 215 VEIGFRHVAQAGLGFLNSSDPPASASQSAGIT 120

VE GF +V QAGL L SS+PPASASQSAGIT

Sbjct 373 VETGFHYVGQAGLELLTSSNPPASASQSAGIT 404

>ref|NP_001185728.1| activating signal cointegrator 1 complex subunit 1 isoform a

[Homo sapiens]

Length=400

Score = 48.5 bits (114), Expect = 1e-06

Identities = 25/35 (72%), Positives = 26/35 (75%), Gaps = 0/35 (0%)

Frame = -3

Query 358 PRLECSGVIWAHCNLRLPSSRHSPASASQVARITG 254

PRLE + I AHCNL LP S SPASASQVA ITG

Sbjct 353 PRLEYNDAISAHCNLCLPGSSDSPASASQVAGITG 387

>ref|NP_060313.3| breast carcinoma-amplified sequence 4 isoform a [Homo sapiens]

Length=211

Score = 48.1 bits (113), Expect = 2e-06

Identities = 24/32 (75%), Positives = 25/32 (79%), Gaps = 0/32 (0%)

Frame = -3

Query 352 LECSGVIWAHCNLRLPSSRHSPASASQVARIT 257

+ECSG I A CNLRLP S SPASASQVA IT

Sbjct 164 VECSGTIPARCNLRLPGSSDSPASASQVAGIT 195

>ref|NP_001166173.1| probable sodium-coupled neutral amino acid transporter 6 isoform

1 [Homo sapiens]

Length=521

Score = 47.8 bits (112), Expect = 2e-06

Identities = 23/34 (68%), Positives = 25/34 (74%), Gaps = 0/34 (0%)

Frame = -3

Query 355 RLECSGVIWAHCNLRLPSSRHSPASASQVARITG 254

RL CSGVI AHCNL LP S + P SAS+VA TG

Sbjct 437 RLACSGVISAHCNLCLPDSSNPPTSASRVAETTG 470

>ref|NP_001177143.1| inositol hexakisphosphate and diphosphoinositol-pentakisphosphate

kinase 1 isoform 6 [Homo sapiens]

Length=1406

Score = 47.8 bits (112), Expect = 2e-06

Identities = 24/39 (62%), Positives = 29/39 (75%), Gaps = 0/39 (0%)

Frame = -2

Query 218 FVEIGFRHVAQAGLGFLNSSDPPASASQSAGITGIRNNS 102

++E F HV QAGL L SSD PASASQSAGITG+ + +

Sbjct 1099 WLETRFCHVGQAGLELLTSSDLPASASQSAGITGVSHRT 1137

>ref|NP_001124384.1| suppressor of G2 allele of SKP1 homolog isoform SGT1B [Homo sapiens]

Length=365

Score = 47.8 bits (112), Expect = 2e-06

Identities = 26/51 (51%), Positives = 30/51 (59%), Gaps = 6/51 (11%)

Frame = -2

Query 215 VEIGFRHVAQAGLGFLNSSDPPASASQSAGITGIRNNS*N*EKKTLWSQXC 63

+E GF V QAGL L SSDPPA SQSAGITG N ++W + C

Sbjct 110 IETGFHRVGQAGLQLLTSSDPPALDSQSAGITGADANF------SVWIKRC 154

>ref|NP_777603.1| hypothetical protein LOC283579 isoform 1 [Homo sapiens]

Length=122

Score = 47.8 bits (112), Expect = 2e-06

Identities = 23/33 (70%), Positives = 27/33 (82%), Gaps = 0/33 (0%)

Frame = -2

Query 212 EIGFRHVAQAGLGFLNSSDPPASASQSAGITGI 114

E+G + AQAGL L SS+PPASASQSAGITG+

Sbjct 30 EMGSHYFAQAGLELLGSSNPPASASQSAGITGV 62

>ref|XP_003119709.1| PREDICTED: hypothetical protein LOC100509912 [Homo sapiens]

ref|XP_003118779.1| PREDICTED: hypothetical protein LOC100506809 [Homo sapiens]

ref|XP_003120910.1| PREDICTED: hypothetical protein LOC100509912 [Homo sapiens]

Length=118

Score = 36.2 bits (82), Expect(2) = 3e-06

Identities = 18/34 (53%), Positives = 22/34 (65%), Gaps = 0/34 (0%)

Frame = +2

Query 131 HFGRLRQEDHLSSGIQGQPGQHGETLSLQKIQKI 232

HFGR R+ D+L G+ Q GQ GE+ SL K KI

Sbjct 56 HFGRPRRADYLRIGVPDQRGQRGESPSLLKNTKI 89

Score = 30.8 bits (68), Expect(2) = 3e-06

Identities = 16/26 (62%), Positives = 17/26 (66%), Gaps = 0/26 (0%)

Frame = +3

Query 246 WRAPVILATWEAEAGEWRELGRRRLQ 323

W PVI A E EAGE E GR+RLQ

Sbjct 93 WWVPVIPAIREGEAGESLEPGRQRLQ 118

>ref|NP_001123992.1| zinc finger protein 195 isoform 1 [Homo sapiens]

Length=629

Score = 47.4 bits (111), Expect = 3e-06

Identities = 23/37 (63%), Positives = 27/37 (73%), Gaps = 0/37 (0%)

Frame = -2

Query 212 EIGFRHVAQAGLGFLNSSDPPASASQSAGITGIRNNS 102

E+GF H QA L L SSD PASASQSAGITG+ + +

Sbjct 76 EMGFHHATQACLELLGSSDLPASASQSAGITGVNHRA 112

>ref|NP_001123991.1| zinc finger protein 195 isoform 2 [Homo sapiens]

Length=606

Score = 47.4 bits (111), Expect = 3e-06

Identities = 23/37 (63%), Positives = 27/37 (73%), Gaps = 0/37 (0%)

Frame = -2

Query 212 EIGFRHVAQAGLGFLNSSDPPASASQSAGITGIRNNS 102

E+GF H QA L L SSD PASASQSAGITG+ + +

Sbjct 76 EMGFHHATQACLELLGSSDLPASASQSAGITGVNHRA 112

>ref|NP_789795.1| sulfotransferase 1C2 isoform b [Homo sapiens]

Length=307

Score = 47.0 bits (110), Expect = 4e-06

Identities = 23/33 (70%), Positives = 26/33 (79%), Gaps = 0/33 (0%)

Frame = -2

Query 212 EIGFRHVAQAGLGFLNSSDPPASASQSAGITGI 114

E GF HVAQAGL L+SS+PPAS SQSA IT +

Sbjct 93 ETGFHHVAQAGLKLLSSSNPPASTSQSAKITDL 125

>ref|NP_001166126.1| zinc finger protein 701 isoform 1 [Homo sapiens]

Length=531

Score = 46.6 bits (109), Expect = 5e-06

Identities = 22/36 (62%), Positives = 26/36 (73%), Gaps = 0/36 (0%)

Frame = -2

Query 209 IGFRHVAQAGLGFLNSSDPPASASQSAGITGIRNNS 102

+GF HV Q GL S DPPASASQSAGITG+ + +

Sbjct 1 MGFLHVGQDGLELPTSGDPPASASQSAGITGVSHRT 36

>ref|NP_002899.1| proto-oncogene c-Rel [Homo sapiens]

Length=619

Score = 46.2 bits (108), Expect = 6e-06

Identities = 23/32 (72%), Positives = 23/32 (72%), Gaps = 0/32 (0%)

Frame = -2

Query 215 VEIGFRHVAQAGLGFLNSSDPPASASQSAGIT 120

VE GFRHV Q GL L S DPP ASQSAGIT

Sbjct 308 VETGFRHVDQDGLELLTSGDPPTLASQSAGIT 339

>ref|XP_003119048.1| PREDICTED: hypothetical protein LOC100506073 [Homo sapiens]

ref|XP_003120529.1| PREDICTED: hypothetical protein LOC100506073 [Homo sapiens]

Length=190

Score = 45.8 bits (107), Expect = 8e-06

Identities = 34/83 (41%), Positives = 46/83 (56%), Gaps = 8/83 (9%)

Frame = -2

Query 350 GVQWRNLGSLQPPPPEFTPFSCLSLPSS*DYRRP-PACLALFFVFF---VEIGFRHVAQA 183

G+QW +L SLQP PP F FSCLSL SS D R P + +F +F V G+ ++

Sbjct 35 GMQWLHLCSLQPLPPGFKRFSCLSLLSSWDCRYTLPFPVNVFCIFSRDGVSAGWSDWSRT 94

Query 182 GLGFLNSSDPPASASQSAGITGI 114

+++ PP QS GITG+

Sbjct 95 PDLVIHTPRPP----QSVGITGM 113

>ref|NP_001167449.1| hypothetical protein LOC283579 isoform 2 [Homo sapiens]

Length=92

Score = 45.8 bits (107), Expect = 8e-06

Identities = 22/32 (69%), Positives = 26/32 (82%), Gaps = 0/32 (0%)

Frame = -2

Query 209 IGFRHVAQAGLGFLNSSDPPASASQSAGITGI 114

+G + AQAGL L SS+PPASASQSAGITG+

Sbjct 1 MGSHYFAQAGLELLGSSNPPASASQSAGITGV 32

>ref|NP_660344.2| hypothetical protein LOC201158 isoform 1 [Homo sapiens]

Length=276

Score = 45.8 bits (107), Expect = 8e-06

Identities = 26/54 (49%), Positives = 30/54 (56%), Gaps = 10/54 (18%)

Frame = -2

Query 245 ACLALFFVFFVEIGF----------RHVAQAGLGFLNSSDPPASASQSAGITGI 114

AC L+ +F F RH+AQ GL L S DPPASA QSAGITG+

Sbjct 133 ACSVLWVIFAFSALFSFTVKWLRRSRHIAQTGLKVLGSRDPPASAFQSAGITGV 186

>ref|NP_001030127.1| sorbin and SH3 domain-containing protein 1 isoform 4 [Homo sapiens]

Length=1151

Score = 45.8 bits (107), Expect = 8e-06

Identities = 21/35 (60%), Positives = 26/35 (75%), Gaps = 0/35 (0%)

Frame = -3

Query 370 SLCHPRLECSGVIWAHCNLRLPSSRHSPASASQVA 266

S+ PRLECSG + AHC+L+L S + P SASQVA

Sbjct 438 SVMSPRLECSGTVIAHCSLKLLDSSNPPTSASQVA 472

>ref|XP_003120922.1| PREDICTED: putative uncharacterized protein NCRNA00269-like [Homo

sapiens]

Length=127

Score = 45.4 bits (106), Expect = 1e-05

Identities = 22/34 (65%), Positives = 26/34 (77%), Gaps = 0/34 (0%)

Frame = -3

Query 358 PRLECSGVIWAHCNLRLPSSRHSPASASQVARIT 257

PRLE SG AHC+L LP S ++PASAS+VA IT

Sbjct 81 PRLEYSGTTSAHCSLHLPGSSNAPASASRVAGIT 114

Score = 32.0 bits (71), Expect(2) = 0.003

Identities = 17/34 (50%), Positives = 21/34 (62%), Gaps = 0/34 (0%)

Frame = -3

Query 355 RLECSGVIWAHCNLRLPSSRHSPASASQVARITG 254

R+E SG I AH +L+LP S + SA VA TG

Sbjct 43 RMEFSGPIMAHYSLKLPGSSNPTMSAFPVAGTTG 76

Score = 24.6 bits (52), Expect(2) = 0.003

Identities = 13/24 (55%), Positives = 15/24 (63%), Gaps = 0/24 (0%)

Frame = -2

Query 191 AQAGLGFLNSSDPPASASQSAGIT 120

A L SS+ PASAS+ AGIT

Sbjct 91 AHCSLHLPGSSNAPASASRVAGIT 114

>ref|XP_003119972.1| PREDICTED: hypothetical protein LOC100508228 [Homo sapiens]

ref|XP_003118923.1| PREDICTED: hypothetical protein LOC100506486 [Homo sapiens]

ref|XP_003120486.1| PREDICTED: hypothetical protein LOC100508228 [Homo sapiens]

Length=125

Score = 45.4 bits (106), Expect = 1e-05

Identities = 23/42 (55%), Positives = 27/42 (65%), Gaps = 0/42 (0%)

Frame = -2

Query 233 LFFVFFVEIGFRHVAQAGLGFLNSSDPPASASQSAGITGIRN 108

L FVF V++GF HV Q GL S D PASASQS IT + +

Sbjct 7 LVFVFLVKMGFHHVVQVGLKLPTSGDLPASASQSGEITCVNH 48

>ref|NP_001003690.1| MAD2L1-binding protein isoform 1 [Homo sapiens]

Length=306

Score = 45.1 bits (105), Expect = 1e-05

Identities = 23/33 (70%), Positives = 25/33 (76%), Gaps = 0/33 (0%)

Frame = -3

Query 358 PRLECSGVIWAHCNLRLPSSRHSPASASQVARI 260

PRLE +G+ AH N RLP SR SPASASQVA I

Sbjct 14 PRLEHNGMTSAHHNFRLPGSRDSPASASQVAEI 46

>ref|XP_003119551.1| PREDICTED: hypothetical protein LOC100505593 [Homo sapiens]

ref|XP_003119234.1| PREDICTED: hypothetical protein LOC100505593 [Homo sapiens]

ref|XP_003120754.1| PREDICTED: hypothetical protein LOC100505593 [Homo sapiens]

Length=91

Score = 45.1 bits (105), Expect = 1e-05

Identities = 26/49 (54%), Positives = 31/49 (64%), Gaps = 4/49 (8%)

Frame = +2

Query 116 CL*SQHFGRLRQEDHLSSGIQGQPGQHGETLSLQKIQKIRPGMLAGACN 262

CL SQ RL+QED LS G QGQ GQH ET SL+ + G LA +C+

Sbjct 35 CLQSQLCKRLKQEDRLSPGFQGQLGQHSETSSLKTV----IGPLAPSCH 79

>ref|XP_003119888.1| PREDICTED: hypothetical protein LOC100508579 [Homo sapiens]

ref|XP_003121045.1| PREDICTED: hypothetical protein LOC100508579 [Homo sapiens]

Length=127

Score = 36.2 bits (82), Expect(2) = 2e-05

Identities = 14/18 (78%), Positives = 14/18 (78%), Gaps = 0/18 (0%)

Frame = -1

Query 234 LIFCIFCRDRVSPCCPGW 181

LI CIFCR VSPCC GW

Sbjct 109 LISCIFCRVGVSPCCRGW 126

Score = 28.1 bits (61), Expect(2) = 2e-05

Identities = 15/38 (40%), Positives = 18/38 (48%), Gaps = 0/38 (0%)

Frame = -3

Query 355 RLECSGVIWAHCNLRLPSSRHSPASASQVARITGARQH 242

RLEC G I A + L S + S Q+A IT H

Sbjct 69 RLECCGAITAQGSFDLMGSGNPFTSVDQIAEITNVGNH 106

>ref|NP_001138489.1| proton-coupled amino acid transporter 3 isoform 1 [Homo sapiens]

Length=511

Score = 44.7 bits (104), Expect = 2e-05

Identities = 22/33 (67%), Positives = 24/33 (73%), Gaps = 0/33 (0%)

Frame = -3

Query 358 PRLECSGVIWAHCNLRLPSSRHSPASASQVARI 260

PRLECSG I AHCN L S +SPA AS+VA I

Sbjct 142 PRLECSGKISAHCNPHLQGSSNSPAQASRVAGI 174

>ref|NP_689573.3| zinc finger protein 573 isoform 1 [Homo sapiens]

Length=607

Score = 44.3 bits (103), Expect = 2e-05

Identities = 19/27 (71%), Positives = 21/27 (78%), Gaps = 0/27 (0%)

Frame = -2

Query 356 KAGVQWRNLGSLQPPPPEFTPFSCLSL 276

+AGVQW +L SLQPPPP F FSC SL

Sbjct 8 QAGVQWPDLSSLQPPPPRFKQFSCHSL 34

>ref|NP_955751.1| putative potassium channel regulatory protein isoform 2 [Homo

sapiens]

Length=229

Score = 44.3 bits (103), Expect = 2e-05

Identities = 24/34 (71%), Positives = 26/34 (77%), Gaps = 0/34 (0%)

Frame = -3

Query 355 RLECSGVIWAHCNLRLPSSRHSPASASQVARITG 254

RL C+GVI AH NLRL S SPASAS+VA ITG

Sbjct 193 RLVCNGVISAHHNLRLWGSSDSPASASRVAGITG 226

>ref|NP_001153587.1| hypothetical protein LOC123207 isoform d [Homo sapiens]

Length=167

Score = 43.9 bits (102), Expect = 3e-05

Identities = 22/35 (63%), Positives = 28/35 (80%), Gaps = 0/35 (0%)

Frame = -2

Query 206 GFRHVAQAGLGFLNSSDPPASASQSAGITGIRNNS 102

G ++AQAGL L SSD PASASQSAGITG+ +++

Sbjct 124 GSCYIAQAGLELLASSDLPASASQSAGITGVSHHT 158

>ref|NP_001153583.1| nitric oxide synthase, endothelial isoform 4 [Homo sapiens]

Length=629

Score = 43.9 bits (102), Expect = 3e-05

Identities = 21/36 (59%), Positives = 23/36 (64%), Gaps = 0/36 (0%)

Frame = -3

Query 358 PRLECSGVIWAHCNLRLPSSRHSPASASQVARITGA 251

PRLECS I AHC+L L S + P S SQV TGA

Sbjct 590 PRLECSSTITAHCSLNLLDSSNPPTSTSQVVGTTGA 625

>ref|NP_001005735.1| serine/threonine-protein kinase Chk2 isoform c [Homo sapiens]

Length=586

Score = 43.9 bits (102), Expect = 3e-05

Identities = 21/31 (68%), Positives = 25/31 (81%), Gaps = 0/31 (0%)

Frame = -2

Query 197 HVAQAGLGFLNSSDPPASASQSAGITGIRNN 105

HV Q+ L L SSDPPASASQSAGI G+R++

Sbjct 112 HVTQSDLELLLSSDPPASASQSAGIRGVRHH 142

>ref|NP_001129224.1| protein SGT1 isoform 2 [Homo sapiens]

Length=677

Score = 43.5 bits (101), Expect = 4e-05

Identities = 20/29 (69%), Positives = 22/29 (76%), Gaps = 0/29 (0%)

Frame = -2

Query 347 VQWRNLGSLQPPPPEFTPFSCLSLPSS*D 261

VQWR+ G LQ PPP FTPF CLSL S+ D

Sbjct 379 VQWRDPGLLQAPPPGFTPFICLSLLSTWD 407

>ref|NP_006668.1| ubiquitin carboxyl-terminal hydrolase 19 isoform 4 [Homo sapiens]

Length=1318

Score = 43.5 bits (101), Expect = 4e-05

Identities = 23/37 (63%), Positives = 27/37 (73%), Gaps = 0/37 (0%)

Frame = -2

Query 212 EIGFRHVAQAGLGFLNSSDPPASASQSAGITGIRNNS 102

E G R+VAQAGL L S DP ASAS +AGITG R+ +

Sbjct 42 ETGSRYVAQAGLEPLASGDPSASASHAAGITGSRHRT 78

>ref|NP_114174.1| nuclear prelamin A recognition factor isoform b [Homo sapiens]

Length=502

Score = 43.1 bits (100), Expect = 5e-05

Identities = 25/41 (61%), Positives = 27/41 (66%), Gaps = 0/41 (0%)

Frame = +3

Query 237 QACWRAPVILATWEAEAGEWRELGRRRLQ*AQITPLHSSLG 359

QA W PVI AT EA A E E GR+RLQ +I PL SSLG

Sbjct 260 QAWWCTPVITATREAAARESLEPGRQRLQRDKIAPLDSSLG 300

>ref|NP_001171696.1| BEN domain-containing protein 2 isoform 2 [Homo sapiens]

Length=645

Score = 42.7 bits (99), Expect = 7e-05

Identities = 22/39 (57%), Positives = 24/39 (62%), Gaps = 0/39 (0%)

Frame = -2

Query 356 KAGVQWRNLGSLQPPPPEFTPFSCLSLPSS*DYRRPPAC 240

+AGVQW + SLQP P F LSLPSS D RR P C

Sbjct 86 QAGVQWHDHSSLQPQPLGLKQFFHLSLPSSWDDRRTPPC 124

>ref|NP_001180442.1| zinc transporter 6 isoform 1 [Homo sapiens]

Length=501

Score = 42.7 bits (99), Expect = 7e-05

Identities = 22/40 (55%), Positives = 26/40 (65%), Gaps = 1/40 (2%)

Frame = -1

Query 231 IFCIFCRDRVSPCCPGWPWIPELK*SSCLSLPKCWDYRHK 112

IF +F RD VSP GW P+LK S+ L LPKCWD R +

Sbjct 68 IFDLF-RDGVSPFWLGWSQTPDLKWSTHLGLPKCWDNRRE 106

>ref|NP_116326.2| chromosome 9 open reading frame 37 [Homo sapiens]

Length=176

Score = 42.7 bits (99), Expect = 7e-05

Identities = 21/29 (73%), Positives = 23/29 (80%), Gaps = 0/29 (0%)

Frame = -2

Query 203 FRHVAQAGLGFLNSSDPPASASQSAGITG 117

F +VAQA L L SS+PPASASQS GITG

Sbjct 3 FHYVAQADLELLTSSNPPASASQSTGITG 31

>ref|NP_777547.1| intraflagellar transport protein 20 homolog [Homo sapiens]

Length=148

Score = 42.7 bits (99), Expect = 7e-05

Identities = 21/37 (57%), Positives = 23/37 (63%), Gaps = 0/37 (0%)

Frame = -3

Query 358 PRLECSGVIWAHCNLRLPSSRHSPASASQVARITGAR 248

PRLEC+G I AHC L L S SP S S+V TG R

Sbjct 77 PRLECTGAISAHCKLCLSDSSDSPTSPSRVGGTTGHR 113

>ref|NP_683685.1| 39S ribosomal protein L10, mitochondrial isoform b [Homo sapiens]

Length=271

Score = 42.7 bits (99), Expect = 7e-05

Identities = 21/28 (75%), Positives = 22/28 (79%), Gaps = 0/28 (0%)

Frame = -3

Query 337 VIWAHCNLRLPSSRHSPASASQVARITG 254

+I AHCNL LP S SPASASQVA ITG

Sbjct 1 MISAHCNLHLPGSSDSPASASQVAGITG 28

>ref|NP_699177.2| BEN domain-containing protein 2 isoform 1 [Homo sapiens]

Length=799

Score = 42.7 bits (99), Expect = 7e-05

Identities = 22/39 (57%), Positives = 24/39 (62%), Gaps = 0/39 (0%)

Frame = -2

Query 356 KAGVQWRNLGSLQPPPPEFTPFSCLSLPSS*DYRRPPAC 240

+AGVQW + SLQP P F LSLPSS D RR P C

Sbjct 86 QAGVQWHDHSSLQPQPLGLKQFFHLSLPSSWDDRRTPPC 124

>ref|NP_872321.2| zinc finger protein 714 [Homo sapiens]

Length=555

Score = 36.2 bits (82), Expect(2) = 9e-05

Identities = 16/21 (77%), Positives = 18/21 (86%), Gaps = 0/21 (0%)

Frame = +2

Query 167 SGIQGQPGQHGETLSLQKIQK 229

SG+Q QPGQHG+T SL KIQK

Sbjct 526 SGVQDQPGQHGKTPSLLKIQK 546

Score = 25.4 bits (54), Expect(2) = 9e-05

Identities = 9/18 (50%), Positives = 15/18 (84%), Gaps = 0/18 (0%)

Frame = +3

Query 105 VISYACNPSTLGG*GRRI 158

++++ACNP+TL G G +I

Sbjct 506 MVAHACNPNTLRGLGEQI 523

>ref|NP_001165113.1| myosin-IIIb isoform 3 [Homo sapiens]

Length=1275

Score = 42.4 bits (98), Expect = 9e-05

Identities = 22/34 (65%), Positives = 25/34 (74%), Gaps = 0/34 (0%)

Frame = -3

Query 355 RLECSGVIWAHCNLRLPSSRHSPASASQVARITG 254

RLEC+ +I A CNLR S SPASAS+VA ITG

Sbjct 1200 RLECNSMISADCNLRPLGSSDSPASASRVAGITG 1233

>ref|NP_001012677.1| arginine-fifty homeobox [Homo sapiens]

Length=315

Score = 42.4 bits (98), Expect = 9e-05

Identities = 19/37 (52%), Positives = 25/37 (68%), Gaps = 0/37 (0%)

Frame = -3

Query 355 RLECSGVIWAHCNLRLPSSRHSPASASQVARITGARQ 245

+LECSG + A+C+L LP S P SAS+VA T R+

Sbjct 41 KLECSGTVSAYCSLNLPGSTDPPTSASRVAATTAIRR 77

>ref|XP_002346405.2| PREDICTED: hypothetical protein LOC100294457 [Homo sapiens]

Length=178

Score = 42.0 bits (97), Expect = 1e-04

Identities = 19/24 (80%), Positives = 20/24 (84%), Gaps = 0/24 (0%)

Frame = +1

Query 118 PVIPALWEAEAGGSLEFRNPRPAW 189

PVIPALWEAEAGGS E R+ PAW

Sbjct 153 PVIPALWEAEAGGSPEPRSLTPAW 176

>ref|NP_597994.3| DNA repair protein RAD51 homolog 1 isoform 2 [Homo sapiens]

ref|NP_001157741.1| DNA repair protein RAD51 homolog 1 isoform 2 [Homo sapiens]

Length=340

Score = 41.2 bits (95), Expect = 2e-04

Identities = 20/34 (59%), Positives = 23/34 (68%), Gaps = 0/34 (0%)

Frame = -3

Query 355 RLECSGVIWAHCNLRLPSSRHSPASASQVARITG 254

RLEC+ VI +C LRL S SPASAS+V TG

Sbjct 83 RLECNSVILVYCTLRLSGSSDSPASASRVVGTTG 116

>ref|NP_612412.2| myosin regulatory light chain 10 [Homo sapiens]

Length=226

Score = 41.2 bits (95), Expect = 2e-04

Identities = 21/33 (64%), Positives = 24/33 (73%), Gaps = 0/33 (0%)

Frame = -3

Query 358 PRLECSGVIWAHCNLRLPSSRHSPASASQVARI 260

PRLE +G+I AHCNL L S +SPASASQ I

Sbjct 63 PRLERNGMISAHCNLCLTGSSNSPASASQAFTI 95

Query= Simvastatin Contig 15

Length=369

Score E

Sequences producing significant alignments: (Bits) Value

ref|NP_872601.1| histone demethylase UTY isoform 1 [Homo sapi... 38.9 2e-11

ref|XP_003119960.1| PREDICTED: putative uncharacterized prote... 55.1 1e-08

ref|XP_003121047.1| PREDICTED: KN motif and ankyrin repeat do... 40.4 3e-08

ref|XP_003118557.1| PREDICTED: histone demethylase UTY-like [... 50.4 3e-07

ref|XP_003119968.1| PREDICTED: hypothetical protein LOC100129... 35.0 4e-07

ref|XP_003119248.1| PREDICTED: hypothetical protein LOC100506... 37.0 5e-07

ref|XP_003118843.1| PREDICTED: zinc finger protein ENSP000003... 42.0 1e-06

ref|XP_003119709.1| PREDICTED: hypothetical protein LOC100509... 48.1 2e-06

ref|XP_003119925.1| PREDICTED: histone demethylase UTY-like [... 47.4 3e-06

ref|NP_001124386.1| protein BTG3 isoform a [Homo sapiens] 37.7 4e-06

ref|XP_003119846.1| PREDICTED: hypothetical protein LOC100507... 46.2 6e-06

ref|NP_001166126.1| zinc finger protein 701 isoform 1 [Homo s... 33.5 7e-06

ref|NP_689672.4| hypothetical protein LOC146556 isoform 1 pre... 45.8 8e-06

ref|NP_004563.2| plakophilin-2 isoform 2b [Homo sapiens] 42.0 9e-06

ref|NP_001177143.1| inositol hexakisphosphate and diphosphoin... 33.5 1e-05

ref|XP_003118634.1| PREDICTED: putative uncharacterized prote... 44.7 2e-05

ref|XP_003120094.1| PREDICTED: putative uncharacterized prote... 44.7 2e-05

ref|NP_872321.2| zinc finger protein 714 [Homo sapiens] 34.3 2e-05

ref|XP_003119509.1| PREDICTED: hypothetical protein LOC100507... 33.9 2e-05

ref|XP_003120115.1| PREDICTED: putative uncharacterized prote... 44.3 2e-05

ref|NP_001158009.1| disrupted in schizophrenia 1 protein isof... 43.9 3e-05

ref|XP_003119512.1| PREDICTED: hypothetical protein LOC100507... 43.9 3e-05

ref|NP_777603.1| hypothetical protein LOC283579 isoform 1 [Ho... 35.0 5e-05

ref|NP_001158011.1| disrupted in schizophrenia 1 protein isof... 42.4 9e-05

ref|XP_003119043.1| PREDICTED: hypothetical protein LOC100506... 42.0 1e-04

ref|NP_997719.2| methyltransferase-like protein 10 [Homo sapi... 42.0 1e-04

ref|NP_001167449.1| hypothetical protein LOC283579 isoform 2 ... 35.0 1e-04

ref|XP_003118780.1| PREDICTED: hypothetical protein LOC100507... 41.6 2e-04

ref|NP_001124384.1| suppressor of G2 allele of SKP1 homolog i... 41.6 2e-04

ref|NP_001136036.1| cGMP-gated cation channel alpha-1 isoform... 41.2 2e-04

ref|NP_060190.2| signal-transducing adaptor protein 2 isoform... 41.2 2e-04

ref|XP_003119048.1| PREDICTED: hypothetical protein LOC100506... 40.8 3e-04

ref|XP_003119710.1| PREDICTED: hypothetical protein LOC100507... 40.8 3e-04

ref|XP_003119905.1| PREDICTED: hypothetical protein LOC100509... 40.8 3e-04

ref|NP_001180442.1| zinc transporter 6 isoform 1 [Homo sapiens] 33.5 3e-04

ref|XP_003120111.1| PREDICTED: putative uncharacterized prote... 40.4 3e-04

ref|NP_062553.1| putative uncharacterized protein C8orf44 [Ho... 40.4 3e-04

ref|XP_002346169.1| PREDICTED: protein MOST-1-like [Homo sapi... 30.4 4e-04

ref|NP_001018121.1| podocalyxin isoform 1 precursor [Homo sap... 39.3 8e-04

ref|NP_002899.1| proto-oncogene c-Rel [Homo sapiens] 39.3 8e-04

ref|XP_003120008.1| PREDICTED: putative uncharacterized prote... 38.9 0.001

ref|NP_001138935.1| protein YIF1B isoform 7 [Homo sapiens] 38.9 0.001

ref|NP_116326.2| chromosome 9 open reading frame 37 [Homo sap... 30.8 0.001

ref|NP_789795.1| sulfotransferase 1C2 isoform b [Homo sapiens] 38.5 0.001

ref|NP_001158312.1| LYR motif-containing protein 4 isoform 2 ... 37.7 0.002

ref|XP_003119819.1| PREDICTED: putative uncharacterized prote... 36.6 0.005

ref|XP_003119972.1| PREDICTED: hypothetical protein LOC100508... 36.6 0.005

ref|NP_001164252.1| hypothetical protein LOC159091 isoform 4 ... 36.6 0.005

ref|XP_003119863.1| PREDICTED: hypothetical protein LOC100505... 36.2 0.007

ref|NP_001019386.1| filamin-binding LIM protein 1 isoform b [... 36.2 0.007

ref|NP_001001415.2| zinc finger protein 429 [Homo sapiens] 35.8 0.009

ref|NP_078825.2| tectonic-1 isoform 3 [Homo sapiens] 35.8 0.009

ALIGNMENTS

>ref|NP_872601.1| histone demethylase UTY isoform 1 [Homo sapiens]

Length=1079

Score = 38.9 bits (89), Expect(3) = 2e-11

Identities = 17/19 (90%), Positives = 18/19 (95%), Gaps = 0/19 (0%)

Frame = +1

Query 64 VETGFHHVGQAALKLLTSG 120

VETGFHHVGQA L+LLTSG

Sbjct 1041 VETGFHHVGQACLELLTSG 1059

Score = 37.0 bits (84), Expect(3) = 2e-11

Identities = 14/20 (70%), Positives = 17/20 (85%), Gaps = 0/20 (0%)

Frame = +2

Query 2 LPSSWDYRRPPSRPSNFCIF 61

LP+SW+YR PS P+NFCIF

Sbjct 1021 LPNSWNYRHLPSCPTNFCIF 1040

Score = 27.7 bits (60), Expect(3) = 2e-11

Identities = 13/16 (82%), Positives = 13/16 (82%), Gaps = 0/16 (0%)

Frame = +3

Query 132 SASQSGGITGVSHCTR 179

SASQS GITGVSH R

Sbjct 1064 SASQSAGITGVSHHAR 1079

>ref|XP_003119960.1| PREDICTED: putative uncharacterized protein NCRNA00269-like [Homo

sapiens]

ref|XP_003118569.1| PREDICTED: putative uncharacterized protein NCRNA00269-like [Homo

sapiens]

ref|XP_003120465.1| PREDICTED: putative uncharacterized protein NCRNA00269-like [Homo

sapiens]

Length=128

Score = 55.1 bits (131), Expect = 1e-08

Identities = 24/36 (67%), Positives = 28/36 (78%), Gaps = 0/36 (0%)

Frame = +1

Query 49 FLYFLVETGFHHVGQAALKLLTSGDPPPQPPKVVGL 156

F FLVETGFHH+GQA L+LLTSGDPP + VG+

Sbjct 74 FFVFLVETGFHHIGQAGLELLTSGDPPTSASQSVGI 109

Score = 39.7 bits (91), Expect = 6e-04

Identities = 25/56 (45%), Positives = 30/56 (54%), Gaps = 0/56 (0%)

Frame = +3

Query 3 SQVAGITDARHLVRLIFVFFSRDGVSPRWPGCSQTPDLR*SATSASQSGGITGVSH 170

S+V+GIT +RH +L FVF G + TSASQS GITGVSH

Sbjct 59 SRVSGITGSRHHAQLFFVFLVETGFHHIGQAGLELLTSGDPPTSASQSVGITGVSH 114

>ref|XP_003121047.1| PREDICTED: KN motif and ankyrin repeat domain-containing protein

3-like [Homo sapiens]

Length=143

Score = 40.4 bits (93), Expect(2) = 3e-08

Identities = 17/23 (74%), Positives = 18/23 (79%), Gaps = 0/23 (0%)

Frame = +3

Query 51 FVFFSRDGVSPRWPGCSQTPDLR 119

F FSRDGVSP WPG S TPDL+

Sbjct 121 FCIFSRDGVSPCWPGWSPTPDLK 143

Score = 33.1 bits (74), Expect(2) = 3e-08

Identities = 13/20 (65%), Positives = 15/20 (75%), Gaps = 0/20 (0%)

Frame = +2

Query 2 LPSSWDYRRPPSRPSNFCIF 61

L SSWD+R P P+NFCIF

Sbjct 105 LLSSWDHRHTPPCPANFCIF 124

>ref|XP_003118557.1| PREDICTED: histone demethylase UTY-like [Homo sapiens]

Length=101

Score = 50.4 bits (119), Expect = 3e-07

Identities = 22/34 (65%), Positives = 26/34 (77%), Gaps = 0/34 (0%)

Frame = +1

Query 58 FLVETGFHHVGQAALKLLTSGDPPPQPPKVVGLQ 159

FL+ET FHHVGQA LKLLTSGDPP + G++

Sbjct 56 FLIETRFHHVGQAGLKLLTSGDPPASASQSAGIR 89

>ref|XP_003119968.1| PREDICTED: hypothetical protein LOC100129516 [Homo sapiens]

ref|XP_003120476.1| PREDICTED: hypothetical protein LOC100129516 [Homo sapiens]

Length=153

Score = 35.0 bits (79), Expect(2) = 4e-07

Identities = 14/20 (70%), Positives = 17/20 (85%), Gaps = 0/20 (0%)

Frame = +3

Query 132 SASQSGGITGVSHCTRPRIS 191

S SQS GITGVSHCT+P ++

Sbjct 22 STSQSAGITGVSHCTQPHLT 41

Score = 34.7 bits (78), Expect(2) = 4e-07

Identities = 15/28 (54%), Positives = 19/28 (68%), Gaps = 0/28 (0%)

Frame = +1

Query 73 GFHHVGQAALKLLTSGDPPPQPPKVVGL 156

GF +VGQA L+ LTSGDPP + G+

Sbjct 2 GFRYVGQAGLEPLTSGDPPASTSQSAGI 29

>ref|XP_003119248.1| PREDICTED: hypothetical protein LOC100506511 [Homo sapiens]

Length=402

Score = 37.0 bits (84), Expect(2) = 5e-07

Identities = 18/32 (57%), Positives = 21/32 (66%), Gaps = 0/32 (0%)

Frame = +1

Query 61 LVETGFHHVGQAALKLLTSGDPPPQPPKVVGL 156

LVET F HV QA L+LL SGDPP + G+

Sbjct 353 LVETVFLHVDQAGLELLISGDPPTSASQSAGI 384

Score = 32.3 bits (72), Expect(2) = 5e-07

Identities = 12/19 (64%), Positives = 14/19 (74%), Gaps = 0/19 (0%)

Frame = +2

Query 2 LPSSWDYRRPPSRPSNFCI 58

LPSSW Y PSRP+NF +

Sbjct 334 LPSSWGYSHAPSRPANFVV 352

>ref|XP_003118843.1| PREDICTED: zinc finger protein ENSP00000375192-like [Homo sapiens]

ref|XP_003120983.1| PREDICTED: zinc finger protein ENSP00000375192-like [Homo sapiens]

Length=245

Score = 42.0 bits (97), Expect(2) = 1e-06

Identities = 18/24 (75%), Positives = 19/24 (80%), Gaps = 0/24 (0%)

Frame = +1

Query 58 FLVETGFHHVGQAALKLLTSGDPP 129

FLVETGFHH QA +LLTS DPP

Sbjct 156 FLVETGFHHFDQAGFELLTSSDPP 179

Score = 26.2 bits (56), Expect(2) = 1e-06

Identities = 13/24 (55%), Positives = 14/24 (59%), Gaps = 0/24 (0%)

Frame = +3

Query 3 SQVAGITDARHLVRLIFVFFSRDG 74

SQ AGI A H +LIFVF G

Sbjct 138 SQAAGIAGACHHAQLIFVFLVETG 161

>ref|XP_003119709.1| PREDICTED: hypothetical protein LOC100509912 [Homo sapiens]

ref|XP_003118779.1| PREDICTED: hypothetical protein LOC100506809 [Homo sapiens]

ref|XP_003120910.1| PREDICTED: hypothetical protein LOC100509912 [Homo sapiens]

Length=118

Score = 48.1 bits (113), Expect = 2e-06

Identities = 27/42 (65%), Positives = 30/42 (72%), Gaps = 1/42 (2%)

Frame = -3

Query 127 ADHLRSGV*EQPGQRGETPSLLKNTKIRRTRWRASVIPATWE 2

AD+LR GV +Q GQRGE+PSLLKNTKI W VIPA E

Sbjct 63 ADYLRIGVPDQRGQRGESPSLLKNTKI-SWAWWVPVIPAIRE 103

>ref|XP_003119925.1| PREDICTED: histone demethylase UTY-like [Homo sapiens]

ref|XP_003120437.1| PREDICTED: histone demethylase UTY-like [Homo sapiens]

Length=101

Score = 47.4 bits (111), Expect = 3e-06

Identities = 21/34 (62%), Positives = 25/34 (74%), Gaps = 0/34 (0%)

Frame = +1

Query 58 FLVETGFHHVGQAALKLLTSGDPPPQPPKVVGLQ 159

FL+ET F HVGQA LKLLTSGDPP + G++

Sbjct 56 FLIETRFRHVGQAGLKLLTSGDPPASASQSAGIR 89

>ref|NP_001124386.1| protein BTG3 isoform a [Homo sapiens]

Length=296

Score = 37.7 bits (86), Expect(2) = 4e-06

Identities = 15/17 (89%), Positives = 15/17 (89%), Gaps = 0/17 (0%)

Frame = +3

Query 66 RDGVSPRWPGCSQTPDL 116

RDGVSP WP CSQTPDL

Sbjct 105 RDGVSPCWPDCSQTPDL 121

Score = 28.5 bits (62), Expect(2) = 4e-06

Identities = 15/24 (63%), Positives = 16/24 (67%), Gaps = 0/24 (0%)

Frame = +2

Query 119 VIRHLSLPKWWDYRREPLHPA*NF 190

VIR PK DYRREPL PA +F

Sbjct 122 VIRPPWPPKALDYRREPLRPASSF 145

>ref|XP_003119846.1| PREDICTED: hypothetical protein LOC100507929 [Homo sapiens]

ref|XP_003119023.1| PREDICTED: hypothetical protein LOC100507664 [Homo sapiens]

ref|XP_003120394.1| PREDICTED: hypothetical protein LOC100507929 [Homo sapiens]

Length=122

Score = 46.2 bits (108), Expect = 6e-06

Identities = 26/68 (39%), Positives = 31/68 (46%), Gaps = 0/68 (0%)

Frame = -3

Query 205 VLLFKEILGRVQWLTPVIPPLWEAEVADHLRSGV*EQPGQRGETPSLLKNTKIRRTRWRA 26

VL F + +QWLTPVI WEA L + + KNTKI W+

Sbjct 4 VLYFINLSSCLQWLTPVIQVFWEAGAGVLLEPRSPRSACATRQNSTSTKNTKISWVWWQV 63

Query 25 SVIPATWE 2

VIP TWE

Sbjct 64 PVIPTTWE 71

>ref|NP_001166126.1| zinc finger protein 701 isoform 1 [Homo sapiens]

Length=531

Score = 33.5 bits (75), Expect(2) = 7e-06

Identities = 15/28 (54%), Positives = 18/28 (65%), Gaps = 0/28 (0%)

Frame = +1

Query 73 GFHHVGQAALKLLTSGDPPPQPPKVVGL 156

GF HVGQ L+L TSGDPP + G+

Sbjct 2 GFLHVGQDGLELPTSGDPPASASQSAGI 29

Score = 32.0 bits (71), Expect(2) = 7e-06

Identities = 17/33 (52%), Positives = 21/33 (64%), Gaps = 0/33 (0%)

Frame = +3

Query 132 SASQSGGITGVSHCTRPRISLNNKTSDM*LEER 230

SASQS GITGVSH T+P + D+ EE+

Sbjct 22 SASQSAGITGVSHRTQPPCFEGLTSKDLVREEK 54

>ref|NP_689672.4| hypothetical protein LOC146556 isoform 1 precursor [Homo sapiens]

Length=402

Score = 45.8 bits (107), Expect = 8e-06

Identities = 22/36 (62%), Positives = 24/36 (67%), Gaps = 0/36 (0%)

Frame = +1

Query 49 FLYFLVETGFHHVGQAALKLLTSGDPPPQPPKVVGL 156

F FLVETGFHHV A L+LL S DPP + VGL

Sbjct 367 FYIFLVETGFHHVAHAGLELLISRDPPTSGSQSVGL 402

>ref|NP_004563.2| plakophilin-2 isoform 2b [Homo sapiens]

Length=881

Score = 42.0 bits (97), Expect(2) = 9e-06

Identities = 18/22 (82%), Positives = 19/22 (87%), Gaps = 0/22 (0%)

Frame = -2

Query 191 RNSRPGAVAHACNPTTLGG*GG 126

RN PGAVAHACNP+TLGG GG

Sbjct 468 RNGWPGAVAHACNPSTLGGQGG 489

Score = 23.1 bits (48), Expect(2) = 9e-06

Identities = 11/24 (46%), Positives = 15/24 (63%), Gaps = 0/24 (0%)

Frame = -3

Query 115 RSGV*EQPGQRGETPSLLKNTKIR 44

RSGV +QP Q G +L N K++

Sbjct 493 RSGVRDQPDQHGLLWNLSSNDKLK 516

>ref|NP_001177143.1| inositol hexakisphosphate and diphosphoinositol-pentakisphosphate

kinase 1 isoform 6 [Homo sapiens]

Length=1406

Score = 33.5 bits (75), Expect(2) = 1e-05

Identities = 16/31 (52%), Positives = 20/31 (65%), Gaps = 0/31 (0%)

Frame = +1

Query 64 VETGFHHVGQAALKLLTSGDPPPQPPKVVGL 156

+ET F HVGQA L+LLTS D P + G+

Sbjct 1100 LETRFCHVGQAGLELLTSSDLPASASQSAGI 1130

Score = 30.8 bits (68), Expect(2) = 1e-05

Identities = 14/17 (83%), Positives = 15/17 (89%), Gaps = 0/17 (0%)

Frame = +3

Query 132 SASQSGGITGVSHCTRP 182

SASQS GITGVSH T+P

Sbjct 1123 SASQSAGITGVSHRTQP 1139

>ref|XP_003118634.1| PREDICTED: putative uncharacterized protein NCRNA00269-like [Homo

sapiens]

ref|XP_003120632.1| PREDICTED: putative uncharacterized protein NCRNA00269-like [Homo

sapiens]

Length=140

Score = 44.7 bits (104), Expect = 2e-05

Identities = 20/33 (61%), Positives = 23/33 (70%), Gaps = 0/33 (0%)

Frame = +1

Query 58 FLVETGFHHVGQAALKLLTSGDPPPQPPKVVGL 156

FLVE GFHHVGQ L+LLTS DPP + G+

Sbjct 79 FLVEAGFHHVGQDGLELLTSNDPPTLASQSAGI 111

Score = 39.7 bits (91), Expect = 6e-04

Identities = 28/68 (42%), Positives = 31/68 (46%), Gaps = 0/68 (0%)

Frame = +3

Query 3 SQVAGITDARHLVRLIFVFFSRDGVSPRWPGCSQTPDLR*SATSASQSGGITGVSHCTRP 182

SQVAG T A H LIFVF G + T ASQS GITGVSH P

Sbjct 61 SQVAGTTGACHHAWLIFVFLVEAGFHHVGQDGLELLTSNDPPTLASQSAGITGVSHRAWP 120

Query 183 RISLNNKT 206

+ + T

Sbjct 121 AVVVKKAT 128

>ref|XP_003120094.1| PREDICTED: putative uncharacterized protein NCRNA00269-like [Homo

sapiens]

ref|XP_003119097.1| PREDICTED: putative uncharacterized protein NCRNA00269-like [Homo

sapiens]

Length=140

Score = 44.7 bits (104), Expect = 2e-05

Identities = 20/33 (61%), Positives = 23/33 (70%), Gaps = 0/33 (0%)

Frame = +1

Query 58 FLVETGFHHVGQAALKLLTSGDPPPQPPKVVGL 156

FLVE GFHHVGQ L+LLTS DPP + G+

Sbjct 79 FLVEAGFHHVGQDGLELLTSNDPPTLASQSAGI 111

Score = 39.7 bits (91), Expect = 6e-04

Identities = 28/68 (42%), Positives = 31/68 (46%), Gaps = 0/68 (0%)

Frame = +3

Query 3 SQVAGITDARHLVRLIFVFFSRDGVSPRWPGCSQTPDLR*SATSASQSGGITGVSHCTRP 182

SQVAG T A H LIFVF G + T ASQS GITGVSH P

Sbjct 61 SQVAGTTGACHHAWLIFVFLVEAGFHHVGQDGLELLTSNDPPTLASQSAGITGVSHRAWP 120

Query 183 RISLNNKT 206

+ + T

Sbjct 121 AVVVKKAT 128

>ref|NP_872321.2| zinc finger protein 714 [Homo sapiens]

Length=555

Score = 34.3 bits (77), Expect(2) = 2e-05

Identities = 16/22 (73%), Positives = 18/22 (82%), Gaps = 0/22 (0%)

Frame = -3

Query 115 RSGV*EQPGQRGETPSLLKNTK 50

RSGV +QPGQ G+TPSLLK K

Sbjct 525 RSGVQDQPGQHGKTPSLLKIQK 546

Score = 29.6 bits (65), Expect(2) = 2e-05

Identities = 13/21 (62%), Positives = 14/21 (67%), Gaps = 0/21 (0%)

Frame = -2

Query 191 RNSRPGAVAHACNPTTLGG*G 129

R + G VAHACNP TL G G

Sbjct 500 RKIQQGMVAHACNPNTLRGLG 520

>ref|XP_003119509.1| PREDICTED: hypothetical protein LOC100507445 [Homo sapiens]

ref|XP_003118667.1| PREDICTED: hypothetical protein LOC100507445 [Homo sapiens]

ref|XP_003120698.1| PREDICTED: hypothetical protein LOC100507445 [Homo sapiens]

Length=121

Score = 33.9 bits (76), Expect(2) = 2e-05

Identities = 23/62 (38%), Positives = 32/62 (52%), Gaps = 2/62 (3%)

Frame = -1

Query 252 RSYLNSHFSLQATYQKSYCLKKF*AGCSGSRL*SHHFGRLRWRIT*GQEFESSLANVVKP 73

+ Y H S+ + + +Y K GC G+ + G RIT QEFE+SL N+VK

Sbjct 26 KHYKQCHLSMFSYHLMNYV--KNVKGCPGAVAHTSTLGGQGGRITRDQEFETSLDNIVKR 83

Query 72 RL 67

RL

Sbjct 84 RL 85

Score = 30.0 bits (66), Expect(2) = 2e-05

Identities = 12/22 (55%), Positives = 14/22 (64%), Gaps = 0/22 (0%)

Frame = -3

Query 67 LLKNTKIRRTRWRASVIPATWE 2

L KN I R W+A V+PA WE

Sbjct 85 LTKNINISRVWWQAPVVPAIWE 106

>ref|XP_003120115.1| PREDICTED: putative uncharacterized protein NCRNA00269-like [Homo

sapiens]

ref|XP_003119111.1| PREDICTED: putative uncharacterized protein NCRNA00269-like [Homo

sapiens]

ref|XP_003118613.1| PREDICTED: putative uncharacterized protein NCRNA00269-like [Homo

sapiens]

ref|XP_003120653.1| PREDICTED: putative uncharacterized protein NCRNA00269-like [Homo

sapiens]

Length=137

Score = 44.3 bits (103), Expect = 2e-05

Identities = 21/36 (59%), Positives = 24/36 (67%), Gaps = 0/36 (0%)

Frame = +1

Query 49 FLYFLVETGFHHVGQAALKLLTSGDPPPQPPKVVGL 156

FL FLVE GF H+GQA LKLL S D P + VG+

Sbjct 79 FLVFLVEMGFRHIGQAGLKLLASSDVPISASQSVGI 114

>ref|NP_001158009.1| disrupted in schizophrenia 1 protein isoform a [Homo sapiens]

Length=886

Score = 43.9 bits (102), Expect = 3e-05

Identities = 23/46 (50%), Positives = 28/46 (61%), Gaps = 0/46 (0%)

Frame = +1

Query 55 YFLVETGFHHVGQAALKLLTSGDPPPQPPKVVGLQA*ATAPGLEFL 192

Y VETGFH+VGQA L+LLTS +PP + G+ A LE L

Sbjct 370 YDKVETGFHYVGQAGLELLTSSNPPASASQSAGITAETLQQRLEDL 415

>ref|XP_003119512.1| PREDICTED: hypothetical protein LOC100507663 [Homo sapiens]

ref|XP_003118669.1| PREDICTED: hypothetical protein LOC100507663 [Homo sapiens]

ref|XP_003120701.1| PREDICTED: hypothetical protein LOC100507663 [Homo sapiens]

Length=124

Score = 43.9 bits (102), Expect = 3e-05

Identities = 26/53 (50%), Positives = 32/53 (61%), Gaps = 4/53 (7%)

Frame = -3

Query 193 KEILGRVQWLTPVIPPLWEAEV--ADHLRSGV*EQPGQRGETPSLLKNTKIRR 41

K ++G+V WLTPVIP LWEAEV + +RS P R P KNTK+ R

Sbjct 68 KAVVGQVWWLTPVIPALWEAEVGGSPEVRSSRPAWPTLR--NPVSTKNTKLAR 118

>ref|NP_777603.1| hypothetical protein LOC283579 isoform 1 [Homo sapiens]

Length=122

Score = 35.0 bits (79), Expect(2) = 5e-05

Identities = 15/17 (89%), Positives = 15/17 (89%), Gaps = 0/17 (0%)

Frame = +3

Query 132 SASQSGGITGVSHCTRP 182

SASQS GITGVSHC RP

Sbjct 52 SASQSAGITGVSHCARP 68

Score = 27.7 bits (60), Expect(2) = 5e-05

Identities = 12/30 (40%), Positives = 17/30 (57%), Gaps = 0/30 (0%)

Frame = +1

Query 67 ETGFHHVGQAALKLLTSGDPPPQPPKVVGL 156

E G H+ QA L+LL S +PP + G+

Sbjct 30 EMGSHYFAQAGLELLGSSNPPASASQSAGI 59

>ref|NP_001158011.1| disrupted in schizophrenia 1 protein isoform c [Homo sapiens]

Length=755

Score = 42.4 bits (98), Expect = 9e-05

Identities = 19/33 (58%), Positives = 22/33 (67%), Gaps = 0/33 (0%)

Frame = +1

Query 58 FLVETGFHHVGQAALKLLTSGDPPPQPPKVVGL 156

FLVE GF+HV Q LKLLTS DPP + G+

Sbjct 712 FLVEMGFYHVDQTGLKLLTSSDPPSSASQSAGI 744

Score = 28.5 bits (62), Expect(2) = 0.004

Identities = 12/18 (67%), Positives = 14/18 (78%), Gaps = 0/18 (0%)

Frame = +3

Query 129 TSASQSGGITGVSHCTRP 182

+SASQS GIT +SHC P

Sbjct 736 SSASQSAGITDMSHCAWP 753

Score = 27.3 bits (59), Expect(2) = 0.004

Identities = 19/42 (46%), Positives = 24/42 (58%), Gaps = 5/42 (11%)

Frame = +2

Query 2 LPSSWDYRRPPSRPSNFCIF**RRGF----TTLARLLSNS*P 115

L SSWDYR PP +NF +F GF T +LL++S P

Sbjct 694 LRSSWDYRCPPPCLANF-VFLVEMGFYHVDQTGLKLLTSSDP 734

>ref|XP_003119043.1| PREDICTED: hypothetical protein LOC100506191 [Homo sapiens]

Length=118

Score = 42.0 bits (97), Expect = 1e-04

Identities = 19/26 (74%), Positives = 19/26 (74%), Gaps = 0/26 (0%)

Frame = -2

Query 128 GGSPEVRSLRAAWPTW*NPVSTKKYK 51

GGS E RS R AWPTW NPVSTK K

Sbjct 62 GGSLEARSSRPAWPTWRNPVSTKNTK 87

Score = 38.1 bits (87), Expect = 0.002

Identities = 22/60 (37%), Positives = 26/60 (44%), Gaps = 0/60 (0%)

Frame = -3

Query 181 GRVQWLTPVIPPLWEAEVADHLRSGV*EQPGQRGETPSLLKNTKIRRTRWRASVIPATWE 2

G WL PVI L EA+ L + P KNTKI + W +I ATWE

Sbjct 44 GCAWWLVPVILALGEAKAGGSLEARSSRPAWPTWRNPVSTKNTKISQAWWSMPMISATWE 103

>ref|NP_997719.2| methyltransferase-like protein 10 [Homo sapiens]

Length=291

Score = 42.0 bits (97), Expect = 1e-04

Identities = 21/33 (64%), Positives = 23/33 (70%), Gaps = 0/33 (0%)

Frame = +1

Query 58 FLVETGFHHVGQAALKLLTSGDPPPQPPKVVGL 156

FL ET F HV QA L+LL S D P PPKV+GL

Sbjct 250 FLAETRFCHVVQAGLELLGSSDSPTWPPKVLGL 282

>ref|NP_001167449.1| hypothetical protein LOC283579 isoform 2 [Homo sapiens]

Length=92

Score = 35.0 bits (79), Expect(2) = 1e-04

Identities = 15/17 (89%), Positives = 15/17 (89%), Gaps = 0/17 (0%)

Frame = +3

Query 132 SASQSGGITGVSHCTRP 182

SASQS GITGVSHC RP

Sbjct 22 SASQSAGITGVSHCARP 38

Score = 26.2 bits (56), Expect(2) = 1e-04

Identities = 11/28 (40%), Positives = 16/28 (58%), Gaps = 0/28 (0%)

Frame = +1

Query 73 GFHHVGQAALKLLTSGDPPPQPPKVVGL 156

G H+ QA L+LL S +PP + G+

Sbjct 2 GSHYFAQAGLELLGSSNPPASASQSAGI 29

>ref|XP_003118780.1| PREDICTED: hypothetical protein LOC100507131 [Homo sapiens]

ref|XP_003120912.1| PREDICTED: hypothetical protein LOC100507131 [Homo sapiens]

Length=165

Score = 41.6 bits (96), Expect = 2e-04

Identities = 29/63 (47%), Positives = 35/63 (56%), Gaps = 6/63 (9%)

Frame = +2

Query 2 LPSSWDYRRPPSRPSNFCIF**RRGFTTLARLLSNS*PQVIRH---LSLPKWWDYRREPL 172

L SSW YR PP +P+NFC+F R +L + P +RH L LPK DYR EP

Sbjct 51 LLSSWYYRCPPPQPANFCVF--TRDRVSLYWPGRSRTPD-LRHSARLGLPKCRDYRCEPP 107

Query 173 HPA 181

PA

Sbjct 108 CPA 110

>ref|NP_001124384.1| suppressor of G2 allele of SKP1 homolog isoform SGT1B [Homo sapiens]

Length=365

Score = 41.6 bits (96), Expect = 2e-04

Identities = 18/31 (59%), Positives = 22/31 (71%), Gaps = 0/31 (0%)

Frame = +1

Query 64 VETGFHHVGQAALKLLTSGDPPPQPPKVVGL 156

+ETGFH VGQA L+LLTS DPP + G+

Sbjct 110 IETGFHRVGQAGLQLLTSSDPPALDSQSAGI 140

>ref|NP_001136036.1| cGMP-gated cation channel alpha-1 isoform 1 [Homo sapiens]

Length=759

Score = 41.2 bits (95), Expect = 2e-04

Identities = 19/33 (58%), Positives = 22/33 (67%), Gaps = 0/33 (0%)

Frame = +1

Query 58 FLVETGFHHVGQAALKLLTSGDPPPQPPKVVGL 156

FLVE GFHHVGQA L+LL S D P + G+

Sbjct 35 FLVEMGFHHVGQAGLELLISSDLPTSASQSAGI 67

>ref|NP_060190.2| signal-transducing adaptor protein 2 isoform 1 [Homo sapiens]

Length=449

Score = 41.2 bits (95), Expect = 2e-04

Identities = 18/31 (59%), Positives = 21/31 (68%), Gaps = 0/31 (0%)

Frame = +1

Query 64 VETGFHHVGQAALKLLTSGDPPPQPPKVVGL 156

VE GFHHV QA L+LLTS DPP + G+

Sbjct 358 VEKGFHHVAQAGLELLTSSDPPTSASQSAGI 388

>ref|XP_003119048.1| PREDICTED: hypothetical protein LOC100506073 [Homo sapiens]

ref|XP_003120529.1| PREDICTED: hypothetical protein LOC100506073 [Homo sapiens]

Length=190

Score = 40.8 bits (94), Expect = 3e-04

Identities = 21/40 (53%), Positives = 24/40 (60%), Gaps = 0/40 (0%)

Frame = +3

Query 48 IFVFFSRDGVSPRWPGCSQTPDLR*SATSASQSGGITGVS 167

+F FSRDGVS W S+TPDL QS GITG+S

Sbjct 75 VFCIFSRDGVSAGWSDWSRTPDLVIHTPRPPQSVGITGMS 114

>ref|XP_003119710.1| PREDICTED: hypothetical protein LOC100507131 [Homo sapiens]

Length=165

Score = 40.8 bits (94), Expect = 3e-04

Identities = 26/60 (44%), Positives = 30/60 (50%), Gaps = 0/60 (0%)

Frame = +2

Query 2 LPSSWDYRRPPSRPSNFCIF**RRGFTTLARLLSNS*PQVIRHLSLPKWWDYRREPLHPA 181

L SSW YR PP +P+NFC+F R S + L LPK DYR EP PA

Sbjct 51 LLSSWYYRCPPPQPANFCVFTRNRVSLYWPGRSRYSDLRHSARLGLPKCRDYRCEPPCPA 110

>ref|XP_003119905.1| PREDICTED: hypothetical protein LOC100509763 [Homo sapiens]

ref|XP_003121086.1| PREDICTED: hypothetical protein LOC100510264 [Homo sapiens]

Length=101

Score = 40.8 bits (94), Expect = 3e-04

Identities = 21/37 (57%), Positives = 23/37 (63%), Gaps = 9/37 (24%)

Frame = -2

Query 236 VISLFKLHIRSLIV*RNSRPGAVAHACNPTTLGG*GG 126

V S FK H SRP A+AHACNP+TLGG GG

Sbjct 71 VFSFFKSH---------SRPSAMAHACNPSTLGGRGG 98

>ref|NP_001180442.1| zinc transporter 6 isoform 1 [Homo sapiens]

Length=501

Score = 33.5 bits (75), Expect(2) = 3e-04

Identities = 17/24 (71%), Positives = 18/24 (75%), Gaps = 1/24 (4%)

Frame = +3

Query 48 IFVFFSRDGVSPRWPGCSQTPDLR 119

IF F RDGVSP W G SQTPDL+

Sbjct 68 IFDLF-RDGVSPFWLGWSQTPDLK 90

Score = 26.2 bits (56), Expect(2) = 3e-04

Identities = 10/13 (77%), Positives = 10/13 (77%), Gaps = 0/13 (0%)

Frame = +2

Query 128 HLSLPKWWDYRRE 166

HL LPK WD RRE

Sbjct 94 HLGLPKCWDNRRE 106

>ref|XP_003120111.1| PREDICTED: putative uncharacterized protein NCRNA00269-like [Homo

sapiens]

ref|XP_003119110.1| PREDICTED: putative uncharacterized protein NCRNA00269-like [Homo

sapiens]

ref|XP_003118626.1| PREDICTED: putative uncharacterized protein NCRNA00269-like [Homo

sapiens]

ref|XP_003120652.1| PREDICTED: putative uncharacterized protein NCRNA00269-like [Homo

sapiens]

Length=130

Score = 40.4 bits (93), Expect = 3e-04

Identities = 17/33 (52%), Positives = 23/33 (70%), Gaps = 0/33 (0%)

Frame = +1

Query 58 FLVETGFHHVGQAALKLLTSGDPPPQPPKVVGL 156

F+++ GFHHVGQA L+ LTSGD P + G+

Sbjct 83 FVIKMGFHHVGQAGLEFLTSGDLPALASQSAGI 115

Score = 39.3 bits (90), Expect = 8e-04

Identities = 25/60 (42%), Positives = 30/60 (50%), Gaps = 0/60 (0%)

Frame = +3

Query 3 SQVAGITDARHLVRLIFVFFSRDGVSPRWPGCSQTPDLR*SATSASQSGGITGVSHCTRP 182

SQ++GI RH RLIFVF + G + ASQS GIT VSHC +P

Sbjct 65 SQLSGIMCVRHYSRLIFVFVIKMGFHHVGQAGLEFLTSGDLPALASQSAGITDVSHCVQP 124

>ref|NP_062553.1| putative uncharacterized protein C8orf44 [Homo sapiens]

Length=159

Score = 40.4 bits (93), Expect = 3e-04

Identities = 17/26 (66%), Positives = 19/26 (74%), Gaps = 0/26 (0%)

Frame = -2

Query 128 GGSPEVRSLRAAWPTW*NPVSTKKYK 51

G SPEVRS + AWPTW NP+ TK K

Sbjct 54 GRSPEVRSSKPAWPTWRNPIFTKNTK 79

Score = 37.0 bits (84), Expect = 0.004

Identities = 22/47 (47%), Positives = 26/47 (56%), Gaps = 4/47 (8%)

Frame = -3

Query 181 GRVQWLTPVIPPLWEAEV--ADHLRSGV*EQPGQRGETPSLLKNTKI 47

GR +WL PVIP LWEA+ + +RS P R P KNTKI

Sbjct 36 GRARWLMPVIPALWEAKAGRSPEVRSSKPAWPTWR--NPIFTKNTKI 80

>ref|XP_002346169.1| PREDICTED: protein MOST-1-like [Homo sapiens]

ref|XP_003118652.1| PREDICTED: protein MOST-1-like [Homo sapiens]

ref|XP_003120676.1| PREDICTED: protein MOST-1-like [Homo sapiens]

Length=99

Score = 30.4 bits (67), Expect(2) = 4e-04

Identities = 11/14 (79%), Positives = 13/14 (93%), Gaps = 0/14 (0%)

Frame = -3

Query 172 QWLTPVIPPLWEAE 131

+WLTPVIP LW+AE

Sbjct 69 RWLTPVIPALWKAE 82

Score = 29.3 bits (64), Expect(2) = 4e-04

Identities = 11/16 (69%), Positives = 12/16 (75%), Gaps = 0/16 (0%)

Frame = -2

Query 128 GGSPEVRSLRAAWPTW 81

GG PE+RS R AW TW

Sbjct 84 GGLPELRSSRPAWTTW 99

>ref|NP_001018121.1| podocalyxin isoform 1 precursor [Homo sapiens]

Length=558

Score = 39.3 bits (90), Expect = 8e-04

Identities = 19/34 (56%), Positives = 23/34 (68%), Gaps = 0/34 (0%)

Frame = +1

Query 61 LVETGFHHVGQAALKLLTSGDPPPQPPKVVGLQA 162

L+ET FHHV QA L+LLTSGD P + G+ A

Sbjct 235 LLETVFHHVSQAGLELLTSGDLPTLASQSAGITA 268

>ref|NP_002899.1| proto-oncogene c-Rel [Homo sapiens]

Length=619

Score = 39.3 bits (90), Expect = 8e-04

Identities = 18/31 (59%), Positives = 21/31 (68%), Gaps = 0/31 (0%)

Frame = +1

Query 64 VETGFHHVGQAALKLLTSGDPPPQPPKVVGL 156

VETGF HV Q L+LLTSGDPP + G+

Sbjct 308 VETGFRHVDQDGLELLTSGDPPTLASQSAGI 338

>ref|XP_003120008.1| PREDICTED: putative uncharacterized protein NCRNA00269-like [Homo

sapiens]

ref|XP_003118926.1| PREDICTED: putative uncharacterized protein NCRNA00269-like [Homo

sapiens]

ref|XP_003120534.1| PREDICTED: putative uncharacterized protein NCRNA00269-like [Homo

sapiens]

Length=123

Score = 38.9 bits (89), Expect = 0.001

Identities = 18/24 (75%), Positives = 18/24 (75%), Gaps = 0/24 (0%)

Frame = +1

Query 58 FLVETGFHHVGQAALKLLTSGDPP 129

FLVETGFH VGQA L LTS D P

Sbjct 39 FLVETGFHRVGQAGLDFLTSSDLP 62

>ref|NP_001138935.1| protein YIF1B isoform 7 [Homo sapiens]

Length=291

Score = 38.9 bits (89), Expect = 0.001

Identities = 16/18 (89%), Positives = 17/18 (95%), Gaps = 0/18 (0%)

Frame = -2

Query 179 PGAVAHACNPTTLGG*GG 126

PGAVAHACNP+TLGG GG

Sbjct 265 PGAVAHACNPSTLGGRGG 282

>ref|NP_116326.2| chromosome 9 open reading frame 37 [Homo sapiens]

Length=176

Score = 30.8 bits (68), Expect(2) = 0.001

Identities = 13/27 (49%), Positives = 18/27 (67%), Gaps = 0/27 (0%)

Frame = +1

Query 76 FHHVGQAALKLLTSGDPPPQPPKVVGL 156

FH+V QA L+LLTS +PP + G+

Sbjct 3 FHYVAQADLELLTSSNPPASASQSTGI 29

Score = 26.9 bits (58), Expect(2) = 0.001

Identities = 13/17 (77%), Positives = 13/17 (77%), Gaps = 0/17 (0%)

Frame = +3

Query 132 SASQSGGITGVSHCTRP 182

SASQS GITG SH RP

Sbjct 22 SASQSTGITGGSHRARP 38

>ref|NP_789795.1| sulfotransferase 1C2 isoform b [Homo sapiens]

Length=307

Score = 38.5 bits (88), Expect = 0.001

Identities = 16/21 (77%), Positives = 18/21 (86%), Gaps = 0/21 (0%)

Frame = +1

Query 67 ETGFHHVGQAALKLLTSGDPP 129

ETGFHHV QA LKLL+S +PP

Sbjct 93 ETGFHHVAQAGLKLLSSSNPP 113

>ref|NP_001158312.1| LYR motif-containing protein 4 isoform 2 [Homo sapiens]

Length=130

Score = 37.7 bits (86), Expect = 0.002

Identities = 15/19 (79%), Positives = 16/19 (85%), Gaps = 0/19 (0%)

Frame = +2

Query 2 LPSSWDYRRPPSRPSNFCI 58

LPSSWDYRR P R +NFCI

Sbjct 103 LPSSWDYRRTPPRLANFCI 121

>ref|XP_003119819.1| PREDICTED: putative uncharacterized protein C14orf165-like [Homo

sapiens]

ref|XP_003118891.1| PREDICTED: putative uncharacterized protein C14orf165-like [Homo

sapiens]

ref|XP_003120627.1| PREDICTED: putative uncharacterized protein C14orf165-like [Homo

sapiens]

Length=110

Score = 36.6 bits (83), Expect = 0.005

Identities = 19/29 (66%), Positives = 19/29 (66%), Gaps = 2/29 (6%)

Frame = +3

Query 33 HLVRLIFVFFSRDGVSPRWPGCSQTPDLR 119

HLV F FSRD V P WPG SQT DLR

Sbjct 84 HLVN--FCIFSRDKVLPCWPGWSQTSDLR 110

>ref|XP_003119972.1| PREDICTED: hypothetical protein LOC100508228 [Homo sapiens]

ref|XP_003118923.1| PREDICTED: hypothetical protein LOC100506486 [Homo sapiens]

ref|XP_003120486.1| PREDICTED: hypothetical protein LOC100508228 [Homo sapiens]

Length=125

Score = 36.6 bits (83), Expect = 0.005

Identities = 17/24 (71%), Positives = 18/24 (75%), Gaps = 0/24 (0%)

Frame = +1

Query 58 FLVETGFHHVGQAALKLLTSGDPP 129

FLV+ GFHHV Q LKL TSGD P

Sbjct 11 FLVKMGFHHVVQVGLKLPTSGDLP 34

>ref|NP_001164252.1| hypothetical protein LOC159091 isoform 4 [Homo sapiens]

Length=98

Score = 36.6 bits (83), Expect = 0.005

Identities = 17/27 (63%), Positives = 19/27 (71%), Gaps = 0/27 (0%)

Frame = +3

Query 3 SQVAGITDARHLVRLIFVFFSRDGVSP 83

S+VAG + RH LIF FSRDGVSP

Sbjct 71 SRVAGTSGTRHHAHLIFCIFSRDGVSP 97

>ref|XP_003119863.1| PREDICTED: hypothetical protein LOC100505579 [Homo sapiens]

ref|XP_003118854.1| PREDICTED: hypothetical protein LOC100505579 [Homo sapiens]

ref|XP_003121024.1| PREDICTED: hypothetical protein LOC100505579 [Homo sapiens]

Length=119

Score = 36.2 bits (82), Expect = 0.007

Identities = 14/16 (88%), Positives = 15/16 (94%), Gaps = 0/16 (0%)

Frame = -2

Query 182 RPGAVAHACNPTTLGG 135

RPG VAHACNP+TLGG

Sbjct 104 RPGTVAHACNPSTLGG 119

>ref|NP_001019386.1| filamin-binding LIM protein 1 isoform b [Homo sapiens]

Length=374

Score = 36.2 bits (82), Expect = 0.007

Identities = 15/17 (89%), Positives = 16/17 (95%), Gaps = 0/17 (0%)

Frame = -2

Query 176 GAVAHACNPTTLGG*GG 126

GAVAHACNP+TLGG GG

Sbjct 346 GAVAHACNPSTLGGRGG 362

>ref|NP_001001415.2| zinc finger protein 429 [Homo sapiens]

Length=674

Score = 35.8 bits (81), Expect = 0.009

Identities = 15/19 (79%), Positives = 16/19 (85%), Gaps = 0/19 (0%)

Frame = -2

Query 182 RPGAVAHACNPTTLGG*GG 126

R G VAHACNP+TLGG GG

Sbjct 644 RMGVVAHACNPSTLGGRGG 662

>ref|NP_078825.2| tectonic-1 isoform 3 [Homo sapiens]

Length=573

Score = 35.8 bits (81), Expect = 0.009

Identities = 21/60 (35%), Positives = 28/60 (47%), Gaps = 5/60 (8%)

Frame = -3

Query 223 SSYISEVLLFKEILGRVQWLTPVIPPLWEAEVADHLRSGV*EQPGQRGETPSLLKNTKIR 44

SS S + G+ W TPVIP LWEAE L E P R + P +++ I+

Sbjct 224 SSLTSSLCTDNNPAGQAYWFTPVIPALWEAEARGSL-----EVPDSRKKVPITVQSIVIQ 278

Query= Simvastatin Contig 21

Length=253

Score E

Sequences producing significant alignments: (Bits) Value

ref|XP_002342694.1| PREDICTED: hypothetical protein LOC100289... 93.2 5e-20

ALIGNMENTS

>ref|XP_002342694.1| PREDICTED: hypothetical protein LOC100289222 [Homo sapiens]

ref|XP_003120065.1| PREDICTED: hypothetical protein LOC100509631 [Homo sapiens]

ref|XP_003120584.1| PREDICTED: hypothetical protein LOC100509631 [Homo sapiens]

Length=201

Score = 93.2 bits (230), Expect = 5e-20

Identities = 55/98 (57%), Positives = 64/98 (66%), Gaps = 14/98 (14%)

Frame = -1

Query 253 QQSVQE-------------EAEHKSLENLQSDDAIEKK*RFSWEKFKPAAEISICNKEPK 113

QQS+QE EAEHK LENLQ D+ IEKK FS EKFK AA+I I N+E

Sbjct 73 QQSIQEVTWVLLKAFSFIREAEHKRLENLQPDNVIEKKILFSEEKFKLAAKIWISNEELN 132

Query 112 VNYQDNGENVSGACQK-PQQHFPSQAWRPKREK*FYGL 2

+N QDNGENV ACQ+ QQ SQ WRP+R+K F+GL

Sbjct 133 INPQDNGENVCRACQRSSQQLLLSQTWRPRRKKWFHGL 170

Query= Simvastatin Contig 23

Length=164

Score E

Sequences producing significant alignments: (Bits) Value

ref|XP_003119989.1| PREDICTED: hypothetical protein LOC100508... 92.8 6e-20

ref|NP_001158312.1| LYR motif-containing protein 4 isoform 2 ... 80.1 4e-16

ref|XP_003119895.1| PREDICTED: uncharacterized protein FLJ395... 79.7 5e-16

ref|XP_003121047.1| PREDICTED: KN motif and ankyrin repeat do... 79.3 7e-16

ref|NP_872601.1| histone demethylase UTY isoform 1 [Homo sapi... 77.4 3e-15

ref|XP_003118634.1| PREDICTED: putative uncharacterized prote... 75.5 1e-14

ref|XP_003120094.1| PREDICTED: putative uncharacterized prote... 75.5 1e-14

ref|XP_003119819.1| PREDICTED: putative uncharacterized prote... 73.9 3e-14

ref|NP_689672.4| hypothetical protein LOC146556 isoform 1 pre... 73.2 5e-14

ref|NP_078926.3| putative uncharacterized protein C11orf80 [H... 69.3 7e-13

ref|XP_003119960.1| PREDICTED: putative uncharacterized prote... 65.9 8e-12

ref|NP_001164252.1| hypothetical protein LOC159091 isoform 4 ... 65.5 1e-11

ref|XP_003118843.1| PREDICTED: zinc finger protein ENSP000003... 64.7 2e-11

ref|NP_001123498.2| hypothetical protein LOC285966 isoform B ... 59.7 2e-11

ref|NP_001158011.1| disrupted in schizophrenia 1 protein isof... 55.5 5e-11

ref|XP_003120115.1| PREDICTED: putative uncharacterized prote... 62.4 9e-11

ref|NP_963998.2| thromboxane A2 receptor isoform beta [Homo s... 61.6 1e-10

ref|XP_003119710.1| PREDICTED: hypothetical protein LOC100507... 60.8 2e-10

ref|XP_003119783.1| PREDICTED: serine/threonine-protein phosp... 60.8 2e-10

ref|XP_003118780.1| PREDICTED: hypothetical protein LOC100507... 60.8 2e-10

ref|NP_862828.1| zinc finger protein 283 [Homo sapiens] 60.1 4e-10

ref|XP_003119248.1| PREDICTED: hypothetical protein LOC100506... 58.5 1e-09

ref|XP_003119846.1| PREDICTED: hypothetical protein LOC100507... 58.5 1e-09

ref|NP_001171696.1| BEN domain-containing protein 2 isoform 2... 57.4 3e-09

ref|NP_699177.2| BEN domain-containing protein 2 isoform 1 [H... 57.4 3e-09

ref|XP_003120922.1| PREDICTED: putative uncharacterized prote... 57.0 4e-09

ref|NP_114174.1| nuclear prelamin A recognition factor isofor... 57.0 4e-09

ref|XP_003119959.1| PREDICTED: hypothetical protein LOC100287... 56.6 5e-09

ref|NP_001155002.1| granulocyte-macrophage colony-stimulating... 56.2 6e-09

ref|NP_001077368.1| platelet glycoprotein VI isoform 1 [Homo ... 54.7 2e-08

ref|XP_003119678.1| PREDICTED: hypothetical protein LOC100506... 54.3 2e-08

ref|NP_115861.1| peptidyl-prolyl cis-trans isomerase-like 3 i... 54.3 2e-08

ref|NP_689573.3| zinc finger protein 573 isoform 1 [Homo sapi... 52.0 1e-07

ref|NP_001180462.1| serine/threonine-protein kinase Nek4 isof... 52.0 1e-07

ref|NP_003148.2| serine/threonine-protein kinase Nek4 isoform... 52.0 1e-07

ref|XP_003119048.1| PREDICTED: hypothetical protein LOC100506... 51.6 2e-07

ref|NP_001120653.1| centromere protein L isoform 1 [Homo sapi... 51.6 2e-07

ref|XP_003118557.1| PREDICTED: histone demethylase UTY-like [... 51.2 2e-07

ref|NP_001185728.1| activating signal cointegrator 1 complex ... 51.2 2e-07

ref|NP_056087.2| protein fantom isoform a [Homo sapiens] 50.1 4e-07

ref|XP_003120008.1| PREDICTED: putative uncharacterized prote... 49.7 6e-07

ref|NP_001138489.1| proton-coupled amino acid transporter 3 i... 49.7 6e-07

ref|NP_009112.1| mitogen-activated protein kinase kinase kina... 49.7 6e-07

ref|XP_003119925.1| PREDICTED: histone demethylase UTY-like [... 49.3 7e-07

ref|NP_001011657.2| zinc finger matrin-type protein 1 isoform... 48.9 1e-06

ref|NP_150646.3| alpha-1A adrenergic receptor isoform 2 [Homo... 47.8 2e-06

ref|NP_001009923.1| hypothetical protein LOC29058 isoform 1 [... 47.8 2e-06

ref|NP_001182556.1| protein AF-10 isoform d [Homo sapiens] 47.4 3e-06

ref|XP_003119709.1| PREDICTED: hypothetical protein LOC100509... 47.4 3e-06

ref|NP_001137385.1| hypothetical protein LOC199870 isoform 2 ... 47.4 3e-06

ref|NP_001137384.1| hypothetical protein LOC199870 isoform 1 ... 47.4 3e-06

ref|XP_003120664.1| PREDICTED: hypothetical protein LOC100510... 47.0 4e-06

ref|XP_003120124.1| PREDICTED: hypothetical protein LOC100507... 47.0 4e-06

ref|XP_003120111.1| PREDICTED: putative uncharacterized prote... 47.0 4e-06

ref|NP_078841.3| cyclin-J-like protein [Homo sapiens] 47.0 4e-06

ref|NP_001108224.1| complement decay-accelerating factor isof... 46.6 5e-06

ref|NP_997719.2| methyltransferase-like protein 10 [Homo sapi... 42.4 6e-06

ref|NP_714912.1| interleukin-12 receptor subunit beta-1 isofo... 46.2 6e-06

ref|XP_003118848.1| PREDICTED: hypothetical protein LOC100130... 45.4 1e-05

ref|XP_003119870.1| PREDICTED: hypothetical protein LOC100130... 45.4 1e-05

ref|XP_003119972.1| PREDICTED: hypothetical protein LOC100508... 45.1 1e-05

ref|NP_001128626.1| zinc transporter ZIP14 isoform c [Homo sa... 45.1 1e-05

ref|NP_001165113.1| myosin-IIIb isoform 3 [Homo sapiens] 44.7 2e-05

ref|NP_149084.2| tripartite motif-containing protein 5 isofor... 44.7 2e-05

ref|NP_612412.2| myosin regulatory light chain 10 [Homo sapiens] 44.7 2e-05

ref|XP_003119660.1| PREDICTED: hypothetical protein LOC100506... 43.9 3e-05

ref|NP_001153585.1| hypothetical protein LOC123207 isoform b ... 43.5 4e-05

ref|NP_874362.3| ankyrin repeat and death domain-containing p... 43.5 4e-05

ref|NP_001003690.1| MAD2L1-binding protein isoform 1 [Homo sa... 43.5 4e-05

ref|XP_003119509.1| PREDICTED: hypothetical protein LOC100507... 43.5 4e-05

ref|NP_683685.1| 39S ribosomal protein L10, mitochondrial iso... 42.7 7e-05

ref|NP_777547.1| intraflagellar transport protein 20 homolog ... 42.0 1e-04

ref|NP_001139736.1| synaptotagmin-14 isoform 2 [Homo sapiens] 41.6 2e-04

ref|NP_001139733.1| synaptotagmin-14 isoform 1 [Homo sapiens] 41.6 2e-04

ref|NP_060313.3| breast carcinoma-amplified sequence 4 isofor... 41.6 2e-04

ref|NP_001153583.1| nitric oxide synthase, endothelial isofor... 41.2 2e-04

ref|XP_938432.4| PREDICTED: hypothetical protein LOC646021 [H... 40.8 3e-04

ref|NP_001166173.1| probable sodium-coupled neutral amino aci... 40.8 3e-04

ref|NP_001018114.1| fumarylacetoacetate hydrolase domain-cont... 40.8 3e-04

ref|NP_001012677.1| arginine-fifty homeobox [Homo sapiens] 40.8 3e-04

ref|NP_000865.2| interferon alpha/beta receptor 2 isoform b p... 39.7 6e-04

ref|NP_001158157.1| protein THEMIS isoform 1 [Homo sapiens] 32.0 7e-04

ref|NP_001138525.1| EF-hand calcium-binding domain-containing... 38.9 0.001

ref|NP_001098016.1| ribonuclease P protein subunit p30 isofor... 38.9 0.001

ref|XP_003119834.1| PREDICTED: zinc finger protein ENSP000003... 38.9 0.001

ref|NP_001129224.1| protein SGT1 isoform 2 [Homo sapiens] 38.5 0.001

ref|NP_001030127.1| sorbin and SH3 domain-containing protein ... 38.1 0.002

ref|XP_003119043.1| PREDICTED: hypothetical protein LOC100506... 37.4 0.003

ref|NP_001136036.1| cGMP-gated cation channel alpha-1 isoform... 37.4 0.003

ref|XP_002343910.2| PREDICTED: testis-specific Y-encoded prot... 36.6 0.005

ref|NP_065184.2| selenoprotein N isoform 1 precursor [Homo sa... 36.6 0.005

ref|NP_057728.1| proline-rich protein 16 [Homo sapiens] 36.6 0.005

ref|NP_660326.2| nucleoredoxin-like protein 2 isoform 2 [Homo... 36.2 0.007

ALIGNMENTS

>ref|XP_003119989.1| PREDICTED: hypothetical protein LOC100508022 [Homo sapiens]

ref|XP_003118579.1| PREDICTED: hypothetical protein LOC100506688 [Homo sapiens]

ref|XP_003120502.1| PREDICTED: hypothetical protein LOC100508022 [Homo sapiens]

Length=176

Score = 92.8 bits (229), Expect = 6e-20

Identities = 41/54 (76%), Positives = 43/54 (80%), Gaps = 0/54 (0%)

Frame = -1

Query 164 EMKSHSVTQPGVQWCNLS*LQPPPPRFKQFFCLSLPSSWDYRHAPSCMANFCIF 3

E +SHSVTQ GVQWCNLS LQPPPP FKQF CLS PSSW+YRH P C ANF F

Sbjct 62 ETESHSVTQAGVQWCNLSSLQPPPPWFKQFSCLSFPSSWNYRHLPPCPANFLYF 115

Score = 44.7 bits (104), Expect = 2e-05

Identities = 22/31 (71%), Positives = 24/31 (78%), Gaps = 0/31 (0%)

Frame = -2

Query 163 R*SLTLSPSLEYSGAISADCNLHLPGSSNSS 71

R SL++ P LEYSG ISA CN LPGSSNSS

Sbjct 123 RWSLSVLPKLEYSGVISAHCNFCLPGSSNSS 153

>ref|NP_001158312.1| LYR motif-containing protein 4 isoform 2 [Homo sapiens]

Length=130

Score = 80.1 bits (196), Expect = 4e-16

Identities = 37/53 (70%), Positives = 40/53 (76%), Gaps = 0/53 (0%)

Frame = -1

Query 164 EMKSHSVTQPGVQWCNLS*LQPPPPRFKQFFCLSLPSSWDYRHAPSCMANFCI 6

+M SHSV Q GV W +LS LQP PP FKQF CLSLPSSWDYR P +ANFCI

Sbjct 69 QMDSHSVAQAGVHWNDLSSLQPLPPWFKQFSCLSLPSSWDYRRTPPRLANFCI 121

>ref|XP_003119895.1| PREDICTED: uncharacterized protein FLJ39582-like [Homo sapiens]

ref|XP_003118861.1| PREDICTED: uncharacterized protein FLJ39582-like [Homo sapiens]

ref|XP_003121056.1| PREDICTED: uncharacterized protein FLJ39582-like [Homo sapiens]

Length=122

Score = 79.7 bits (195), Expect = 5e-16

Identities = 35/51 (69%), Positives = 39/51 (77%), Gaps = 0/51 (0%)

Frame = -1

Query 161 MKSHSVTQPGVQWCNLS*LQPPPPRFKQFFCLSLPSSWDYRHAPSCMANFC 9

+KSHSV Q GVQW +LS QPPPPRFKQF CLS+PSSWDYRH P + C

Sbjct 67 LKSHSVAQAGVQWHDLSSPQPPPPRFKQFSCLSIPSSWDYRHNPDKTSQHC 117

>ref|XP_003121047.1| PREDICTED: KN motif and ankyrin repeat domain-containing protein

3-like [Homo sapiens]

Length=143

Score = 79.3 bits (194), Expect = 7e-16

Identities = 36/52 (70%), Positives = 40/52 (77%), Gaps = 0/52 (0%)

Frame = -1

Query 158 KSHSVTQPGVQWCNLS*LQPPPPRFKQFFCLSLPSSWDYRHAPSCMANFCIF 3

+SHSV Q GV+W +L LQ PPPRFKQF LSL SSWD+RH P C ANFCIF

Sbjct 73 ESHSVAQAGVRWHDLGSLQSPPPRFKQFSYLSLLSSWDHRHTPPCPANFCIF 124

>ref|NP_872601.1| histone demethylase UTY isoform 1 [Homo sapiens]

Length=1079

Score = 77.4 bits (189), Expect = 3e-15

Identities = 32/44 (73%), Positives = 37/44 (85%), Gaps = 0/44 (0%)

Frame = -1

Query 134 GVQWCNLS*LQPPPPRFKQFFCLSLPSSWDYRHAPSCMANFCIF 3

G+QWC+LS LQPPPP FK+F LSLP+SW+YRH PSC NFCIF

Sbjct 997 GMQWCDLSSLQPPPPGFKRFSHLSLPNSWNYRHLPSCPTNFCIF 1040

>ref|XP_003118634.1| PREDICTED: putative uncharacterized protein NCRNA00269-like [Homo

sapiens]

ref|XP_003120632.1| PREDICTED: putative uncharacterized protein NCRNA00269-like [Homo

sapiens]

Length=140

Score = 75.5 bits (184), Expect = 1e-14

Identities = 39/54 (73%), Positives = 40/54 (75%), Gaps = 0/54 (0%)

Frame = -2

Query 163 R*SLTLSPSLEYSGAISADCNLHLPGSSNSSASASLVAGTIGMHHHAWLIFVFL 2

R SL L P LE G ISA CNLHLPGSS+ ASAS VAGT G HHAWLIFVFL

Sbjct 27 RQSLALLPKLECHGTISAHCNLHLPGSSDFPASASQVAGTTGACHHAWLIFVFL 80

>ref|XP_003120094.1| PREDICTED: putative uncharacterized protein NCRNA00269-like [Homo

sapiens]

ref|XP_003119097.1| PREDICTED: putative uncharacterized protein NCRNA00269-like [Homo

sapiens]

Length=140

Score = 75.5 bits (184), Expect = 1e-14

Identities = 39/54 (73%), Positives = 40/54 (75%), Gaps = 0/54 (0%)

Frame = -2

Query 163 R*SLTLSPSLEYSGAISADCNLHLPGSSNSSASASLVAGTIGMHHHAWLIFVFL 2

R SL L P LE G ISA CNLHLPGSS+ ASAS VAGT G HHAWLIFVFL

Sbjct 27 RQSLALLPKLECHGTISAHCNLHLPGSSDFPASASQVAGTTGACHHAWLIFVFL 80

>ref|XP_003119819.1| PREDICTED: putative uncharacterized protein C14orf165-like [Homo

sapiens]

ref|XP_003118891.1| PREDICTED: putative uncharacterized protein C14orf165-like [Homo

sapiens]

ref|XP_003120627.1| PREDICTED: putative uncharacterized protein C14orf165-like [Homo

sapiens]

Length=110

Score = 73.9 bits (180), Expect = 3e-14

Identities = 35/54 (65%), Positives = 39/54 (73%), Gaps = 0/54 (0%)

Frame = -1

Query 164 EMKSHSVTQPGVQWCNLS*LQPPPPRFKQFFCLSLPSSWDYRHAPSCMANFCIF 3

EM+ S Q G+QW +LS LQP PRFKQF CLSLPSS DYRH P + NFCIF

Sbjct 38 EMEFCSAAQAGMQWLSLSSLQPLHPRFKQFSCLSLPSSCDYRHVPPHLVNFCIF 91

>ref|NP_689672.4| hypothetical protein LOC146556 isoform 1 precursor [Homo sapiens]

Length=402

Score = 73.2 bits (178), Expect = 5e-14

Identities = 35/51 (69%), Positives = 36/51 (71%), Gaps = 0/51 (0%)

Frame = -1

Query 155 SHSVTQPGVQWCNLS*LQPPPPRFKQFFCLSLPSSWDYRHAPSCMANFCIF 3

S SV Q GVQW NL LQP PP FKQF CL LPSSWDYR P +ANF IF

Sbjct 320 SRSVAQAGVQWRNLGSLQPLPPGFKQFSCLILPSSWDYRSVPPYLANFYIF 370

>ref|NP_078926.3| putative uncharacterized protein C11orf80 [Homo sapiens]

Length=677

Score = 69.3 bits (168), Expect = 7e-13

Identities = 35/52 (68%), Positives = 37/52 (72%), Gaps = 0/52 (0%)

Frame = -2

Query 163 R*SLTLSPSLEYSGAISADCNLHLPGSSNSSASASLVAGTIGMHHHAWLIFV 8

R SLTL P E SGA+SA CNLHLPGSS+S AS VAG HHHAWLI V

Sbjct 106 RWSLTLLPRPECSGAVSAHCNLHLPGSSDSHASVPRVAGITDAHHHAWLIMV 157

>ref|XP_003119960.1| PREDICTED: putative uncharacterized protein NCRNA00269-like [Homo

sapiens]

ref|XP_003118569.1| PREDICTED: putative uncharacterized protein NCRNA00269-like [Homo

sapiens]

ref|XP_003120465.1| PREDICTED: putative uncharacterized protein NCRNA00269-like [Homo

sapiens]

Length=128

Score = 65.9 bits (159), Expect = 8e-12

Identities = 37/54 (69%), Positives = 38/54 (71%), Gaps = 0/54 (0%)

Frame = -2

Query 163 R*SLTLSPSLEYSGAISADCNLHLPGSSNSSASASLVAGTIGMHHHAWLIFVFL 2

R SL LSP LE SGAISA CNL L GSS S ASAS V+G G HHA L FVFL

Sbjct 25 RRSLALSPRLECSGAISAHCNLRLLGSSYSLASASRVSGITGSRHHAQLFFVFL 78

>ref|NP_001164252.1| hypothetical protein LOC159091 isoform 4 [Homo sapiens]

Length=98

Score = 65.5 bits (158), Expect = 1e-11

Identities = 35/49 (72%), Positives = 37/49 (76%), Gaps = 0/49 (0%)

Frame = -2

Query 157 SLTLSPSLEYSGAISADCNLHLPGSSNSSASASLVAGTIGMHHHAWLIF 11

SLT+SP LE SG ISA CNL LPGSS+S AS S VAGT G HHA LIF

Sbjct 39 SLTVSPRLECSGMISAHCNLCLPGSSDSPASDSRVAGTSGTRHHAHLIF 87

>ref|XP_003118843.1| PREDICTED: zinc finger protein ENSP00000375192-like [Homo sapiens]

ref|XP_003120983.1| PREDICTED: zinc finger protein ENSP00000375192-like [Homo sapiens]

Length=245

Score = 64.7 bits (156), Expect = 2e-11

Identities = 35/52 (68%), Positives = 37/52 (72%), Gaps = 0/52 (0%)

Frame = -2

Query 157 SLTLSPSLEYSGAISADCNLHLPGSSNSSASASLVAGTIGMHHHAWLIFVFL 2

SLTLSP LE +GAIS CNL L GSS+S AS S AG G HHA LIFVFL

Sbjct 106 SLTLSPKLECNGAISVHCNLRLLGSSDSLASTSQAAGIAGACHHAQLIFVFL 157

>ref|NP_001123498.2| hypothetical protein LOC285966 isoform B [Homo sapiens]

Length=815

Score = 59.7 bits (143), Expect(2) = 2e-11

Identities = 28/37 (76%), Positives = 29/37 (79%), Gaps = 0/37 (0%)

Frame = -2

Query 112 ADCNLHLPGSSNSSASASLVAGTIGMHHHAWLIFVFL 2

+ CNLHL G SNSS SAS VAGT G HHAWLIFVFL

Sbjct 390 SSCNLHLLGLSNSSLSASCVAGTTGTRHHAWLIFVFL 426

Score = 24.6 bits (52), Expect(2) = 2e-11

Identities = 10/16 (63%), Positives = 13/16 (82%), Gaps = 0/16 (0%)

Frame = -1

Query 158 KSHSVTQPGVQWCNLS 111

+SHSV Q G+QW +LS

Sbjct 375 ESHSVIQVGMQWRDLS 390

>ref|NP_001158011.1| disrupted in schizophrenia 1 protein isoform c [Homo sapiens]

Length=755

Score = 55.5 bits (132), Expect(2) = 5e-11

Identities = 24/32 (75%), Positives = 25/32 (79%), Gaps = 0/32 (0%)

Frame = -1

Query 107 LQPPPPRFKQFFCLSLPSSWDYRHAPSCMANF 12

LQP PP FKQF CLSL SSWDYR P C+ANF

Sbjct 679 LQPLPPEFKQFSCLSLRSSWDYRCPPPCLANF 710

Score = 27.7 bits (60), Expect(2) = 5e-11

Identities = 11/16 (69%), Positives = 12/16 (75%), Gaps = 0/16 (0%)

Frame = -3

Query 162 DEVSLCHPAWSTVVQS 115

D VSLC P WS VV+S

Sbjct 661 DGVSLCRPVWSAVVRS 676

>ref|XP_003120115.1| PREDICTED: putative uncharacterized protein NCRNA00269-like [Homo

sapiens]

ref|XP_003119111.1| PREDICTED: putative uncharacterized protein NCRNA00269-like [Homo

sapiens]

ref|XP_003118613.1| PREDICTED: putative uncharacterized protein NCRNA00269-like [Homo

sapiens]

ref|XP_003120653.1| PREDICTED: putative uncharacterized protein NCRNA00269-like [Homo

sapiens]

Length=137

Score = 62.4 bits (150), Expect = 9e-11

Identities = 37/55 (68%), Positives = 38/55 (70%), Gaps = 1/55 (1%)

Frame = -2

Query 163 R*SLTLSPSLEYSGAISADCNLHLPGSSNSSASASLVAGTIGMHHHAWLIF-VFL 2

R SLTLS LE SGAI A CNL L GS+ ASAS VAG G HHAWLIF VFL

Sbjct 29 RWSLTLSSRLECSGAILAHCNLRLLGSNEPPASASRVAGITGACHHAWLIFLVFL 83

>ref|NP_963998.2| thromboxane A2 receptor isoform beta [Homo sapiens]

Length=407

Score = 61.6 bits (148), Expect = 1e-10

Identities = 33/47 (71%), Positives = 35/47 (75%), Gaps = 0/47 (0%)

Frame = -2

Query 163 R*SLTLSPSLEYSGAISADCNLHLPGSSNSSASASLVAGTIGMHHHA 23

R SLTL PSLEYSG ISA CNL LPGSS+S ASAS AG G+ H A

Sbjct 328 RRSLTLWPSLEYSGTISAHCNLRLPGSSDSRASASRAAGITGVSHCA 374

>ref|XP_003119710.1| PREDICTED: hypothetical protein LOC100507131 [Homo sapiens]

Length=165

Score = 60.8 bits (146), Expect = 2e-10

Identities = 31/54 (58%), Positives = 35/54 (65%), Gaps = 0/54 (0%)

Frame = -1

Query 164 EMKSHSVTQPGVQWCNLS*LQPPPPRFKQFFCLSLPSSWDYRHAPSCMANFCIF 3

EM+S S Q GVQW +L LQ PRFK+ CLSL SSW YR P ANFC+F

Sbjct 17 EMESRSGAQVGVQWRDLGSLQLSSPRFKRLSCLSLLSSWYYRCPPPQPANFCVF 70

>ref|XP_003119783.1| PREDICTED: serine/threonine-protein phosphatase 5-like [Homo

sapiens]

ref|XP_003118828.1| PREDICTED: serine/threonine-protein phosphatase 5-like [Homo

sapiens]

ref|XP_003120999.1| PREDICTED: serine/threonine-protein phosphatase 5-like [Homo

sapiens]

Length=171

Score = 60.8 bits (146), Expect = 2e-10

Identities = 30/51 (59%), Positives = 36/51 (71%), Gaps = 0/51 (0%)

Frame = -2

Query 154 LTLSPSLEYSGAISADCNLHLPGSSNSSASASLVAGTIGMHHHAWLIFVFL 2

L LSP L+ SG I+A C+L+L G + SAS VA T GMHHH WLIF+FL

Sbjct 121 LALSPRLKCSGTITAHCSLNLLGPRDPPTSASQVAVTEGMHHHTWLIFLFL 171

>ref|XP_003118780.1| PREDICTED: hypothetical protein LOC100507131 [Homo sapiens]

ref|XP_003120912.1| PREDICTED: hypothetical protein LOC100507131 [Homo sapiens]

Length=165

Score = 60.8 bits (146), Expect = 2e-10

Identities = 31/54 (58%), Positives = 35/54 (65%), Gaps = 0/54 (0%)

Frame = -1

Query 164 EMKSHSVTQPGVQWCNLS*LQPPPPRFKQFFCLSLPSSWDYRHAPSCMANFCIF 3

EM+S S Q GVQW +L LQ PRFK+ CLSL SSW YR P ANFC+F

Sbjct 17 EMESRSGAQVGVQWRDLGSLQLSSPRFKRLSCLSLLSSWYYRCPPPQPANFCVF 70

>ref|NP_862828.1| zinc finger protein 283 [Homo sapiens]

Length=679

Score = 60.1 bits (144), Expect = 4e-10

Identities = 30/51 (59%), Positives = 33/51 (65%), Gaps = 3/51 (5%)

Frame = -1

Query 161 MKSHSVTQPGVQWCNLS*LQPPPPRFKQFFCLSLPSSWDYRHAPSCMANFC 9

M+S SV Q GVQWC+L LQ PPP F F CLSL SSWDY S + FC

Sbjct 1 MESRSVAQAGVQWCDLGSLQAPPPGFTLFSCLSLLSSWDY---SSGFSGFC 48

>ref|XP_003119248.1| PREDICTED: hypothetical protein LOC100506511 [Homo sapiens]

Length=402

Score = 58.5 bits (140), Expect = 1e-09

Identities = 28/45 (63%), Positives = 32/45 (72%), Gaps = 0/45 (0%)

Frame = -1

Query 140 QPGVQWCNLS*LQPPPPRFKQFFCLSLPSSWDYRHAPSCMANFCI 6

Q GV+ +L LQP PP FK+F CLSLPSSW Y HAPS ANF +

Sbjct 308 QAGVRCRDLDSLQPLPPGFKRFSCLSLPSSWGYSHAPSRPANFVV 352

>ref|XP_003119846.1| PREDICTED: hypothetical protein LOC100507929 [Homo sapiens]

ref|XP_003119023.1| PREDICTED: hypothetical protein LOC100507664 [Homo sapiens]

ref|XP_003120394.1| PREDICTED: hypothetical protein LOC100507929 [Homo sapiens]

Length=122

Score = 58.5 bits (140), Expect = 1e-09

Identities = 31/54 (58%), Positives = 37/54 (69%), Gaps = 0/54 (0%)

Frame = +3

Query 3 KNTKISHA*WCMPIVPATREAEAEELLEPGRWRLQSAEIAPLYSRLGDRVRLHL 164

KNTKIS W +P++P T EAEA E LEPG+ LQ I L+S LG+RVRL L

Sbjct 52 KNTKISWVWWQVPVIPTTWEAEAGESLEPGKSSLQRTMILTLHSSLGNRVRLCL 105

>ref|NP_001171696.1| BEN domain-containing protein 2 isoform 2 [Homo sapiens]

Length=645

Score = 57.4 bits (137), Expect = 3e-09

Identities = 29/44 (66%), Positives = 30/44 (69%), Gaps = 0/44 (0%)

Frame = -1

Query 155 SHSVTQPGVQWCNLS*LQPPPPRFKQFFCLSLPSSWDYRHAPSC 24

S SVTQ GVQW + S LQP P KQFF LSLPSSWD R P C

Sbjct 81 SGSVTQAGVQWHDHSSLQPQPLGLKQFFHLSLPSSWDDRRTPPC 124

>ref|NP_699177.2| BEN domain-containing protein 2 isoform 1 [Homo sapiens]

Length=799

Score = 57.4 bits (137), Expect = 3e-09

Identities = 29/44 (66%), Positives = 30/44 (69%), Gaps = 0/44 (0%)

Frame = -1

Query 155 SHSVTQPGVQWCNLS*LQPPPPRFKQFFCLSLPSSWDYRHAPSC 24

S SVTQ GVQW + S LQP P KQFF LSLPSSWD R P C

Sbjct 81 SGSVTQAGVQWHDHSSLQPQPLGLKQFFHLSLPSSWDDRRTPPC 124

>ref|XP_003120922.1| PREDICTED: putative uncharacterized protein NCRNA00269-like [Homo

sapiens]

Length=127

Score = 57.0 bits (136), Expect = 4e-09

Identities = 28/36 (78%), Positives = 30/36 (84%), Gaps = 0/36 (0%)

Frame = -2

Query 154 LTLSPSLEYSGAISADCNLHLPGSSNSSASASLVAG 47

LTLSP LEYSG SA C+LHLPGSSN+ ASAS VAG

Sbjct 77 LTLSPRLEYSGTTSAHCSLHLPGSSNAPASASRVAG 112

Score = 40.8 bits (94), Expect = 3e-04

Identities = 22/40 (55%), Positives = 27/40 (68%), Gaps = 0/40 (0%)

Frame = -2

Query 154 LTLSPSLEYSGAISADCNLHLPGSSNSSASASLVAGTIGM 35

L LS +E+SG I A +L LPGSSN + SA VAGT G+

Sbjct 38 LALSLRMEFSGPIMAHYSLKLPGSSNPTMSAFPVAGTTGL 77

>ref|NP_114174.1| nuclear prelamin A recognition factor isoform b [Homo sapiens]

Length=502

Score = 57.0 bits (136), Expect = 4e-09

Identities = 28/45 (63%), Positives = 33/45 (74%), Gaps = 0/45 (0%)

Frame = +3

Query 9 TKISHA*WCMPIVPATREAEAEELLEPGRWRLQSAEIAPLYSRLG 143

++IS A WC P++ ATREA A E LEPGR RLQ +IAPL S LG

Sbjct 256 SEISQAWWCTPVITATREAAARESLEPGRQRLQRDKIAPLDSSLG 300

>ref|XP_003119959.1| PREDICTED: hypothetical protein LOC100287290 [Homo sapiens]

ref|XP_002342446.2| PREDICTED: putative uncharacterized protein C3orf66 [Homo sapiens]

ref|XP_002346604.2| PREDICTED: putative uncharacterized protein C3orf66 [Homo sapiens]

Length=70

Score = 56.6 bits (135), Expect = 5e-09

Identities = 25/41 (61%), Positives = 31/41 (76%), Gaps = 4/41 (9%)

Frame = -1

Query 161 MKSHSVTQPGVQWCNLS*LQPPPPRFKQFFCLSLPSSWDYR 39

M+SH+VT+ G+QWC+L PPRFK+F CL LPSSWD R

Sbjct 1 MESHTVTRAGMQWCDLG----SPPRFKRFCCLCLPSSWDSR 37

>ref|NP_001155002.1| granulocyte-macrophage colony-stimulating factor receptor subunit

alpha isoform f precursor [Homo sapiens]

Length=434

Score = 56.2 bits (134), Expect = 6e-09

Identities = 27/40 (68%), Positives = 28/40 (70%), Gaps = 0/40 (0%)

Frame = -1

Query 164 EMKSHSVTQPGVQWCNLS*LQPPPPRFKQFFCLSLPSSWD 45

E SHSVTQ GVQW NL LQPP PR K+F CL LP S D

Sbjct 314 EFGSHSVTQAGVQWHNLGSLQPPSPRLKRFSCLRLPGSDD 353

>ref|NP_001077368.1| platelet glycoprotein VI isoform 1 [Homo sapiens]

Length=620

Score = 54.7 bits (130), Expect = 2e-08

Identities = 30/53 (57%), Positives = 35/53 (67%), Gaps = 0/53 (0%)

Frame = +1

Query 4 KIQKLAMHDGACR*SQLLGRLRQKNCLNLGGGGCSQLRLHHCTPGWVTE*DFI 162

K QKLA GA SQ L LR +N L+LGG GCS+LR HHCT VT+ DF+

Sbjct 555 KTQKLARCGGASLYSQQLRGLRWENGLSLGGRGCSELRSHHCTLARVTKPDFV 607

>ref|XP_003119678.1| PREDICTED: hypothetical protein LOC100506928 [Homo sapiens]

ref|XP_003118761.1| PREDICTED: hypothetical protein LOC100506928 [Homo sapiens]

ref|XP_003120899.1| PREDICTED: hypothetical protein LOC100506928 [Homo sapiens]

Length=159

Score = 54.3 bits (129), Expect = 2e-08

Identities = 29/49 (60%), Positives = 32/49 (66%), Gaps = 0/49 (0%)

Frame = -2

Query 163 R*SLTLSPSLEYSGAISADCNLHLPGSSNSSASASLVAGTIGMHHHAWL 17

R LTL P LE SG I+A +L LPGSSN SAS +GT GM HH WL

Sbjct 105 REGLTLLPRLECSGMITAHYSLGLPGSSNPPTSASQGSGTTGMCHHTWL 153

>ref|NP_115861.1| peptidyl-prolyl cis-trans isomerase-like 3 isoform PPIL3a [Homo

sapiens]

Length=165

Score = 54.3 bits (129), Expect = 2e-08

Identities = 25/36 (70%), Positives = 27/36 (75%), Gaps = 0/36 (0%)

Frame = -1

Query 164 EMKSHSVTQPGVQWCNLS*LQPPPPRFKQFFCLSLP 57

EM+S V Q GVQW +L LQPPPP FKQ FCLSLP

Sbjct 26 EMESRCVPQAGVQWRDLGSLQPPPPGFKQVFCLSLP 61

>ref|NP_689573.3| zinc finger protein 573 isoform 1 [Homo sapiens]

Length=607

Score = 52.0 bits (123), Expect = 1e-07

Identities = 25/34 (74%), Positives = 27/34 (80%), Gaps = 0/34 (0%)

Frame = -1

Query 161 MKSHSVTQPGVQWCNLS*LQPPPPRFKQFFCLSL 60

M+S SV Q GVQW +LS LQPPPPRFKQF C SL

Sbjct 1 MESCSVAQAGVQWPDLSSLQPPPPRFKQFSCHSL 34

>ref|NP_001180462.1| serine/threonine-protein kinase Nek4 isoform 2 [Homo sapiens]

Length=752

Score = 52.0 bits (123), Expect = 1e-07

Identities = 29/45 (65%), Positives = 31/45 (69%), Gaps = 0/45 (0%)

Frame = -2

Query 157 SLTLSPSLEYSGAISADCNLHLPGSSNSSASASLVAGTIGMHHHA 23

SL LSP LE SG I A NL L GSS+S ASAS VAG G+ HHA

Sbjct 368 SLALSPKLECSGTILAHSNLRLLGSSDSPASASRVAGITGVCHHA 412

>ref|NP_003148.2| serine/threonine-protein kinase Nek4 isoform 1 [Homo sapiens]

Length=841

Score = 52.0 bits (123), Expect = 1e-07

Identities = 29/45 (65%), Positives = 31/45 (69%), Gaps = 0/45 (0%)

Frame = -2

Query 157 SLTLSPSLEYSGAISADCNLHLPGSSNSSASASLVAGTIGMHHHA 23

SL LSP LE SG I A NL L GSS+S ASAS VAG G+ HHA

Sbjct 457 SLALSPKLECSGTILAHSNLRLLGSSDSPASASRVAGITGVCHHA 501

>ref|XP_003119048.1| PREDICTED: hypothetical protein LOC100506073 [Homo sapiens]

ref|XP_003120529.1| PREDICTED: hypothetical protein LOC100506073 [Homo sapiens]

Length=190

Score = 51.6 bits (122), Expect = 2e-07

Identities = 26/45 (58%), Positives = 31/45 (69%), Gaps = 1/45 (2%)

Frame = -1

Query 134 GVQWCNLS*LQPPPPRFKQFFCLSLPSSWDYRHA-PSCMANFCIF 3

G+QW +L LQP PP FK+F CLSL SSWD R+ P + FCIF

Sbjct 35 GMQWLHLCSLQPLPPGFKRFSCLSLLSSWDCRYTLPFPVNVFCIF 79

>ref|NP_001120653.1| centromere protein L isoform 1 [Homo sapiens]

Length=390

Score = 51.6 bits (122), Expect = 2e-07

Identities = 27/47 (58%), Positives = 30/47 (64%), Gaps = 0/47 (0%)

Frame = -2

Query 154 LTLSPSLEYSGAISADCNLHLPGSSNSSASASLVAGTIGMHHHAWLI 14

L LSP LEYSG I DCNL L GSS+ S A VAGT G HH ++

Sbjct 142 LILSPRLEYSGTILVDCNLCLLGSSDPSTLAFQVAGTAGACHHTRIV 188

>ref|XP_003118557.1| PREDICTED: histone demethylase UTY-like [Homo sapiens]

Length=101

Score = 51.2 bits (121), Expect = 2e-07

Identities = 28/50 (56%), Positives = 30/50 (60%), Gaps = 0/50 (0%)

Frame = -1

Query 161 MKSHSVTQPGVQWCNLS*LQPPPPRFKQFFCLSLPSSWDYRHAPSCMANF 12

M+S SV GVQW NLS LQPPP FK F LSL SS D R C+ F

Sbjct 5 MESCSVPHAGVQWHNLSSLQPPPSGFKPFSYLSLLSSRDQRRPLPCLVTF 54

>ref|NP_001185728.1| activating signal cointegrator 1 complex subunit 1 isoform a

[Homo sapiens]

Length=400

Score = 51.2 bits (121), Expect = 2e-07

Identities = 27/41 (66%), Positives = 30/41 (74%), Gaps = 0/41 (0%)

Frame = -2

Query 157 SLTLSPSLEYSGAISADCNLHLPGSSNSSASASLVAGTIGM 35

S L P LEY+ AISA CNL LPGSS+S ASAS VAG G+

Sbjct 348 SFALLPRLEYNDAISAHCNLCLPGSSDSPASASQVAGITGV 388

>ref|NP_056087.2| protein fantom isoform a [Homo sapiens]

Length=1315

Score = 50.1 bits (118), Expect = 4e-07

Identities = 27/44 (62%), Positives = 28/44 (64%), Gaps = 0/44 (0%)

Frame = -2

Query 157 SLTLSPSLEYSGAISADCNLHLPGSSNSSASASLVAGTIGMHHH 26

SL LSP L S AISA CN LPGSS+ ASAS V G G HH

Sbjct 1099 SLALSPGLGCSSAISAHCNFRLPGSSDFPASASQVDGITGACHH 1142

>ref|XP_003120008.1| PREDICTED: putative uncharacterized protein NCRNA00269-like [Homo

sapiens]

ref|XP_003118926.1| PREDICTED: putative uncharacterized protein NCRNA00269-like [Homo

sapiens]

ref|XP_003120534.1| PREDICTED: putative uncharacterized protein NCRNA00269-like [Homo

sapiens]

Length=123

Score = 49.7 bits (117), Expect = 6e-07

Identities = 27/39 (70%), Positives = 29/39 (75%), Gaps = 0/39 (0%)

Frame = -2

Query 118 ISADCNLHLPGSSNSSASASLVAGTIGMHHHAWLIFVFL 2

ISA +LHLPGSS S ASAS +AG M HHA LIFVFL

Sbjct 2 ISAHGSLHLPGSSYSPASASQIAGITVMCHHAGLIFVFL 40

>ref|NP_001138489.1| proton-coupled amino acid transporter 3 isoform 1 [Homo sapiens]

Length=511

Score = 49.7 bits (117), Expect = 6e-07

Identities = 27/39 (70%), Positives = 28/39 (72%), Gaps = 0/39 (0%)

Frame = -2

Query 163 R*SLTLSPSLEYSGAISADCNLHLPGSSNSSASASLVAG 47

R +L LSP LE SG ISA CN HL GSSNS A AS VAG

Sbjct 135 RWNLALSPRLECSGKISAHCNPHLQGSSNSPAQASRVAG 173

>ref|NP_009112.1| mitogen-activated protein kinase kinase kinase kinase 1 isoform

2 [Homo sapiens]

Length=833

Score = 49.7 bits (117), Expect = 6e-07

Identities = 26/37 (71%), Positives = 27/37 (73%), Gaps = 0/37 (0%)

Frame = -2

Query 145 SPSLEYSGAISADCNLHLPGSSNSSASASLVAGTIGM 35

SP LE SG IS CNL LPGSSNS ASAS VAG G+

Sbjct 797 SPRLECSGTISPHCNLLLPGSSNSPASASRVAGITGL 833

>ref|XP_003119925.1| PREDICTED: histone demethylase UTY-like [Homo sapiens]

ref|XP_003120437.1| PREDICTED: histone demethylase UTY-like [Homo sapiens]

Length=101

Score = 49.3 bits (116), Expect = 7e-07

Identities = 27/50 (54%), Positives = 29/50 (58%), Gaps = 0/50 (0%)

Frame = -1

Query 161 MKSHSVTQPGVQWCNLS*LQPPPPRFKQFFCLSLPSSWDYRHAPSCMANF 12

M+S SV GVQW NLS LQPPP FK F LSL S D R C+ F

Sbjct 5 MESCSVPHAGVQWHNLSSLQPPPSGFKPFSYLSLLRSRDQRRPLPCLVTF 54

>ref|NP_001011657.2| zinc finger matrin-type protein 1 isoform 1 [Homo sapiens]

Length=638

Score = 48.9 bits (115), Expect = 1e-06

Identities = 24/31 (78%), Positives = 27/31 (88%), Gaps = 0/31 (0%)

Frame = -2

Query 136 LEYSGAISADCNLHLPGSSNSSASASLVAGT 44

LE SGAISA C+LHLPGSS+S ASAS +AGT

Sbjct 9 LECSGAISAHCSLHLPGSSDSPASASQIAGT 39

>ref|NP_150646.3| alpha-1A adrenergic receptor isoform 2 [Homo sapiens]

Length=475

Score = 47.8 bits (112), Expect = 2e-06

Identities = 25/38 (66%), Positives = 26/38 (69%), Gaps = 0/38 (0%)

Frame = -2

Query 136 LEYSGAISADCNLHLPGSSNSSASASLVAGTIGMHHHA 23

LE SG I A CNL LPGS +S ASAS AGT GM H A

Sbjct 432 LECSGMILAHCNLRLPGSRDSPASASQAAGTTGMCHQA 469

>ref|NP_001009923.1| hypothetical protein LOC29058 isoform 1 [Homo sapiens]

Length=183

Score = 47.8 bits (112), Expect = 2e-06

Identities = 27/43 (63%), Positives = 30/43 (70%), Gaps = 0/43 (0%)

Frame = -2

Query 163 R*SLTLSPSLEYSGAISADCNLHLPGSSNSSASASLVAGTIGM 35

R SL LSP LE SG ISA CNLHL SS+SSASAS + + M

Sbjct 23 RWSLVLSPRLEPSGVISAHCNLHLLASSDSSASASRLCQRVMM 65

>ref|NP_001182556.1| protein AF-10 isoform d [Homo sapiens]

Length=126

Score = 47.4 bits (111), Expect = 3e-06

Identities = 22/39 (57%), Positives = 27/39 (70%), Gaps = 0/39 (0%)

Frame = -1

Query 158 KSHSVTQPGVQWCNLS*LQPPPPRFKQFFCLSLPSSWDY 42

+S SV Q VQWC+LS LQP P FK+F CLSLP+ +

Sbjct 82 ESRSVAQAKVQWCDLSPLQPLLPGFKRFSCLSLPNGMQF 120

>ref|XP_003119709.1| PREDICTED: hypothetical protein LOC100509912 [Homo sapiens]

ref|XP_003118779.1| PREDICTED: hypothetical protein LOC100506809 [Homo sapiens]

ref|XP_003120910.1| PREDICTED: hypothetical protein LOC100509912 [Homo sapiens]

Length=118

Score = 47.4 bits (111), Expect = 3e-06

Identities = 24/35 (69%), Positives = 27/35 (78%), Gaps = 1/35 (2%)

Frame = +3

Query 3 KNTKISHA*WCMPIVPATREAEAEELLEPGRWRLQ 107

KNTKIS A W +P++PA RE EA E LEPGR RLQ

Sbjct 85 KNTKISWA-WWVPVIPAIREGEAGESLEPGRQRLQ 118

>ref|NP_001137385.1| hypothetical protein LOC199870 isoform 2 [Homo sapiens]

Length=312

Score = 47.4 bits (111), Expect = 3e-06

Identities = 23/34 (68%), Positives = 27/34 (80%), Gaps = 0/34 (0%)

Frame = -2

Query 136 LEYSGAISADCNLHLPGSSNSSASASLVAGTIGM 35

LE +G ISA CNLHLPGSS+S AS+S VAG G+

Sbjct 50 LECNGTISAHCNLHLPGSSDSPASSSRVAGITGI 83

>ref|NP_001137384.1| hypothetical protein LOC199870 isoform 1 [Homo sapiens]

Length=341

Score = 47.4 bits (111), Expect = 3e-06

Identities = 23/34 (68%), Positives = 27/34 (80%), Gaps = 0/34 (0%)

Frame = -2

Query 136 LEYSGAISADCNLHLPGSSNSSASASLVAGTIGM 35

LE +G ISA CNLHLPGSS+S AS+S VAG G+

Sbjct 50 LECNGTISAHCNLHLPGSSDSPASSSRVAGITGI 83

>ref|XP_003120664.1| PREDICTED: hypothetical protein LOC100510512 [Homo sapiens]

Length=177

Score = 47.0 bits (110), Expect = 4e-06

Identities = 28/51 (55%), Positives = 30/51 (59%), Gaps = 0/51 (0%)

Frame = -2

Query 163 R*SLTLSPSLEYSGAISADCNLHLPGSSNSSASASLVAGTIGMHHHAWLIF 11

R L L LE SG I C+L L GS +S SAS VAGT GMH HA L F

Sbjct 111 RQGLALLSRLECSGMIITYCSLKLLGSRDSPVSASQVAGTTGMHQHARLNF 161

>ref|XP_003120124.1| PREDICTED: hypothetical protein LOC100507236, partial [Homo sapiens]

ref|XP_003119212.1| PREDICTED: hypothetical protein LOC100507236, partial [Homo sapiens]

Length=174

Score = 47.0 bits (110), Expect = 4e-06

Identities = 28/51 (55%), Positives = 30/51 (59%), Gaps = 0/51 (0%)

Frame = -2

Query 163 R*SLTLSPSLEYSGAISADCNLHLPGSSNSSASASLVAGTIGMHHHAWLIF 11

R L L LE SG I C+L L GS +S SAS VAGT GMH HA L F

Sbjct 111 RQGLALLSRLECSGMIITYCSLKLLGSRDSPVSASQVAGTTGMHQHARLNF 161

>ref|XP_003120111.1| PREDICTED: putative uncharacterized protein NCRNA00269-like [Homo

sapiens]

ref|XP_003119110.1| PREDICTED: putative uncharacterized protein NCRNA00269-like [Homo

sapiens]

ref|XP_003118626.1| PREDICTED: putative uncharacterized protein NCRNA00269-like [Homo

sapiens]

ref|XP_003120652.1| PREDICTED: putative uncharacterized protein NCRNA00269-like [Homo

sapiens]

Length=130

Score = 47.0 bits (110), Expect = 4e-06

Identities = 27/54 (50%), Positives = 36/54 (67%), Gaps = 0/54 (0%)

Frame = -2

Query 163 R*SLTLSPSLEYSGAISADCNLHLPGSSNSSASASLVAGTIGMHHHAWLIFVFL 2

R SLTL P LE GAI A +L LPG +S AS S ++G + + H++ LIFVF+

Sbjct 31 RQSLTLLPRLECGGAILAHYSLCLPGLRDSLASVSQLSGIMCVRHYSRLIFVFV 84

>ref|NP_078841.3| cyclin-J-like protein [Homo sapiens]

Length=435

Score = 47.0 bits (110), Expect = 4e-06

Identities = 23/29 (80%), Positives = 24/29 (83%), Gaps = 0/29 (0%)

Frame = -2

Query 148 LSPSLEYSGAISADCNLHLPGSSNSSASA 62

LSP L+ SG ISA CNLHLPGSSNS ASA

Sbjct 99 LSPRLKCSGMISAHCNLHLPGSSNSPASA 127

>ref|NP_001108224.1| complement decay-accelerating factor isoform 2 precursor [Homo

sapiens]

Length=440

Score = 46.6 bits (109), Expect = 5e-06

Identities = 22/39 (57%), Positives = 26/39 (67%), Gaps = 0/39 (0%)

Frame = -1

Query 155 SHSVTQPGVQWCNLS*LQPPPPRFKQFFCLSLPSSWDYR 39

S VTQ G++WC+ S LQ P FK+ F SLPSSW YR

Sbjct 362 SRPVTQAGMRWCDRSSLQSRTPGFKRSFHFSLPSSWYYR 400

>ref|NP_997719.2| methyltransferase-like protein 10 [Homo sapiens]

Length=291

Score = 42.4 bits (98), Expect(2) = 6e-06

Identities = 18/24 (75%), Positives = 19/24 (80%), Gaps = 0/24 (0%)

Frame = -2

Query 73 SASASLVAGTIGMHHHAWLIFVFL 2

S SAS V GT G HHHAW+IFVFL

Sbjct 228 STSASRVGGTTGTHHHAWIIFVFL 251

Score = 23.5 bits (49), Expect(2) = 6e-06

Identities = 12/20 (60%), Positives = 12/20 (60%), Gaps = 0/20 (0%)

Frame = -3

Query 135 WSTVVQSQLTATSTSQVQAI 76

WSTV LTA TS QAI

Sbjct 207 WSTVAGFWLTAALTSWAQAI 226

>ref|NP_714912.1| interleukin-12 receptor subunit beta-1 isoform 2 precursor [Homo

sapiens]

Length=381

Score = 46.2 bits (108), Expect = 6e-06

Identities = 24/40 (60%), Positives = 26/40 (65%), Gaps = 0/40 (0%)

Frame = -2

Query 124 GAISADCNLHLPGSSNSSASASLVAGTIGMHHHAWLIFVF 5

G ISA CNL LP S +S ASAS VAG G+ HH LI F

Sbjct 342 GMISAHCNLRLPDSRDSPASASRVAGITGICHHTRLILYF 381

>ref|XP_003118848.1| PREDICTED: hypothetical protein LOC100130156 [Homo sapiens]

Length=147

Score = 45.4 bits (106), Expect = 1e-05

Identities = 21/33 (64%), Positives = 24/33 (73%), Gaps = 0/33 (0%)

Frame = -3

Query 162 DEVSLCHPAWSTVVQSQLTATSTSQVQAILLPQ 64

+ VSLC P WS V QLTAT+ S +QAILLPQ

Sbjct 111 ERVSLCCPGWSAVAPPQLTATTASWIQAILLPQ 143

>ref|XP_003119870.1| PREDICTED: hypothetical protein LOC100130156 [Homo sapiens]

ref|XP_003121031.1| PREDICTED: hypothetical protein LOC100130156 [Homo sapiens]

Length=147

Score = 45.4 bits (106), Expect = 1e-05

Identities = 21/33 (64%), Positives = 24/33 (73%), Gaps = 0/33 (0%)

Frame = -3

Query 162 DEVSLCHPAWSTVVQSQLTATSTSQVQAILLPQ 64

+ VSLC P WS V QLTAT+ S +QAILLPQ

Sbjct 111 ERVSLCCPGWSAVAPPQLTATTASWIQAILLPQ 143

>ref|XP_003119972.1| PREDICTED: hypothetical protein LOC100508228 [Homo sapiens]

ref|XP_003118923.1| PREDICTED: hypothetical protein LOC100506486 [Homo sapiens]

ref|XP_003120486.1| PREDICTED: hypothetical protein LOC100508228 [Homo sapiens]

Length=125

Score = 45.1 bits (105), Expect = 1e-05

Identities = 21/28 (75%), Positives = 23/28 (83%), Gaps = 0/28 (0%)

Frame = -1

Query 164 EMKSHSVTQPGVQWCNLS*LQPPPPRFK 81

EM+SHSVTQ GVQW +LS LQP PP FK

Sbjct 89 EMESHSVTQGGVQWHDLSSLQPQPPGFK 116

>ref|NP_001128626.1| zinc transporter ZIP14 isoform c [Homo sapiens]

Length=481

Score = 45.1 bits (105), Expect = 1e-05

Identities = 22/37 (60%), Positives = 26/37 (71%), Gaps = 0/37 (0%)

Frame = -1

Query 161 MKSHSVTQPGVQWCNLS*LQPPPPRFKQFFCLSLPSS 51

M+ SV Q GVQWC+LS LQP P K+ CLSLPS+

Sbjct 445 MEFCSVAQAGVQWCHLSSLQPLPLGLKRLSCLSLPSN 481

>ref|NP_001165113.1| myosin-IIIb isoform 3 [Homo sapiens]

Length=1275

Score = 44.7 bits (104), Expect = 2e-05

Identities = 25/42 (60%), Positives = 28/42 (67%), Gaps = 0/42 (0%)

Frame = -2

Query 157 SLTLSPSLEYSGAISADCNLHLPGSSNSSASASLVAGTIGMH 32

S TL LE + ISADCNL GSS+S ASAS VAG G+H

Sbjct 1194 SFTLLLRLECNSMISADCNLRPLGSSDSPASASRVAGITGIH 1235

>ref|NP_149084.2| tripartite motif-containing protein 5 isoform delta [Homo sapiens]

Length=326

Score = 44.7 bits (104), Expect = 2e-05

Identities = 19/26 (74%), Positives = 23/26 (89%), Gaps = 0/26 (0%)

Frame = -3

Query 135 WSTVVQSQLTATSTSQVQAILLPQPP 58

WS + +S+ TATSTSQ+QAILLPQPP

Sbjct 300 WSAMARSRFTATSTSQIQAILLPQPP 325

>ref|NP_612412.2| myosin regulatory light chain 10 [Homo sapiens]

Length=226

Score = 44.7 bits (104), Expect = 2e-05

Identities = 24/33 (73%), Positives = 25/33 (76%), Gaps = 0/33 (0%)

Frame = -2

Query 157 SLTLSPSLEYSGAISADCNLHLPGSSNSSASAS 59

SL LSP LE +G ISA CNL L GSSNS ASAS

Sbjct 58 SLALSPRLERNGMISAHCNLCLTGSSNSPASAS 90

>ref|XP_003119660.1| PREDICTED: hypothetical protein LOC100506579 [Homo sapiens]

ref|XP_003118706.1| PREDICTED: hypothetical protein LOC100506579 [Homo sapiens]

ref|XP_003120865.1| PREDICTED: hypothetical protein LOC100510190 [Homo sapiens]

Length=147

Score = 43.9 bits (102), Expect = 3e-05

Identities = 20/34 (59%), Positives = 22/34 (65%), Gaps = 0/34 (0%)

Frame = -3

Query 159 EVSLCHPAWSTVVQSQLTATSTSQVQAILLPQPP 58

+V LCHP W+TV SQLT SQ Q IL QPP

Sbjct 30 QVLLCHPGWNTVASSQLTVDLDSQAQVILPLQPP 63

>ref|NP_001153585.1| hypothetical protein LOC123207 isoform b [Homo sapiens]

Length=167

Score = 43.5 bits (101), Expect = 4e-05

Identities = 23/35 (66%), Positives = 25/35 (72%), Gaps = 0/35 (0%)

Frame = -2

Query 163 R*SLTLSPSLEYSGAISADCNLHLPGSSNSSASAS 59

R +L LSP LE SG + A CNLHL GSS S ASAS

Sbjct 133 RQNLALSPKLECSGVVLAHCNLHLLGSSYSPASAS 167

>ref|NP_874362.3| ankyrin repeat and death domain-containing protein 1A [Homo sapiens]

Length=522

Score = 43.5 bits (101), Expect = 4e-05

Identities = 19/26 (74%), Positives = 23/26 (89%), Gaps = 0/26 (0%)

Frame = -3

Query 135 WSTVVQSQLTATSTSQVQAILLPQPP 58

WST+ +SQLTATS S+VQ IL+PQPP

Sbjct 496 WSTMARSQLTATSASRVQMILVPQPP 521

>ref|NP_001003690.1| MAD2L1-binding protein isoform 1 [Homo sapiens]

Length=306

Score = 43.5 bits (101), Expect = 4e-05

Identities = 24/41 (59%), Positives = 28/41 (69%), Gaps = 0/41 (0%)

Frame = -2

Query 157 SLTLSPSLEYSGAISADCNLHLPGSSNSSASASLVAGTIGM 35

SLTLSP LE++G SA N LPGS +S ASAS VA I +

Sbjct 9 SLTLSPRLEHNGMTSAHHNFRLPGSRDSPASASQVAEIIDL 49

>ref|XP_003119509.1| PREDICTED: hypothetical protein LOC100507445 [Homo sapiens]

ref|XP_003118667.1| PREDICTED: hypothetical protein LOC100507445 [Homo sapiens]

ref|XP_003120698.1| PREDICTED: hypothetical protein LOC100507445 [Homo sapiens]

Length=121

Score = 43.5 bits (101), Expect = 4e-05

Identities = 21/35 (60%), Positives = 22/35 (63%), Gaps = 0/35 (0%)

Frame = +3

Query 3 KNTKISHA*WCMPIVPATREAEAEELLEPGRWRLQ 107

KN IS W P+VPA E EA E LEP RWRLQ

Sbjct 87 KNINISRVWWQAPVVPAIWETEAGESLEPRRWRLQ 121

>ref|NP_683685.1| 39S ribosomal protein L10, mitochondrial isoform b [Homo sapiens]

Length=271

Score = 42.7 bits (99), Expect = 7e-05

Identities = 21/27 (78%), Positives = 22/27 (82%), Gaps = 0/27 (0%)

Frame = -2

Query 118 ISADCNLHLPGSSNSSASASLVAGTIG 38

ISA CNLHLPGSS+S ASAS VAG G

Sbjct 2 ISAHCNLHLPGSSDSPASASQVAGITG 28

>ref|NP_777547.1| intraflagellar transport protein 20 homolog [Homo sapiens]

Length=148

Score = 42.0 bits (97), Expect = 1e-04

Identities = 23/40 (58%), Positives = 26/40 (65%), Gaps = 0/40 (0%)

Frame = -2

Query 157 SLTLSPSLEYSGAISADCNLHLPGSSNSSASASLVAGTIG 38

SL +SP LE +GAISA C L L SS+S S S V GT G

Sbjct 72 SLAVSPRLECTGAISAHCKLCLSDSSDSPTSPSRVGGTTG 111

>ref|NP_001139736.1| synaptotagmin-14 isoform 2 [Homo sapiens]

Length=600

Score = 41.6 bits (96), Expect = 2e-04

Identities = 21/31 (68%), Positives = 23/31 (75%), Gaps = 0/31 (0%)

Frame = -2

Query 136 LEYSGAISADCNLHLPGSSNSSASASLVAGT 44

LEYSG I A CN L GS++SSASAS V GT

Sbjct 34 LEYSGTILAHCNFRLLGSNDSSASASQVTGT 64

>ref|NP_001139733.1| synaptotagmin-14 isoform 1 [Homo sapiens]

Length=619

Score = 41.6 bits (96), Expect = 2e-04

Identities = 21/31 (68%), Positives = 23/31 (75%), Gaps = 0/31 (0%)

Frame = -2

Query 136 LEYSGAISADCNLHLPGSSNSSASASLVAGT 44

LEYSG I A CN L GS++SSASAS V GT

Sbjct 34 LEYSGTILAHCNFRLLGSNDSSASASQVTGT 64

>ref|NP_060313.3| breast carcinoma-amplified sequence 4 isoform a [Homo sapiens]

Length=211

Score = 41.6 bits (96), Expect = 2e-04

Identities = 21/31 (68%), Positives = 24/31 (78%), Gaps = 0/31 (0%)

Frame = -2

Query 139 SLEYSGAISADCNLHLPGSSNSSASASLVAG 47

++E SG I A CNL LPGSS+S ASAS VAG

Sbjct 163 NVECSGTIPARCNLRLPGSSDSPASASQVAG 193

>ref|NP_001153583.1| nitric oxide synthase, endothelial isoform 4 [Homo sapiens]

Length=629

Score = 41.2 bits (95), Expect = 2e-04

Identities = 23/44 (53%), Positives = 26/44 (60%), Gaps = 0/44 (0%)

Frame = -2

Query 154 LTLSPSLEYSGAISADCNLHLPGSSNSSASASLVAGTIGMHHHA 23

LTL P LE S I+A C+L+L SSN S S V GT G H A

Sbjct 586 LTLWPRLECSSTITAHCSLNLLDSSNPPTSTSQVVGTTGACHDA 629

>ref|XP_938432.4| PREDICTED: hypothetical protein LOC646021 [Homo sapiens]

Length=359

Score = 40.8 bits (94), Expect = 3e-04

Identities = 21/30 (70%), Positives = 22/30 (74%), Gaps = 0/30 (0%)

Frame = -2

Query 148 LSPSLEYSGAISADCNLHLPGSSNSSASAS 59

LSP LE SG I A CNL LPG S+S ASAS

Sbjct 302 LSPRLECSGVILAHCNLRLPGLSDSPASAS 331

>ref|NP_001166173.1| probable sodium-coupled neutral amino acid transporter 6 isoform

1 [Homo sapiens]

Length=521

Score = 40.8 bits (94), Expect = 3e-04

Identities = 24/39 (62%), Positives = 24/39 (62%), Gaps = 0/39 (0%)

Frame = -2

Query 154 LTLSPSLEYSGAISADCNLHLPGSSNSSASASLVAGTIG 38

L LS L SG ISA CNL LP SSN SAS VA T G

Sbjct 432 LILSHRLACSGVISAHCNLCLPDSSNPPTSASRVAETTG 470

>ref|NP_001018114.1| fumarylacetoacetate hydrolase domain-containing protein 1 isoform

1 [Homo sapiens]

Length=248

Score = 40.8 bits (94), Expect = 3e-04

Identities = 20/34 (59%), Positives = 25/34 (74%), Gaps = 0/34 (0%)

Frame = -2

Query 163 R*SLTLSPSLEYSGAISADCNLHLPGSSNSSASA 62

R LTLSP LE S AI+A C+L LPGSSN +++

Sbjct 213 RQGLTLSPKLECSSAITAHCSLELPGSSNPPSAS 246

>ref|NP_001012677.1| arginine-fifty homeobox [Homo sapiens]

Length=315

Score = 40.8 bits (94), Expect = 3e-04

Identities = 21/38 (56%), Positives = 26/38 (69%), Gaps = 0/38 (0%)

Frame = -2

Query 157 SLTLSPSLEYSGAISADCNLHLPGSSNSSASASLVAGT 44

S TL LE SG +SA C+L+LPGS++ SAS VA T

Sbjct 35 SFTLLSKLECSGTVSAYCSLNLPGSTDPPTSASRVAAT 72

>ref|NP_000865.2| interferon alpha/beta receptor 2 isoform b precursor [Homo sapiens]

ref|NP_997467.1| interferon alpha/beta receptor 2 isoform b precursor [Homo sapiens]

Length=331

Score = 39.7 bits (91), Expect = 6e-04

Identities = 17/33 (52%), Positives = 20/33 (61%), Gaps = 0/33 (0%)

Frame = -1

Query 122 CNLS*LQPPPPRFKQFFCLSLPSSWDYRHAPSC 24

C+ + LQ P KQ CLS PSSWDY+ A C

Sbjct 296 CSHNALQSETPELKQSSCLSFPSSWDYKRASLC 328

>ref|NP_001158157.1| protein THEMIS isoform 1 [Homo sapiens]

Length=680

Score = 32.0 bits (71), Expect(2) = 7e-04

Identities = 14/20 (70%), Positives = 15/20 (75%), Gaps = 0/20 (0%)

Frame = -1

Query 134 GVQWCNLS*LQPPPPRFKQF 75

GVQW +L LQP PP FKQF

Sbjct 588 GVQWRDLGSLQPLPPGFKQF 607

Score = 26.6 bits (57), Expect(2) = 7e-04

Identities = 13/22 (60%), Positives = 13/22 (60%), Gaps = 0/22 (0%)

Frame = -2

Query 91 PGSSNSSASASLVAGTIGMHHH 26

PG SASAS VAG G HH

Sbjct 602 PGFKQFSASASHVAGITGTPHH 623

>ref|NP_001138525.1| EF-hand calcium-binding domain-containing protein 5 isoform b

[Homo sapiens]

Length=856

Score = 38.9 bits (89), Expect = 0.001

Identities = 19/34 (56%), Positives = 21/34 (62%), Gaps = 0/34 (0%)

Frame = -1

Query 140 QPGVQWCNLS*LQPPPPRFKQFFCLSLPSSWDYR 39

Q GVQW N S LQPP P K+ L S+WDYR

Sbjct 810 QAGVQWRNCSSLQPPTPGLKRSSHHRLLSNWDYR 843

>ref|NP_001098016.1| ribonuclease P protein subunit p30 isoform a [Homo sapiens]

Length=322

Score = 38.9 bits (89), Expect = 0.001

Identities = 21/33 (64%), Positives = 21/33 (64%), Gaps = 0/33 (0%)

Frame = -1

Query 155 SHSVTQPGVQWCNLS*LQPPPPRFKQFFCLSLP 57

SHSVTQ GVQW NL LQP P K LSLP

Sbjct 267 SHSVTQAGVQWHNLGSLQPLPLGLKPSSHLSLP 299

>ref|XP_003119834.1| PREDICTED: zinc finger protein ENSP00000375192-like [Homo sapiens]

ref|XP_003119017.1| PREDICTED: zinc finger protein ENSP00000375192-like [Homo sapiens]

ref|XP_003120400.1| PREDICTED: zinc finger protein ENSP00000375192-like [Homo sapiens]

Length=105

Score = 38.9 bits (89), Expect = 0.001

Identities = 20/36 (56%), Positives = 22/36 (62%), Gaps = 0/36 (0%)

Frame = -2

Query 112 ADCNLHLPGSSNSSASASLVAGTIGMHHHAWLIFVF 5

A C+L L GS + S S VA TIG HH LIFVF

Sbjct 2 AHCSLDLSGSGDPPTSTSQVARTIGACHHVQLIFVF 37

>ref|NP_001129224.1| protein SGT1 isoform 2 [Homo sapiens]

Length=677

Score = 38.5 bits (88), Expect = 0.001

Identities = 17/29 (59%), Positives = 19/29 (66%), Gaps = 0/29 (0%)

Frame = -1

Query 131 VQWCNLS*LQPPPPRFKQFFCLSLPSSWD 45

VQW + LQ PPP F F CLSL S+WD

Sbjct 379 VQWRDPGLLQAPPPGFTPFICLSLLSTWD 407

>ref|NP_001030127.1| sorbin and SH3 domain-containing protein 1 isoform 4 [Homo sapiens]

Length=1151

Score = 38.1 bits (87), Expect = 0.002

Identities = 20/35 (58%), Positives = 23/35 (66%), Gaps = 0/35 (0%)

Frame = -2

Query 148 LSPSLEYSGAISADCNLHLPGSSNSSASASLVAGT 44

+SP LE SG + A C+L L SSN SAS VAGT

Sbjct 440 MSPRLECSGTVIAHCSLKLLDSSNPPTSASQVAGT 474

>ref|XP_003119043.1| PREDICTED: hypothetical protein LOC100506191 [Homo sapiens]

Length=118

Score = 37.4 bits (85), Expect = 0.003

Identities = 19/35 (55%), Positives = 22/35 (63%), Gaps = 0/35 (0%)

Frame = +3

Query 3 KNTKISHA*WCMPIVPATREAEAEELLEPGRWRLQ 107

KNTKIS A W MP++ AT E E L P R R+Q

Sbjct 84 KNTKISQAWWSMPMISATWETEVGGSLGPRRQRVQ 118

>ref|NP_001136036.1| cGMP-gated cation channel alpha-1 isoform 1 [Homo sapiens]

Length=759

Score = 37.4 bits (85), Expect = 0.003

Identities = 20/39 (52%), Positives = 24/39 (62%), Gaps = 1/39 (2%)

Frame = -2

Query 145 SPSLEYSGAISADCNLHLPGSSNSSASASLVAGTIGMHH 29

SP LE SGAISA C+LHLP SS+ + +G HH

Sbjct 6 SPRLECSGAISAHCSLHLPDSSDFQLIFVFLV-EMGFHH 43

>ref|XP_002343910.2| PREDICTED: testis-specific Y-encoded protein 2-like [Homo sapiens]

Length=140

Score = 36.6 bits (83), Expect = 0.005

Identities = 17/28 (61%), Positives = 20/28 (72%), Gaps = 0/28 (0%)

Frame = -1

Query 164 EMKSHSVTQPGVQWCNLS*LQPPPPRFK 81

EM+SH VTQ GV+W +L LQ PP FK

Sbjct 106 EMESHYVTQAGVKWHDLGSLQTLPPSFK 133

>ref|NP_065184.2| selenoprotein N isoform 1 precursor [Homo sapiens]

Length=590

Score = 36.6 bits (83), Expect = 0.005

Identities = 19/32 (60%), Positives = 20/32 (63%), Gaps = 0/32 (0%)

Frame = -1

Query 155 SHSVTQPGVQWCNLS*LQPPPPRFKQFFCLSL 60

S SVTQ GVQWC+ S LQP P CLSL

Sbjct 102 SCSVTQTGVQWCSHSSLQPQLPWLNUSSCLSL 133

>ref|NP_057728.1| proline-rich protein 16 [Homo sapiens]

Length=281

Score = 36.6 bits (83), Expect = 0.005

Identities = 18/22 (82%), Positives = 19/22 (87%), Gaps = 0/22 (0%)

Frame = -3

Query 126 VVQSQLTATSTSQVQAILLPQP 61

+ QS LTATS SQVQAILLPQP

Sbjct 1 MAQSGLTATSASQVQAILLPQP 22

>ref|NP_660326.2| nucleoredoxin-like protein 2 isoform 2 [Homo sapiens]

Length=135

Score = 36.2 bits (82), Expect = 0.007

Identities = 21/33 (64%), Positives = 22/33 (67%), Gaps = 0/33 (0%)

Frame = -2

Query 157 SLTLSPSLEYSGAISADCNLHLPGSSNSSASAS 59

SL L P LE SG I A CNL L GSS+S A AS

Sbjct 103 SLALLPRLECSGVILAHCNLCLLGSSDSLALAS 135

Query= Simvastatin Contig 26

Length=460

Score E

Sequences producing significant alignments: (Bits) Value

ref|NP_000423.2| myosin regulatory light chain 2, ventricular... 151 1e-37

ref|NP_612412.2| myosin regulatory light chain 10 [Homo sapiens] 120 2e-28

ref|NP_037424.2| myosin regulatory light chain 2, skeletal mu... 105 7e-24

ref|NP_067046.1| myosin regulatory light chain 2, atrial isof... 97.8 2e-21

ref|NP_002468.1| myosin light chain 5 [Homo sapiens] 93.2 5e-20

ref|NP_006462.1| myosin regulatory light chain 12A [Homo sapi... 73.9 3e-14

ref|NP_291024.1| myosin regulatory light chain 12B [Homo sapi... 73.9 3e-14

ref|NP_006088.2| myosin regulatory light polypeptide 9 isofor... 72.8 6e-14

ref|XP_373042.2| PREDICTED: calcium-dependent protein kinase ... 71.6 1e-13

ref|XP_001717112.1| PREDICTED: calcium-dependent protein kina... 68.6 1e-12

ref|NP_005176.1| calmodulin-like protein 3 [Homo sapiens] 54.7 2e-08

ref|NP_001159578.1| calmodulin isoform 2 [Homo sapiens] 52.8 7e-08

ref|NP_852667.1| myosin regulatory light polypeptide 9 isofor... 52.8 7e-08

ref|NP_001734.1| calmodulin [Homo sapiens] >ref|NP_008819.1| ... 52.8 7e-08

ref|NP_524147.2| myosin light polypeptide 6 isoform 2 [Homo s... 43.9 3e-05

ref|NP_004356.2| centrin-3 [Homo sapiens] 43.5 4e-05

ref|NP_002466.1| myosin light chain 6B [Homo sapiens] 41.6 2e-04

ref|NP_066299.2| myosin light polypeptide 6 isoform 1 [Homo s... 39.7 6e-04

ref|NP_619650.2| calmodulin-like protein 6 [Homo sapiens] 39.3 8e-04

ref|NP_000249.1| myosin light chain 3 [Homo sapiens] 38.9 0.001

ref|NP_524146.1| myosin light chain 1/3, skeletal muscle isof... 37.7 0.002

ref|NP_524144.1| myosin light chain 1/3, skeletal muscle isof... 37.7 0.002

ref|NP_004335.1| centrin-2 [Homo sapiens] 37.7 0.002

ALIGNMENTS

>ref|NP_000423.2| myosin regulatory light chain 2, ventricular/cardiac muscle isoform

[Homo sapiens]

Length=166

Score = 151 bits (382), Expect = 1e-37

Identities = 73/73 (100%), Positives = 73/73 (100%), Gaps = 0/73 (0%)

Frame = +2

Query 2 DPEETILNAFKVFDPEGKGVLKADYVREMLTTQAERFSKEEVDQMFAAFPPDVTGNLDYK 181

DPEETILNAFKVFDPEGKGVLKADYVREMLTTQAERFSKEEVDQMFAAFPPDVTGNLDYK

Sbjct 94 DPEETILNAFKVFDPEGKGVLKADYVREMLTTQAERFSKEEVDQMFAAFPPDVTGNLDYK 153

Query 182 NLVHIITHGEEKD 220

NLVHIITHGEEKD

Sbjct 154 NLVHIITHGEEKD 166

>ref|NP_612412.2| myosin regulatory light chain 10 [Homo sapiens]

Length=226

Score = 120 bits (302), Expect = 2e-28

Identities = 56/73 (77%), Positives = 65/73 (90%), Gaps = 0/73 (0%)

Frame = +2

Query 2 DPEETILNAFKVFDPEGKGVLKADYVREMLTTQAERFSKEEVDQMFAAFPPDVTGNLDYK 181

DPEETIL+AFKVFD EGKG +KAD ++E L TQA+RFS+EEV QMFAAFPPDV GNLDY+

Sbjct 154 DPEETILHAFKVFDTEGKGFVKADVIKEKLMTQADRFSEEEVKQMFAAFPPDVCGNLDYR 213

Query 182 NLVHIITHGEEKD 220

NL ++ITHGEEKD

Sbjct 214 NLCYVITHGEEKD 226

>ref|NP_037424.2| myosin regulatory light chain 2, skeletal muscle isoform [Homo

sapiens]

Length=169

Score = 105 bits (263), Expect = 7e-24

Identities = 46/75 (62%), Positives = 60/75 (80%), Gaps = 0/75 (0%)

Frame = +2

Query 2 DPEETILNAFKVFDPEGKGVLKADYVREMLTTQAERFSKEEVDQMFAAFPPDVTGNLDYK 181

DPE+ I AFKV DPEGKG +K ++ E+LTTQ +RFS+EE+ M+AAFPPDV GN+DYK

Sbjct 95 DPEDVITGAFKVLDPEGKGTIKKKFLEELLTTQCDRFSQEEIKNMWAAFPPDVGGNVDYK 154

Query 182 NLVHIITHGEEKD*E 226

N+ ++ITHG+ KD E

Sbjct 155 NICYVITHGDAKDQE 169

>ref|NP_067046.1| myosin regulatory light chain 2, atrial isoform [Homo sapiens]

Length=175

Score = 97.8 bits (242), Expect = 2e-21

Identities = 43/73 (59%), Positives = 59/73 (81%), Gaps = 0/73 (0%)

Frame = +2

Query 2 DPEETILNAFKVFDPEGKGVLKADYVREMLTTQAERFSKEEVDQMFAAFPPDVTGNLDYK 181

DPEE IL+AF++FDP GKGV+ D +++L TQA++FS EV+QMFA P D+ GN+DYK

Sbjct 102 DPEEAILSAFRMFDPSGKGVVNKDEFKQLLLTQADKFSPAEVEQMFALTPMDLAGNIDYK 161

Query 182 NLVHIITHGEEKD 220

+L +IITHG+EK+

Sbjct 162 SLCYIITHGDEKE 174

>ref|NP_002468.1| myosin light chain 5 [Homo sapiens]

Length=173

Score = 93.2 bits (230), Expect = 5e-20

Identities = 42/73 (58%), Positives = 56/73 (77%), Gaps = 0/73 (0%)

Frame = +2

Query 2 DPEETILNAFKVFDPEGKGVLKADYVREMLTTQAERFSKEEVDQMFAAFPPDVTGNLDYK 181

D EETILNAFK+ DP+GKG + +Y++ +L +QA++ + EEVDQMF DV GNLDYK

Sbjct 100 DAEETILNAFKMLDPDGKGKINKEYIKRLLMSQADKMTAEEVDQMFQFASIDVAGNLDYK 159

Query 182 NLVHIITHGEEKD 220

L ++ITHGEEK+

Sbjct 160 ALSYVITHGEEKE 172

>ref|NP_006462.1| myosin regulatory light chain 12A [Homo sapiens]

Length=171

Score = 73.9 bits (180), Expect = 3e-14

Identities = 34/74 (46%), Positives = 48/74 (65%), Gaps = 1/74 (1%)

Frame = +2

Query 2 DPEETILNAFKVFDPEGKGVLKADYVREMLTTQAERFSKEEVDQMFAAFPPDVTGNLDYK 181

DPE+ I NAF FD E G ++ DY+RE+LTT +RF+ EEVD+++ P D GN +Y

Sbjct 97 DPEDVIRNAFACFDEEATGTIQEDYLRELLTTMGDRFTDEEVDELYREAPIDKKGNFNYI 156

Query 182 NLVHIITHG-EEKD 220

I+ HG ++KD

Sbjct 157 EFTRILKHGAKDKD 170

>ref|NP_291024.1| myosin regulatory light chain 12B [Homo sapiens]

ref|NP_001138416.1| myosin regulatory light chain 12B [Homo sapiens]

ref|NP_001138417.1| myosin regulatory light chain 12B [Homo sapiens]

Length=172

Score = 73.9 bits (180), Expect = 3e-14

Identities = 34/74 (46%), Positives = 48/74 (65%), Gaps = 1/74 (1%)

Frame = +2

Query 2 DPEETILNAFKVFDPEGKGVLKADYVREMLTTQAERFSKEEVDQMFAAFPPDVTGNLDYK 181

DPE+ I NAF FD E G ++ DY+RE+LTT +RF+ EEVD+++ P D GN +Y

Sbjct 98 DPEDVIRNAFACFDEEATGTIQEDYLRELLTTMGDRFTDEEVDELYREAPIDKKGNFNYI 157

Query 182 NLVHIITHG-EEKD 220

I+ HG ++KD

Sbjct 158 EFTRILKHGAKDKD 171

>ref|NP_006088.2| myosin regulatory light polypeptide 9 isoform a [Homo sapiens]

Length=172

Score = 72.8 bits (177), Expect = 6e-14

Identities = 34/74 (46%), Positives = 47/74 (64%), Gaps = 1/74 (1%)

Frame = +2

Query 2 DPEETILNAFKVFDPEGKGVLKADYVREMLTTQAERFSKEEVDQMFAAFPPDVTGNLDYK 181

DPE+ I NAF FD E G + D++RE+LTT +RF+ EEVD+M+ P D GN +Y

Sbjct 98 DPEDVIRNAFACFDEEASGFIHEDHLRELLTTMGDRFTDEEVDEMYREAPIDKKGNFNYV 157

Query 182 NLVHIITHG-EEKD 220

I+ HG ++KD

Sbjct 158 EFTRILKHGAKDKD 171

>ref|XP_373042.2| PREDICTED: calcium-dependent protein kinase 7-like [Homo sapiens]

ref|XP_945499.1| PREDICTED: calcium-dependent protein kinase 7-like [Homo sapiens]

Length=377

Score = 71.6 bits (174), Expect = 1e-13

Identities = 33/74 (45%), Positives = 48/74 (65%), Gaps = 1/74 (1%)

Frame = +2

Query 2 DPEETILNAFKVFDPEGKGVLKADYVREMLTTQAERFSKEEVDQMFAAFPPDVTGNLDYK 181

DPE+ I NAF FD E G ++ DY+RE+LTT ++F+ EEVD+++ P D GN +Y

Sbjct 303 DPEDVIRNAFACFDEEATGTIQEDYLRELLTTVGDQFTDEEVDELYTEAPIDKKGNFNYI 362

Query 182 NLVHIITHG-EEKD 220

I+ HG ++KD

Sbjct 363 EFTSILKHGVKDKD 376

>ref|XP_001717112.1| PREDICTED: calcium-dependent protein kinase 7-like [Homo sapiens]

Length=377

Score = 68.6 bits (166), Expect = 1e-12

Identities = 32/74 (44%), Positives = 47/74 (64%), Gaps = 1/74 (1%)

Frame = +2

Query 2 DPEETILNAFKVFDPEGKGVLKADYVREMLTTQAERFSKEEVDQMFAAFPPDVTGNLDYK 181

DPE+ I NAF FD E G ++ DY+RE+LTT ++F+ EEVD+++ D GN +Y

Sbjct 303 DPEDVIRNAFACFDEEATGTIQEDYLRELLTTVGDQFTDEEVDELYTEASIDKKGNFNYI 362

Query 182 NLVHIITHG-EEKD 220

I+ HG ++KD

Sbjct 363 EFTSILKHGVKDKD 376

>ref|NP_005176.1| calmodulin-like protein 3 [Homo sapiens]

Length=149

Score = 54.7 bits (130), Expect = 2e-08

Identities = 26/66 (40%), Positives = 39/66 (60%), Gaps = 0/66 (0%)

Frame = +2

Query 2 DPEETILNAFKVFDPEGKGVLKADYVREMLTTQAERFSKEEVDQMFAAFPPDVTGNLDYK 181

D EE I AF+VFD +G G + A +R ++T E+ S EEVD+M A D G ++Y+

Sbjct 81 DNEEEIREAFRVFDKDGNGFVSAAELRHVMTRLGEKLSDEEVDEMIRAADTDGDGQVNYE 140

Query 182 NLVHII 199

V ++

Sbjct 141 EFVRVL 146

>ref|NP_001159578.1| calmodulin isoform 2 [Homo sapiens]

Length=113

Score = 52.8 bits (125), Expect = 7e-08

Identities = 25/67 (38%), Positives = 39/67 (59%), Gaps = 0/67 (0%)

Frame = +2

Query 2 DPEETILNAFKVFDPEGKGVLKADYVREMLTTQAERFSKEEVDQMFAAFPPDVTGNLDYK 181

D EE I AF+VFD +G G + A +R ++T E+ + EEVD+M D G ++Y+

Sbjct 45 DSEEEIREAFRVFDKDGNGYISAAELRHVMTNLGEKLTDEEVDEMIREADIDGDGQVNYE 104

Query 182 NLVHIIT 202

V ++T

Sbjct 105 EFVQMMT 111

>ref|NP_852667.1| myosin regulatory light polypeptide 9 isoform b [Homo sapiens]

Length=118

Score = 52.8 bits (125), Expect = 7e-08

Identities = 24/56 (43%), Positives = 36/56 (65%), Gaps = 1/56 (1%)

Frame = +2

Query 56 GVLKADYVREMLTTQAERFSKEEVDQMFAAFPPDVTGNLDYKNLVHIITHG-EEKD 220

G + D++RE+LTT +RF+ EEVD+M+ P D GN +Y I+ HG ++KD

Sbjct 62 GFIHEDHLRELLTTMGDRFTDEEVDEMYREAPIDKKGNFNYVEFTRILKHGAKDKD 117

>ref|NP_001734.1| calmodulin [Homo sapiens]

ref|NP_008819.1| calmodulin isoform 1 [Homo sapiens]

ref|NP_005175.2| calmodulin [Homo sapiens]

Length=149

Score = 52.8 bits (125), Expect = 7e-08

Identities = 25/67 (38%), Positives = 39/67 (59%), Gaps = 0/67 (0%)

Frame = +2

Query 2 DPEETILNAFKVFDPEGKGVLKADYVREMLTTQAERFSKEEVDQMFAAFPPDVTGNLDYK 181

D EE I AF+VFD +G G + A +R ++T E+ + EEVD+M D G ++Y+

Sbjct 81 DSEEEIREAFRVFDKDGNGYISAAELRHVMTNLGEKLTDEEVDEMIREADIDGDGQVNYE 140

Query 182 NLVHIIT 202

V ++T

Sbjct 141 EFVQMMT 147

>ref|NP_524147.2| myosin light polypeptide 6 isoform 2 [Homo sapiens]

Length=151

Score = 43.9 bits (102), Expect = 3e-05

Identities = 22/66 (34%), Positives = 37/66 (57%), Gaps = 1/66 (1%)

Frame = +2

Query 11 ETILNAFKVFDPEGKGVLKADYVREMLTTQAERFSKEEVDQMFAAFPPDVTGNLDYKNLV 190

E + +VFD EG G + +R +L T E+ ++EEV +M A D G ++Y+ LV

Sbjct 87 EDYVEGLRVFDKEGNGTVMGAEIRHVLVTLGEKMTEEEV-EMLVAGHEDSNGCINYEELV 145

Query 191 HIITHG 208

++ +G

Sbjct 146 RMVLNG 151

>ref|NP_004356.2| centrin-3 [Homo sapiens]

Length=167

Score = 43.5 bits (101), Expect = 4e-05

Identities = 22/67 (33%), Positives = 33/67 (50%), Gaps = 0/67 (0%)

Frame = +2

Query 2 DPEETILNAFKVFDPEGKGVLKADYVREMLTTQAERFSKEEVDQMFAAFPPDVTGNLDYK 181

DP E IL AFK+FD + G + +R + E S EE+ M F D G ++ +

Sbjct 98 DPHEEILKAFKLFDDDDSGKISLRNLRRVARELGENMSDEELRAMIEEFDKDGDGEINQE 157

Query 182 NLVHIIT 202

+ I+T

Sbjct 158 EFIAIMT 164

>ref|NP_002466.1| myosin light chain 6B [Homo sapiens]

Length=208

Score = 41.6 bits (96), Expect = 2e-04

Identities = 24/65 (37%), Positives = 38/65 (59%), Gaps = 2/65 (3%)

Frame = +2

Query 11 ETILNAFKVFDPEGKGVLKADYVREMLTTQAERFSKEEVDQMFAAFPPDVTGNLDYKN-L 187

E L F+VFD EG G + +R +LTT E+ ++EEV+ + A D G ++Y+ L

Sbjct 144 EDYLEGFRVFDKEGNGKVMGAELRHVLTTLGEKMTEEEVETVLAGH-EDSNGCINYEAFL 202

Query 188 VHIIT 202

HI++

Sbjct 203 KHILS 207

>ref|NP_066299.2| myosin light polypeptide 6 isoform 1 [Homo sapiens]

Length=151

Score = 39.7 bits (91), Expect = 6e-04

Identities = 22/66 (34%), Positives = 34/66 (52%), Gaps = 1/66 (1%)

Frame = +2

Query 11 ETILNAFKVFDPEGKGVLKADYVREMLTTQAERFSKEEVDQMFAAFPPDVTGNLDYKNLV 190

E + +VFD EG G + +R +L T E+ ++EEV +M A D G ++Y+ V

Sbjct 87 EDYVEGLRVFDKEGNGTVMGAEIRHVLVTLGEKMTEEEV-EMLVAGHEDSNGCINYEAFV 145

Query 191 HIITHG 208

I G

Sbjct 146 RHILSG 151

>ref|NP_619650.2| calmodulin-like protein 6 [Homo sapiens]

Length=181

Score = 39.3 bits (90), Expect = 8e-04

Identities = 20/65 (31%), Positives = 34/65 (53%), Gaps = 0/65 (0%)

Frame = +2

Query 8 EETILNAFKVFDPEGKGVLKADYVREMLTTQAERFSKEEVDQMFAAFPPDVTGNLDYKNL 187

E + AF+VFD EGKG + + ++ +L E ++ E +QM D +DY+

Sbjct 109 ESELRAAFRVFDKEGKGYIDWNTLKYVLMNAGEPLNEVEAEQMMKEADKDGDRTIDYEEF 168

Query 188 VHIIT 202

V ++T

Sbjct 169 VAMMT 173

>ref|NP_000249.1| myosin light chain 3 [Homo sapiens]

Length=195

Score = 38.9 bits (89), Expect = 0.001

Identities = 21/65 (33%), Positives = 37/65 (57%), Gaps = 2/65 (3%)

Frame = +2

Query 11 ETILNAFKVFDPEGKGVLKADYVREMLTTQAERFSKEEVDQMFAAFPPDVTGNLDYKNLV 190

E + +VFD EG G + +R +L T ER +++EV+++ A D G ++Y+ V

Sbjct 131 EDFVEGLRVFDKEGNGTVMGAELRHVLATLGERLTEDEVEKLMAG-QEDSNGCINYEAFV 189

Query 191 -HIIT 202

HI++

Sbjct 190 KHIMS 194

>ref|NP_524146.1| myosin light chain 1/3, skeletal muscle isoform isoform 3f [Homo

sapiens]

Length=150

Score = 37.7 bits (86), Expect = 0.002

Identities = 21/65 (33%), Positives = 35/65 (54%), Gaps = 2/65 (3%)

Frame = +2

Query 11 ETILNAFKVFDPEGKGVLKADYVREMLTTQAERFSKEEVDQMFAAFPPDVTGNLDYKNLV 190

E + +VFD EG G + +R +L T E+ +EEV+ + A D G ++Y+ V

Sbjct 86 EDFVEGLRVFDKEGNGTVMGAELRHVLATLGEKMKEEEVEALMAG-QEDSNGCINYEAFV 144

Query 191 -HIIT 202

HI++

Sbjct 145 KHIMS 149

>ref|NP_524144.1| myosin light chain 1/3, skeletal muscle isoform isoform 1f [Homo

sapiens]

Length=194

Score = 37.7 bits (86), Expect = 0.002

Identities = 21/65 (33%), Positives = 35/65 (54%), Gaps = 2/65 (3%)

Frame = +2

Query 11 ETILNAFKVFDPEGKGVLKADYVREMLTTQAERFSKEEVDQMFAAFPPDVTGNLDYKNLV 190

E + +VFD EG G + +R +L T E+ +EEV+ + A D G ++Y+ V

Sbjct 130 EDFVEGLRVFDKEGNGTVMGAELRHVLATLGEKMKEEEVEALMAG-QEDSNGCINYEAFV 188

Query 191 -HIIT 202

HI++

Sbjct 189 KHIMS 193

>ref|NP_004335.1| centrin-2 [Homo sapiens]

Length=172

Score = 37.7 bits (86), Expect = 0.002

Identities = 20/64 (32%), Positives = 32/64 (50%), Gaps = 1/64 (1%)

Frame = +2

Query 8 EETILNAFKVFDPEGKGVLK-ADYVREMLTTQAERFSKEEVDQMFAAFPPDVTGNLDYKN 184

+E I D EG G + D++ M +E+ +KEE+ + F F D TG + +KN

Sbjct 66 KEEIKKMISEIDKEGTGKMNFGDFLTVMTQKMSEKDTKEEILKAFKLFDDDETGKISFKN 125

Query 185 LVHI 196

L +

Sbjct 126 LKRV 129

Query= Simvastatin Contig 28

Length=155

Score E

Sequences producing significant alignments: (Bits) Value

ref|NP_001167449.1| hypothetical protein LOC283579 isoform 2 ... 36.2 0.007

ref|NP_777603.1| hypothetical protein LOC283579 isoform 1 [Ho... 36.2 0.007

ref|NP_963998.2| thromboxane A2 receptor isoform beta [Homo s... 35.8 0.009

ALIGNMENTS

>ref|NP_001167449.1| hypothetical protein LOC283579 isoform 2 [Homo sapiens]

Length=92

Score = 36.2 bits (82), Expect = 0.007

Identities = 17/18 (95%), Positives = 17/18 (95%), Gaps = 0/18 (0%)

Frame = -1

Query 155 ASASQSAGITGVSHCA*P 102

ASASQSAGITGVSHCA P

Sbjct 21 ASASQSAGITGVSHCARP 38

>ref|NP_777603.1| hypothetical protein LOC283579 isoform 1 [Homo sapiens]

Length=122

Score = 36.2 bits (82), Expect = 0.007

Identities = 17/18 (95%), Positives = 17/18 (95%), Gaps = 0/18 (0%)

Frame = -1

Query 155 ASASQSAGITGVSHCA*P 102

ASASQSAGITGVSHCA P

Sbjct 51 ASASQSAGITGVSHCARP 68

>ref|NP_963998.2| thromboxane A2 receptor isoform beta [Homo sapiens]

Length=407

Score = 35.8 bits (81), Expect = 0.009

Identities = 16/22 (73%), Positives = 19/22 (87%), Gaps = 0/22 (0%)

Frame = -1

Query 155 ASASQSAGITGVSHCA*PIFLY 90

ASAS++AGITGVSHCA P L+

Sbjct 359 ASASRAAGITGVSHCARPCMLF 380

Query= Simvastatin Contig 29

Length=175

Score E

Sequences producing significant alignments: (Bits) Value

ref|NP_853516.1| gap junction gamma-3 protein [Homo sapiens] 52.4 9e-08

ref|XP_001725136.1| PREDICTED: gap junction gamma-3 protein i... 40.0 5e-04

ref|XP_003120097.1| PREDICTED: gap junction gamma-3 protein i... 38.1 0.002

ALIGNMENTS

>ref|NP_853516.1| gap junction gamma-3 protein [Homo sapiens]

Length=279

Score = 52.4 bits (124), Expect = 9e-08

Identities = 25/25 (100%), Positives = 25/25 (100%), Gaps = 0/25 (0%)

Frame = -1

Query 76 MCGRFLRRLLAEESRRSTPVGRLLL 2

MCGRFLRRLLAEESRRSTPVGRLLL

Sbjct 1 MCGRFLRRLLAEESRRSTPVGRLLL 25

>ref|XP_001725136.1| PREDICTED: gap junction gamma-3 protein isoform 1 [Homo sapiens]

ref|XP_001718277.1| PREDICTED: gap junction gamma-3 protein isoform 1 [Homo sapiens]

ref|XP_001724553.1| PREDICTED: gap junction gamma-3 protein isoform 1 [Homo sapiens]

ref|XP_002344414.1| PREDICTED: gap junction gamma-3 protein isoform 1 [Homo sapiens]

Length=222

Score = 40.0 bits (92), Expect = 5e-04

Identities = 22/26 (85%), Positives = 22/26 (85%), Gaps = 1/26 (3%)

Frame = -1

Query 79 RMCGRFLRR-LLAEESRRSTPVGRLL 5

RMCGRFLR LLAEES STPVGRLL

Sbjct 18 RMCGRFLRWWLLAEESWHSTPVGRLL 43

>ref|XP_003120097.1| PREDICTED: gap junction gamma-3 protein isoform 2 [Homo sapiens]

ref|XP_003119091.1| PREDICTED: gap junction gamma-3 protein isoform 2 [Homo sapiens]

ref|XP_003118639.1| PREDICTED: gap junction gamma-3 protein isoform 2 [Homo sapiens]

ref|XP_003120637.1| PREDICTED: gap junction gamma-3 protein isoform 2 [Homo sapiens]

Length=207

Score = 38.1 bits (87), Expect = 0.002

Identities = 21/25 (84%), Positives = 21/25 (84%), Gaps = 1/25 (4%)

Frame = -1

Query 76 MCGRFLRR-LLAEESRRSTPVGRLL 5

MCGRFLR LLAEES STPVGRLL

Sbjct 1 MCGRFLRWWLLAEESWHSTPVGRLL 25

Query= Simvastatin Contig 31

Length=470

Score E

Sequences producing significant alignments: (Bits) Value

ref|NP_060190.2| signal-transducing adaptor protein 2 isoform... 60.1 4e-10

ref|XP_003119960.1| PREDICTED: putative uncharacterized prote... 58.5 1e-09

ref|XP_003119512.1| PREDICTED: hypothetical protein LOC100507... 56.6 5e-09

ref|XP_002344133.1| PREDICTED: hypothetical protein LOC100287... 53.1 5e-08

ref|XP_003118634.1| PREDICTED: putative uncharacterized prote... 52.8 7e-08

ref|XP_003120094.1| PREDICTED: putative uncharacterized prote... 52.8 7e-08

ref|XP_003119248.1| PREDICTED: hypothetical protein LOC100506... 52.4 9e-08

ref|NP_872601.1| histone demethylase UTY isoform 1 [Homo sapi... 50.8 3e-07

ref|XP_003118557.1| PREDICTED: histone demethylase UTY-like [... 50.4 3e-07

ref|NP_777603.1| hypothetical protein LOC283579 isoform 1 [Ho... 49.3 7e-07

ref|XP_003120115.1| PREDICTED: putative uncharacterized prote... 48.9 1e-06

ref|XP_003119968.1| PREDICTED: hypothetical protein LOC100129... 48.9 1e-06

ref|NP_001167449.1| hypothetical protein LOC283579 isoform 2 ... 47.8 2e-06

ref|NP_001166126.1| zinc finger protein 701 isoform 1 [Homo s... 47.8 2e-06

ref|NP_001158009.1| disrupted in schizophrenia 1 protein isof... 47.8 2e-06

ref|NP_116326.2| chromosome 9 open reading frame 37 [Homo sap... 47.8 2e-06

ref|XP_003119925.1| PREDICTED: histone demethylase UTY-like [... 47.4 3e-06

ref|XP_003120111.1| PREDICTED: putative uncharacterized prote... 47.0 4e-06

ref|NP_001153587.1| hypothetical protein LOC123207 isoform d ... 46.6 5e-06

ref|NP_001177143.1| inositol hexakisphosphate and diphosphoin... 46.6 5e-06

ref|XP_003119948.1| PREDICTED: hypothetical protein LOC100508... 45.4 1e-05

ref|XP_003118780.1| PREDICTED: hypothetical protein LOC100507... 45.4 1e-05

ref|NP_001158011.1| disrupted in schizophrenia 1 protein isof... 45.4 1e-05

ref|NP_002899.1| proto-oncogene c-Rel [Homo sapiens] 45.4 1e-05

ref|NP_001124384.1| suppressor of G2 allele of SKP1 homolog i... 45.1 1e-05

ref|NP_001180442.1| zinc transporter 6 isoform 1 [Homo sapiens] 44.7 2e-05

ref|NP_001180304.1| NADH dehydrogenase [ubiquinone] 1 alpha s... 44.3 2e-05

ref|NP_689672.4| hypothetical protein LOC146556 isoform 1 pre... 43.9 3e-05

ref|NP_789795.1| sulfotransferase 1C2 isoform b [Homo sapiens] 43.9 3e-05

ref|NP_001123992.1| zinc finger protein 195 isoform 1 [Homo s... 43.5 4e-05

ref|NP_001123991.1| zinc finger protein 195 isoform 2 [Homo s... 43.5 4e-05

ref|XP_003120008.1| PREDICTED: putative uncharacterized prote... 43.1 5e-05

ref|NP_001018121.1| podocalyxin isoform 1 precursor [Homo sap... 43.1 5e-05

ref|XP_002346169.1| PREDICTED: protein MOST-1-like [Homo sapi... 43.1 5e-05

ref|NP_001005735.1| serine/threonine-protein kinase Chk2 isof... 42.4 9e-05

ref|NP_001136036.1| cGMP-gated cation channel alpha-1 isoform... 41.2 2e-04

ref|NP_062553.1| putative uncharacterized protein C8orf44 [Ho... 41.2 2e-04

ref|XP_003119043.1| PREDICTED: hypothetical protein LOC100506... 40.4 3e-04

ref|XP_003120941.1| PREDICTED: ubiquitin carboxyl-terminal hy... 40.0 5e-04

ref|XP_002347843.2| PREDICTED: ubiquitin carboxyl-terminal hy... 40.0 5e-04

ref|NP_001171632.1| glycogenin-2 isoform d [Homo sapiens] 40.0 5e-04

ref|NP_003909.2| glycogenin-2 isoform b [Homo sapiens] 40.0 5e-04

ref|XP_003118843.1| PREDICTED: zinc finger protein ENSP000003... 39.7 6e-04

ref|NP_001139025.1| hypothetical protein LOC401082 [Homo sapi... 39.7 6e-04

ref|NP_006668.1| ubiquitin carboxyl-terminal hydrolase 19 iso... 38.5 0.001

ref|XP_003119710.1| PREDICTED: hypothetical protein LOC100507... 37.7 0.002

ref|XP_003119972.1| PREDICTED: hypothetical protein LOC100508... 37.4 0.003

ref|NP_001012680.1| 4F2 cell-surface antigen heavy chain isof... 37.4 0.003

ref|XP_003119834.1| PREDICTED: zinc finger protein ENSP000003... 37.0 0.004

ALIGNMENTS

>ref|NP_060190.2| signal-transducing adaptor protein 2 isoform 1 [Homo sapiens]

Length=449

Score = 60.1 bits (144), Expect = 4e-10

Identities = 31/40 (78%), Positives = 32/40 (80%), Gaps = 1/40 (2%)

Frame = +2

Query 2 ETGFHPVCQAGLELLTSGSPPTSSSQSAGWITGVSHHIRP 121

E GFH V QAGLELLTS PPTS+SQSAG ITGVSHH P

Sbjct 359 EKGFHHVAQAGLELLTSSDPPTSASQSAG-ITGVSHHTWP 397

>ref|XP_003119960.1| PREDICTED: putative uncharacterized protein NCRNA00269-like [Homo

sapiens]

ref|XP_003118569.1| PREDICTED: putative uncharacterized protein NCRNA00269-like [Homo

sapiens]

ref|XP_003120465.1| PREDICTED: putative uncharacterized protein NCRNA00269-like [Homo

sapiens]

Length=128

Score = 58.5 bits (140), Expect = 1e-09

Identities = 30/45 (67%), Positives = 34/45 (76%), Gaps = 1/45 (2%)

Frame = +2

Query 2 ETGFHPVCQAGLELLTSGSPPTSSSQSAGWITGVSHHIRPLVFYF 136

ETGFH + QAGLELLTSG PPTS+SQS G ITGVSH + + F

Sbjct 80 ETGFHHIGQAGLELLTSGDPPTSASQSVG-ITGVSHRAQQMRIIF 123

>ref|XP_003119512.1| PREDICTED: hypothetical protein LOC100507663 [Homo sapiens]

ref|XP_003118669.1| PREDICTED: hypothetical protein LOC100507663 [Homo sapiens]

ref|XP_003120701.1| PREDICTED: hypothetical protein LOC100507663 [Homo sapiens]

Length=124

Score = 56.6 bits (135), Expect = 5e-09

Identities = 30/40 (75%), Positives = 32/40 (80%), Gaps = 1/40 (2%)

Frame = -3

Query 120 GRMWWLTPVIHPALCEDEVGGLPEVRSSRPA*QTG*NPVS 1

G++WWLTPVI PAL E EVGG PEVRSSRPA T NPVS

Sbjct 72 GQVWWLTPVI-PALWEAEVGGSPEVRSSRPAWPTLRNPVS 110

>ref|XP_002344133.1| PREDICTED: hypothetical protein LOC100287593 [Homo sapiens]

ref|XP_003119679.1| PREDICTED: hypothetical protein LOC100287593 [Homo sapiens]

ref|XP_003120900.1| PREDICTED: hypothetical protein LOC100287593 [Homo sapiens]

Length=120

Score = 53.1 bits (126), Expect = 5e-08

Identities = 31/72 (44%), Positives = 44/72 (62%), Gaps = 2/72 (2%)

Frame = -2

Query 214 VFLFQIPKVHNPLERICESITF-TTLKKIKY*WPDVVAYTCNPPSTL*G*GGWTA*GQEF 38

+ L+++ ++N + +C +F TT K + W VA+T NP STL G GGW GQEF

Sbjct 49 LLLYEVYLINNQPKNLCSHFSFPTTYIKKERLWLGPVAHTYNP-STLGGRGGWITRGQEF 107

Query 37 KTSLTNRMKPRL 2

KTSL N ++P L

Sbjct 108 KTSLANMVEPCL 119

>ref|XP_003118634.1| PREDICTED: putative uncharacterized protein NCRNA00269-like [Homo

sapiens]

ref|XP_003120632.1| PREDICTED: putative uncharacterized protein NCRNA00269-like [Homo

sapiens]

Length=140

Score = 52.8 bits (125), Expect = 7e-08

Identities = 29/42 (70%), Positives = 30/42 (72%), Gaps = 1/42 (2%)

Frame = +2

Query 2 ETGFHPVCQAGLELLTSGSPPTSSSQSAGWITGVSHHIRPLV 127

E GFH V Q GLELLTS PPT +SQSAG ITGVSH P V

Sbjct 82 EAGFHHVGQDGLELLTSNDPPTLASQSAG-ITGVSHRAWPAV 122

>ref|XP_003120094.1| PREDICTED: putative uncharacterized protein NCRNA00269-like [Homo

sapiens]

ref|XP_003119097.1| PREDICTED: putative uncharacterized protein NCRNA00269-like [Homo

sapiens]

Length=140

Score = 52.8 bits (125), Expect = 7e-08

Identities = 29/42 (70%), Positives = 30/42 (72%), Gaps = 1/42 (2%)

Frame = +2

Query 2 ETGFHPVCQAGLELLTSGSPPTSSSQSAGWITGVSHHIRPLV 127

E GFH V Q GLELLTS PPT +SQSAG ITGVSH P V

Sbjct 82 EAGFHHVGQDGLELLTSNDPPTLASQSAG-ITGVSHRAWPAV 122

>ref|XP_003119248.1| PREDICTED: hypothetical protein LOC100506511 [Homo sapiens]

Length=402

Score = 52.4 bits (124), Expect = 9e-08

Identities = 29/42 (70%), Positives = 33/42 (79%), Gaps = 1/42 (2%)

Frame = +2

Query 2 ETGFHPVCQAGLELLTSGSPPTSSSQSAGWITGVSHHIRPLV 127

ET F V QAGLELL SG PPTS+SQSAG ITGV+H RP++

Sbjct 355 ETVFLHVDQAGLELLISGDPPTSASQSAG-ITGVTHWARPVL 395

>ref|NP_872601.1| histone demethylase UTY isoform 1 [Homo sapiens]

Length=1079

Score = 50.8 bits (120), Expect = 3e-07

Identities = 29/39 (75%), Positives = 30/39 (77%), Gaps = 1/39 (2%)

Frame = +2

Query 2 ETGFHPVCQAGLELLTSGSPPTSSSQSAGWITGVSHHIR 118

ETGFH V QA LELLTSG S+SQSAG ITGVSHH R

Sbjct 1042 ETGFHHVGQACLELLTSGGLLASASQSAG-ITGVSHHAR 1079

>ref|XP_003118557.1| PREDICTED: histone demethylase UTY-like [Homo sapiens]

Length=101

Score = 50.4 bits (119), Expect = 3e-07

Identities = 27/36 (75%), Positives = 29/36 (81%), Gaps = 1/36 (2%)

Frame = +2

Query 2 ETGFHPVCQAGLELLTSGSPPTSSSQSAGWITGVSH 109

ET FH V QAGL+LLTSG PP S+SQSAG I GVSH

Sbjct 59 ETRFHHVGQAGLKLLTSGDPPASASQSAG-IRGVSH 93

>ref|NP_777603.1| hypothetical protein LOC283579 isoform 1 [Homo sapiens]

Length=122

Score = 49.3 bits (116), Expect = 7e-07

Identities = 27/40 (68%), Positives = 29/40 (73%), Gaps = 1/40 (2%)

Frame = +2

Query 2 ETGFHPVCQAGLELLTSGSPPTSSSQSAGWITGVSHHIRP 121

E G H QAGLELL S +PP S+SQSAG ITGVSH RP

Sbjct 30 EMGSHYFAQAGLELLGSSNPPASASQSAG-ITGVSHCARP 68

>ref|XP_003120115.1| PREDICTED: putative uncharacterized protein NCRNA00269-like [Homo

sapiens]

ref|XP_003119111.1| PREDICTED: putative uncharacterized protein NCRNA00269-like [Homo

sapiens]

ref|XP_003118613.1| PREDICTED: putative uncharacterized protein NCRNA00269-like [Homo

sapiens]

ref|XP_003120653.1| PREDICTED: putative uncharacterized protein NCRNA00269-like [Homo

sapiens]

Length=137

Score = 48.9 bits (115), Expect = 1e-06

Identities = 25/44 (57%), Positives = 30/44 (69%), Gaps = 1/44 (2%)

Frame = +2

Query 2 ETGFHPVCQAGLELLTSGSPPTSSSQSAGWITGVSHHIRPLVFY 133

E GF + QAGL+LL S P S+SQS G ITG+SHH RP F+

Sbjct 85 EMGFRHIGQAGLKLLASSDVPISASQSVG-ITGMSHHARPAPFF 127

>ref|XP_003119968.1| PREDICTED: hypothetical protein LOC100129516 [Homo sapiens]

ref|XP_003120476.1| PREDICTED: hypothetical protein LOC100129516 [Homo sapiens]

Length=153

Score = 48.9 bits (115), Expect = 1e-06

Identities = 27/38 (72%), Positives = 29/38 (77%), Gaps = 1/38 (2%)

Frame = +2

Query 8 GFHPVCQAGLELLTSGSPPTSSSQSAGWITGVSHHIRP 121

GF V QAGLE LTSG PP S+SQSAG ITGVSH +P

Sbjct 2 GFRYVGQAGLEPLTSGDPPASTSQSAG-ITGVSHCTQP 38

>ref|NP_001167449.1| hypothetical protein LOC283579 isoform 2 [Homo sapiens]

Length=92

Score = 47.8 bits (112), Expect = 2e-06

Identities = 26/38 (69%), Positives = 28/38 (74%), Gaps = 1/38 (2%)

Frame = +2

Query 8 GFHPVCQAGLELLTSGSPPTSSSQSAGWITGVSHHIRP 121

G H QAGLELL S +PP S+SQSAG ITGVSH RP

Sbjct 2 GSHYFAQAGLELLGSSNPPASASQSAG-ITGVSHCARP 38

>ref|NP_001166126.1| zinc finger protein 701 isoform 1 [Homo sapiens]

Length=531

Score = 47.8 bits (112), Expect = 2e-06

Identities = 27/41 (66%), Positives = 29/41 (71%), Gaps = 1/41 (2%)

Frame = +2

Query 8 GFHPVCQAGLELLTSGSPPTSSSQSAGWITGVSHHIRPLVF 130

GF V Q GLEL TSG PP S+SQSAG ITGVSH +P F

Sbjct 2 GFLHVGQDGLELPTSGDPPASASQSAG-ITGVSHRTQPPCF 41

>ref|NP_001158009.1| disrupted in schizophrenia 1 protein isoform a [Homo sapiens]

Length=886

Score = 47.8 bits (112), Expect = 2e-06

Identities = 23/29 (80%), Positives = 25/29 (87%), Gaps = 0/29 (0%)

Frame = +2

Query 2 ETGFHPVCQAGLELLTSGSPPTSSSQSAG 88

ETGFH V QAGLELLTS +PP S+SQSAG

Sbjct 374 ETGFHYVGQAGLELLTSSNPPASASQSAG 402

>ref|NP_116326.2| chromosome 9 open reading frame 37 [Homo sapiens]

Length=176

Score = 47.8 bits (112), Expect = 2e-06

Identities = 26/42 (62%), Positives = 29/42 (70%), Gaps = 1/42 (2%)

Frame = +2

Query 11 FHPVCQAGLELLTSGSPPTSSSQSAGWITGVSHHIRPLVFYF 136

FH V QA LELLTS +PP S+SQS G ITG SH RP +F

Sbjct 3 FHYVAQADLELLTSSNPPASASQSTG-ITGGSHRARPGPVHF 43

>ref|XP_003119925.1| PREDICTED: histone demethylase UTY-like [Homo sapiens]

ref|XP_003120437.1| PREDICTED: histone demethylase UTY-like [Homo sapiens]

Length=101

Score = 47.4 bits (111), Expect = 3e-06

Identities = 26/36 (73%), Positives = 28/36 (78%), Gaps = 1/36 (2%)

Frame = +2

Query 2 ETGFHPVCQAGLELLTSGSPPTSSSQSAGWITGVSH 109

ET F V QAGL+LLTSG PP S+SQSAG I GVSH

Sbjct 59 ETRFRHVGQAGLKLLTSGDPPASASQSAG-IRGVSH 93

>ref|XP_003120111.1| PREDICTED: putative uncharacterized protein NCRNA00269-like [Homo

sapiens]

ref|XP_003119110.1| PREDICTED: putative uncharacterized protein NCRNA00269-like [Homo

sapiens]

ref|XP_003118626.1| PREDICTED: putative uncharacterized protein NCRNA00269-like [Homo

sapiens]

ref|XP_003120652.1| PREDICTED: putative uncharacterized protein NCRNA00269-like [Homo

sapiens]

Length=130

Score = 47.0 bits (110), Expect = 4e-06

Identities = 25/40 (63%), Positives = 30/40 (75%), Gaps = 1/40 (2%)

Frame = +2

Query 8 GFHPVCQAGLELLTSGSPPTSSSQSAGWITGVSHHIRPLV 127

GFH V QAGLE LTSG P +SQSAG IT VSH ++P++

Sbjct 88 GFHHVGQAGLEFLTSGDLPALASQSAG-ITDVSHCVQPVL 126

>ref|NP_001153587.1| hypothetical protein LOC123207 isoform d [Homo sapiens]

Length=167

Score = 46.6 bits (109), Expect = 5e-06

Identities = 25/40 (63%), Positives = 29/40 (73%), Gaps = 1/40 (2%)

Frame = +2

Query 2 ETGFHPVCQAGLELLTSGSPPTSSSQSAGWITGVSHHIRP 121

+TG + QAGLELL S P S+SQSAG ITGVSHH +P

Sbjct 122 KTGSCYIAQAGLELLASSDLPASASQSAG-ITGVSHHTQP 160

>ref|NP_001177143.1| inositol hexakisphosphate and diphosphoinositol-pentakisphosphate

kinase 1 isoform 6 [Homo sapiens]

Length=1406

Score = 46.6 bits (109), Expect = 5e-06

Identities = 27/40 (68%), Positives = 29/40 (73%), Gaps = 1/40 (2%)

Frame = +2

Query 2 ETGFHPVCQAGLELLTSGSPPTSSSQSAGWITGVSHHIRP 121

ET F V QAGLELLTS P S+SQSAG ITGVSH +P

Sbjct 1101 ETRFCHVGQAGLELLTSSDLPASASQSAG-ITGVSHRTQP 1139

>ref|XP_003119948.1| PREDICTED: hypothetical protein LOC100508257 [Homo sapiens]

ref|XP_003118917.1| PREDICTED: hypothetical protein LOC100507149 [Homo sapiens]

ref|XP_003120447.1| PREDICTED: hypothetical protein LOC100508257 [Homo sapiens]

Length=139

Score = 45.4 bits (106), Expect = 1e-05

Identities = 38/99 (39%), Positives = 48/99 (49%), Gaps = 19/99 (19%)

Frame = +2

Query 2 ETGFHPVCQAGLELLTSGSPPTSSSQSAGWITGVSHHIRPLVFYFF*GGKCNGLTNSFQG 181

ETG H V QAGL+LL S SPPT +SQSAG IT SH L K L SF+

Sbjct 33 ETGSHYVSQAGLQLLGSSSPPTLASQSAG-ITCTSHRACRL--------KHLNLKGSFKI 83

Query 182 IMDLRYLK*KDSWNFLLPIV----------RLYWSGTVY 268

+ Y K S++F + L++SG +Y

Sbjct 84 VEGFIYFKLTYSFDFHSAHIFICISGEGFFNLFYSGKIY 122

>ref|XP_003118780.1| PREDICTED: hypothetical protein LOC100507131 [Homo sapiens]

ref|XP_003120912.1| PREDICTED: hypothetical protein LOC100507131 [Homo sapiens]

Length=165

Score = 45.4 bits (106), Expect = 1e-05

Identities = 26/45 (58%), Positives = 28/45 (63%), Gaps = 1/45 (2%)

Frame = +1

Query 1 RDGVSSCLSGWS*TPDLRQSTHLILTKCWVDYRCKPPHPATSILF 135

RD VS G S TPDLR S L L KC DYRC+PP PA +LF

Sbjct 72 RDRVSLYWPGRSRTPDLRHSARLGLPKC-RDYRCEPPCPAIRLLF 115

>ref|NP_001158011.1| disrupted in schizophrenia 1 protein isoform c [Homo sapiens]

Length=755

Score = 45.4 bits (106), Expect = 1e-05

Identities = 25/41 (61%), Positives = 30/41 (74%), Gaps = 1/41 (2%)

Frame = +2

Query 2 ETGFHPVCQAGLELLTSGSPPTSSSQSAGWITGVSHHIRPL 124

E GF+ V Q GL+LLTS PP+S+SQSAG IT +SH PL

Sbjct 715 EMGFYHVDQTGLKLLTSSDPPSSASQSAG-ITDMSHCAWPL 754

>ref|NP_002899.1| proto-oncogene c-Rel [Homo sapiens]

Length=619

Score = 45.4 bits (106), Expect = 1e-05

Identities = 24/40 (60%), Positives = 25/40 (63%), Gaps = 0/40 (0%)

Frame = +2

Query 2 ETGFHPVCQAGLELLTSGSPPTSSSQSAGWITGVSHHIRP 121

ETGF V Q GLELLTSG PPT +SQSAG RP

Sbjct 309 ETGFRHVDQDGLELLTSGDPPTLASQSAGITVNFPERPRP 348

>ref|NP_001124384.1| suppressor of G2 allele of SKP1 homolog isoform SGT1B [Homo sapiens]

Length=365

Score = 45.1 bits (105), Expect = 1e-05

Identities = 24/33 (73%), Positives = 25/33 (76%), Gaps = 1/33 (3%)

Frame = +2

Query 2 ETGFHPVCQAGLELLTSGSPPTSSSQSAGWITG 100

ETGFH V QAGL+LLTS PP SQSAG ITG

Sbjct 111 ETGFHRVGQAGLQLLTSSDPPALDSQSAG-ITG 142

>ref|NP_001180442.1| zinc transporter 6 isoform 1 [Homo sapiens]

Length=501

Score = 44.7 bits (104), Expect = 2e-05

Identities = 22/36 (62%), Positives = 24/36 (67%), Gaps = 0/36 (0%)

Frame = +1

Query 1 RDGVSSCLSGWS*TPDLRQSTHLILTKCWVDYRCKP 108

RDGVS GWS TPDL+ STHL L KCW + R P

Sbjct 73 RDGVSPFWLGWSQTPDLKWSTHLGLPKCWDNRRELP 108

>ref|NP_001180304.1| NADH dehydrogenase [ubiquinone] 1 alpha subcomplex subunit 11

isoform 2 [Homo sapiens]

Length=228

Score = 44.3 bits (103), Expect = 2e-05

Identities = 24/39 (62%), Positives = 30/39 (77%), Gaps = 1/39 (2%)

Frame = +2

Query 2 ETGFHPVCQAGLELLTSGSPPTSSSQSAGWITGVSHHIR 118

+TG H V QAGL+LL S SP TS+SQSAG I G+SH ++

Sbjct 105 KTGSHCVVQAGLKLLASSSPHTSASQSAG-IIGMSHCVQ 142

>ref|NP_689672.4| hypothetical protein LOC146556 isoform 1 precursor [Homo sapiens]

Length=402

Score = 43.9 bits (102), Expect = 3e-05

Identities = 21/29 (73%), Positives = 21/29 (73%), Gaps = 0/29 (0%)

Frame = +2

Query 2 ETGFHPVCQAGLELLTSGSPPTSSSQSAG 88

ETGFH V AGLELL S PPTS SQS G

Sbjct 373 ETGFHHVAHAGLELLISRDPPTSGSQSVG 401

>ref|NP_789795.1| sulfotransferase 1C2 isoform b [Homo sapiens]

Length=307

Score = 43.9 bits (102), Expect = 3e-05

Identities = 20/28 (72%), Positives = 24/28 (86%), Gaps = 0/28 (0%)

Frame = +2

Query 2 ETGFHPVCQAGLELLTSGSPPTSSSQSA 85

ETGFH V QAGL+LL+S +PP S+SQSA

Sbjct 93 ETGFHHVAQAGLKLLSSSNPPASTSQSA 120

>ref|NP_001123992.1| zinc finger protein 195 isoform 1 [Homo sapiens]

Length=629

Score = 43.5 bits (101), Expect = 4e-05

Identities = 24/40 (60%), Positives = 27/40 (68%), Gaps = 1/40 (2%)

Frame = +2

Query 2 ETGFHPVCQAGLELLTSGSPPTSSSQSAGWITGVSHHIRP 121

E GFH QA LELL S P S+SQSAG ITGV+H +P

Sbjct 76 EMGFHHATQACLELLGSSDLPASASQSAG-ITGVNHRAQP 114

>ref|NP_001123991.1| zinc finger protein 195 isoform 2 [Homo sapiens]

Length=606

Score = 43.5 bits (101), Expect = 4e-05

Identities = 24/40 (60%), Positives = 27/40 (68%), Gaps = 1/40 (2%)

Frame = +2

Query 2 ETGFHPVCQAGLELLTSGSPPTSSSQSAGWITGVSHHIRP 121

E GFH QA LELL S P S+SQSAG ITGV+H +P

Sbjct 76 EMGFHHATQACLELLGSSDLPASASQSAG-ITGVNHRAQP 114

>ref|XP_003120008.1| PREDICTED: putative uncharacterized protein NCRNA00269-like [Homo

sapiens]

ref|XP_003118926.1| PREDICTED: putative uncharacterized protein NCRNA00269-like [Homo

sapiens]

ref|XP_003120534.1| PREDICTED: putative uncharacterized protein NCRNA00269-like [Homo

sapiens]

Length=123

Score = 43.1 bits (100), Expect = 5e-05

Identities = 24/40 (60%), Positives = 26/40 (65%), Gaps = 1/40 (2%)

Frame = +2

Query 2 ETGFHPVCQAGLELLTSGSPPTSSSQSAGWITGVSHHIRP 121

ETGFH V QAGL+ LTS P + SAG ITGVSH P

Sbjct 42 ETGFHRVGQAGLDFLTSSDLPALACPSAG-ITGVSHRAWP 80

>ref|NP_001018121.1| podocalyxin isoform 1 precursor [Homo sapiens]

Length=558

Score = 43.1 bits (100), Expect = 5e-05

Identities = 22/29 (76%), Positives = 23/29 (80%), Gaps = 0/29 (0%)

Frame = +2

Query 2 ETGFHPVCQAGLELLTSGSPPTSSSQSAG 88

ET FH V QAGLELLTSG PT +SQSAG

Sbjct 237 ETVFHHVSQAGLELLTSGDLPTLASQSAG 265

>ref|XP_002346169.1| PREDICTED: protein MOST-1-like [Homo sapiens]

ref|XP_003118652.1| PREDICTED: protein MOST-1-like [Homo sapiens]

ref|XP_003120676.1| PREDICTED: protein MOST-1-like [Homo sapiens]

Length=99

Score = 43.1 bits (100), Expect = 5e-05

Identities = 24/34 (71%), Positives = 26/34 (77%), Gaps = 3/34 (8%)

Frame = -3

Query 123 SGRMW--WLTPVIHPALCEDEVGGLPEVRSSRPA 28

S R W WLTPVI PAL + E GGLPE+RSSRPA

Sbjct 63 SCRHWARWLTPVI-PALWKAEAGGLPELRSSRPA 95

>ref|NP_001005735.1| serine/threonine-protein kinase Chk2 isoform c [Homo sapiens]

Length=586

Score = 42.4 bits (98), Expect = 9e-05

Identities = 24/41 (59%), Positives = 27/41 (66%), Gaps = 1/41 (2%)

Frame = +2

Query 2 ETGFHPVCQAGLELLTSGSPPTSSSQSAGWITGVSHHIRPL 124

ET V Q+ LELL S PP S+SQSAG I GV HH RP+

Sbjct 107 ETESGHVTQSDLELLLSSDPPASASQSAG-IRGVRHHPRPV 146

>ref|NP_001136036.1| cGMP-gated cation channel alpha-1 isoform 1 [Homo sapiens]

Length=759

Score = 41.2 bits (95), Expect = 2e-04

Identities = 21/29 (73%), Positives = 22/29 (76%), Gaps = 0/29 (0%)

Frame = +2

Query 2 ETGFHPVCQAGLELLTSGSPPTSSSQSAG 88

E GFH V QAGLELL S PTS+SQSAG

Sbjct 38 EMGFHHVGQAGLELLISSDLPTSASQSAG 66

>ref|NP_062553.1| putative uncharacterized protein C8orf44 [Homo sapiens]

Length=159

Score = 41.2 bits (95), Expect = 2e-04

Identities = 23/39 (59%), Positives = 26/39 (67%), Gaps = 1/39 (2%)

Frame = -3

Query 120 GRMWWLTPVIHPALCEDEVGGLPEVRSSRPA*QTG*NPV 4

GR WL PVI PAL E + G PEVRSS+PA T NP+

Sbjct 36 GRARWLMPVI-PALWEAKAGRSPEVRSSKPAWPTWRNPI 73

>ref|XP_003119043.1| PREDICTED: hypothetical protein LOC100506191 [Homo sapiens]

Length=118

Score = 40.4 bits (93), Expect = 3e-04

Identities = 24/40 (60%), Positives = 25/40 (63%), Gaps = 1/40 (2%)

Frame = -3

Query 120 GRMWWLTPVIHPALCEDEVGGLPEVRSSRPA*QTG*NPVS 1

G WWL PVI AL E + GG E RSSRPA T NPVS

Sbjct 44 GCAWWLVPVIL-ALGEAKAGGSLEARSSRPAWPTWRNPVS 82

>ref|XP_003120941.1| PREDICTED: ubiquitin carboxyl-terminal hydrolase 32-like [Homo

sapiens]

Length=790

Score = 40.0 bits (92), Expect = 5e-04

Identities = 23/40 (58%), Positives = 27/40 (68%), Gaps = 1/40 (2%)

Frame = +2

Query 20 VCQAGLELLTSGSPPTSSSQSAGWITGVSHHIRPLVFYFF 139

V QAGLELL S SP +SQSAG ITG+SH +V + F

Sbjct 633 VAQAGLELLVSSSPSALASQSAG-ITGMSHCTWLVVLFLF 671

>ref|XP_002347843.2| PREDICTED: ubiquitin carboxyl-terminal hydrolase 32-like isoform

1, partial [Homo sapiens]

Length=780

Score = 40.0 bits (92), Expect = 5e-04

Identities = 23/40 (58%), Positives = 27/40 (68%), Gaps = 1/40 (2%)

Frame = +2

Query 20 VCQAGLELLTSGSPPTSSSQSAGWITGVSHHIRPLVFYFF 139

V QAGLELL S SP +SQSAG ITG+SH +V + F

Sbjct 630 VAQAGLELLVSSSPSALASQSAG-ITGMSHCTWLVVLFLF 668

>ref|NP_001171632.1| glycogenin-2 isoform d [Homo sapiens]

Length=430

Score = 40.0 bits (92), Expect = 5e-04

Identities = 20/29 (69%), Positives = 22/29 (76%), Gaps = 0/29 (0%)

Frame = +2

Query 2 ETGFHPVCQAGLELLTSGSPPTSSSQSAG 88

ET FH QAGLELL S + PTS+SQSAG

Sbjct 3 ETEFHHGAQAGLELLRSSNSPTSASQSAG 31

>ref|NP_003909.2| glycogenin-2 isoform b [Homo sapiens]

Length=501

Score = 40.0 bits (92), Expect = 5e-04

Identities = 20/29 (69%), Positives = 22/29 (76%), Gaps = 0/29 (0%)

Frame = +2

Query 2 ETGFHPVCQAGLELLTSGSPPTSSSQSAG 88

ET FH QAGLELL S + PTS+SQSAG

Sbjct 3 ETEFHHGAQAGLELLRSSNSPTSASQSAG 31

>ref|XP_003118843.1| PREDICTED: zinc finger protein ENSP00000375192-like [Homo sapiens]

ref|XP_003120983.1| PREDICTED: zinc finger protein ENSP00000375192-like [Homo sapiens]

Length=245

Score = 39.7 bits (91), Expect = 6e-04

Identities = 19/28 (68%), Positives = 20/28 (72%), Gaps = 0/28 (0%)

Frame = +2

Query 2 ETGFHPVCQAGLELLTSGSPPTSSSQSA 85

ETGFH QAG ELLTS PP +SQSA

Sbjct 159 ETGFHHFDQAGFELLTSSDPPALASQSA 186

>ref|NP_001139025.1| hypothetical protein LOC401082 [Homo sapiens]

Length=95

Score = 39.7 bits (91), Expect = 6e-04

Identities = 22/41 (54%), Positives = 27/41 (66%), Gaps = 1/41 (2%)

Frame = +2

Query 2 ETGFHPVCQAGLELLTSGSPPTSSSQSAGWITGVSHHIRPL 124

E G H V QAGLELL S + PTS+ Q+AG TG+ H P+

Sbjct 14 EDGSHYVAQAGLELLGSSNSPTSAYQAAG-TTGLHHCTPPI 53

>ref|NP_006668.1| ubiquitin carboxyl-terminal hydrolase 19 isoform 4 [Homo sapiens]

Length=1318

Score = 38.5 bits (88), Expect = 0.001

Identities = 22/39 (57%), Positives = 24/39 (62%), Gaps = 1/39 (2%)

Frame = +2

Query 2 ETGFHPVCQAGLELLTSGSPPTSSSQSAGWITGVSHHIR 118

ETG V QAGLE L SG P S+S +AG ITG H R

Sbjct 42 ETGSRYVAQAGLEPLASGDPSASASHAAG-ITGSRHRTR 79

>ref|XP_003119710.1| PREDICTED: hypothetical protein LOC100507131 [Homo sapiens]

Length=165

Score = 37.7 bits (86), Expect = 0.002

Identities = 23/45 (52%), Positives = 26/45 (58%), Gaps = 1/45 (2%)

Frame = +1

Query 1 RDGVSSCLSGWS*TPDLRQSTHLILTKCWVDYRCKPPHPATSILF 135

R+ VS G S DLR S L L KC DYRC+PP PA +LF

Sbjct 72 RNRVSLYWPGRSRYSDLRHSARLGLPKCR-DYRCEPPCPAIRLLF 115

>ref|XP_003119972.1| PREDICTED: hypothetical protein LOC100508228 [Homo sapiens]

ref|XP_003118923.1| PREDICTED: hypothetical protein LOC100506486 [Homo sapiens]

ref|XP_003120486.1| PREDICTED: hypothetical protein LOC100508228 [Homo sapiens]

Length=125

Score = 37.4 bits (85), Expect = 0.003

Identities = 21/34 (62%), Positives = 24/34 (71%), Gaps = 1/34 (2%)

Frame = +2

Query 8 GFHPVCQAGLELLTSGSPPTSSSQSAGWITGVSH 109

GFH V Q GL+L TSG P S+SQS G IT V+H

Sbjct 16 GFHHVVQVGLKLPTSGDLPASASQS-GEITCVNH 48

>ref|NP_001012680.1| 4F2 cell-surface antigen heavy chain isoform b [Homo sapiens]

Length=631

Score = 37.4 bits (85), Expect = 0.003

Identities = 21/33 (64%), Positives = 23/33 (70%), Gaps = 1/33 (3%)

Frame = +2

Query 2 ETGFHPVCQAGLELLTSGSPPTSSSQSAGWITG 100

ETGFH V QA +E LTS P S+S SAG ITG

Sbjct 70 ETGFHHVSQADIEFLTSIDPTASASGSAG-ITG 101

>ref|XP_003119834.1| PREDICTED: zinc finger protein ENSP00000375192-like [Homo sapiens]

ref|XP_003119017.1| PREDICTED: zinc finger protein ENSP00000375192-like [Homo sapiens]

ref|XP_003120400.1| PREDICTED: zinc finger protein ENSP00000375192-like [Homo sapiens]

Length=105

Score = 37.0 bits (84), Expect = 0.004

Identities = 18/29 (63%), Positives = 19/29 (66%), Gaps = 0/29 (0%)

Frame = +2

Query 2 ETGFHPVCQAGLELLTSGSPPTSSSQSAG 88

ETGFH V LELL S PPT +SQS G

Sbjct 40 ETGFHHVAPLVLELLDSSDPPTLASQSGG 68

Database: Homo sapiens RefSeq protein

Posted date: Nov 24, 2010 5:42 PM

Number of letters in database: 18,406,605

Number of sequences in database: 34,071

Lambda K H

0.318 0.134 0.401

Gapped

Lambda K H

0.267 0.0410 0.140

Matrix: BLOSUM62

Gap Penalties: Existence: 11, Extension: 1

Number of Sequences: 34071

Number of Hits to DB: 53045222

Number of extensions: 1390014

Number of successful extensions: 4012

Number of sequences better than 0.01: 34

Number of HSP's better than 0.01 without gapping: 0

Number of HSP's gapped: 3982

Number of HSP's successfully gapped: 101

Length of database: 18406605

T: 12

A: 40

X1: 16 (7.3 bits)

X2: 38 (14.6 bits)

X3: 64 (24.7 bits)

S1: 41 (20.4 bits)
