# Supplementary material for: Simvastatin Sodium Salt and Fluvastatin Interact with Human Gap Junction Gamma-3 Protein
Source: PLoS One. 2016 Feb 10;11(2):e0148266. doi: 10.1371/journal.pone.0148266 (PMC4749215; doi:10.1371/journal.pone.0148266)
Supplement: S5 Table — Data from Human Protein Atlas using keyword GJC3, http://www.proteinatlas.org/ENSG00000176402-GJC3/tissue accessed 18 May 2015. §RNA-Seq data expressed as number of Fragments Per Kilobase gene model and Million reads (FPKM): Not detected 0–1; Low 1–10; Medium 10–50; High >50. (DOCX) [file pone.0148266.s024.docx]

| **RNA expression (FPKM)^§^** | **Organ system** | **Protein localization score** |
| --- | --- | --- |
| ++ | *Liver and pancreas* |  |
|  | Liver | Low |
|  | Gallbladder | High |
| ++++ | Pancreas | High |
|  | *Digestive tract* |  |
| n/a | Oral mucosa | Medium |
| ++ | Salivary gland | High |
|  | Esophegus | High |
|  | Stomach | High |
|  | Duodenum | High |
|  | Small intestine | High |
|  | Appendix | Medium |
|  | Colon | High |
|  | Rectum | High |
|  | *Urinary tract* |  |
| ++ | Kidney | Medium |
|  | Urinary bladder | Medium |
|  | *Male reproductive system* |  |
| ++ | Testis | Medium |
| n/a | Epididymis | Medium |
| ++++ | Prostate | Medium |
| n/a | Seminal vesicle | Medium |
|  | *Breast and female reproductive system* |  |
| n/a | Breast | High |
| n/a | Vagina | High |
| n/a | Cervix, uterine | High |
|  | Endometrium | Medium |
|  | Fallopian tube | Medium |
|  | Ovary | Medium |
| ++ | Placenta | High |
|  | *Skin and soft tissues* |  |
| ++ | Skin | High |
| ++ | Adipose tissue | *nil* |
|  | Skeletal muscle | *nil* |
|  | Smooth muscle | Low |
| n/a | Soft Tissue | Medium |
|  | *Blood and immune system (hematopoietic)* |  |
|  | Bone marrow | High |
|  | Lymph node | Medium |
|  | Tonsil | High |
|  | Spleen | Medium |
|  | *Central nervous system* |  |
|  | Cerebral cortex | High |
|  | Hippocampus | High |
|  | Lateral ventricle | Low |
|  | Cerebellum | High |
|  | *Endocrine glands* |  |
|  | Thyroid gland | High |
|  | Parathyroid gland | Medium |
|  | Adrenal gland | Medium |
|  | *Respiratory system (lung)* |  |
|  | Nasopharynx | High |
|  | Bronchus | Medium |
|  | Lung | Medium |
|  | *Cardiovascular system* |  |
|  | Heart muscle | Medium |
